# Minimal Change Disease: Pathogenetic Insights from Glomerular Proteomics

**DOI:** 10.3390/ijms25115613

**Published:** 2024-05-21

**Authors:** Andrada Alina Bărar, Ioana-Ecaterina Pralea, Yuriy Maslyennikov, Raluca Munteanu, Ioana Berindan-Neagoe, Radu Pîrlog, Ioana Rusu, Andreea Nuțu, Crina Claudia Rusu, Diana Tania Moldovan, Alina Ramona Potra, Dacian Tirinescu, Maria Ticala, Florin Ioan Elec, Cristina Adela Iuga, Ina Maria Kacso

**Affiliations:** 1 Department of Nephrology, Faculty of Medicine, “Iuliu Hațieganu” University of Medicine and Pharmacy, 400012 Cluj-Napoca, Romania; barar_andrada_alina@elearn.umfcluj.ro (A.A.B.); maslyennikov_yuriy@elearn.umfcluj.ro (Y.M.); claudia.rusu@umfcluj.ro (C.C.R.); diana.moldovan@umfcluj.ro (D.T.M.); alina.potra@umfcluj.ro (A.R.P.); tirinescu.dacian@umfcluj.ro (D.T.); cosa.maria@umfcluj.ro (M.T.); maria.kacso@umfcluj.ro (I.M.K.); 2 Department of Proteomics and Metabolomics, Research Center for Advanced Medicine–MedFuture, “Iuliu Hațieganu” University of Medicine and Pharmacy Cluj-Napoca, Louis Pasteur Street 4-6, 400349 Cluj-Napoca, Romania; pralea.ioana@umfcluj.ro; 3 Department of In Vivo Studies, Research Center for Advanced Medicine–MedFuture, “Iuliu Hațieganu” University of Medicine and Pharmacy Cluj-Napoca, Louis Pasteur Street 6, 400349 Cluj-Napoca, Romania; muresan.raluca@umfcluj.ro; 4 Research Center for Functional Genomics, Biomedicine and Translational Medicine, “Iuliu Hațieganu” University of Medicine and Pharmacy, 400337 Cluj-Napoca, Romania; ioananeagoe29@gmail.com (I.B.-N.); pirlog.radu@yahoo.com (R.P.); andreeanutu.an@gmail.com (A.N.); 5 Department of Pathology, Regional Institute of Gastroenterology and Hepatology, 400394 Cluj-Napoca, Romania; ioana.russu@yahoo.com; 6 Department of Urology, Faculty of Medicine, “Iuliu Hațieganu” University of Medicine and Pharmacy, 400012 Cluj-Napoca, Romania; florinelec@elearn.umfcluj.ro; 7 Department of Pharmaceutical Analysis, Faculty of Pharmacy, “Iuliu Hațieganu” University of Medicine and Pharmacy, 400349 Cluj-Napoca, Romania

**Keywords:** proteomics, minimal change disease, podocyte cytoskeleton, laser capture microdissection, tandem mass spectrometry

## Abstract

The mechanism underlying podocyte dysfunction in minimal change disease (MCD) remains unknown. This study aimed to shed light on the potential pathophysiology of MCD using glomerular proteomic analysis. Shotgun proteomics using label-free quantitative mass spectrometry was performed on formalin-fixed, paraffin-embedded (FFPE) renal biopsies from two groups of samples: control (CTR) and MCD. Glomeruli were excised from FFPE renal biopsies using laser capture microdissection (LCM), and a single-pot solid-phase-enhanced sample preparation (SP3) digestion method was used to improve yield and protein identifications. Principal component analysis (PCA) revealed a distinct separation between the CTR and MCD groups. Forty-eight proteins with different abundance between the two groups (*p*-value ≤ 0.05 and |FC| ≥ 1.5) were identified. These may represent differences in podocyte structure, as well as changes in endothelial or mesangial cells and extracellular matrix, and some were indeed found in several of these structures. However, most differentially expressed proteins were linked to the podocyte cytoskeleton and its dynamics. Some of these proteins are known to be involved in focal adhesion (NID1 and ITGA3) or slit diaphragm signaling (ANXA2, TJP1 and MYO1C), while others are structural components of the actin and microtubule cytoskeleton of podocytes (ACTR3 and NES). This study suggests the potential of mass spectrometry-based shotgun proteomic analysis with LCM glomeruli to yield valuable insights into the pathogenesis of podocytopathies like MCD. The most significantly dysregulated proteins in MCD could be attributable to cytoskeleton dysfunction or may be a compensatory response to cytoskeleton malfunction caused by various triggers.

## 1. Introduction

The only histological abnormality seen in a kidney biopsy analysis in minimal change disease (MCD) is podocyte foot process effacement (FPE) on electron microscopy, while the glomeruli appear normal or almost normal on light microscopy [1,2]. The mechanism of proteinuria in MCD is incompletely understood [2,3], but regardless of the initial insult, podocyte dysfunction is the main cause of alteration of the glomerular filtration barrier. Over the last two decades, comprehensive research efforts have provided important insights into podocyte biology [2,4,5].

Podocytes are terminally differentiated cells with a major role in permselectivity of glomerular filtration and have a complex structure adapted for this function. Their cell body branches into primary, secondary, and tertiary interdigitating foot processes (FPs) and contains a complex cytoskeleton that is anchored to the glomerular basement membrane (GBM) at the level of focal adhesion (FA) and at the distal end of the tertiary FP to the proteins of the slit diaphragm (SD). Both the SD and FA function as mechanical anchors and major signaling sites for the cytoskeleton [6], which is a highly adaptable and dynamic structure. While microtubules and intermediate filaments predominate in the cell body and primary cell processes, longitudinal bundles of microfilaments containing actin, myosin, and alpha-actinin are seen in the FPs [7,8,9,10].

Podocytes do not divide; therefore, rearrangements of the podocyte cytoskeleton assure their adaptability in the face of injury. Foot process effacement has long been associated with proteinuria and involves simplification and flattening of the apical actin meshwork at the SD with replacement of SD at the base of the tertiary FP by occluding the junction [8,11]. This initial rapid and reversible phase is then followed at a later stage by retraction of FPs into the body of the podocyte. These modifications are triggered by signals from both the SD and FA. In focal diseases with limited podocyte injury, the switch to this “motility” phenotype aims to prevent further podocyte detachment. FPE is a consequence of cytoskeleton rearrangement made in order to strengthen adhesions to the GBM. Therefore, filtration selectivity is sacrificed for podocyte preservation [8,12,13]. However, in genetic or diffuse forms of proteinuric diseases, dysfunction of the cytoskeleton itself can be the cause of both FPE and proteinuria. A hypothesis proven by several forms of hereditary nephrotic syndrome with defective or absent podocyte proteins or by knockout in vivo models is that MCD belongs to this category of disease [2,14,15,16]. Other putative factors involved in malfunctioning of the cytoskeleton remain to be discovered. The absence of immune complexes and inflammatory cells in the glomeruli points towards circulating factors, most likely produced by T cells [2,17,18,19,20].

Data from proteomic studies on MCD, however, are very scarce. Transcriptomic studies have previously suggested that cells from MCD displayed an inflammatory signature apparently governed by IL1 and IL7 [21]. Kidney tissue transcriptomic profile-based clustering identified tumor necrosis factor activation variability in MCD and in focal and segmental glomerulosclerosis (FSGS) [22]. A transcriptomic multi-approach bioinformatics analysis found dysregulation of cell adhesion complexes in patients with nephrotic syndrome (MCD, FSGS, or membranous nephropathy (MN)) [23]. Urine proteome studies attempted to find potential biomarkers for MCD. Urine proteome profiles were shown to differentiate between different types of nephrotic syndrome (MCD, FSGS, MN) [24,25]. Specific proteins (C9, CD14, SERPINA1, apolipoprotein A or urinary β2-MG 1) or panels of proteins have been proposed as potential urinary biomarkers in MCD [25,26,27].

However, there are only two papers reporting on tissue liquid chromatography–tandem mass spectrometry (LC–MS/MS) after laser capture microdissection (LCM) in MCD, both published only in abstract form. The first suggests activation of innate immune pathways with loss of extracellular matrix and basement membrane-specific components [28]. From the second abstract comparing MCD to FSGS, we understand that proteins regulating cell–cell and cell–matrix adhesion and differentiation were upregulated in primary FSGS, with immune regulatory pathways (predominantly complement system) being upregulated in secondary FSGS compared to MCD [29].

Proteomic analysis of cultured immortalized podocytes offered insights into the complexity of these terminally differentiated cells: deep mapping identified almost 9000 different proteins with highly expressed proteasome activity in the undifferentiated state and markedly increased expression of lysosomal proteins in the differentiated state [30]. Notably, there are significant differences between proteome analysis of the cultured podocytes and that from in vivo models. In the latter, a preference for phosphorylation of actin filament-associated proteins in the differentiated state and a perturbation of synthesis of mitochondrial proteins in disease-susceptible conditions were identified [30,31]. A proteomic approach applied to kidney tissue is an interesting tool for research, as it directly reflects tissular changes that might be linked to a pathological condition [32].

Tissue proteomics from in vivo-induced podocyte disease data derive from the study of animal models of FSGS in which intracellular signaling pathways such as Jak-Stat, TCA cycle, mTOR pathway, mechanical stress responses (e.g., involving filamin B) and other cytoskeleton components are modified [33,34,35]. Data on MCD from in vivo models are nevertheless lacking.

Recent advances in proteomic techniques such as utilizing LC–MS/MS after LCM have enabled proteomic studies in human kidney biopsy tissue. Results from such studies have enhanced our comprehension of glomerular disorders such as MN, amyloidosis, C3 glomerulopathy or fibrillary glomerulonephritis [36]. We aimed to apply this method to gain more insights into the possible pathophysiology of MCD.

## 2. Results

### 2.1. Characteristics of the Study Participants

Clinical and laboratory parameter of the patients and controls are presented in Supplementary Table S1. All continuous data are presented as means ± standard deviation (SD). The average age of the MCD participants was 43 ± 22 years, with 60% being male. Serum creatinine levels in the MCD group were 80.79 ± 25.72 (μmol/L), serum albumin was 1.86 ± 0.75 g/dL, whereas total serum protein was 4.14 ± 0.99 g/dL in the MCD group. In addition, the levels of proteinuria were 14.75 ± 8.02 g/24 h. Standard initial treatment was corticotherapy. If no response was obtained in 16 weeks, alternative immunosuppression was introduced (calcineurin inhibitors or cyclophosphamide). All patients were in stable clinical remission at 6 months. Two of them experienced relapse that was approached with a similar therapeutic strategy, with remission. The MCD and CTR groups exhibited significant differences in serum urea, albumin, and total protein levels (*p* < 0.05, two-tailed independent *t*-test, Supplementary Table S1).

### 2.2. LC–MS/MS Analysis

**Figure 1:**
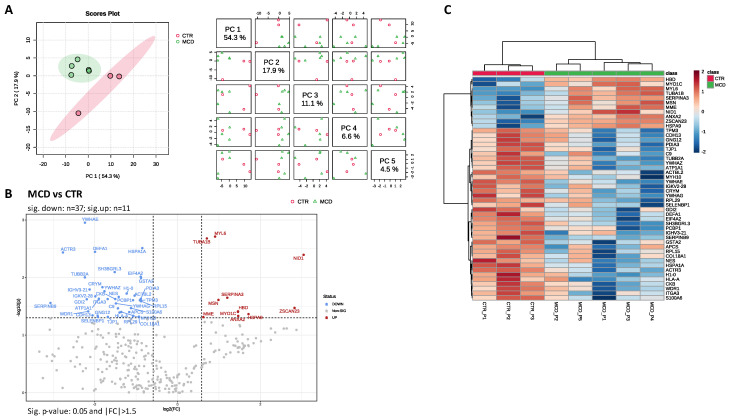
Differential expression analysis results. (**A**) Principal component analysis (PCA): (left) score plot between the selected PCs; (right) pairwise score plots between the first five PCs; the explained variance of each PC is shown in the corresponding diagonal cell; (**B**) volcano plot of differentially expressed proteins (*t*-test, independent unequal variance, *p* ≤ 0.05 and |FC| > 1.5) in MCD group vs. CTR; Red dots represent differentially expressed proteins with higher abundance in MCD group, while the blue dots correspond to with higher abundance in CTR group; black proteins showed no significant difference between groups; (**C**) proteins differentially expressed between MCD and CTR samples depicted as a heatmap of hierarchical cluster analysis results (*t*-test, independent unequal variance, *p* ≤ 0.05; Euclidean distance and clustering algorithm using averages).

A tissue proteomic profile was acquired from a total of eight FFPE renal tissue samples divided into two groups (CTR, *n* = 3 and MCD, *n* = 5). Comparative analysis resulted in the identification of 321 proteins with quantitative values based on 2546 identified peptides. The Progenesis QIp dataset was further processed by filtering out reverse sequences (*n* = 9; FDR dataset = 2.80%) and yeast ADH-1 (P00330). Further, technical replicates were averaged, and the final report contained 311 proteins identified with at least 1 unique peptide (Supplementary Table S2, Supplementary Information File S1). For differential proteomic analysis, the Metaboanalyst 5.0 (version 2023-06-23) one-factor statistical analysis module was used as stated in the Materials and Methods section. After data post-processing, the final list of total proteins was reduced to 305 (Supplementary Table S3). Principal component analysis (PCA) was implemented for data overview and pattern discovery (Figure 1A). PCA chemometrics analysis performed using all 305 proteins was able to explain more than 80% of the protein variation via three principal components (PCs), PC1 comprising 54.3% of the variation showing clear separation between samples from the CTR and MCD groups.

Forty-eight glomerular proteins with differential abundance between the two groups were identified based on *t*-tests (independent, two-tailed, unequal variance, *p* ≤ 0.05). The proteome profile across groups of *t*-test-significant proteins is presented in Figure 1B,C as a volcano plot and heatmap. The MCD group presented 11 proteins with significantly higher abundance than in CTRs (Supplementary Table S4).

### 2.3. PPI Network of MCD Related Proteins

Protein–protein interaction analysis with STRING (https://string-db.org/ accessed on 5 January 2024) indicated that the significantly different proteins highlighted by the differential expression analysis had more interactions among themselves than what would be expected (for a random set of proteins of the same size and degree of distribution drawn from the genome), as reflected in the PPI enrichment *p*-value of 7.66 × 10^−5^.

**Figure 2:**
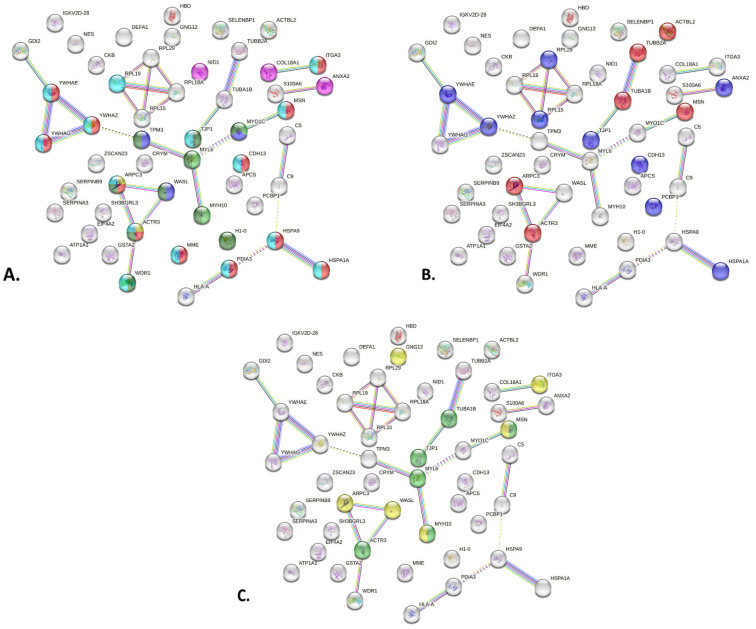
STRING network enrichment analysis using the proteins differentially expressed between MCD and CTR samples. (**A**) Enriched terms using the Compartments subcellular localization database. (**B**) Enriched terms using Gene Ontology Molecular Function database. (**C**) Enriched pathways highlighted by KEGG database.

STRING network enrichment analysis [37] results are depicted in Supplementary Table S5 and Figure 2. Enrichment analysis conducted using the Compartments database revealed proteins with connections and associations with major networks linked to podocytes and GBM in terms of subcellular localization (Supplementary Table S5, Figure 2A): Arp2/3 protein complex (Gene Ontology Cellular Component (GOCC):0005885, strength 1.93) depicted in yellow (ARPC3, ACTR3); myosin complex (GOCC:0016459, strength 1.27) shown in light green (MYH10, MYL6, MYO1C); basement membrane (GOCC:0005604, strength 1.27) pink colored (NID1, ANXA2, COL18A1); actin filament (GOCC:0005884, strength 1.24) shown in purple (WASL, MYO1C, TPM3); focal adhesion (GOCC:0005925, strength 1.23) represented in red (ARPC3, ACTR3, YWHAE, CDH13, HSPA9, PDIA3, YWHAG, ITGA3, MSN, HSPA1A, YWHAZ, MME); actin cytoskeleton (GOCC:0015629, strength 1.02) depicted in dark green (WASL, ARPC3, ACTR3, H1-0, TJP1, MYH10, WDR1, MYL6, MYO1C, TPM3) and anchoring junction (GOCC:0070161, strength 1.01) depicted in light blue (RPL19, ARPC3, ACTR3, YWHAE, CDH13, HSPA9, PDIA3, YWHAG, ITGA3, TJP1, MSN, HSPA1A, YWHAZ, MME, WDR1). Based on the GO Molecular Function database (Figure 2B), the majority of the enriched terms were linked to the structure and stability of the podocyte: proteins within the dataset were found to be structural constituent of cytoskeleton (GO:0005200, strength 1.33), depicted in red (ARPC3, ACTR3, TUBA1B, MSN, TUBB2A, ACTBL2), and many had cadherin-binding function (GO:0045296, strength 1.01), depicted in purple (YWHAE, CDH13, RPL29, PCBP1, RPL15, ANXA2, TJP1, HSPA1A, YWHAZ), which ultimately also relates to cytoskeleton function. Furthermore, many proteins were interconnected, as shown in Figure 2B and Supplementary Table S5, being part of the finely tuned dynamic structure of the active cytoskeleton. The KEGG database (Figure 2C) highlighted tight junction pathways (has04530, strength 1.16), depicted in green (TUBA1B, TJP1, MYL6, MYH10, ACTR3, MSN), and actin cytoskeleton regulation (has04810, strength 1.04), depicted in yellow (GNG12, ITGA3, MSN, ARPC3, WASL, MYH10).

Some of the proteins mentioned in the databases are associated with pathological conditions that are not related to MCD. We did not find a pertinent explanation that could link them to disturbances in MCD. However, the majority could be integrated from a pathological point of view and distilled to SD, FA, and other structural components of the actin and microtubule cytoskeleton of podocytes.

All significantly different proteins were further characterized using the Human Protein Atlas Database [38] using normal kidney tissue expression and pathology prognosis (Supplementary Table S4). Overall, 95% of the significantly different proteins had medium and high expression in normal kidney tissue. Moreover, 22 proteins had medium and high expression in glomerular cells. Sixteen proteins (two with highest expression in MCD) presented high normal kidney tissue expression (HSPA9, MME, PDIA3, EIF4A2, COL18A1, PCBP1, H1-0, MYH10, NES, ITGA3, YWHAZ, CRYM, ATP1A1, CDH13, WDR1, ACTR3), eight of them (MME, EIF4A2, COL18A1, H1-0, NES, ITGA3, YWHAZ, CDH13) having high expression in normal glomerular cells.

**Figure 3:**
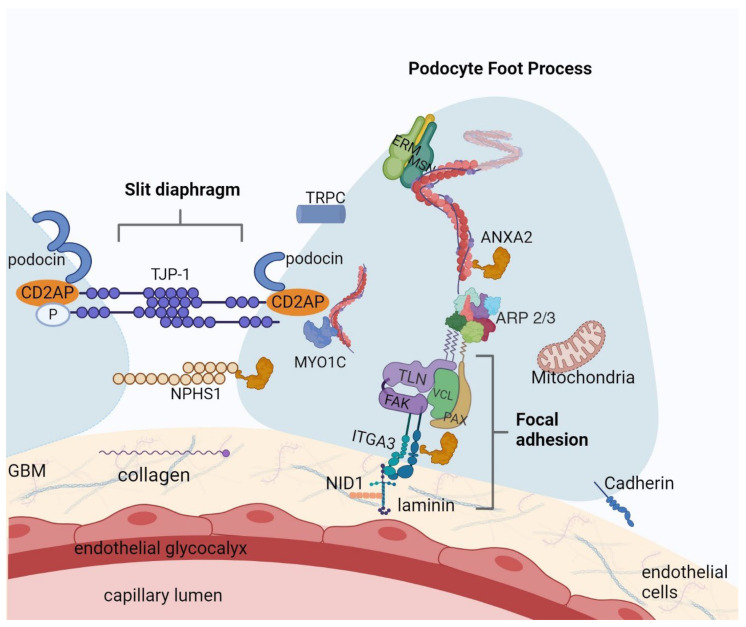
Proteins differentiated in MCD: an illustration of podocyte foot process with focal adhesion and slit diaphragm structure. Some of the proteins with statistically significant upregulation (ANXA2, NID1, MSN, MYO1C) or downregulation (ARP3, ITGA3, TJP-1, COL18A1 component of collagen, CDH13 member of cadherin) in MCD versus CTR are represented at the podocyte level. For understanding, we also incorporated proteins that are presented in the podocytes, but did not exhibit statistical significance in our study (NPHS1, podocin, CD2AP, TRPC, ERM, TLN, FAK, VCL, PAX, laminin network). This figure was created with BioRender.com.

In addition, proteins that showed statistically significant upregulation or downregulation in MCD (compared to CTR) are illustrated in Figure 3. Although glomeruli were dissected as a whole and some of the proteins were also expressed in more than one location in the glomeruli (e.g., mesangial cells/endothelium), most of them have a meaningful and documented role in the structure and function of the podocyte. The depicted localization of different proteins reflects data available in the literature, as presented in the Discussion section. Proteins presented in Figure 3 are listed in Supplementary Table S4, displaying varying levels of abundance between the MCD and CTR groups.

### 2.4. Immunohistochemistry

**Figure 4:**
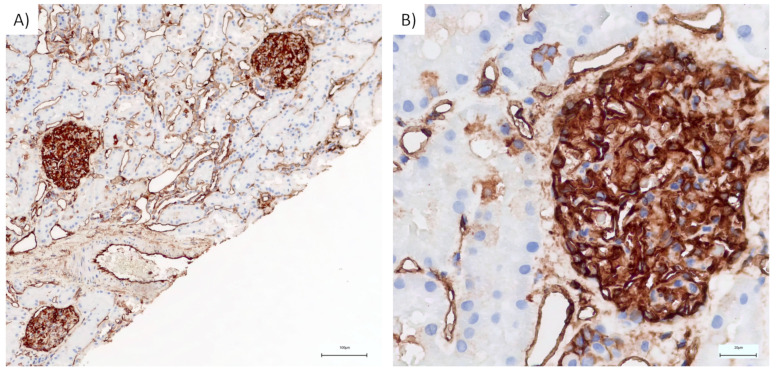
Immunohistochemical annexin staining of one of the kidney biopsies. (**A**) ANXA2 staining, 10× magnification, showing positive staining at the luminal border of the tubules and intense staining in the glomerulus. (**B**) ANXA2, 40× magnification showing intense staining in one of the positive glomeruli.

Immunohistochemistry staining was performed on three MCD cases. ANXA2 antibody staining produced a strong (3+) mesangial glomerular stain in two samples, while a moderate (2+) stain was observed in the third sample. The median H-score for ANXA2 staining at the glomerular level was 300. Figure 4 depicts immunohistochemical ANXA2 staining of one of the kidney biopsies.

## 3. Discussion

The precise mechanisms that regulate the dynamic and morphology of the podocyte cytoskeleton remain unknown. Moreover, it is becoming increasingly clear that they play a crucial role in the pathogenesis of podocyte injury in kidney disorders, particularly in MCD. Using STRING enrichment analysis and the Compartments database, we were able to identify proteins that were linked to the actin cytoskeleton (actin filament, myosin complex), FA, anchoring junction (which is SD-related), and basement membrane, as illustrated in Figure 2. Based on the identified clusters, we investigated proteins that may be associated with the pathogenesis of MCD. Out of the differentially expressed proteins in the glomeruli of MCD patients compared to controls, some were reported in several glomerular cell types (e.g., ANXA2 has been reported in endothelial, epithelial, and mesangial cells, NID1 is found in mesangial matrix and glomerular basement membrane). However, the majority of differentially expressed proteins could be traced back to regulation of the podocyte cytoskeleton using STRING network analysis, as shown in Figure 2. Some of those proteins are known to be associated to the main anchoring points and signaling hubs of the podocyte, namely, SD (ANXA2, TJP1 and MYO1C) and FA (NID1 and ITGA3), and interestingly, others are structural components of the actin and microtubule cytoskeleton of podocytes (ACTR3 and NES). Distribution of these proteins in the podocyte is depicted in Figure 3.

The SD is a highly specialized structure of the podocyte. The apical junctional complex structures vanish during podocyte differentiation and are replaced by a highly differentiated anchoring junction, the SD. Slit diaphragm-associated proteins are essential for permselectivity of the glomerular filtration, and SD-mediated signals influence cytoskeleton rearrangement, including FPE [39,40,41]. In our study, ANXA2, a critical component of the SD, was more abundant, whereas TJP1 was less represented in MCD when compared to controls.

ANXA2 is found in kidney endothelial, mesangial, and epithelial cells. In podocytes, ANXA2 and nephrin are colocalized in the SD [42]. However, ANXA2 signaling may also be triggered following integrin binding at the level of cell adhesions to the GBM: in endothelial and epithelial cells, ANXA2 links to proteins at E-cadherin-based adherent junctions and is then internalized into the cytoplasm [43,44]. Evidence suggests that ANXA2 interacts with the actin cytoskeleton and alters its dynamics: it directly binds to F actin through a specific binding site [45] and it reduces actin monomer polymerization by reducing growth at the barbed end of the actin filament, hence impairing the cell’s protrusive and retractile capacity [46]. ANXA2 also regulates the activation of Rho and Rac1 GTPases [46,47,48,49].

The most extensive evidence for ANXA2’s pathogenic role in kidney disease is derived from studies in lupus nephritis: ANXA2 mediates the binding of anti-dsDNA antibodies to mesangial cells in situ [50] and represents an antigen for circulating autoantibodies [51] and CD4+ lymphocytes [52] and a marker of severity of renal involvement [52,53,54,55]. ANXA2 may contribute to several other kidney diseases, such as diabetic nephropathy or acute kidney injury. It influences a variety of pathways, including integrin-mediated activation of integrin-linked kinase (ILK) at the level of FA with activation of the NF-kB pathway [56], macrophage recruitment and fibrosis [57], and complement activation (directly binds to factor H and impairs its activity) [58].

With regard to MCD, a recent study found IgG4 antibodies against ANXA2 in 17% of children with non-genetic steroid-resistant nephrotic syndrome in a large multicenter study in China [42]. Anti-ANXA2 antibodies caused proteinuria and podocyte injury in mice and altered migration and adhesion of cultured podocytes. This was triggered by tyrosine 24-mediated phosphorylation of ANXA2 upon antibody binding, followed by induction of CD42 and RAC1 kinase [42]. We discovered ANXA2 to be increased in MCD. It is worth noting that all of our patients were adults who responded to therapy. The majority were corticotherapy-sensitive, while the others required additional immunosuppression. ANXA2 staining was confirmed by immunohistochemistry with intense staining, as depicted in Figure 4.

Several apical junctional complex constituents, such as P-cadherin, catenin, and TJP1, are localized at the SD and form molecular complexes with the actin filaments and podocyte-specific proteins [39]. Our findings demonstrate a considerable decrease in TJP1, a critical component of the SD, compared to normal tissue. TJP1 is essential for glomerular structure [59,60,61]. Loss of TJP1 impairs interdigitating podocyte architecture with loss of the SD, rupture and effacement of the FP and weakened adhesion of the latter to the GBM, paralleled by persistent proteinuria. TJP1 inactivation may affect actin filament organization with flattening of the FP [39]. The reduction in and redistribution of TJP1 in glomerular podocytes has also been seen in animal models of diabetes [59,60,61].

MYO1C is found to colocalize with other SD proteins such as Neph1 in rat podocytes. MYO1C knockdown reduced nephrin at the SD by >70% and reduces migration capabilities of the podocyte. MYO1C’s capacity to interact with membranes, F-actin, Neph1, and nephrin [62] supports the concept that this motor protein actively contributes to the dynamic architecture of the filtration slit. MYO1C, like all class I myosins, is linked to the Arp2/3 complex-mediated polymerization and assembly of actin [63] and is crucial in the creation and maintenance of cortical tension and associated processes like motility, endocytosis, and exocytosis [64,65].

The second category of differentially expressed proteins are those associated with FA. Focal adhesion proteins are major mechanical stabilizers of podocytes, but also an important signaling point for cytoskeletal rearrangements. We found several FA-linked proteins, e.g., NID1 and ITGA3, which were up- and downregulated, respectively, in MCD when compared to controls, as they may have a significant impact on FA function.

Nidogens presumably cross-link collagen and laminin networks with one another and the cell surface, and although not essential for GBM formation [66,67], they contribute to the overall strength and stress resistance of the basement membrane [68]. Nidogens have been shown to be upregulated in the mesangial matrix and the GBM in glomerular disorders, such as lupus nephritis, IgA nephropathy, and diabetic nephropathy [69,70], but it is a novelty for NID1 to be increased in MCD, presumably as adaptation during FPE.

One importantly FA-related downregulated protein was ITGA3, a component of integrin α3β1, the most prevalent integrin in glomeruli and a key molecule for the adhesion of podocytes to the GBM. Indeed, proteinuria and FPE are part of the severe developmental phenotype induced by selective deletion of the ITGA3 subunit [71]. Disorganization of the GBM and incompletely developed and flattened FP are the morphological changes associated with this condition [72]. It seems that upon contact with the extracellular matrix (ECM), integrin α3β1 transmits signals that induce specific responses, such as adhesion, migration, filopodial extension, and in the case of podocytes, the assembly of the foot process [73]. In human disease, mutations of the ITGA3 subunit led to atrophic glomeruli, FSGS, widespread interstitial fibrosis, proteinuria, severe renal abnormalities, and early mortality [74,75,76]. Interestingly, downregulated ITGA3 expression was also reported in the early stages of human and experimental diabetic nephropathy [77].

The third category of proteins that are differentially expressed are structural proteins of the actin cytoskeleton of podocytes and include ACTR3 and NES, both of which are decreased in MCD.

ACTR3 is a component of the ARP2/3 complex, which is essential for cross-linking actin at a fixed angle and promotes actin filament branching and expansion via actin nucleation [78]. These actions are mediated by SD and FA [6,79] signals, and involve protein phosphorylation (e.g., nephrin phosphorylation), followed by recruitment of Rho small GTPase family members (such as Rac1, CDc42) and activation of WASP proteins. ARP2/3 complex suppression reduces peripheral actin turnover, ARP2/3 incorporation into the actin network, and migratory behavior. ARP2/3 complex knockdown caused unstable cellular protrusions and impaired FA in epithelial cells [80]. Proteinuria and GSFS are caused by a point mutation in ACTR3 at BUF/Mna in rats [81].

NES levels were significantly reduced in MCD glomeruli compared to healthy controls. NES is a cytoskeleton-linked class VI intermediate filament protein that is only expressed in differentiated and mature podocytes [82]. NES knockdown reduces the number of processes extending from cells in the murine podocyte cell line, supporting the idea that NES maintains cell shape and architecture, which is required for podocyte function [83]. NES is mostly found in podocyte primary processes according to immunoelectron microscopy. NES levels are significantly lower in proteinuric kidney injuries caused by FPE, such as MN, FSGS, IgA nephropathy, and proteinuric diabetic nephropathy [84]. They are also significantly lower in murine models of podocyte damage [85,86]. NES expression was negatively correlated with proteinuria, but positively correlated with nephrin expression in human lupus nephritis and MRL/lpr mice and may help to mitigate oxidative damage in the glomeruli [87].

Regarding podocytopathies, a novel concept is being defined. The histomorphological lesions of MCD, FSGS, and maybe other glomerular diseases with podocyte involvement are non-specific and represent various patterns of podocyte injury rather than defining a specific disease [18,88,89,90]. As a result, it is suggested that it is crucial to rename this family of disorders as “podocytopathies” [1,13,89]. Proteomic analysis can help pinpoint the trigger or triggers that cause podocyte injury. The goal of this strategy is to define an individual prognosis and course of treatment.

The most significant limitation of our study is the low number of patients included; however, at this stage of glomerular proteomic research, existing studies in the literature include only a small number of patients [91]. Due to the limited number of patients in each group and the inherent biological variability and heterogeneity of human samples, the proteins identified in this investigation exhibit a low level of precision, as indicated by a coefficient of variation (CV) exceeding 20%. The Supplementary Tables S2 and S4 contain the relevant data for further examination. Even though participants were few, the proteomic profiles of the patients were overall very similar, allowing differentiation from controls. However, some proteins differed between MCD participants. These differences could be explained by distinct/complementary pathogenetic mechanisms. Differentially expressed proteins in MCD versus controls might be the consequence of the same pathogenic chain or might reflect slightly different mechanisms in different patients. We plan to confirm these findings with larger and more uniform samples of MCD patients, as well as correlate them with urinary and plasma proteomics, with the goal of progressing towards a theragnostic tool and personalized treatment.

Another significant constraint was that our healthy controls consisted of tissue obtained from patients undergoing nephrectomy (who had normal renal function and were not diagnosed with chronic kidney disease). Furthermore, a pathologist conducted a light-microscopy examination of the tissue and observed no alterations. We acknowledge that this is not the ideal control, and an influence on proteomic analysis of the neoplasm cannot be excluded. However, ethical considerations arise in glomerular proteomic studies concerning the obtainment of glomerular tissue from controls lacking any indication for kidney biopsy. Tissue obtained from autopsy studies is heavily influenced by hypoxia and other disease-associated conditions. The use of tissue obtained from nephrectomies is consistent with previous research [92]. An alternative would be tissue from transplant donors, which unfortunately was not an available option for us.

## 4. Materials and Methods

### 4.1. Sample Collection and Storage

An observational, retrospective, and cross-sectional study was conducted using MCD formalin-fixed, paraffin-embedded (FFPE) biopsies obtained from the Emergency Hospital of Cluj-Napoca, along with control (CTR) kidney tissue collected from the Institute of Urology and Transplantation of Cluj-Napoca. The study included patients with sufficient tissue for proteomic analysis and biopsies from 2019 to 2022. An experienced pathologist confirmed the MCD diagnosis of all study participants using light and electronic microscopy and immunofluorescence. Control kidney tissue was obtained from patients who underwent nephrectomies for tumors (with normal renal function and without a chronic kidney disease diagnostic), with kidney tissue harvested at a site distant from the tumor. A pathologist confirmed the absence of pathological alterations in these samples. We initially proved the feasibility of such a proteomic approach in glomerulopathies in our center [93].

Patient data were extracted from the Emergency Hospital of Cluj-Napoca records of the patients following consent. Paraclinical data are presented as means ± standard deviation (SD) in Supplementary Table S1. Clinical and laboratory data were collected for each patient included in the study: age, gender, renal function, serum albumin, proteinuria from 24 h urine collection, and therapeutic response.

### 4.2. Laser Capture Microdissection Sample Preparation

The 10 µm sections were affixed to MMI RNAse/DNAse-free membrane slides (Molecular Machines & Industries, Eching, Germany) and subjected to staining via Mayer’s hematoxylin staining protocol. The laser capture microdissection system MMI CellCut Plus (MMI AG, Glattbrugg, Switzerland) was used to perform the isolation of glomerular sections. In the LCM protocol, the tissue image is visualized on an LCD monitor, and subsequently a skilled pathologist identifies and demarcates each glomerulus. After identifying the glomeruli, the tissue sections were isolated in an environment free from contamination. This is achieved by placing the tissue sections between a clean glass slide and the MMI membrane. Sections of tissue were collected using adherent MMI collection caps, which were designed to prevent contamination during the collection process. Glomeruli were collected for each case and subsequently subjected to proteomic analysis.

### 4.3. Sample Preparation for Proteome Profiling

LCM microdissections were resuspended in 20 µL Rapigest^®^ 0.1% (prepared in 100 mM ammonium bicarbonate) (Waters Corporation, Milford, MA, USA) and transferred into protein low-retention tubes. Protein extraction was facilitated by sonication (3 × 3 s, A: 80%) and two heat incubation steps (95 °C, 15 min and 80 °C, 60 min). Sample preparation was further conducted by means of a single-pot solid-phase-enhanced sample preparation (SP3) protocol using two types of carboxylate-modified paramagnetic beads (Sera-Mag SpeedBeads purchased from GE Healthcare, Chicago, IL, USA), as described by Hughes et al. [94]. A quantity of 15 µL of supernatant was reduced and alkylated (final concentration of DTT 5 mM and IAA 10 mM, respectively). Briefly, 10 µL SP3 bead stock (20 µg/µL) was added to each sample, and binding of proteins to magnetic beads was induced by 100% acetonitrile (ACN) addition. Beads were incubated on a magnetic rack for 2 min and the supernatant was discarded. The beads were further washed with 100% EtOH and 100% ACN, then redissolved in Rapigest^®^ 0.1% buffer supplemented with trypsin at an enzyme-to-protein ratio of 1:25 (*w*/*w*). ACN 100% was used to initiate the peptide binding to the magnetic beads. Incubation and washing steps were repeated as already indicated. Beads were pelleted, the supernatant was removed, and beads were dried for 5 min at RT. Peptides were eluted using DMSO 2% (*v*/*v* in water). Supernatant containing purified peptides was transferred into a new vial and acidified using 1% FA. Prior to LC–MS analysis, a digestion of yeast alcohol dehydrogenase (ADH1) (Waters Corporation, Milford, MA, USA) in 5% ACN and 0.1% formic acid (FA) was added as an internal standard protein (final concentration of ADH was 25 fmol/μL).

### 4.4. LC−MS Analysis

LC−MS analyses were performed using a NanoAQUITY UPLC system (Waters Corporation, Milford, MA, USA) coupled with a Synapt G2-S high-definition mass spectrometer (Waters Corporation, Wilmslow, UK) via a NanoLockSpray dual electrospray ion source. Peptides were trapped on an ACQUITY UPLC M-Class Trap Symmetry C18 column, 5 μm particles, 180 μm × 20 mm (Waters Corporation, Wexford, Ireland), for 2 min at 5 μL/min in 0.5% solvent B (0.1% (*v*/*v*) formic acid in acetonitrile). An ACQUITY UPLC M-Class reverse phase C18 column HSS T3, 1.8 μm, 75 μm × 250 mm (Waters Corporation, Wexford, Ireland) was used for peptide separation [41]. Peptides were separated running a gradient from 5% to 85% (*v*/*v*) mobile phase B at a flow rate of 300 nL/min over a 45 min gradient. The column was heated to 55 °C. MS analysis of eluting peptides was performed by ion-mobility separation (IMS)-enhanced data-independent acquisition (DIA) in UDMSE mode [95]. Lock mass compound Glu-1-fibrinopeptide B (GluFib) (100 fmol/µL) was delivered by auxiliary pump at a flow rate of 0.3 μL/min, the spectra of the doubly charged species (*m*/*z* 785.8426) being recorded every 45 s.

### 4.5. Data Processing and Label-Free Quantification

Raw MS data were processed by Progenesis QIP version 4.2 (nonlinear dynamics, Waters Corporation) where data were post-acquisition lock-mass-corrected using the doubly charged monoisotopic ion of GluFib and aligned with the most suitable reference run identified automatically by the software. The “normalization to all proteins” option was used, by which protein amounts in individual runs are normalized to one run automatically selected as the normalization reference. Data were searched against the UniPortKB/Swiss-Prot Human target-decoy database containing 20,361 proteins (downloaded January 2022) to which yeast alcohol dehydrogenase (P000330) was added, using the following parameters: trypsin as digestion agent, maximum missed cleavages: one; fixed modification: carbamidomethylation and methionine oxidation; hydroxylation of asparagine, methylation at aspartic acid, proline, lysine; lysine methylation and lysine formylation at N-term as variable modifications; false-discovery rate (FDR) was set to <1%. Ion-matching requirements implemented were: (i) minimum one fragment ion match per peptide ion; (ii) three fragment ions matched per protein identification and (iii) at least one peptide match per protein identification. Peptides with mass error of more than 20 ppm and length <5 amino acids were removed. Protein quantitation was performed using ADH1 (at concentration 25 fmol/uL). The reviewed list of proteins was exported for subsequent data analysis. Technical replicates were averaged, and the final protein list included a total of 311 proteins. Metaboanalyst 5.0 (version 2023-06-23) was used for subsequent post-processing protein analysis. Here, proteins with more than 30% missing values were removed. The remaining missing values were estimated using k-nearest neighbor based on similar features—KNN (feature-wise) option. No further filtering was applied. Data were log10-transformed and scaled using the Pareto scaling option. For differential expression analysis, two-sample *t*-tests with unequal group variance were applied with a *p*-value threshold of 0.05 and fold change |FC| ≥ 1.5. Data visualizations (PCA plots, heatmaps) were obtained using the same online tool.

The Human Protein Atlas v.23.0 (www.proteinatlas.org) version 22 (accessed on 12 April 2023) [38,96] was used for obtaining data regarding normal kidney tissue distribution and prognostic attributes of the significant proteins. Standard gene names were used for protein annotation.

The protein–protein interaction (PPI) network was retrieved using the online STRING database [37]. The following query parameters were used: i. retrieve a full STRING network of functional associations and physical subnetwork; ii. minimum required interactions score cutoff confidence ≥ 0.70, iii. maximum number of interactors for the first shell (query proteins): 5 with confidence score cutoff 0.7. StringApp (version 12.0) was used to retrieve functional enrichment for Gene Ontology terms, KEGG Pathways, and Compartments applying a medium FDR stringency of 5%. Terms enriched were selected by means of strength parameter (strength ≥ 1).

### 4.6. Immunohistochemistry

Paraffin blocks were cut at 2 μm and mounted on silane-coated slides. Immunohistochemistry (IHC) staining was performed using a fully automated slide preparation system (Leica BOND, Leica Byosistems, Deer Park, IL, USA). IHC staining using the standard manufacturer’s instructions was used for annexin 2/ANXA2 antibody (C-10, SC-28385). Membranous and cytoplasmatic staining was considered positive staining. The staining intensity was graded as negative, weak, moderate, or strong (score 0, 1, 2, or 3, respectively). Each intensity score was visually estimated. The resulting combined score (H—score) was calculated as the sum of the percentage of stained cells multiplied by the intensity scores (minimum 0, maximum 300).

## 5. Conclusions

In conclusion, this study suggests the potential of glomerular proteomic analysis to yield valuable insights into the pathogenesis of podocytopathies like MCD. Most of the significantly different proteins (41.66%) were involved in podocyte cytoskeleton regulation, and these protein alterations could be attributed to cytoskeleton dysfunction, which leads to FPE and proteinuria. Otherwise, these variations could be considered a compensatory response to changes in podocyte anatomy and function caused by various triggers. Either of these hypotheses implies that the previously discussed proteins may play an important role in the pathogenesis of MCD.

We plan to conduct prospective studies comparing MCD patients to controls to validate the current results and take a step forward towards theragnostics by linking tissue to blood and urine proteomics. The urine proteome in particular is an interesting candidate to reflect changes in tissular proteins in the setting of podocytopathies, as it is directly influenced by changes in permselectivity and may be a valuable non-invasive tool to stratify patients for prognosis and response to therapy.

## Data Availability

The original data presented in the study are openly available in MassIVE repository at doi:10.25345/C5JH3DD42.

## Supplementary Materials

The supporting information can be downloaded at https://www.mdpi.com/article/10.3390/ijms25115613/s1.
