# Supplementary material for: Minimal Change Disease: Pathogenetic Insights from Glomerular Proteomics
Source: Int J Mol Sci. 2024 May 21;25(11):5613. doi: 10.3390/ijms25115613 (PMC11171934; doi:10.3390/ijms25115613)
Supplement: Supplementary file 1 [file ijms-25-05613-s001.zip › Supplementary information File S1 Progenesis QIp report of identified proteins.pdf]

2301_LCM_CTRvsMCD

Experiment: 2301_LCM_CTRvsMCD

Report created: 4/23/2024 12:35:36 PM

Proteins

Protein building options

Protein grouping **Group similar proteins**
Protein quantitation **Absolute Quantitation using Hi-3**

Accession	Peptides	Score	Anova (p)*	Fold	Tags	Description	Average Normalised Abundances	
							CTR	MCD
P13645	72 (72)	543.78	0.53	1.85		Keratin_ type I cytoskeletal 10 OS=Homo sapiens OX=9606 GN=KRT10 PE=1 SV=6	15.19	8.19
P02768	53 (53)	479.23	0.03	4.87		Albumin OS=Homo sapiens OX=9606 GN=ALB PE=1 SV=2	2.95	0.61
P04264	74 (74)	477.72	0.19	1.14		Keratin_ type II cytoskeletal 1 OS=Homo sapiens OX=9606 GN=KRT1 PE=1 SV=6	20.21	17.66
P08670	56 (56)	477.06	0.50	1.52		Vimentin OS=Homo sapiens OX=9606 GN=VIM PE=1 SV=4	3.55	2.33
P00330	91 (91)	394.51	6.30e-004	3.09		Alcohol dehydrogenase 1 OS=Saccharomyces cerevisiae (strain ATCC 204508 / S288c) OX=559292 GN=ADH1 PE=1 SV=5	21.67	67.00
P35908	45 (45)	372.42	0.07	1.32		Keratin_ type II cytoskeletal 2 epidermal OS=Homo sapiens OX=9606 GN=KRT2 PE=1 SV=2	7.74	10.19
P63261	46 (46)	360.87	0.64	1.12		Actin_ cytoplasmic 2 OS=Homo sapiens OX=9606 GN=ACTG1 PE=1 SV=1	4.44	4.95
P35579	51 (51)	354.00	0.16	1.15		Myosin-9 OS=Homo sapiens OX=9606 GN=MYH9 PE=1 SV=4	0.29	0.33
O43707	36 (36)	295.37	0.44	1.93		Alpha-actinin-4 OS=Homo sapiens OX=9606 GN=ACTN4 PE=1 SV=2	2.10	1.09
P35527	35 (35)	254.07	3.25e-003	2.42		Keratin_ type I cytoskeletal 9 OS=Homo sapiens OX=9606 GN=KRT9 PE=1 SV=3	3.04	7.36
P02545	29 (29)	226.95	3.19e-004	2.55		Prelamin-A/C OS=Homo sapiens OX=9606 GN=LMNA PE=1 SV=1	1.54	3.93
P11047	29 (29)	192.47	0.02	1.61		Laminin subunit gamma-1 OS=Homo sapiens OX=9606 GN=LAMC1 PE=1 SV=3	1.11	1.79
O00468	23 (23)	173.21	5.71e-004	2.78		Agrin OS=Homo sapiens OX=9606 GN=AGRN PE=1 SV=6	3.42	9.49
P68871	23 (23)	167.29	0.03	1.51		Hemoglobin subunit beta OS=Homo sapiens OX=9606 GN=HBB PE=1 SV=2	3.19	4.81
P55268	25 (25)	158.98	0.05	1.36		Laminin subunit beta-2 OS=Homo sapiens OX=9606 GN=LAMB2 PE=1 SV=2	0.40	0.55
P48681	24 (24)	149.14	5.70e-004	2.67		Nestin OS=Homo sapiens OX=9606 GN=NES PE=1 SV=2	0.27	0.10
P13647	22 (22)	148.49	0.90	1.35		Keratin_ type II cytoskeletal 5 OS=Homo sapiens OX=9606 GN=KRT5 PE=1 SV=3	1.41	1.04
P07355	17 (17)	143.55	2.07e-005	2.76		Annexin A2 OS=Homo sapiens OX=9606 GN=ANXA2 PE=1 SV=2	0.30	0.84
P11142	20 (20)	139.40	4.70e-003	1.39		Heat shock cognate 71 kDa protein OS=Homo sapiens OX=9606 GN=HSPA8 PE=1 SV=1	0.35	0.26
P02533	16 (16)	130.29	0.68	1.14		Keratin_ type I cytoskeletal 14 OS=Homo sapiens OX=9606 GN=KRT14 PE=1 SV=4	1.32	1.16
P18206	19 (19)	125.27	1.37e-005	2.83		Vinculin OS=Homo sapiens OX=9606 GN=VCL PE=1 SV=4	0.52	1.48
P12883	18 (18)	117.16	5.76e-004	3.02			3.21	9.69

Accession	Peptides	Score	Anova (p)*	Fold	Tags	Description	Average Normalised Abundances	
							CTR	MCD
						Myosin-7 OS=Homo sapiens OX=9606 GN=MYH7 PE=1 SV=5		
P14543	21 (21)	114.53	2.68e-004	8.25		Nidogen-1 OS=Homo sapiens OX=9606 GN=NID1 PE=1 SV=3	0.54	4.49
P08572	18 (18)	112.46	8.94e-005	2.74		Collagen alpha-2(IV) chain OS=Homo sapiens OX=9606 GN=COL4A2 PE=1 SV=4	0.67	1.83
P02538	12 (12)	105.02	3.53e-003	2.18		Keratin_ type II cytoskeletal 6A OS=Homo sapiens OX=9606 GN=KRT6A PE=1 SV=3	1.32	2.87
P00738	16 (16)	103.39	0.04	3.47		Haptoglobin OS=Homo sapiens OX=9606 GN=HP PE=1 SV=1	0.18	0.05
Q96JY6	13 (13)	103.16	0.76	1.07		PDZ and LIM domain protein 2 OS=Homo sapiens OX=9606 GN=PDLIM2 PE=1 SV=1	0.32	0.30
P33778	13 (13)	102.12	2.52e-004	2.67		Histone H2B type 1-B OS=Homo sapiens OX=9606 GN=H2BC3 PE=1 SV=2	8.96	23.93
P68363	12 (12)	101.83	1.11e-004	1.64		Tubulin alpha-1B chain OS=Homo sapiens OX=9606 GN=TUBA1B PE=1 SV=1	0.49	0.81
P26038	16 (16)	99.21	1.49e-004	1.98		Moesin OS=Homo sapiens OX=9606 GN=MSN PE=1 SV=3	0.76	1.50
P01024	17 (17)	98.56	0.44	1.17		Complement C3 OS=Homo sapiens OX=9606 GN=C3 PE=1 SV=2	0.46	0.54
P53420	18 (18)	92.91	1.93e-003	1.89		Collagen alpha-4(IV) chain OS=Homo sapiens OX=9606 GN=COL4A4 PE=1 SV=3	0.77	1.46
P69905	17 (17)	91.74	4.72e-004	1.75		Hemoglobin subunit alpha OS=Homo sapiens OX=9606 GN=HBA1 PE=1 SV=2	4.27	7.46
P11021	15 (15)	90.27	0.93	1.09		Endoplasmic reticulum chaperone BiP OS=Homo sapiens OX=9606 GN=HSPA5 PE=1 SV=2	0.88	0.80
P02787	12 (12)	79.58	4.41e-005	2.13		Serotransferrin OS=Homo sapiens OX=9606 GN=TF PE=1 SV=3	0.14	0.30
P02462	13 (13)	78.69	0.03	1.53		Collagen alpha-1(IV) chain OS=Homo sapiens OX=9606 GN=COL4A1 PE=1 SV=4	0.50	0.33
P15311	13 (13)	74.44	0.90	2.08		Ezrin OS=Homo sapiens OX=9606 GN=EZR PE=1 SV=4	3.04	1.46
P62805	9 (9)	73.27	0.70	1.05		Histone H4 OS=Homo sapiens OX=9606 GN=H4C1 PE=1 SV=2	1.54	1.47
P01860	11 (11)	70.03	0.01	2.30		Immunoglobulin heavy constant gamma 3 OS=Homo sapiens OX=9606 GN=IGHG3 PE=1 SV=2	0.60	0.26
P68431	6 (6)	69.54	0.18	1.33		Histone H3.1 OS=Homo sapiens OX=9606 GN=H3C1 PE=1 SV=2	0.53	0.70
Q15599	9 (9)	68.86	0.30	1.44		Na(+)/H(+) exchange regulatory cofactor NHE-RF2 OS=Homo sapiens OX=9606 GN=SLC9A3R2 PE=1 SV=2	0.08	0.06
P0CG47	8 (8)	67.42	8.55e-005	1.90		Polyubiquitin-B OS=Homo sapiens OX=9606 GN=UBB PE=1 SV=1	1.22	2.32
P07911	10 (10)	67.19	0.01	7.31		Uromodulin OS=Homo sapiens OX=9606 GN=UMOD PE=1 SV=1	0.41	0.06
P07437	10 (10)	66.31	1.39e-003	1.65		Tubulin beta chain OS=Homo sapiens OX=9606 GN=TUBB PE=1 SV=2	0.45	0.27
Q02413	10 (10)	64.26	0.09	6.84		Desmoglein-1 OS=Homo sapiens OX=9606 GN=DSG1 PE=1 SV=2	0.27	0.04
P67936	9 (9)	62.57	0.24	1.62		Tropomyosin alpha-4 chain OS=Homo sapiens OX=9606 GN=TPM4 PE=1 SV=3	0.75	0.46
P06396	10 (10)	61.80	1.85e-003	4.18		Gelsolin OS=Homo sapiens OX=9606 GN=GSN PE=1 SV=1	0.37	0.09
P08779	10 (10)	60.94	0.15	1.51		Keratin_ type I cytoskeletal 16 OS=Homo sapiens OX=9606 GN=KRT16 PE=1 SV=4	0.21	0.32
Q01955	12 (12)	59.01	0.28	1.22		Collagen alpha-3(IV) chain OS=Homo sapiens OX=9606 GN=COL4A3 PE=1 SV=3	0.88	0.72
P32119	8 (8)	57.47	5.53e-003	1.95		Peroxiredoxin-2 OS=Homo sapiens OX=9606 GN=PRDX2 PE=1 SV=5	0.57	0.29
P0DMV8	7 (7)	56.30	1.07e-006	1.80		Heat shock 70 kDa protein 1A OS=Homo sapiens OX=9606 GN=HSPA1A PE=1 SV=1	0.27	0.15

Accession	Peptides	Score	Anova (p)*	Fold	Tags	Description	Average Normalised Abundances	
							CTR	MCD
P01876	6 (6)	55.01	0.05	3.77		Immunoglobulin heavy constant alpha 1 OS=Homo sapiens OX=9606 GN=IGHA1 PE=1 SV=2	0.39	0.10
P02679	8 (8)	54.79	0.03	1.53		Fibrinogen gamma chain OS=Homo sapiens OX=9606 GN=FGG PE=1 SV=3	0.18	0.12
P04406	7 (7)	54.62	0.14	1.32		Glyceraldehyde-3-phosphate dehydrogenase OS=Homo sapiens OX=9606 GN=GAPDH PE=1 SV=3	0.70	0.53
P00761	14 (14)	53.64	2.95e-003	2.16		Trypsin OS=Sus scrofa OX=9823 PE=1 SV=1	13.26	28.70
P07195	8 (8)	53.25	0.40	1.23		L-lactate dehydrogenase B chain OS=Homo sapiens OX=9606 GN=LDHB PE=1 SV=2	0.29	0.24
P08238	8 (8)	52.77	7.27e-003	1.63		Heat shock protein HSP 90-beta OS=Homo sapiens OX=9606 GN=HSP90AB1 PE=1 SV=4	0.22	0.14
P00558	10 (10)	52.47	2.40e-005	5.13		Phosphoglycerate kinase 1 OS=Homo sapiens OX=9606 GN=PGK1 PE=1 SV=3	4.66	23.89
P02675	8 (8)	52.36	0.86	1.03		Fibrinogen beta chain OS=Homo sapiens OX=9606 GN=FGB PE=1 SV=2	0.18	0.17
P50895	8 (8)	52.33	5.27e-003	3.62		Basal cell adhesion molecule OS=Homo sapiens OX=9606 GN=BCAM PE=1 SV=2	0.10	0.03
P04083	7 (7)	52.15	0.74	1.04		Annexin A1 OS=Homo sapiens OX=9606 GN=ANXA1 PE=1 SV=2	0.12	0.12
P12036	10 (10)	50.14	0.19	1.00		Neurofilament heavy polypeptide OS=Homo sapiens OX=9606 GN=NEFH PE=1 SV=4	3.50	3.50
P61981	7 (7)	49.90	1.07e-004	2.80		14-3-3 protein gamma OS=Homo sapiens OX=9606 GN=YWHAG PE=1 SV=2	0.45	0.16
Q5TZA2	10 (10)	49.77	0.13	1.81		Rootletin OS=Homo sapiens OX=9606 GN=CROCC PE=1 SV=1	0.71	0.39
Q06830	6 (6)	49.59	1.67e-003	2.24		Peroxiredoxin-1 OS=Homo sapiens OX=9606 GN=PRDX1 PE=1 SV=1	0.24	0.11
Q05682	8 (8)	49.06	0.36	1.20		Caldesmon OS=Homo sapiens OX=9606 GN=CALD1 PE=1 SV=3	0.11	0.09
Q03252	9 (9)	47.61	9.47e-003	1.42		Lamin-B2 OS=Homo sapiens OX=9606 GN=LMNB2 PE=1 SV=4	0.18	0.13
P02671	8 (8)	47.30	0.92	1.03		Fibrinogen alpha chain OS=Homo sapiens OX=9606 GN=FGA PE=1 SV=2	0.18	0.18
P04004	8 (8)	47.00	0.01	2.01		Vitronectin OS=Homo sapiens OX=9606 GN=VTN PE=1 SV=1	0.79	1.60
P08758	7 (7)	46.24	5.85e-004	1.77		Annexin A5 OS=Homo sapiens OX=9606 GN=ANXA5 PE=1 SV=2	0.33	0.58
P14618	8 (8)	46.11	8.60e-005	2.23		Pyruvate kinase PKM OS=Homo sapiens OX=9606 GN=PKM PE=1 SV=4	0.49	1.08
P09467	9 (9)	45.83	1.80e-004	3.07		Fructose-1_6-bisphosphatase 1 OS=Homo sapiens OX=9606 GN=FBP1 PE=1 SV=5	1.29	3.95
Q07157	9 (9)	45.36	5.63e-004	3.21		Tight junction protein ZO-1 OS=Homo sapiens OX=9606 GN=TJP1 PE=1 SV=3	0.15	0.05
O14745	7 (7)	45.19	0.28	1.39		Na(+)/H(+) exchange regulatory cofactor NHE-RF1 OS=Homo sapiens OX=9606 GN=SLC9A3R1 PE=1 SV=4	0.46	0.33
P25705	7 (7)	44.43	0.38	1.45		ATP synthase subunit alpha_ mitochondrial OS=Homo sapiens OX=9606 GN=ATP5F1A PE=1 SV=1	0.18	0.13
P04792	6 (6)	42.82	0.03	1.82		Heat shock protein beta-1 OS=Homo sapiens OX=9606 GN=HSPB1 PE=1 SV=2	0.37	0.20
Q5T749	7 (7)	42.72	0.09	1.56		Keratinocyte proline-rich protein OS=Homo sapiens OX=9606 GN=KPRP PE=1 SV=1	0.56	0.87
Q8N3V7	8 (8)	41.05	0.06	1.94		Synaptopodin OS=Homo sapiens OX=9606 GN=SYNPO PE=1 SV=2	0.10	0.05
P19012	7 (7)	40.32	0.04	2.88		Keratin_ type I cytoskeletal 15 OS=Homo sapiens OX=9606 GN=KRT15 PE=1 SV=3	0.16	0.06

Accession	Peptides	Score	Anova (p)*	Fold	Tags	Description	Average Normalised Abundances	
							CTR	MCD
P35749	8 (8)	39.99	1.31e-003	2.61		Myosin-11 OS=Homo sapiens OX=9606 GN=MYH11 PE=1 SV=3	0.32	0.12
P02042	5 (5)	39.04	4.88e-007	2.75		Hemoglobin subunit delta OS=Homo sapiens OX=9606 GN=HBD PE=1 SV=2	2.01	5.54
P08473	7 (7)	38.63	4.65e-004	1.54		Neprilysin OS=Homo sapiens OX=9606 GN=MME PE=1 SV=2	0.22	0.34
Q9NZN3	7 (7)	38.59	6.41e-006	3.72		EH domain-containing protein 3 OS=Homo sapiens OX=9606 GN=EHD3 PE=1 SV=2	0.65	2.41
P60660	6 (6)	38.41	1.11e-005	1.88		Myosin light polypeptide 6 OS=Homo sapiens OX=9606 GN=MYL6 PE=1 SV=2	0.37	0.69
Q93088	6 (6)	38.30	1.36e-003	2.25		Betaine--homocysteine S-methyltransferase 1 OS=Homo sapiens OX=9606 GN=BHMT PE=1 SV=2	1.11	2.50
Q03154	7 (7)	37.97	0.08	3.88		Aminoacylase-1 OS=Homo sapiens OX=9606 GN=ACY1 PE=1 SV=1	0.34	0.09
P62937	5 (5)	36.84	1.21e-003	4.17		Peptidyl-prolyl cis-trans isomerase A OS=Homo sapiens OX=9606 GN=PPIA PE=1 SV=2	0.23	0.05
P05556	6 (6)	36.80	0.15	1.49		Integrin beta-1 OS=Homo sapiens OX=9606 GN=ITGB1 PE=1 SV=2	0.31	0.20
P30086	6 (6)	36.41	0.08	1.36		Phosphatidylethanolamine-binding protein 1 OS=Homo sapiens OX=9606 GN=PEBP1 PE=1 SV=3	0.40	0.55
P10909	7 (7)	36.35	0.01	1.24		Clusterin OS=Homo sapiens OX=9606 GN=CLU PE=1 SV=1	0.15	0.12
P59665	4 (4)	35.90	5.02e-006	3.98		Neutrophil defensin 1 OS=Homo sapiens OX=9606 GN=DEFA1 PE=1 SV=1	0.39	0.10
Q6S8J3	9 (9)	35.86	0.32	1.03		POTE ankyrin domain family member E OS=Homo sapiens OX=9606 GN=POTEE PE=2 SV=3	1.40	1.44
Q14847	6 (6)	35.54	0.26	1.40		LIM and SH3 domain protein 1 OS=Homo sapiens OX=9606 GN=LASP1 PE=1 SV=2	0.18	0.13
B9A064	5 (5)	35.39	0.02	3.77		Immunoglobulin lambda-like polypeptide 5 OS=Homo sapiens OX=9606 GN=IGLL5 PE=2 SV=2	0.30	0.08
P02649	6 (6)	35.35	2.99e-003	2.04		Apolipoprotein E OS=Homo sapiens OX=9606 GN=APOE PE=1 SV=1	0.21	0.10
P12109	6 (6)	35.16	0.27	1.42		Collagen alpha-1(VI) chain OS=Homo sapiens OX=9606 GN=COL6A1 PE=1 SV=3	0.09	0.07
P60174	5 (5)	34.53	6.25e-003	3.16		Triosephosphate isomerase OS=Homo sapiens OX=9606 GN=TPI1 PE=1 SV=4	0.17	0.05
P0C0L4	6 (6)	34.43	0.01	2.36		Complement C4-A OS=Homo sapiens OX=9606 GN=C4A PE=1 SV=2	0.08	0.03
P37802	6 (6)	34.36	0.13	1.32		Transgelin-2 OS=Homo sapiens OX=9606 GN=TAGLN2 PE=1 SV=3	0.28	0.21
P19440	5 (5)	33.95	0.31	1.21		Glutathione hydrolase 1 proenzyme OS=Homo sapiens OX=9606 GN=GGT1 PE=1 SV=2	0.36	0.44
P63104	4 (4)	33.48	8.99e-005	3.39		14-3-3 protein zeta/delta OS=Homo sapiens OX=9606 GN=YWHAZ PE=1 SV=1	0.14	0.04
Q16555	6 (6)	33.05	6.92e-003	2.95		Dihydropyrimidinase-related protein 2 OS=Homo sapiens OX=9606 GN=DPYSL2 PE=1 SV=1	0.07	0.02
P06576	6 (6)	32.91	0.13	1.27		ATP synthase subunit beta_ mitochondrial OS=Homo sapiens OX=9606 GN=ATP5F1B PE=1 SV=3	0.23	0.29
P81605	3 (3)	32.30	0.52	2.07		Dermcidin OS=Homo sapiens OX=9606 GN=DCD PE=1 SV=2	1.19	0.58
O14950	5 (5)	32.28	8.41e-003	1.94		Myosin regulatory light chain 12B OS=Homo sapiens OX=9606 GN=MYL12B PE=1 SV=2	0.12	0.06
P14625	5 (5)	31.66	0.39	1.11		Endoplasmin OS=Homo sapiens OX=9606 GN=HSP90B1 PE=1 SV=1	0.11	0.10
O75083	6 (6)	31.38	1.07e-003	4.45			0.05	0.01

Accession	Peptides	Score	Anova (p)*	Fold	Tags	Description	Average Normalised Abundances	
							CTR	MCD
						WD repeat-containing protein 1 OS=Homo sapiens OX=9606 GN=WDR1 PE=1 SV=4		
P68133	4 (4)	31.12	0.66	2.47		Actin_ alpha skeletal muscle OS=Homo sapiens OX=9606 GN=ACTA1 PE=1 SV=1	2.02	0.82
P01871	5 (5)	30.72	2.13e-003	8.08		Immunoglobulin heavy constant mu OS=Homo sapiens OX=9606 GN=IGHM PE=1 SV=4	0.07	8.51e-003
P10412	4 (4)	30.69	9.52e-004	2.74		Histone H1.4 OS=Homo sapiens OX=9606 GN=H1-4 PE=1 SV=2	7.08	19.42
P08729	6 (6)	30.35	0.92	1.06		Keratin_ type II cytoskeletal 7 OS=Homo sapiens OX=9606 GN=KRT7 PE=1 SV=5	0.46	0.44
Q6UXI9	5 (5)	30.17	7.49e-003	2.06		Nephronectin OS=Homo sapiens OX=9606 GN=NPNT PE=2 SV=3	0.10	0.05
P09210	5 (5)	30.15	1.13e-005	1.84		Glutathione S-transferase A2 OS=Homo sapiens OX=9606 GN=GSTA2 PE=1 SV=4	0.37	0.20
P39060	5 (5)	29.76	2.45e-003	1.90		Collagen alpha-1(XVIII) chain OS=Homo sapiens OX=9606 GN=COL18A1 PE=1 SV=5	0.14	0.07
P06733	5 (5)	29.55	0.05	2.40		Alpha-enolase OS=Homo sapiens OX=9606 GN=ENO1 PE=1 SV=2	0.30	0.13
Q86Y46	8 (8)	29.33	0.02	1.72		Keratin_ type II cytoskeletal 73 OS=Homo sapiens OX=9606 GN=KRT73 PE=1 SV=1	1.62	2.79
P01009	5 (5)	29.17	0.02	2.77		Alpha-1-antitrypsin OS=Homo sapiens OX=9606 GN=SERPINA1 PE=1 SV=3	0.07	0.03
P00915	5 (5)	29.08	1.85e-005	2.85		Carbonic anhydrase 1 OS=Homo sapiens OX=9606 GN=CA1 PE=1 SV=2	0.45	1.29
P22626	4 (4)	28.61	0.28	1.21		Heterogeneous nuclear ribonucleoproteins A2/B1 OS=Homo sapiens OX=9606 GN=HNRNPA2B1 PE=1 SV=2	0.15	0.12
P01857	4 (4)	28.60	9.26e-003	2.99		Immunoglobulin heavy constant gamma 1 OS=Homo sapiens OX=9606 GN=IGHG1 PE=1 SV=1	0.15	0.05
P01834	3 (3)	28.42	1.92e-003	8.91		Immunoglobulin kappa constant OS=Homo sapiens OX=9606 GN=IGKC PE=1 SV=2	0.26	0.03
P30101	5 (5)	28.34	1.25e-004	1.85		Protein disulfide-isomerase A3 OS=Homo sapiens OX=9606 GN=PDIA3 PE=1 SV=4	0.11	0.06
P59998	4 (4)	28.31	0.02	2.08		Actin-related protein 2/3 complex subunit 4 OS=Homo sapiens OX=9606 GN=ARPC4 PE=1 SV=3	0.07	0.03
P06702	3 (3)	28.05	0.04	7.83		Protein S100-A9 OS=Homo sapiens OX=9606 GN=S100A9 PE=1 SV=1	0.36	0.05
Q99798	6 (6)	27.77	0.19	1.65		Aconitate hydratase_ mitochondrial OS=Homo sapiens OX=9606 GN=ACO2 PE=1 SV=2	0.05	0.03
P35580	6 (6)	27.70	2.93e-004	2.56		Myosin-10 OS=Homo sapiens OX=9606 GN=MYH10 PE=1 SV=3	0.18	0.07
P05062	4 (4)	27.64	0.08	3.96		Fructose-bisphosphate aldolase B OS=Homo sapiens OX=9606 GN=ALDOB PE=1 SV=2	0.26	0.07
O00592	4 (4)	27.56	5.79e-003	2.79		Podocalyxin OS=Homo sapiens OX=9606 GN=PODXL PE=1 SV=2	0.21	0.07
P01833	4 (4)	25.93	0.04	2.84		Polymeric immunoglobulin receptor OS=Homo sapiens OX=9606 GN=PIGR PE=1 SV=4	0.47	0.17
P68104	5 (5)	25.89	2.70e-003	1.84		Elongation factor 1-alpha 1 OS=Homo sapiens OX=9606 GN=EEF1A1 PE=1 SV=1	0.66	1.21
Q04695	10 (10)	25.77	0.76	1.90		Keratin_ type I cytoskeletal 17 OS=Homo sapiens OX=9606 GN=KRT17 PE=1 SV=2	4.55	2.39
P00918	5 (5)	25.59	4.45e-005	2.96		Carbonic anhydrase 2 OS=Homo sapiens OX=9606 GN=CA2 PE=1 SV=2	1.42	4.21
P05787	5 (5)	24.94	0.70	1.19		Keratin_ type II cytoskeletal 8 OS=Homo sapiens OX=9606 GN=KRT8 PE=1 SV=7	1.22	1.02

Accession	Peptides	Score	Anova (p)*	Fold	Tags	Description	Average Normalised Abundances	
							CTR	MCD
P55290	5 (5)	24.69	2.99e-003	4.15		Cadherin-13 OS=Homo sapiens OX=9606 GN=CDH13 PE=1 SV=1	0.06	0.02
P0DP25	5 (5)	24.13	0.01	2.20		Calmodulin-3 OS=Homo sapiens OX=9606 GN=CALM3 PE=1 SV=1	0.17	0.08
Q7Z406	4 (4)	24.08	0.05	2.38		Myosin-14 OS=Homo sapiens OX=9606 GN=MYH14 PE=1 SV=2	0.13	0.05
P09382	4 (4)	23.67	0.03	2.18		Galectin-1 OS=Homo sapiens OX=9606 GN=LGALS1 PE=1 SV=2	0.08	0.04
P00338	4 (4)	23.64	0.10	3.62		L-lactate dehydrogenase A chain OS=Homo sapiens OX=9606 GN=LDHA PE=1 SV=2	1.20	0.33
P01591	3 (3)	23.31	0.06	9.51		Immunoglobulin J chain OS=Homo sapiens OX=9606 GN=JCHAIN PE=1 SV=4	0.23	0.02
P04075	4 (4)	22.64	0.11	1.41		Fructose-bisphosphate aldolase A OS=Homo sapiens OX=9606 GN=ALDOA PE=1 SV=2	0.13	0.09
P05783	4 (4)	22.40	0.16	1.49		Keratin_ type I cytoskeletal 18 OS=Homo sapiens OX=9606 GN=KRT18 PE=1 SV=2	0.21	0.31
Q01995	4 (4)	22.33	0.03	2.61		Transgelin OS=Homo sapiens OX=9606 GN=TAGLN PE=1 SV=4	0.07	0.03
P06703	3 (3)	22.03	2.55e-004	2.18		Protein S100-A6 OS=Homo sapiens OX=9606 GN=S100A6 PE=1 SV=1	0.46	0.21
P29400	5 (5)	21.75	0.56	1.70		Collagen alpha-5(IV) chain OS=Homo sapiens OX=9606 GN=COL4A5 PE=1 SV=2	0.94	0.55
Q15365	4 (4)	21.62	3.22e-004	2.11		Poly(rC)-binding protein 1 OS=Homo sapiens OX=9606 GN=PCBP1 PE=1 SV=2	0.09	0.04
O00159	4 (4)	21.56	2.51e-004	2.75		Unconventional myosin-Ic OS=Homo sapiens OX=9606 GN=MYO1C PE=1 SV=4	0.04	0.10
Q12931	3 (3)	21.54	7.71e-005	2.97		Heat shock protein 75 kDa_ mitochondrial OS=Homo sapiens OX=9606 GN=TRAP1 PE=1 SV=3	0.78	2.31
P23526	4 (4)	21.15	0.01	1.57		Adenosylhomocysteinase OS=Homo sapiens OX=9606 GN=AHCY PE=1 SV=4	0.09	0.14
O76013	4 (4)	20.46	0.01	1.68		Keratin_ type I cuticular Ha6 OS=Homo sapiens OX=9606 GN=KRT36 PE=1 SV=1	0.18	0.11
Q3LXA3	5 (5)	20.35	1.06e-004	2.77		Triokinase/FMN cyclase OS=Homo sapiens OX=9606 GN=TKFC PE=1 SV=2	0.20	0.55
P26006	3 (3)	20.12	2.25e-004	3.22		Integrin alpha-3 OS=Homo sapiens OX=9606 GN=ITGA3 PE=1 SV=5	0.09	0.03
P02743	3 (3)	19.87	1.06e-003	2.24		Serum amyloid P-component OS=Homo sapiens OX=9606 GN=APCS PE=1 SV=2	0.12	0.05
P23396	3 (3)	19.71	3.98e-003	2.70		40S ribosomal protein S3 OS=Homo sapiens OX=9606 GN=RPS3 PE=1 SV=2	0.05	0.02
Q9NP85	3 (3)	19.48	9.06e-003	9.32		Podocin OS=Homo sapiens OX=9606 GN=NPHS2 PE=1 SV=1	0.03	3.07e-003
P68371	3 (3)	19.33	2.64e-003	2.46		Tubulin beta-4B chain OS=Homo sapiens OX=9606 GN=TUBB4B PE=1 SV=1	0.28	0.12
Q96KP4	3 (3)	19.26	0.18	1.82		Cytosolic non-specific dipeptidase OS=Homo sapiens OX=9606 GN=CNDP2 PE=1 SV=2	0.12	0.07
Q9HC38	4 (4)	19.05	0.55	2.26		Glyoxalase domain-containing protein 4 OS=Homo sapiens OX=9606 GN=GLOD4 PE=1 SV=1	2.23	0.99
P17661	4 (4)	18.74	4.48e-004	2.31		Desmin OS=Homo sapiens OX=9606 GN=DES PE=1 SV=3	1.41	3.26
Q08554	3 (3)	18.71	0.12	3.94		Desmocollin-1 OS=Homo sapiens OX=9606 GN=DSC1 PE=1 SV=2	0.34	0.09
P12110	4 (4)	18.49	0.02	2.73		Collagen alpha-2(VI) chain OS=Homo sapiens OX=9606 GN=COL6A2 PE=1 SV=4	0.15	0.05
P00966	3 (3)	18.24	0.02	3.13		Argininosuccinate synthase OS=Homo sapiens OX=9606 GN=ASS1 PE=1 SV=2	0.16	0.05
P30041	3 (3)	18.23	0.02	2.01		Peroxiredoxin-6 OS=Homo sapiens OX=9606 GN=PRDX6 PE=1 SV=3	0.03	0.02
P09493	3 (3)	18.19	0.04	2.28		Tropomyosin alpha-1 chain OS=Homo sapiens OX=9606 GN=TPM1 PE=1 SV=2	0.07	0.03

Accession	Peptides	Score	Anova (p)*	Fold	Tags	Description	Average Normalised Abundances	
							CTR	MCD
P07900	3 (3)	17.95	0.01	2.27		Heat shock protein HSP 90-alpha OS=Homo sapiens OX=9606 GN=HSP90AA1 PE=1 SV=5	0.07	0.03
Q13885	2 (2)	17.38	4.65e-005	4.70		Tubulin beta-2A chain OS=Homo sapiens OX=9606 GN=TUBB2A PE=1 SV=1	0.08	0.02
P05023	3 (3)	17.36	3.36e-004	3.73		Sodium/potassium-transporting ATPase subunit alpha-1 OS=Homo sapiens OX=9606 GN=ATP1A1 PE=1 SV=1	0.05	0.01
P01040	3 (3)	17.17	0.22	2.42		Cystatin-A OS=Homo sapiens OX=9606 GN=CSTA PE=1 SV=1	0.18	0.07
P14550	3 (3)	17.00	0.10	1.52		Aldo-keto reductase family 1 member A1 OS=Homo sapiens OX=9606 GN=AKR1A1 PE=1 SV=3	0.10	0.16
P07737	3 (3)	16.73	0.02	3.53		Profilin-1 OS=Homo sapiens OX=9606 GN=PFN1 PE=1 SV=2	0.03	7.72e-003
P17066	3 (3)	16.54	0.02	1.99		Heat shock 70 kDa protein 6 OS=Homo sapiens OX=9606 GN=HSPA6 PE=1 SV=2	0.09	0.05
Q9BR76	3 (3)	16.01	0.31	1.32		Coronin-1B OS=Homo sapiens OX=9606 GN=CORO1B PE=1 SV=1	0.06	0.04
P01011	3 (3)	15.90	1.66e-005	2.30		Alpha-1-antichymotrypsin OS=Homo sapiens OX=9606 GN=SERPINA3 PE=1 SV=2	0.24	0.56
Q08380	3 (3)	15.73	0.03	2.76		Galectin-3-binding protein OS=Homo sapiens OX=9606 GN=LGALS3BP PE=1 SV=1	0.09	0.03
Q5D862	3 (3)	15.40	0.10	3.58		Filaggrin-2 OS=Homo sapiens OX=9606 GN=FLG2 PE=1 SV=1	0.36	0.10
Q9NSB2	3 (3)	15.28	0.96	2.11		Keratin_ type II cuticular Hb4 OS=Homo sapiens OX=9606 GN=KRT84 PE=2 SV=2	2.67	1.27
Q9BSE5	3 (3)	15.17	0.10	1.92		Agmatinase_ mitochondrial OS=Homo sapiens OX=9606 GN=AGMAT PE=1 SV=2	0.36	0.19
P52566	3 (3)	15.12	0.04	2.18		Rho GDP-dissociation inhibitor 2 OS=Homo sapiens OX=9606 GN=ARHGDIB PE=1 SV=3	0.11	0.05
O43809	2 (2)	15.03	5.20e-004	3.03		Cleavage and polyadenylation specificity factor subunit 5 OS=Homo sapiens OX=9606 GN=NUDT21 PE=1 SV=1	4.67	14.17
P04040	3 (3)	14.95	7.50e-003	2.17		Catalase OS=Homo sapiens OX=9606 GN=CAT PE=1 SV=3	0.15	0.07
P01859	3 (3)	14.94	1.03e-003	5.70		Immunoglobulin heavy constant gamma 2 OS=Homo sapiens OX=9606 GN=IGHG2 PE=1 SV=2	0.17	0.03
Q13642	3 (3)	14.77	0.14	2.79		Four and a half LIM domains protein 1 OS=Homo sapiens OX=9606 GN=FHL1 PE=1 SV=4	0.02	6.61e-003
P50395	2 (2)	14.64	2.53e-004	4.56		Rab GDP dissociation inhibitor beta OS=Homo sapiens OX=9606 GN=GDI2 PE=1 SV=2	0.13	0.03
P06727	3 (3)	14.56	2.54e-005	4.77		Apolipoprotein A-IV OS=Homo sapiens OX=9606 GN=APOA4 PE=1 SV=4	1.68	8.03
Q14240	3 (3)	14.42	2.75e-006	1.85		Eukaryotic initiation factor 4A-II OS=Homo sapiens OX=9606 GN=EIF4A2 PE=1 SV=2	0.09	0.05
P55060	2 (2)	14.24	0.21	1.64		Exportin-2 OS=Homo sapiens OX=9606 GN=CSE1L PE=1 SV=3	0.19	0.11
P22061	2 (2)	14.11	0.07	3.00		Protein-L-isoaspartate(D-aspartate) O-methyltransferase OS=Homo sapiens OX=9606 GN=PCMT1 PE=1 SV=4	4.25	12.73
P20700	3 (3)	13.75	0.03	1.58		Lamin-B1 OS=Homo sapiens OX=9606 GN=LMNB1 PE=1 SV=2	0.12	0.19
P04899	2 (2)	13.45	8.93e-003	2.24		Guanine nucleotide-binding protein G(i) subunit alpha-2 OS=Homo sapiens OX=9606 GN=GNAI2 PE=1 SV=3	0.06	0.03
P52943	2 (2)	13.22	0.31	1.76		Cysteine-rich protein 2 OS=Homo sapiens OX=9606 GN=CRIP2 PE=1 SV=1	0.03	0.02
P04439	2 (2)	13.21	6.32e-003	2.64			0.06	0.02

Accession	Peptides	Score	Anova (p)*	Fold	Tags	Description	Average Normalised Abundances	
							CTR	MCD
						HLA class I histocompatibility antigen_ A alpha chain OS=Homo sapiens OX=9606 GN=HLA-A PE=1 SV=2		
Q7Z794	3 (3)	13.10	5.55e-006	6.22		Keratin_ type II cytoskeletal 1b OS=Homo sapiens OX=9606 GN=KRT77 PE=1 SV=3	2.20	13.69
Q9BW30	2 (2)	12.91	0.08	2.46		Tubulin polymerization-promoting protein family member 3 OS=Homo sapiens OX=9606 GN=TPPP3 PE=1 SV=1	0.04	0.02
P61626	2 (2)	12.54	0.03	1.72		Lysozyme C OS=Homo sapiens OX=9606 GN=LYZ PE=1 SV=1	0.24	0.14
P21291	2 (2)	12.41	0.03	19.10		Cysteine and glycine-rich protein 1 OS=Homo sapiens OX=9606 GN=CSRP1 PE=1 SV=3	0.02	8.04e-004
P61224	2 (2)	12.40	2.21e-005	3.25		Ras-related protein Rap-1b OS=Homo sapiens OX=9606 GN=RAP1B PE=1 SV=1	0.43	1.41
P05109	2 (2)	12.06	0.04	3.45		Protein S100-A8 OS=Homo sapiens OX=9606 GN=S100A8 PE=1 SV=1	0.20	0.06
P31946	2 (2)	11.91	4.16e-003	5.97		14-3-3 protein beta/alpha OS=Homo sapiens OX=9606 GN=YWHAB PE=1 SV=3	0.04	6.06e-003
P02760	2 (2)	11.68	0.01	2.20		Protein AMBP OS=Homo sapiens OX=9606 GN=AMBP PE=1 SV=1	0.18	0.08
P13646	2 (2)	11.67	0.60	1.61		Keratin_ type I cytoskeletal 13 OS=Homo sapiens OX=9606 GN=KRT13 PE=1 SV=4	6.60	4.11
Q9UN36	2 (2)	11.65	0.01	57.51		Protein NDRG2 OS=Homo sapiens OX=9606 GN=NDRG2 PE=1 SV=2	0.02	3.71e-004
P05089	2 (2)	11.62	0.04	10.11		Arginase-1 OS=Homo sapiens OX=9606 GN=ARG1 PE=1 SV=2	0.17	0.02
P00403	2 (2)	11.44	0.20	1.45		Cytochrome c oxidase subunit 2 OS=Homo sapiens OX=9606 GN=MT-CO2 PE=1 SV=1	0.20	0.14
Q562R1	2 (2)	11.43	6.57e-005	2.05		Beta-actin-like protein 2 OS=Homo sapiens OX=9606 GN=ACTBL2 PE=1 SV=2	0.47	0.23
P06753	2 (2)	11.28	5.09e-004	1.87		Tropomyosin alpha-3 chain OS=Homo sapiens OX=9606 GN=TPM3 PE=1 SV=2	0.07	0.04
P42765	2 (2)	11.19	0.74	1.25		3-ketoacyl-CoA thiolase_ mitochondrial OS=Homo sapiens OX=9606 GN=ACAA2 PE=1 SV=2	0.07	0.09
Q9NZT1	2 (2)	11.13	0.23	1.55		Calmodulin-like protein 5 OS=Homo sapiens OX=9606 GN=CALML5 PE=1 SV=2	0.47	0.30
P52758	2 (2)	11.09	1.73e-004	2.63		2-iminobutanoate/2-iminopropanoate deaminase OS=Homo sapiens OX=9606 GN=RIDA PE=1 SV=1	1.13	2.99
Q13228	2 (2)	11.08	6.28e-003	3.80		Methanethiol oxidase OS=Homo sapiens OX=9606 GN=SELENBP1 PE=1 SV=2	0.12	0.03
P61978	2 (2)	11.00	0.08	1.96		Heterogeneous nuclear ribonucleoprotein K OS=Homo sapiens OX=9606 GN=HNRNPK PE=1 SV=1	0.03	0.01
P13797	3 (3)	10.86	0.07	2.15		Plastin-3 OS=Homo sapiens OX=9606 GN=PLS3 PE=1 SV=4	0.15	0.07
P54652	2 (2)	10.80	0.16	2.39		Heat shock-related 70 kDa protein 2 OS=Homo sapiens OX=9606 GN=HSPA2 PE=1 SV=1	0.04	0.01
P37837	2 (2)	10.77	0.28	1.35		Transaldolase OS=Homo sapiens OX=9606 GN=TALDO1 PE=1 SV=2	0.06	0.05
P09496	2 (2)	10.73	0.05	5.56		Clathrin light chain A OS=Homo sapiens OX=9606 GN=CLTA PE=1 SV=1	0.42	0.08
Q7Z4W1	2 (2)	10.72	0.05	2.03		L-xylulose reductase OS=Homo sapiens OX=9606 GN=DCXR PE=1 SV=2	0.16	0.08
P30038	2 (2)	10.70	0.33	1.94		Delta-1-pyrroline-5-carboxylate dehydrogenase_ mitochondrial OS=Homo sapiens OX=9606 GN=ALDH4A1 PE=1 SV=3	0.04	0.02
Q9UBI6	2 (2)	10.69	3.36e-003	3.80		Guanine nucleotide-binding protein G (I)/G(S)/G(O) subunit gamma-12	0.09	0.02

Accession	Peptides	Score	Anova (p)*	Fold	Tags	Description	Average Normalised Abundances	
							CTR	MCD
						OS=Homo sapiens OX=9606 GN=GNG12 PE=1 SV=3		
P10809	2 (2)	10.69	0.55	1.19		60 kDa heat shock protein_ mitochondrial OS=Homo sapiens OX=9606 GN=HSPD1 PE=1 SV=2	0.16	0.19
P14923	2 (2)	10.67	0.05	3.27		Junction plakoglobin OS=Homo sapiens OX=9606 GN=JUP PE=1 SV=3	0.23	0.07
P38646	2 (2)	10.50	1.52e-006	3.28		Stress-70 protein_ mitochondrial OS=Homo sapiens OX=9606 GN=HSPA9 PE=1 SV=2	0.20	0.67
P12277	2 (2)	10.39	3.81e-004	2.83		Creatine kinase B-type OS=Homo sapiens OX=9606 GN=CKB PE=1 SV=1	0.05	0.02
P23246	2 (2)	10.28	0.05	2.60		Splicing factor_ proline- and glutamine-rich OS=Homo sapiens OX=9606 GN=SFPQ PE=1 SV=2	0.02	7.72e-003
P61313	2 (2)	10.23	7.35e-004	1.84		60S ribosomal protein L15 OS=Homo sapiens OX=9606 GN=RPL15 PE=1 SV=2	0.10	0.05
P62424	2 (2)	10.12	0.09	1.75		60S ribosomal protein L7a OS=Homo sapiens OX=9606 GN=RPL7A PE=1 SV=2	0.09	0.05
Q15366	2 (2)	9.98	0.02	2.17		Poly(rC)-binding protein 2 OS=Homo sapiens OX=9606 GN=PCBP2 PE=1 SV=1	0.29	0.13
P50995	2 (2)	9.84	0.01	1.90		Annexin A11 OS=Homo sapiens OX=9606 GN=ANXA11 PE=1 SV=1	0.03	0.02
Q14195	2 (2)	9.60	0.03	1.97		Dihydropyrimidinase-related protein 3 OS=Homo sapiens OX=9606 GN=DPYSL3 PE=1 SV=1	0.05	0.02
P07196	2 (2)	9.46	0.03	1.94		Neurofilament light polypeptide OS=Homo sapiens OX=9606 GN=NEFL PE=1 SV=3	0.05	0.03
O95571	2 (2)	9.45	0.12	1.97		Persulfide dioxygenase ETHE1_ mitochondrial OS=Homo sapiens OX=9606 GN=ETHE1 PE=1 SV=2	0.01	5.73e-003
P09211	2 (2)	9.32	0.01	4.63		Glutathione S-transferase P OS=Homo sapiens OX=9606 GN=GSTP1 PE=1 SV=2	0.04	7.67e-003
Q96AC1	2 (2)	8.82	0.84	1.17		Fermitin family homolog 2 OS=Homo sapiens OX=9606 GN=FERMT2 PE=1 SV=1	0.01	9.77e-003
P16104	3 (3)	8.52	0.52	1.11		Histone H2AX OS=Homo sapiens OX=9606 GN=H2AX PE=1 SV=2	0.54	0.60
Q99456	2 (2)	8.33	8.20e-005	3.05		Keratin_ type I cytoskeletal 12 OS=Homo sapiens OX=9606 GN=KRT12 PE=1 SV=1	0.78	2.39
Q2TBA0	1 (1)	7.77	2.20e-006	5.92		Kelch-like protein 40 OS=Homo sapiens OX=9606 GN=KLHL40 PE=1 SV=2	0.99	5.83
P08590	1 (1)	7.69	0.31	13.85		Myosin light chain 3 OS=Homo sapiens OX=9606 GN=MYL3 PE=1 SV=3	0.01	0.16
P16520	1 (1)	7.30	2.00e-003	2.67		Guanine nucleotide-binding protein G (I)/G(S)/G(T) subunit beta-3 OS=Homo sapiens OX=9606 GN=GNB3 PE=1 SV=1	0.11	0.04
A0A0A0MRZ8	1 (1)	7.25	5.30e-003	4.92		Immunoglobulin kappa variable 3D-11 OS=Homo sapiens OX=9606 GN=IGKV3D-11 PE=3 SV=6	0.15	0.03
A0A0B4J1V1	1 (1)	7.23	5.80e-004	4.36		Immunoglobulin heavy variable 3-21 OS=Homo sapiens OX=9606 GN=IGHV3-21 PE=1 SV=1	0.09	0.02
P48741	1 (1)	7.21	1.42e-004	3.57		Putative heat shock 70 kDa protein 7 OS=Homo sapiens OX=9606 GN=HSPA7 PE=5 SV=2	3.15	11.26
P52565	1 (1)	7.17	1.33e-003	4.16		Rho GDP-dissociation inhibitor 1 OS=Homo sapiens OX=9606 GN=ARHGDIA PE=1 SV=3	0.09	0.02
Q14103	1 (1)	7.15	0.35	1.36		Heterogeneous nuclear ribonucleoprotein D0 OS=Homo sapiens OX=9606 GN=HNRNPD PE=1 SV=1	0.11	0.08
P16444	1 (1)	6.75	0.01	5.39		Dipeptidase 1 OS=Homo sapiens OX=9606 GN=DPEP1 PE=1 SV=3	0.12	0.02
P51153	1 (1)	6.75	0.42	1.14		Ras-related protein Rab-13 OS=Homo sapiens OX=9606 GN=RAB13 PE=1 SV=1	0.05	0.04

Accession	Peptides	Score	Anova (p)*	Fold	Tags	Description	Average Normalised Abundances	
							CTR	MCD
P27348	1 (1)	6.61	3.34e-003	29.31		14-3-3 protein theta OS=Homo sapiens OX=9606 GN=YWHAQ PE=1 SV=1	0.02	5.27e-004
Q9BVC6	1 (1)	6.58	0.24	1.35		Transmembrane protein 109 OS=Homo sapiens OX=9606 GN=TMEM109 PE=1 SV=1	0.08	0.06
P01619	1 (1)	6.58	5.36e-003	3.26		Immunoglobulin kappa variable 3-20 OS=Homo sapiens OX=9606 GN=IGKV3-20 PE=1 SV=2	0.07	0.02
Q9BVQ7	1 (1)	6.56	0.83	1.04		Spermatogenesis-associated protein 5-like protein 1 OS=Homo sapiens OX=9606 GN=SPATA5L1 PE=1 SV=2	0.08	0.08
Q9NVA2	1 (1)	6.54	0.02	3.24		Septin-11 OS=Homo sapiens OX=9606 GN=SEPTIN11 PE=1 SV=3	0.11	0.03
Q15517	1 (1)	6.54	0.17	1.98		Corneodesmosin OS=Homo sapiens OX=9606 GN=CDSN PE=1 SV=3	0.35	0.18
P01889	1 (1)	6.51	2.00e-003	6.23		HLA class I histocompatibility antigen_ B alpha chain OS=Homo sapiens OX=9606 GN=HLA-B PE=1 SV=3	0.07	0.01
P31944	1 (1)	6.45	0.05	2.26		Caspase-14 OS=Homo sapiens OX=9606 GN=CASP14 PE=1 SV=2	0.44	0.20
Q86UL8	1 (1)	6.37	6.81e-003	4.65		Membrane-associated guanylate kinase_ WW and PDZ domain-containing protein 2 OS=Homo sapiens OX=9606 GN=MAGI2 PE=1 SV=3	0.05	0.01
Q02252	1 (1)	6.32	0.84	1.16		Methylmalonate-semialdehyde dehydrogenase [acylating]_ mitochondrial OS=Homo sapiens OX=9606 GN=ALDH6A1 PE=1 SV=2	0.08	0.07
P52209	1 (1)	6.22	0.02	4.53		6-phosphogluconate dehydrogenase_ decarboxylating OS=Homo sapiens OX=9606 GN=PGD PE=1 SV=3	0.03	6.80e-003
P47755	1 (1)	6.18	0.90	1.02		F-actin-capping protein subunit alpha-2 OS=Homo sapiens OX=9606 GN=CAPZA2 PE=1 SV=3	0.10	0.10
P01877	1 (1)	6.13	0.03	3.55		Immunoglobulin heavy constant alpha 2 OS=Homo sapiens OX=9606 GN=IGHA2 PE=1 SV=4	0.04	9.85e-003
P02748	1 (1)	6.11	8.34e-003	2.71		Complement component C9 OS=Homo sapiens OX=9606 GN=C9 PE=1 SV=2	0.05	0.02
P18124	1 (1)	6.10	0.03	2.06		60S ribosomal protein L7 OS=Homo sapiens OX=9606 GN=RPL7 PE=1 SV=1	0.06	0.03
Q969L2	1 (1)	6.07	0.05	2.78		Protein MAL2 OS=Homo sapiens OX=9606 GN=MAL2 PE=1 SV=1	0.07	0.03
Q9BVM4	1 (1)	6.06	0.06	4.09		Gamma-glutamylaminecyclotransferase OS=Homo sapiens OX=9606 GN=GGACT PE=1 SV=2	0.04	0.01
Q00796	1 (1)	6.05	0.18	2.50		Sorbitol dehydrogenase OS=Homo sapiens OX=9606 GN=SORD PE=1 SV=4	0.06	0.02
P14649	1 (1)	6.03	0.93	1.02		Myosin light chain 6B OS=Homo sapiens OX=9606 GN=MYL6B PE=1 SV=1	0.68	0.69
P13489	1 (1)	6.00	2.44e-003	3.61		Ribonuclease inhibitor OS=Homo sapiens OX=9606 GN=RNH1 PE=1 SV=2	0.04	0.01
Q9H299	1 (1)	5.98	2.70e-005	3.74		SH3 domain-binding glutamic acid-rich-like protein 3 OS=Homo sapiens OX=9606 GN=SH3BGRL3 PE=1 SV=1	0.06	0.02
P62942	1 (1)	5.94	0.11	1.74		Peptidyl-prolyl cis-trans isomerase FKBP1A OS=Homo sapiens OX=9606 GN=FKBP1A PE=1 SV=2	0.05	0.03
P35237	1 (1)	5.94	1.11e-003	22.01		Serpin B6 OS=Homo sapiens OX=9606 GN=SERPINB6 PE=1 SV=3	0.03	1.28e-003
P61158	1 (1)	5.92	1.97e-007	6.96		Actin-related protein 3 OS=Homo sapiens OX=9606 GN=ACTR3 PE=1 SV=3	0.08	0.01
P50453	1 (1)	5.83	3.26e-004	14.53		Serpin B9 OS=Homo sapiens OX=9606 GN=SERPINB9 PE=1 SV=1	0.02	1.50e-003
Q92597	1 (1)	5.82	0.04	1.32		Protein NDRG1 OS=Homo sapiens OX=9606 GN=NDRG1 PE=1 SV=1	0.07	0.09
Q96A08	1 (1)	5.82	0.19	2.01			0.18	0.09

Accession	Peptides	Score	Anova (p)*	Fold	Tags	Description	Average Normalised Abundances	
							CTR	MCD
						Histone H2B type 1-A OS=Homo sapiens OX=9606 GN=H2BC1 PE=1 SV=3		
P45880	1 (1)	5.79	2.50e-003	2.99		Voltage-dependent anion-selective channel protein 2 OS=Homo sapiens OX=9606 GN=VDAC2 PE=1 SV=2	0.08	0.03
P07858	1 (1)	5.72	0.22	1.97		Cathepsin B OS=Homo sapiens OX=9606 GN=CTSB PE=1 SV=3	0.61	0.31
P02766	1 (1)	5.72	0.08	2.58		Transthyretin OS=Homo sapiens OX=9606 GN=TTR PE=1 SV=1	0.04	0.01
P59910	1 (1)	5.66	2.44e-004	2.95		DnaJ homolog subfamily B member 13 OS=Homo sapiens OX=9606 GN=DNAJB13 PE=1 SV=1	0.88	2.60
P61604	1 (1)	5.62	0.87	1.09		10 kDa heat shock protein_ mitochondrial OS=Homo sapiens OX=9606 GN=HSPE1 PE=1 SV=2	0.10	0.10
P12035	1 (1)	5.62	0.04	5.22		Keratin_ type II cytoskeletal 3 OS=Homo sapiens OX=9606 GN=KRT3 PE=1 SV=3	0.12	0.02
P61106	1 (1)	5.57	8.90e-003	39.83		Ras-related protein Rab-14 OS=Homo sapiens OX=9606 GN=RAB14 PE=1 SV=4	9.15e-003	2.30e-004
P51148	1 (1)	5.55	0.04	2.77		Ras-related protein Rab-5C OS=Homo sapiens OX=9606 GN=RAB5C PE=1 SV=2	0.03	9.60e-003
A0A075B6P5	1 (1)	5.55	3.55e-004	3.95		Immunoglobulin kappa variable 2-28 OS=Homo sapiens OX=9606 GN=IGKV2-28 PE=3 SV=1	0.11	0.03
P22528	1 (1)	5.53	0.04	5.87		Cornifin-B OS=Homo sapiens OX=9606 GN=SPRR1B PE=1 SV=2	0.16	0.03
Q9BQI9	1 (1)	5.49	0.14	3.38		Nuclear receptor-interacting protein 2 OS=Homo sapiens OX=9606 GN=NRIP2 PE=1 SV=3	0.01	4.39e-003
Q14894	1 (1)	5.47	2.39e-004	3.51		Ketimine reductase mu-crystallin OS=Homo sapiens OX=9606 GN=CRYM PE=1 SV=1	0.07	0.02
P07339	1 (1)	5.47	0.31	1.49		Cathepsin D OS=Homo sapiens OX=9606 GN=CTSD PE=1 SV=1	0.08	0.06
P28072	1 (1)	5.44	1.49e-004	2.83		Proteasome subunit beta type-6 OS=Homo sapiens OX=9606 GN=PSMB6 PE=1 SV=4	0.25	0.71
P62750	1 (1)	5.40	0.60	1.15		60S ribosomal protein L23a OS=Homo sapiens OX=9606 GN=RPL23A PE=1 SV=1	0.06	0.06
P62258	1 (1)	5.39	7.96e-009	4.71		14-3-3 protein epsilon OS=Homo sapiens OX=9606 GN=YWHAE PE=1 SV=1	0.17	0.04
Q2M2I5	1 (1)	5.38	5.72e-004	3.29		Keratin_ type I cytoskeletal 24 OS=Homo sapiens OX=9606 GN=KRT24 PE=1 SV=1	10.23	33.64
P14136	1 (1)	5.37	5.08e-003	31.51		Glial fibrillary acidic protein OS=Homo sapiens OX=9606 GN=GFAP PE=1 SV=1	0.02	7.45e-004
O75971	1 (1)	5.36	5.59e-004	3.27		snRNA-activating protein complex subunit 5 OS=Homo sapiens OX=9606 GN=SNAPC5 PE=1 SV=1	24.20	79.12
P19013	1 (1)	5.34	0.22	1.76		Keratin_ type II cytoskeletal 4 OS=Homo sapiens OX=9606 GN=KRT4 PE=1 SV=5	0.63	0.36
P47914	1 (1)	5.28	0.42	1.41		60S ribosomal protein L29 OS=Homo sapiens OX=9606 GN=RPL29 PE=1 SV=2	0.03	0.02
P04259	1 (1)	5.28	0.04	8.17		Keratin_ type II cytoskeletal 6B OS=Homo sapiens OX=9606 GN=KRT6B PE=1 SV=5	0.33	0.04
P07305	1 (1)	5.27	4.71e-004	2.34		Histone H1.0 OS=Homo sapiens OX=9606 GN=H1-0 PE=1 SV=3	0.21	0.09
P29401	1 (1)	5.24	2.22e-003	6.17		Transketolase OS=Homo sapiens OX=9606 GN=TKT PE=1 SV=3	0.03	5.15e-003
P02792	1 (1)	5.17	0.51	1.46		Ferritin light chain OS=Homo sapiens OX=9606 GN=FTL PE=1 SV=2	0.10	0.07
Q9H0W9	1 (1)	5.04	0.04	6.14		Ester hydrolase C11orf54 OS=Homo sapiens OX=9606 GN=C11orf54 PE=1 SV=1	0.13	0.02
P69892	3 (3)	5.04	0.11	4.03			0.11	0.03

Accession	Peptides	Score	Anova (p)*	Fold	Tags	Description	Average Normalised Abundances	
							CTR	MCD
						Hemoglobin subunit gamma-2 OS=Homo sapiens OX=9606 GN=HBG2 PE=1 SV=2		
P02008	1 (1)	5.00	2.39e-004	4.04		Hemoglobin subunit zeta OS=Homo sapiens OX=9606 GN=HBZ PE=1 SV=2	17.83	72.07
Q9H082	1 (1)	4.91	8.57e-003	2.81		Ras-related protein Rab-33B OS=Homo sapiens OX=9606 GN=RAB33B PE=1 SV=1	0.05	0.02
P08311	1 (1)	4.90	0.05	2.26		Cathepsin G OS=Homo sapiens OX=9606 GN=CTSG PE=1 SV=2	0.06	0.03
P04350	1 (1)	4.83	0.10	17.38		Tubulin beta-4A chain OS=Homo sapiens OX=9606 GN=TUBB4A PE=1 SV=2	0.02	9.68e-004
Q58FF8	1 (1)	4.77	9.45e-003	2.70		Putative heat shock protein HSP 90-beta 2 OS=Homo sapiens OX=9606 GN=HSP90AB2P PE=1 SV=2	0.16	0.06
A0A075B6H7	1 (1)	4.63	0.01	4.34		Probable non-functional immunoglobulin kappa variable 3-7 OS=Homo sapiens OX=9606 GN=IGKV3-7 PE=1 SV=1	0.02	4.00e-003
P21695	1 (1)	4.62	3.17e-003	2.72		Glycerol-3-phosphate dehydrogenase [NAD(+)]_ cytoplasmic OS=Homo sapiens OX=9606 GN=GPD1 PE=1 SV=4	0.13	0.05
Q9NZ72	1 (1)	4.38	0.14	2.55		Stathmin-3 OS=Homo sapiens OX=9606 GN=STMN3 PE=1 SV=3	0.19	0.07
P78417	1 (1)	4.29	0.03	3.31		Glutathione S-transferase omega-1 OS=Homo sapiens OX=9606 GN=GSTO1 PE=1 SV=2	0.06	0.02
Q3MJ62	1 (1)	4.17	8.11e-008	7.09		Zinc finger and SCAN domain-containing protein 23 OS=Homo sapiens OX=9606 GN=ZSCAN23 PE=1 SV=1	0.30	2.15
Q9BUT1	1 (1)	4.13	2.45e-006	7.32		3-hydroxybutyrate dehydrogenase type 2 OS=Homo sapiens OX=9606 GN=BDH2 PE=1 SV=2	0.39	2.86
P12882	1 (1)	3.84	0.64	1.25		Myosin-1 OS=Homo sapiens OX=9606 GN=MYH1 PE=1 SV=3	0.17	0.21
Q9H2M3	1 (1)	3.53	8.77e-007	4.77		S-methylmethionine--homocysteine S-methyltransferase BHMT2 OS=Homo sapiens OX=9606 GN=BHMT2 PE=1 SV=1	0.64	3.06
Q8N257	2 (2)	0.00	0.12	1.42		Histone H2B type 3-B OS=Homo sapiens OX=9606 GN=H2BU1 PE=1 SV=3	0.10	0.07
Q5TEC6	2 (2)	0.00	0.91	1.02		Histone HIST2H3PS2 OS=Homo sapiens OX=9606 GN=H3-2 PE=1 SV=1	0.25	0.24
P48668	2 (2)	0.00	0.10	4.88		Keratin_ type II cytoskeletal 6C OS=Homo sapiens OX=9606 GN=KRT6C PE=1 SV=3	0.31	0.06

Tags	
	reverse
	no peptides
	one unique peptide
	Anova p-value ≤ 0.05
	Max fold change ≥ 1.5

P13645

Keratin_ type I cytoskeletal 10 OS=Homo sapiens OX=9606 GN=KRT10 PE=1 SV=6
72 peptides

Sequence	Peptide Ion	Score	Hits	Mass	Charge	Tags	Conflicts	Modifications	In quantitation	Drift time (ms)

Sequence	Peptide Ion	Score	Hits	Mass	Charge	Tags	Conflicts	Modifications	In quantitation	Drift time (ms)
ADINGLRR	729	0.00	1	913.5076	2		0		no	3.95
ADLEMQIESLTEELAYLK	4212	8.12	1	2095.0314	3		0		no	5.33
ADLEMQIESLTEELAYLK	12271	---	---	2095.0292	2		0		no	8.17
ADLEMQIESLTEELAYLKK	706	9.29	10	2223.1341	3		0		no	5.61
ADLEMQIESLTEELAYLKK	1019	6.87	1	2239.1253	3		0	[2] Hydroxyl DKNP	no	5.61
ADLEMQIESLTEELAYLKK	1276	7.16	3	2239.1250	3		0	[2] Hydroxyl DKNP	no	5.61
AEQNRK	3405	0.00	1	744.3876	1		0		no	8.24
AETECQNTEYQQLLDIK	257	9.42	16	2081.9627	3		0	[5] Carbamidomethyl C	no	4.85
AETECQNTEYQQLLDIK	248	9.42	16	2081.9579	2		0	[5] Carbamidomethyl C	no	7.55
+AETECQNTEYQQLLDIK	3561	0.00	1	2064.9369	3		0	[N-term] Ammonia-loss | [5] Carbamidomethyl C	no	4.50
ALEESNYELEGK	105	9.43	16	1380.6461	2		0		no	5.47
AWFNEK	555	0.00	1	793.3756	1		0		no	8.73
DAEAWFNEK	3744	7.65	1	1108.4891	2		0		no	4.29
DAEAWFNEK	5010	7.34	1	1124.4881	2		0	[1] Hydroxyl DKNP	no	4.43
DAEAWFNEK	2303	7.64	1	1124.4822	2		0	[9] Hydroxyl DKNP	no	4.43
DELTLTK	163	0.00	10	818.4396	1		0		no	9.14
EADINGLRR	576	0.00	1	1042.5523	2		0		no	4.22
ELTLTK	1897	0.00	1	703.4109	1		0		no	8.03
ELTTEIDNNIEQISSYK	920	9.62	8	1995.9650	3		0		no	4.71
ELTTEIDNNIEQISSYK	527	9.62	12	1995.9622	2		0		no	7.41
ENEVALR	3020	0.00	1	829.4277	1		0		no	8.86
GSLGGGFSSGGFSGGSFSR	5548	9.17	2	1706.7647	3		0		no	4.50
GSLGGGFSSGGFSGGSFSR	242	9.17	14	1706.7659	2		0		no	6.37
GSSGGGCFGGSSGGYGGLGGFGGGSFR	1362	9.06	2	2341.9734	3		0	[7] Carbamidomethyl C	no	6.09
GSSGGGCFGGSSGGYGGLGGFGGGSFR	3471	9.06	2	2341.9761	2		0	[7] Carbamidomethyl C	no	8.10
IKEWYEK	1884	8.89	2	994.5190	3		0		no	2.91
IQTYR	1089	0.00	1	679.3656	1		0		no	7.89
IQTYR	2318	0.00	1	679.3656	2		0		no	3.39
IRLEN	6284	---	---	625.3542	2		0		no	3.05
IRLEN	904	0.00	1	625.3555	1		0		no	7.41
IRLENEI	489	0.00	2	867.4804	2		0		no	3.88
IRLENEI	1055	---	---	867.4814	1		0		no	9.90
IRLENEIQ	888	0.00	1	995.5400	2		0		no	4.16
IRLENEIQTYR	237	8.60	14	1433.7643	2		0		no	5.75
IRLENEIQTYR	158	8.60	14	1433.7714	3		0		no	3.74
KDAEAWFNEK	1947	9.39	2	1236.5760	3		0		no	3.46
KDAEAWFNEK	744	9.39	5	1236.5801	2		0		no	4.92
LAADDFR	57	9.19	16	806.3969	2		0		no	3.67

Sequence	Peptide Ion	Score	Hits	Mass	Charge	Tags	Conflicts	Modifications	In quantitation	Drift time (ms)
LAADDFR	267	9.19	11	806.3930	1		0		no	9.00
LAADDFRLK	770	6.78	1	1047.5757	2		0		no	4.50
LASYLDK	674	8.92	7	808.4332	2		0		no	3.46
LASYLDK	7420	---	---	808.4345	1		0		no	9.70
LASYLDKVR	3753	---	---	1063.6024	3		0		no	3.32
LASYLDKVR	2291	8.15	2	1063.6030	2		0		no	4.78
LDELTLTK	338	0.00	4	931.5217	1		0		no	10.80
LDELTLTK	3253	---	---	931.5217	2		0		no	3.88
LENEIQTYR	5586	---	---	1164.5761	1		0		no	11.98
LENEIQTYR	138	9.30	16	1164.5794	2		0		no	4.85
+LENEIQTYR	4139	0.00	1	1147.5334	2		0	[N-term] Ammonia-loss	no	4.64
LKYEN	2778	---	---	647.3276	2		0		no	3.12
LKYEN	717	0.00	1	647.3281	1		0		no	7.55
LKYENE	595	0.00	2	776.3709	2		0		no	3.39
LKYENE	179	0.00	3	776.3702	1		0		no	8.73
LKYENEVALR	153	8.07	15	1233.6767	2		0		yes	5.12
LKYENEVALR	135	8.07	15	1233.6796	3		0		yes	3.74
+LKYENEVALR	308	0.00	3	1216.6498	3		0	[N-term] Ammonia-loss	no	3.74
NEIQTYR	3145	0.00	1	922.4487	1		0		no	9.76
NEVALR	1532	0.00	2	700.3868	1		0		no	7.96
NHEEEMKDLR	3323	8.08	1	1299.5879	2		0		no	5.33
NHEEEMKDLR	285	8.36	15	1299.5945	3		0		no	3.74
+NHEEEMKDLR	3352	0.00	1	1282.5602	3		0	[N-term] Ammonia-loss	no	3.67
NHEEEMKDLR	1668	6.81	1	1315.5845	3		0	[1] Hydroxyl DKNP	no	3.74
NHEEEMKDLR	835	7.91	2	1315.5849	3		0	[6] Oxidation M	no	3.74
NHEEEMKDLR	968	7.97	1	1315.5843	3		0	[8] Hydroxyl DKNP	no	3.74
NVQALEIELQSQLALK	2388	8.74	3	1796.0033	3		0		no	4.64
NVQALEIELQSQLALK	5006	8.74	2	1795.9882	2		0		no	6.99
NVSTGDVNVEMNAAPGVDLTQLLNNMR	3933	7.65	1	2887.3644	3		0	[15] Hydroxyl DKNP	no	7.55
NVSTGDVNVEMNAAPGVDLTQLLNNMR	451	7.47	1	2903.3651	3		0	[1] Hydroxyl DKNP | [24] Hydroxyl DKNP	no	7.34
QSLEASLAETEGR	98	9.37	15	1389.6784	2		0		yes	5.33
QSLEASLAETEGR	1548	9.37	1	1389.6763	3		0		yes	3.95
QSVEADINGLR	360	9.28	14	1200.6123	2		0		no	4.78
QSVEADINGLR+	3470	0.00	1	1182.5986	2		0	[C-term] Dehydrated	no	4.71
QSVEADINGLRR	145	8.52	15	1356.7198	3		0		no	3.74
QSVEADINGLRR	204	8.52	7	1356.7137	2		0		no	5.61
RVLDELTLTK	6318	---	---	1186.6922	3		0		no	3.39
RVLDELTLTK	716	7.75	6	1186.6914	2		0		no	4.85
SEITELR	892	8.74	3	846.4448	2		0		no	3.81
SEITELRR	800	8.33	7	1002.5508	3		0		no	3.05
SEITELRR	136	8.79	15	1002.5509	2		0		no	4.29

Sequence	Peptide Ion	Score	Hits	Mass	Charge	Tags	Conflicts	Modifications	In quantitation	Drift time (ms)
SGGGGGGGGCGGGGGVSSLR	4219	8.58	2	1548.6674	2		0	[10] Carbamidomethyl C	no	5.61
SKELTTEIDNNIEQISSYK	2441	9.26	2	2211.0881	2		0		no	8.03
SKELTTEIDNNIEQISSYK	222	9.26	16	2211.0984	3		0		no	4.78
SLLEGEGSSGGGGR	126	9.22	16	1261.5945	2		0		no	4.71
SQYEQLAEQNR	410	9.43	16	1364.6342	2		0		no	5.26
SQYEQLAEQNR	4116	9.43	2	1364.6364	3		0		no	3.53
SQYEQLAEQNRK	479	9.29	16	1492.7287	2		0		no	6.02
SQYEQLAEQNRK	104	9.29	16	1492.7402	3		0		yes	3.88
+SQYEQLAEQNRK	832	0.00	2	1475.7025	3		0	[N-term] Ammonia-loss	no	3.81
TELRR	1489	0.00	1	673.3882	2		0		no	3.19
TELRR	2439	0.00	1	673.3880	1		0		no	7.83
TIDDLKNQILNLTTDNANILLQIDNAR	3568	8.65	2	3051.6207	3		0		no	6.37
VLDELTLTK	861	8.63	2	1030.5903	1		0		no	11.70
VLDELTLTKADLEMQIESLTEELAYLK	2179	8.63	2	3107.6170	3		0		no	6.86
VLDELTLTKADLEMQIESLTEELAYLK	5159	---	---	3107.6018	4		0		no	5.68
VLDELTLTKADLEMQIESLTEELAYLK	10660	---	---	3123.5993	4		0	[9] Hydroxyl DKNP	no	5.68
VLDELTLTKADLEMQIESLTEELAYLK	4567	7.35	1	3123.6012	3		0	[9] Hydroxyl DKNP	no	6.72
VRALEESNYELEGK	4068	6.69	2	1635.8140	3		0		no	4.16
VTMQNLNDR	2842	8.48	2	1089.5278	2		0		no	4.43
VTMQNLNDR	2058	6.84	4	1105.5180	2		0	[3] Oxidation M	no	4.43
VTMQNLNDR	619	8.72	7	1105.5192	2		0	[3] Oxidation M	no	4.43
WFNEK	705	0.00	1	722.3395	1		0		no	7.89
YCVQLSQIQAQISALEEQLQQIR	588	9.52	4	2745.4067	3		0	[2] Carbamidomethyl C	no	6.99
YENEVALR	3109	0.00	1	992.4920	1		0		no	10.25
YENEVALR	1338	0.00	1	992.4987	2		0		no	4.36
YENEVALR	202	9.22	16	992.4955	2		0		no	4.36
YEQLAEQNRK	12458	0.00	6	1277.6330	2		0		no	3.88
YEQLAEQNRK	488	0.00	8	1277.6385	2		0		no	5.19

Tags	
	Anova p-value ≤ 0.05
	power>0.8
	q<0.05

P02768

Albumin OS=Homo sapiens OX=9606 GN=ALB PE=1 SV=2
53 peptides

Sequence	Peptide Ion	Score	Hits	Mass	Charge	Tags	Conflicts	Modifications	In quantitation	Drift time (ms)	Av No Ab
											CT
AACLLPK	1774	7.72	2	771.4313	1		0	[3] Carbamidomethyl C	no	9.35	
AACLLPK	482	7.72	4	771.4315	2		0	[3] Carbamidomethyl C	no	3.67	
AAFTECCQAADK	1353	8.81	4	1370.5608	2		0	[6] Carbamidomethyl C|[7] Carbamidomethyl C	no	5.26	
ADDKETCFAEEGK	2821	8.02	4	1498.6245	3		0	[7] Carbamidomethyl C	no	3.88	
AEFAEVSK	4888	8.74	1	879.4331	1		0		no	9.76	
AEFAEVSK	407	8.87	6	879.4357	2		0		no	3.67	
AEFAEVSKLVTDLTK	7168	6.70	1	1649.8880	3		0		no	4.50	
AVMDDFAAFVEK	689	8.05	2	1357.6238	2		0	[5] Hydroxyl DKNP	no	5.19	
AVMDDFAAFVEK	3116	7.11	3	1373.6206	2		0	[4] Hydroxyl DKNP|[5] Hydroxyl DKNP	no	5.19	
AVMDDFAAFVEKCCK	7221	6.79	1	1819.7946	3		0	[4] Hydroxyl DKNP|[13] Carbamidomethyl C|[14] Carbamidomethyl C|[15] Methyl K	no	4.57	
CCAAADPHECYAK	11052	---	---	1551.5907	2		0	[1] Carbamidomethyl C|[2] Carbamidomethyl C|[10] Carbamidomethyl C	no	5.89	
CCAAADPHECYAK	1114	8.34	4	1551.5924	3		0	[1] Carbamidomethyl C|[2] Carbamidomethyl C|[10] Carbamidomethyl C	no	4.22	
CCAAADPHECYAK	12025	6.83	1	1583.5838	2		0	[1] Carbamidomethyl C|[2] Carbamidomethyl C|[7] Hydroxyl DKNP|[10] Carbamidomethyl C|[13] Hydroxyl DKNP	no	5.82	
CCTESLVNR	207	9.20	8	1137.4968	2		0	[1] Carbamidomethyl C|[2] Carbamidomethyl C	yes	4.64	1.7[illegible]
CTVATLR	3674	0.00	1	819.4266	1		0	[1] Carbamidomethyl C	no	9.14	
DDNPNLPR	1171	8.28	4	939.4436	2		0		no	4.09	
DELRDEGK	437	0.00	1	960.4555	2		0		no	3.95	
DVFLGMFLYEYAR	3021	7.91	2	1638.7736	2		0	[6] Oxidation M	no	6.44	
ECCEKPLLEK	1220	7.69	4	1304.6111	3		0	[2] Carbamidomethyl C|[3]	no	3.81	

Sequence	Peptide Ion	Score	Hits	Mass	Charge	Tags	Conflicts	Modifications	In quantitation	Drift time (ms)	Av No Ab
											CT
								Carbamidomethyl C			
ECCEKPLLEK	4171	7.69	2	1304.6103	2		0	[2] Carbamidomethyl C | [3] Carbamidomethyl C	no	5.40	
EFAEVSK	4759	0.00	1	808.3949	1		0		no	8.80	
ETYGEMADCCAK	10321	7.40	1	1433.5392	2		0	[9] Carbamidomethyl C | [10] Carbamidomethyl C	no	5.47	
ETYGEMADCCAK	664	8.18	4	1449.5211	2		0	[6] Oxidation M | [9] Carbamidomethyl C | [10] Carbamidomethyl C	no	5.40	
FAEVSK	906	0.00	3	679.3545	1		0		no	7.96	
FQNALLVR	765	8.25	4	959.5553	2		0		no	4.16	
KECCEKPLLEK	5126	0.00	1	1432.7057	3		0	[3] Carbamidomethyl C | [4] Carbamidomethyl C	no	4.09	
KLVAASQAALGL	4164	6.73	2	1140.6788	2		0		no	4.71	
KLVAASQAALGL	4666	6.62	1	1154.6968	2		0	[1] Methyl K	no	4.64	
KQTALVELVK	2489	7.54	3	1127.6921	2		0		no	4.64	
KVPQVSTPTLVEVSR	357	8.71	4	1638.9299	3		0		no	3.95	
KVPQVSTPTLVEVSR	4300	8.71	3	1638.9249	2		0		no	6.37	
+KVPQVSTPTLVEVSR	6689	7.50	1	1666.9066	2		0	[N-term] Formyl K N-TERM	no	6.51	
LAKTYETTLEK	2905	6.48	2	1295.6945	3		0		no	3.67	
LAKTYETTLEK	10928	---	---	1295.6946	2		0		no	5.26	
LCTVATLR	359	8.69	4	932.5167	2		0	[2] Carbamidomethyl C	no	4.02	
LDELRDEGK	828	8.97	4	1073.5428	3		0		yes	3.25	
LDELRDEGK	344	8.97	6	1073.5392	2		0		yes	4.50	
LKECCEKPLLEK	3386	8.16	1	1545.7960	4		0	[4] Carbamidomethyl C | [5] Carbamidomethyl C	no	3.39	
LKECCEKPLLEK	970	8.29	3	1545.7908	3		0	[4] Carbamidomethyl C | [5] Carbamidomethyl C	no	4.29	
LKECCEKPLLEK	1052	8.16	1	1545.7945	3		0	[4] Carbamidomethyl C | [5] Carbamidomethyl C	no	4.29	
LKECCEKPLLEK	2624	8.29	3	1545.7922	4		0	[4] Carbamidomethyl C | [5] Carbamidomethyl C	no	3.39	
LVAASQAALGL	680	8.53	4	1012.5910	1		0		no	11.43	
LVNEVTEFAK	144	9.23	8	1148.6144	2		0		yes	4.57	1.[illegible]

Sequence	Peptide Ion	Score	Hits	Mass	Charge	Tags	Conflicts	Modifications	In quantitation	Drift time (ms)	Av Nc Ab CT
LVNEVTEFAK	4458	9.17	1	1148.6093	1		0		yes	12.74	
LVNEVTEFAK	4704	7.08	2	1162.6095	2		0	[10] Methyl K	no	4.64	
LVTDLTK	2106	8.87	2	788.4638	1		0		no	9.21	
LVTDLTK	440	8.87	6	788.4649	2		0		no	3.46	
QEPERNECFLQHK	12502	6.94	1	1713.7830	4		0	[8] Carbamidomethyl C	no	3.39	
QEPERNECFLQHK	6826	6.94	1	1713.7809	3		0	[8] Carbamidomethyl C	no	4.09	
QNCELFEQLGEYK	3008	8.34	4	1656.7480	2		0	[3] Carbamidomethyl C	no	6.23	
QTALVELVK	307	8.95	6	999.6009	2		0		no	4.29	
QTALVELVK+	1228	0.00	3	981.5865	2		0	[C-term] Dehydrated	no	4.16	
RPCFSALEVDETYVPK	4210	8.62	4	1909.9163	2		0	[3] Carbamidomethyl C	no	7.27	
RPCFSALEVDETYVPK	368	8.62	4	1909.9272	3		0	[3] Carbamidomethyl C	no	4.85	
SHCIAEVENDEMPADLPSLAADFVESK	8021	7.24	1	2989.3184	3		0	[3] Carbamidomethyl C|[9] Hydroxyl DKNP	no	5.89	
SLHTLFGDKLCTVATLR	1213	6.61	1	1931.0237	3		0	[11] Carbamidomethyl C	no	5.06	
SLHTLFGDKLCTVATLR	792	6.61	1	1931.0192	2		0	[11] Carbamidomethyl C	no	7.34	
TCVADESAENCDK	335	8.95	4	1497.5742	2		0	[2] Carbamidomethyl C|[11] Carbamidomethyl C	no	5.40	
TDLTK	403	0.00	3	576.3128	1		0		no	6.86	
TYETTLEK	5500	9.12	1	983.4820	1		0		no	11.22	
TYETTLEK	380	9.12	4	983.4866	2		0		no	4.16	
VFDEFKPLVEEPQNLIK	487	9.07	5	2044.0930	3		0		no	4.57	
VFDEFKPLVEEPQNLIK	5787	9.07	1	2044.0807	2		0		no	7.69	
VPQVSTPTLVEVSR	405	9.11	4	1510.8366	2		0		no	5.75	
VPQVSTPTLVEVSR	3499	9.11	4	1510.8348	3		0		no	4.02	
VTKCCTESLVNR	6280	6.86	1	1465.7028	3		0	[4] Carbamidomethyl C|[5] Carbamidomethyl C	no	3.67	
YEIAR	829	0.00	4	650.3393	1		0		no	7.83	
YETTLEK	7987	0.00	1	882.4320	1		0		no	10.11	
YICENQDSISSK	616	8.76	6	1442.6362	2		0	[3] Carbamidomethyl C	no	5.54	
YKAAFTECCQAADK	2826	7.93	4	1675.7414	3		0	[2] Methyl K|[8] Carbamidomethyl C|[9] Carbamidomethyl C	no	4.02	
YLYEIAR	4632	8.84	1	926.4846	1		0		no	10.80	

Sequence	Peptide Ion	Score	Hits	Mass	Charge	Tags	Conflicts	Modifications	In quantitation	Drift time (ms)	Av Nc Ab
											CT
YLYEIAR	364	8.84	6	926.4873	2		0		no	4.22	

	Tags
	Anova p-value ≤ 0.05
	power>0.8
	q<0.05

P04264

Keratin_ type II cytoskeletal 1 OS=Homo sapiens OX=9606 GN=KRT1 PE=1 SV=6
74 peptides

Sequence	Peptide Ion	Score	Hits	Mass	Charge	Tags	Conflicts	Modifications	In quantitation
AEAESLYQSK	342	8.97	16	1124.5362	2		0		no
AEAESLYQSKYEELQITAGR	1143	8.42	4	2285.1154	3		0		no
AENEFVTIKK	140	0.00	2	1177.6238	2		0		no
AENEFVTIKK	14999	0.00	2	1177.6471	2		0		no
AQYEDIAQK	63	8.89	15	1064.5212	2		0		yes
AQYEDIAQK	1395	8.46	2	1064.5135	1		0		no
+AQYEDIAQK	1145	0.00	1	1047.4915	2		0	[N-term] Ammonia-loss	no
AQYEDIAQK	2678	5.44	2	1080.4991	2		0	[9] Hydroxyl DKNP	no
DGAYMTK	1820	0.00	3	800.3361	1		0	[7] Hydroxyl DKNP	no
DLDSIIAEVK	627	0.00	4	1101.5915	1		0		no
DLDSIIAEVK	4950	---	---	1101.5934	2		0		no
DVDGAYMTK	406	8.55	15	998.4419	2		0		no
DVDGAYMTK	11583	---	---	998.4413	1		0		no
DVDGAYMTK	542	7.85	1	1014.4365	2		0	[1] Hydroxyl DKNP	no
DVDGAYMTK	412	8.36	15	1014.4357	2		0	[9] Hydroxyl DKNP	no
DYQELMNTK	1129	8.85	3	1140.5234	2		0		no
DYQELMNTK	5118	5.71	1	1156.4997	2		0	[1] Hydroxyl DKNP	no
DYQELMNTK	2629	7.02	3	1156.5086	2		0	[6] Oxidation M	no
DYQELMNTK	1513	7.45	3	1154.5339	2		0	[9] Methyl K	no
EDIAQK	1420	0.00	1	702.3541	1		0		no
EDLAR	1634	7.74	2	602.3037	1		0		no
EELQITAGR	4007	0.00	2	1015.5304	1		0		no
EGEESR	320	0.00	8	705.2939	1		0		no
ELQITAGR	1295	0.00	1	886.4830	1		0		no
ENEFVTIKK	986	0.00	2	1106.5959	2		0		no
FLEQQNQVLQTK	362	9.00	13	1474.7776	2		0		no
FSSCGGGGGSFGAGGGFGSR	3840	9.06	2	1764.7326	3		0		no

Sequence	Peptide Ion	Score	Hits	Mass	Charge	Tags	Conflicts	Modifications	In quantitation
								[4] Carbamidomethyl C	
FSSCGGGGGSFGAGGGFGSR	691	9.06	13	1764.7274	2		0	[4] Carbamidomethyl C	no
FVTIKK	5747	---	---	734.4676	2		0		no
FVTIKK	1646	0.00	2	734.4687	1		0		no
GEESR	510	0.00	1	576.2521	1		0		no
GGGGGGYGSGGSSYGSGGGSYGSGGGGGGGR	2659	7.83	9	2382.9353	3		0		no
GGGGGGYGSGGSSYGSGGGSYGSGGGGGGGR	9499	---	---	2382.9455	2		0		no
IEISELNR	547	8.50	9	972.5230	2		0		no
ISVAR	1204	0.00	2	544.3342	1		0		no
KDVDGAYMTK	3535	7.82	2	1126.5293	2		0		no
LALDLEIATYR	1342	8.46	8	1276.7021	2		0		no
LDSELKNMQDMVEDYR	2772	8.61	2	1984.8879	3		0		no
LDSELKNMQDMVEDYR	4324	6.86	1	2000.8820	3		0	[10] Hydroxyl DKNP	no
LDSELKNMQDMVEDYR	4980	6.57	1	2000.8815	3		0	[10] Hydroxyl DKNP	no
LDSELKNMQDMVEDYR	1150	7.67	4	2016.8764	3		0	[6] Hydroxyl DKNP|[7] Hydroxyl DKNP	no
LDSIIAEVK	2095	0.00	1	986.5649	1		0		no
LEGEESR	170	0.00	10	818.3780	1		0		no
LEGEESR	1425	---	---	818.3790	2		0		no
LLRDYQELMNTK	871	8.78	3	1522.7841	3		0		no
LLRDYQELMNTK	4247	8.78	2	1522.7765	2		0		no
LLRDYQELMNTK	560	7.91	7	1538.7772	3		0	[9] Oxidation M	no
LLRDYQELMNTK	4363	7.91	2	1538.7700	2		0	[9] Oxidation M	no
LLRDYQELMNTK	6155	---	---	1536.7924	2		0	[12] Methyl K	no
LLRDYQELMNTK	633	7.34	1	1536.7955	3		0	[12] Methyl K	no
LNDLEDALQQAKEDLAR	2693	7.75	2	1940.9741	3		0		no
LYQSK	1704	0.00	2	637.3430	1		0		no
LYQSK	5307	---	---	637.3432	2		0		no
MSGECAPNVSVSVSTSHTTISGGGSR	2440	8.02	2	2564.1534	3		0	[5] Carbamidomethyl C	no
MSGECAPNVSVSVSTSHTTISGGGSR	5011	7.29	1	2580.1417	3		0	[1] Oxidation M|[5] Carbamidomethyl C	no
NAENEFVTIKK	1199	0.00	1	1274.6511	2		0		no
NEFVTIKK	3997	0.00	1	977.5534	1		0		no
NEFVTIKK	2018	0.00	2	977.5514	2		0		no
NKLNDLEDALQQAK	2625	7.94	2	1598.8255	3		0		no
NKLNDLEDALQQAK	4186	7.94	2	1598.8213	2		0		no
NLGGSK	293	0.00	3	574.3089	1		0		no
NMQDMVEDYR	773	8.88	11	1299.5248	2		0		no
NMQDMVEDYR	1622	7.74	1	1315.5180	2		0	[4] Hydroxyl DKNP	no
NMQDMVEDYR	1343	6.67	3	1315.5177	2		0	[4] Hydroxyl DKNP	no

Sequence	Peptide Ion	Score	Hits	Mass	Charge	Tags	Conflicts	Modifications	In quantitation
NMQDMVEDYR	1064	7.89	4	1315.5181	2		0	[4] Hydroxyl DKNP	no
QITAGR	1732	0.00	2	644.3594	1		0		no
SDQSRLDSELK	1975	7.51	2	1276.6256	3		0		no
SGGGFSSGSAGIINYQR	3566	---	---	1656.7975	3		0		no
SGGGFSSGSAGIINYQR	1036	8.60	12	1656.7834	2		0		no
SGYRSGGGFSSGSAGIINYQR	1377	6.11	1	2120.0011	3		0		no
SISISVAR	1400	8.74	2	831.4816	1		0		no
SISISVAR	278	8.74	12	831.4828	2		0		no
SISVAR	4487	---	---	631.3642	2		0		no
SISVAR	169	0.00	8	631.3666	1		0		no
SKAEAE	1267	0.00	2	615.2870	1		0		no
SKAEAE	2368	---	---	615.2856	2		0		no
SKAEAES	887	0.00	1	702.3186	1		0		no
SKAEAES	2695	---	---	702.3183	2		0		no
SKAEAES	3205	0.00	2	684.3074	1		0		no
SKAEAES	9789	---	---	684.3069	2		0		no
SKAEAESL	4086	0.00	1	815.4013	1		0		no
SKAEAESL	1852	---	---	815.4088	2		0		no
SKAEAESLYQSK	281	8.66	16	1339.6622	2		0		no
SKAEAESLYQSK	274	8.66	16	1339.6630	3		0		no
SKAEAESLYQSK+	585	0.00	7	1321.6503	3		0	[C-term] Dehydrated	no
SLDLDSIIAEVK	3986	8.13	1	1301.7055	3		0		no
SLDLDSIIAEVK	9900	---	---	1301.7132	1		0		no
SLDLDSIIAEVK	32	8.70	15	1301.7150	2		0		no
SLDLDSIIAEVKAQYEDIAQK	1958	7.95	4	2348.2035	3		0		no
SLNNQFASFIDK	3027	8.51	4	1382.6751	2		0		no
SLVNLGGSK	83	8.61	15	873.4962	2		0		no
SLVNLGGSK	788	8.61	3	873.4914	1		0		no
SLYQSK	1119	0.00	2	724.3755	1		0		no
SLYQSK	2286	0.00	2	724.3748	2		0		no
TLLEGEESR	39	8.66	14	1032.5181	2		0		yes
TLLEGEESR	880	8.72	3	1032.5093	1		0		yes
TLLEGEESR+	1698	0.00	1	1014.5078	2		0	[C-term] Dehydrated	no
TNAENE	8479	---	---	658.2558	2		0		no
TNAENE	930	0.00	2	658.2563	1		0		no
TNAENEF	1804	0.00	2	805.3236	1		0		no
TNAENEFVTIK	106	9.15	16	1264.6349	2		0		no
TNAENEFVTIKK	42	9.18	15	1392.7412	3		0		yes
TNAENEFVTIKK	137	9.18	15	1392.7280	2		0		yes
VTIKK	6780	---	---	587.3987	2		0		no
VTIKK	810	0.00	2	587.4010	1		0		no
WELLQQVDTSTR	1164	8.92	4	1474.7384	2		0		no
YEDEINKR	1658	8.40	2	1065.5082	3		0		no
YEDEINKR	1238	8.40	3	1065.5093	2		0		no

Sequence	Peptide Ion	Score	Hits	Mass	Charge	Tags	Conflicts	Modifications	In quantitation
YEELQITAGR	80	9.30	16	1178.5996	2		0		no
YEELQITAGR	3360	9.30	2	1178.5917	1		0		no

Tags	
	Anova p-value ≤ 0.05
	power>0.8
	q<0.05

P08670

Vimentin OS=Homo sapiens OX=9606 GN=VIM PE=1 SV=4
56 peptides

Sequence	Peptide Ion	Score	Hits	Mass	Charge	Tags	Conflicts	Modifications	In quantitation	Drift time (ms)	A A
DGQVINETSQHHDDLE	3243	7.74	3	1835.7923	3		0		no	4.02	
EEAENTLQSFR	3990	8.53	2	1322.6119	2		0		no	5.06	
EGEESR	2140	0.00	1	705.2932	1		0		no	7.48	
EKLQEEMLQR	4046	5.61	1	1302.6467	2		0		no	5.06	
EMEENFAVEAANYQDTIGR	6152	8.31	2	2201.9489	2		0	[5] Hydroxyl DKNP	no	7.83	
EMEENFAVEAANYQDTIGR	3295	8.31	2	2201.9503	3		0	[5] Hydroxyl DKNP	no	5.61	
EQQNK	3700	0.00	1	645.3088	1		0		no	7.13	
ETNLDSLPLVDTHSKR	7945	7.67	1	1823.9317	4		0		no	3.60	
ETNLDSLPLVDTHSKR	3034	7.68	2	1823.9327	3		0		no	4.50	
EYQDLLNVK	474	8.58	8	1120.5767	2		0		no	4.71	
FADLSEAANR	312	8.77	12	1092.5262	2		0		no	4.36	
FADLSEAANRNNDALR	8837	8.62	2	1775.8500	2		0		no	7.20	
FANYIDK	1277	8.40	2	869.4272	2		0		no	3.67	
FANYIDKVR	2357	8.36	2	1124.5998	3		0		no	3.39	
FLEQQNK	581	8.60	9	905.4622	2		0		no	3.88	
FLEQQNK	8474	7.93	1	905.4548	1		0		no	10.25	
ILLAELEQLK	2288	8.23	1	1168.7088	2		0		no	5.06	
ILLAELEQLK	2884	8.16	1	1168.7072	2		0		no	5.06	
ILLAELEQLKGQGK	630	8.54	7	1538.9077	3		0		no	4.09	
ILLAELEQLKGQGK	2961	8.54	2	1538.8999	2		0		no	6.51	
ISLPLPNFSSLNLR	4451	8.08	2	1569.8856	2		0		no	6.09	
ISLPLPNFSSLNLRETNLDSLPLVDTHSK	2345	5.91	1	3235.6931	3		0	[29] Hydroxyl DKNP	yes	8.17	
ISLPLPNFSSLNLRETNLDSLPLVDTHSK	319	5.91	1	3235.7105	4		0	[29] Hydroxyl DKNP	yes	5.61	1
ISLPLPNFSSLNLRETNLDSLPLVDTHSK	6574	---	---	3235.7039	5		0	[29] Hydroxyl DKNP	yes	5.19	
KLLEGEESR	972	8.22	3	1059.5530	2		0		no	4.71	
KVESLQEEIAFLK	1406	9.12	4	1532.8450	2		0		no	6.16	
KVESLQEEIAFLK	1158	9.12	5	1532.8503	3		0		no	4.02	

Sequence	Peptide Ion	Score	Hits	Mass	Charge	Tags	Conflicts	Modifications	In quantitation	Drift time (ms)	A A
LDLER	1636	8.39	1	644.3495	1		0		no	7.83	
LDLER	1951	8.39	1	644.3507	2		0		no	3.19	
LEGEESR	5849	0.00	1	818.3757	1		0		no	9.07	
LEQQNK	6577	0.00	1	758.3927	1		0		no	8.52	
LGDLYEEEMR	1781	8.11	3	1269.5613	2		0	[9] Oxidation M	no	4.92	
LGDLYEEEMRELR	2412	6.75	1	1683.7954	3		0	[3] Hydroxyl DKNP|[9] Oxidation M	no	4.16	
LLEGEESR	11149	---	---	931.4581	1		0		no	10.32	
LLEGEESR	535	8.62	10	931.4629	2		0		no	3.95	
LQDEIQNMKEEMAR	1056	7.81	2	1765.7994	3		0	[9] Hydroxyl DKNP|[12] Oxidation M	no	4.22	
LQDEIQNMKEEMAR	2024	7.71	2	1781.7925	3		0	[7] Hydroxyl DKNP|[8] Oxidation M|[9] Hydroxyl DKNP	no	4.22	
LQEEMLQREEAENTLQSFR	4856	7.15	2	2366.1144	3		0	[13] Hydroxyl DKNP	no	4.92	
MALDIEIATYR	2416	8.36	2	1310.6543	2		0	[1] Oxidation M	no	5.12	
MALDIEIATYRK	2461	7.72	1	1438.7487	3		0	[1] Oxidation M	no	4.02	
MFGGPGTASRPSSSR	3039	7.17	2	1509.7003	3		0	[1] Oxidation M	no	4.16	
NLQEAEEWYK	758	9.03	11	1308.6022	2		0		no	5.06	
QDVDNASLAR	466	8.71	12	1087.5290	2		0		no	4.29	
QMREMEENFAVEAANYQDTIGR	1860	6.94	2	2633.1502	3		0	[2] Oxidation M|[15] Hydroxyl DKNP	no	5.33	
QQYESVAAK	5094	8.94	1	1022.5018	1		0		no	12.19	
QQYESVAAK	431	8.94	9	1022.5071	2		0		no	4.50	
+QQYESVAAK	1823	0.00	1	1005.4851	2		0	[N-term] Ammonia-loss	no	4.36	
QQYESVAAKNLQEAEEWYK	452	5.77	1	2313.0812	4		0		yes	4.57	
QQYESVAAKNLQEAEEWYK	2217	5.85	1	2329.0963	3		0	[10] Hydroxyl DKNP	no	5.19	
QQYESVAAKNLQEAEEWYK	5018	5.85	1	2329.0927	2		0	[10] Hydroxyl DKNP	no	8.59	
QVDQLTNDK	950	8.32	4	1059.5216	2		0		no	4.29	
QVDQLTNDKAR	4480	8.13	2	1286.6586	2		0		no	5.19	
QVDQLTNDKAR	881	8.13	8	1286.6603	3		0		no	3.67	
QVQSLTCEVDALKGTNESLER	2594	8.12	3	2376.1529	3		0	[7] Carbamidomethyl C	no	6.02	
RQVDQLTNDK	2132	8.16	2	1215.6199	2		0		no	4.78	
RQVDQLTNDK	678	5.36	1	1229.6370	2		0	[10] Methyl K	no	5.06	
SEAANR	4108	0.00	1	646.3057	1		0		no	7.06	
SKFADLSEAANR	6241	---	---	1307.6449	2		0		yes	5.12	
SKFADLSEAANR	528	5.10	2	1307.6495	3		0		yes	3.53	2
SSVPGVR	2813	7.94	1	700.3859	1		0		no	7.96	
SSVPGVR	3685	7.94	1	700.3867	2		0		no	3.25	
SYVTTSTR	5979	4.52	1	913.4665	2		0		no	3.81	
SYVTTSTR	2156	8.81	2	913.4511	1		0		no	10.25	
SYVTTSTR	481	8.81	7	913.4532	2		0		no	3.95	
TLLIK	827	6.95	1	586.4065	1		0		no	7.41	

Sequence	Peptide Ion	Score	Hits	Mass	Charge	Tags	Conflicts	Modifications	In quantitation	Drift time (ms)
TNEKVELQELNDR	5858	8.73	2	1586.7874	2		0		no	6.16
TNEKVELQELNDR	769	8.73	7	1586.7924	3		0		no	3.95
TTSTR	1112	0.00	1	564.2878	1		0		no	6.44
TYSLGSALRPSTSR	7326	7.88	1	1494.7779	2		0		no	6.30
VERDNLAEDIMR	3414	0.00	1	1475.7023	2		0	[11] Oxidation M	no	5.82
VEVERDNLAEDIMR	6098	6.90	1	1687.8201	3		0		no	3.95
VEVERDNLAEDIMR	292	6.35	8	1703.8209	3		0	[11] Hydroxyl DKNP	no	3.88
VEVERDNLAEDIMR	2800	8.46	2	1703.8131	2		0	[13] Oxidation M	no	6.44
VEVERDNLAEDIMR	12727	7.26	1	1719.8086	2		0	[6] Hydroxyl DKNP|[11] Hydroxyl DKNP	no	6.58

Tags	
	Anova p-value ≤ 0.05
	power>0.8
	q<0.05

P00330

Alcohol dehydrogenase 1 OS=Saccharomyces cerevisiae (strain ATCC 204508 / S288c) OX=559292 GN=ADH1 PE=1 SV=5
91 peptides

Sequence	Peptide Ion	Score	Hits	Mass	Charge	Tags	Conflicts	Modifications	In quantitation	Drift time (ms)
ALDFFAR	174	0.00	7	838.4341	1		0		no	9.56
ALDFFAR	667	0.00	6	838.4341	2		0		no	3.81
ANELL	334	0.00	12	540.2922	1		0		no	6.44
ANELLI	436	0.00	1	653.3757	1		0		no	7.83
ANELLINVK	91	9.37	13	1012.5921	1		0		no	11.91
+ANELLINVK	101	0.00	8	995.5700	2		0	[N-term] Ammonia-loss	no	4.09
ANGTTVLVGMPAGAK	128	8.93	16	1385.7345	2		0		no	5.47
ANGTTVLVGMPAGAK	343	7.76	6	1401.7270	2		0	[11] Hydroxyl DKNP	no	5.54
ANGTTVLVGMPAGAK	363	7.58	10	1401.7270	2		0	[11] Hydroxyl DKNP	no	5.61
ATDGGAHGVINVSVSEA	2992	0.00	1	1564.7427	2		0		no	5.75
ATDGGAHGVINVSVSEAA	3138	0.00	2	1635.7830	2		0		no	5.96
ATDGGAHGVINVSVSEAAIEASTR	2889	---	---	2311.1383	4		0		no	4.78
ATDGGAHGVINVSVSEAAIEASTR	925	9.24	7	2311.1385	2		0		no	8.66
ATDGGAHGVINVSVSEAAIEASTR	38	9.24	16	2311.1475	3		0		no	6.23
ATDGGAHGVINVSVSEAAIEASTR+	938	0.00	4	2293.1257	3		0	[C-term] Dehydrated	no	6.02
ATDGGAHGVINVSVSEAAIEASTRYVR	2708	7.90	3	2729.3648	3		0		no	7.20
ATDGGAHGVINVSVSEAAIEASTRYVR	355	7.90	10	2729.3706	4		0		no	5.33
CCSDVFNQVVK	8	9.52	16	1354.6131	2		0	[1] Carbamidomethyl	yes	5.33

Sequence	Peptide Ion	Score	Hits	Mass	Charge	Tags	Conflicts	Modifications	In quantitation	Drift time (ms)
								C|[2] Carbamidomethyl C		
CCSDVFNQVVK	4356	---	---	1354.6067	1		0	[1] Carbamidomethyl C|[2] Carbamidomethyl C	yes	13.57
CCSDVFNQVVK	955	7.32	5	1370.5970	2		0	[1] Carbamidomethyl C|[2] Carbamidomethyl C|[4] Hydroxyl DKNP	no	5.19
CCSDVFNQVVKSISIVGSYVGNR	2578	7.81	8	2587.2392	3		0	[1] Carbamidomethyl C|[2] Carbamidomethyl C	no	6.79
CCSDVFNQVVKSISIVGSYVGNR	1555	7.81	8	2587.2398	3		0	[1] Carbamidomethyl C|[2] Carbamidomethyl C	no	5.54
DFFAR	3296	0.00	6	654.3155	1		0		no	4.16
DFFAR	26	0.00	6	654.3154	1		0		no	7.62
DGGEGK	233	0.00	9	561.2410	1		0		no	6.37
DGGEGKEELFR	252	0.00	6	1235.5804	2		0		no	5.33
DGGEGKEELFR	1139	0.00	4	1235.5776	1		0		no	12.60
DIPVPKPK	27	8.49	11	892.5482	2		0		no	3.95
DIPVPKPK	1960	---	---	892.5343	1		0		no	10.11
DIVGAVLK	513	0.00	11	813.4951	1		0		no	9.21
DIVGAVLK	45	9.36	16	813.5057	2		0		no	3.67
DIVGAVLK	18	9.36	16	813.4985	1		0		no	9.14
DIVGAVLK	13196	8.54	3	813.4979	1		0		no	1.80
DYAGIK	8013	---	---	665.3358	2		0		no	3.12
DYAGIK	49	0.00	9	665.3397	1		0		no	7.89
EALDF	221	0.00	3	557.2501	1		0		no	6.44
EALDFFAR	92	9.34	7	967.4768	1		0		no	10.73
EALDFFAR	7	9.34	5	967.4906	2		0		yes	4.16
EALDFFAR+	36	0.00	5	949.4707	2		0	[C-term] Dehydrated	no	4.02
EELFR	151	8.75	13	692.3501	1		0		no	7.96
EELFR	230	8.75	13	692.3529	2		0		no	3.53
EELFR+	550	0.00	9	674.3395	2		0	[C-term] Dehydrated	no	3.19
EKDIV	583	0.00	6	584.3175	1		0		no	6.72
EKDIVGAVLK	20	9.31	16	1070.6453	2		0		no	4.50
EKDIVGAVLK	397	9.31	16	1070.6373	3		0		no	3.46
EKDIVGAVLK	3946	---	---	1070.6341	1		0		no	12.33
EKDIVGAVLK+	1328	---	---	1052.6224	3		0	[C-term] Dehydrated	no	3.19
ESHGK	288	0.00	9	556.2628	1		0		no	6.37
ESHGKLEYK	2079	0.00	1	1089.5507	2		0		no	4.50
FYESHGK	102	0.00	1	866.3924	1		0		no	9.70
FYESHGKLEYK	389	0.00	7	1399.6786	2		0		no	5.61

Sequence	Peptide Ion	Score	Hits	Mass	Charge	Tags	Conflicts	Modifications	In quantitation	Drift time (ms)
FYESHGKLEYK	373	0.00	7	1399.6792	3		0		no	3.74
GAVLK	51	0.00	15	486.3185	1		0		no	6.16
GAVLK	37	0.00	13	486.3189	1		0		no	6.09
GAVLK	2065	---	---	486.3171	2		0		no	2.63
GDYAGIK	2	0.00	2	722.3644	1		0		no	8.38
GDYAGIK	688	0.00	1	738.3529	1		0	[2] Hydroxyl DKNP	no	8.45
GGEGKEELFR	1006	0.00	6	1120.5533	2		0		no	4.92
GGEGKEELFR	2375	---	---	1120.5519	1		0		no	11.77
GGEVFIDFTK	1000	0.00	10	1111.5545	1		0		no	12.47
GIDGGEGK	25	0.00	4	731.3476	1		0		no	8.10
GIDGGEGKEELFR	33	0.00	2	1405.6923	2		0		no	6.23
GIDGGEGKEELFR	2963	0.00	2	1405.6894	2		0		no	4.36
GIDGGEGKEELFR	8681	---	---	1405.6813	3		0		no	3.81
GSYVGNR	54	0.00	5	751.3629	1		0		no	8.31
GSYVGNR	14842	0.00	5	751.3631	1		0		no	0.97
GSYVGNR	7294	0.00	5	751.3634	1		0		no	1.73
GVIFYESHGK	10759	---	---	1151.5560	3		0	[10] Hydroxyl DKNP	no	3.12
GVIFYESHGK	1773	6.96	1	1151.5581	2		0	[10] Hydroxyl DKNP	no	4.64
GVIFYESHGKLEYK	95	8.87	16	1668.8592	3		0		no	4.16
GVIFYESHGKLEYK	1839	---	---	1668.8418	2		0		no	6.58
IDGGEGKEELFR	721	0.00	6	1348.6631	2		0		no	5.96
IFYESHGKLEYK	193	0.00	8	1512.7670	3		0		no	3.95
IGDYAGI	693	0.00	1	689.3387	1		0		no	7.48
IGDYAGIK	15	9.28	2	835.4558	2		0		no	3.67
IGDYAGIK	35	9.62	15	835.4451	1		0		no	9.56
IGDYAGIK	239	6.73	2	851.4364	2		0	[8] Hydroxyl DKNP	no	3.88
IGDYAGIK	597	6.89	1	867.4313	2		0	[3] Hydroxyl DKNP | [8] Hydroxyl DKNP	no	3.95
IVGAVLK	1468	---	---	698.4688	1		0		no	7.96
IVGAVLK	738	0.00	2	698.4686	2		0		no	3.46
IVGSYVGNR	480	0.00	2	963.5125	1		0		no	10.87
KDIVGAVLK	1350	0.00	2	941.5900	2		0		no	4.02
LDFFAR	236	0.00	5	767.3980	2		0		no	3.53
LDFFAR	8096	0.00	3	767.3855	2		0		no	4.16
LDFFAR	34	0.00	5	767.3983	1		0		no	9.07
LEYKDIPVPKPK	2089	7.48	3	1425.8146	3		0		no	3.81
LGIDGGEGK	483	0.00	2	844.4292	2		0		no	3.53
LGIDGGEGK	116	0.00	2	844.4293	1		0		no	9.07
LGIDGGEGKEELFR	99	0.00	4	1518.7700	2		0		no	6.58
LGIDGGEGKEELFR	651	0.00	4	1518.7715	3		0		no	4.02
LINVK	68	0.00	2	585.3871	1		0		no	7.20
LLINVK	74	0.00	1	698.4702	1		0		no	8.52
LPLVGGHEGAGVVVGMGENVK	7458	---	---	2018.0516	2		0		no	8.31
LPLVGGHEGAGVVVGMGENVK	173	8.85	16	2018.0655	3		0		no	4.64

Sequence	Peptide Ion	Score	Hits	Mass	Charge	Tags	Conflicts	Modifications	In quantitation	Drift time (ms)
LPLVGGHEGAGVVVGMGENVK	476	7.80	8	2034.0556	3		0	[16] Oxidation M	no	4.64
LPLVGGHEGAGVVVGMGENVK	603	6.47	1	2034.0552	3		0	[16] Oxidation M	no	4.57
LPLVGGHEGAGVVVGMGENVK	559	7.56	4	2034.0553	3		0	[19] Hydroxyl DKNP	no	4.64
LPLVGGHEGAGVVVGMGENVK	1137	7.56	3	2034.0566	3		0	[19] Hydroxyl DKNP	no	5.75
LPLVGGHEGAGVVVGMGENVK	1611	6.84	1	2034.0535	3		0	[19] Hydroxyl DKNP	no	4.64
LPLVGGHEGAGVVVGMGENVK	610	7.10	3	2032.0785	4		0	[21] Methyl K	no	4.64
LPLVGGHEGAGVVVGMGENVK	582	7.10	3	2032.0744	3		0	[21] Methyl K	no	5.47
NELLINVK	272	0.00	1	941.5557	2		0		no	4.02
NELLINVK	423	---	---	941.5528	1		0		no	11.08
SANLMAGHWVAISGAAGGLGSLAVQYAK	849	8.28	10	2699.3750	3		0		no	7.83
SANLMAGHWVAISGAAGGLGSLAVQYAK	2116	6.30	1	2715.3644	3		0	[3] Hydroxyl DKNP	no	7.83
SDVFNQVVK	671	0.00	13	1034.5386	1		0		no	11.29
SEAAIEASTR	2103	0.00	1	1033.5045	1		0		no	11.50
SIGGEVFIDFTK	47	9.64	16	1311.6775	2		0		no	5.19
SIGGEVFIDFTKEK	3804	---	---	1568.8063	2		0		no	6.72
SIGGEVFIDFTKEK	820	7.94	9	1568.8093	3		0		no	4.29
SISIV	322	0.00	7	499.2998	1		0		no	6.16
SISIVGSYVGNR	3	9.32	9	1250.6738	2		0		yes	5.06
SISIVGSYVGNR	660	9.18	7	1250.6614	1		0		no	13.43
SISIVGSYVGNR	1186	7.16	2	1266.6509	2		0	[11] Hydroxyl DKNP	no	4.92
SISIVGSYVGNR+	600	0.00	5	1232.6479	2		0	[C-term] Dehydrated	no	4.85
SISIVGSYVGNRADTR	2166	7.02	5	1693.8598	3		0		no	4.43
SIVGSYVGNR	514	0.00	1	1050.5453	2		0		no	4.29
SIVGSYVGNR	112	0.00	2	1050.5462	1		0		no	11.77
SPIKVVGLSTLPEIYEK	772	6.72	13	1872.0561	3		0		no	4.43
SYVGNR	444	0.00	5	694.3410	1		0		no	7.83
VDTSK	71	0.00	7	548.2828	1		0		no	6.37
VGAVLK	759	---	---	585.3850	2		0		no	3.05
VGAVLK	569	0.00	8	585.3851	1		0		no	7.13
VGAVLK	9	0.00	9	585.3899	1		0		no	7.06
VGAVLK	2092	---	---	585.3804	2		0		no	2.98
VGSYVGNR	87	0.00	3	850.4309	1		0		no	9.49
VLGIDGGEGK	19	9.30	16	943.5098	2		0		no	3.95
VLGIDGGEGK	271	9.30	13	943.4965	1		0		no	10.11
VLGIDGGEGKEELFR	53	9.42	16	1617.8403	2		0		no	7.06
VLGIDGGEGKEELFR	12712	9.42	16	1617.8042	2		0		no	6.02
VPKPK	206	0.00	8	567.3756	1		0		no	6.79
VVDTSK	147	0.00	9	647.3499	1		0		no	7.55
VVGLSTLPEIYEK	1788	7.42	7	1462.7854	2		0	[8] Hydroxyl DKNP	no	5.75
VVGLSTLPEIYEK	531	7.55	2	1462.7861	2		0	[13] Hydroxyl DKNP	no	6.02
YAGIK	287	0.00	9	550.3127	1		0		no	6.58
YESHGK	653	0.00	4	719.3259	2		0		no	3.25

Sequence	Peptide Ion	Score	Hits	Mass	Charge	Tags	Conflicts	Modifications	In quantitation	Drift time (ms)
YESHGKLEYK	2145	---	---	1252.6060	3		0		no	3.46
YESHGKLEYK	493	0.00	5	1252.6098	2		0		no	4.99
YVGNR	754	0.00	1	607.3087	1		0		no	7.13
YVVDTSK	89	9.33	16	810.4202	2		0		no	3.60
YVVDTSK	156	9.33	11	810.4129	1		0		no	9.07
YVVDTSK	249	8.37	5	810.4126	1		0		no	9.07

Tags	
	Anova p-value ≤ 0.05
	power>0.8
	q<0.05

P35908

Keratin_ type II cytoskeletal 2 epidermal OS=Homo sapiens OX=9606 GN=KRT2 PE=1 SV=2
45 peptides

Sequence	Peptide Ion	Score	Hits	Mass	Charge	Tags	Conflicts	Modifications	In quantitati
AENDFVTLKK	323	0.00	6	1163.6207	2		0		no
AQYEEIAQR	133	8.51	15	1106.5413	2		0		no
AQYEEIAQR	6344	---	---	1106.5361	1		0		no
DNAYMIK	3292	0.00	2	853.4003	1		0		no
DPEIQNVK	3000	0.00	1	941.4849	2		0		no
DVDNAYMIK	520	8.68	9	1067.4984	2		0		no
DVDNAYMIK	2020	7.67	1	1083.4935	2		0	[4] Hydroxyl DKNP	no
DVDNAYMIK	683	8.46	6	1083.4932	2		0	[9] Hydroxyl DKNP	no
DVDNAYMIK	933	7.44	1	1083.4940	2		0	[9] Hydroxyl DKNP	no
GFSSGSAVVSGGSR	629	8.99	10	1253.5999	2		0		no
GGGFGGGSSFGGGSGFSGGGFGGGGFGGGR	3812	7.91	2	2398.0071	3		0		no
GGGFGGGSSFGGGSGFSGGGFGGGGFGGGR	13962	---	---	2398.0157	2		0		no
GGGGFGSR	6580	---	---	693.3218	2		0		no
GGGGFGSR	3527	0.00	2	693.3200	1		0		no
GGSISGGGYGSGGGK	564	9.07	12	1196.5434	2		0		no
GLGGTK	544	0.00	3	531.3019	1		0		no
GSSSGGGYSSGSSSYGSGGR	3083	8.20	2	1739.6935	2		0		no
HGGGGGGFGGGGFGSR	3967	8.63	2	1319.5763	2		0		no
HGGGGGGFGGGGFGSR	1979	8.63	2	1319.5749	3		0		no
HGGGGGGFGGGGFGSR+	6130	0.00	1	1301.5685	3		0	[C-term] Dehydrated	no
KLLEGEECR	870	8.44	3	1132.5573	2		0	[8] Carbamidomethyl C	no
LLEGEECR	10169	---	---	1004.4545	1		0		no

Sequence	Peptide Ion	Score	Hits	Mass	Charge	Tags	Conflicts	Modifications	In quantitati
								[7] Carbamidomethyl C	
LLEGEECR	317	8.54	16	1004.4646	2		0	[7] Carbamidomethyl C	no
LLRDYQELMNVK	4450	8.75	2	1520.7901	2		0		no
LLRDYQELMNVK	912	8.75	3	1520.8043	3		0		no
LLRDYQELMNVK	3040	7.07	1	1536.7959	3		0	[10] Hydroxyl DKNP	no
LNDLEEALQQAK	3632	8.08	2	1370.6940	2		0		no
LNDLEEALQQAKEDLAR	6043	7.61	1	1954.9998	3		0		no
LQGEIAHVK	3022	7.68	2	993.5589	2		0		no
LQGEIAHVK	8489	---	---	993.5611	3		0		no
NKLNDLEEALQQAK	3172	8.23	2	1612.8448	3		0		no
NKLNDLEEALQQAK	4191	8.23	2	1612.8381	2		0		no
NQEIEFLK	4636	0.00	1	1019.5275	1		0		no
NVQDAIADAEQR	863	8.84	9	1328.6307	2		0		no
NVQDAIADAEQRGEHALK	1725	7.82	2	1963.9759	4		0		no
NVQDAIADAEQRGEHALK	1504	7.82	2	1963.9749	3		0		no
PEIQNVK	1105	0.00	1	826.4538	1		0		no
PEIQNVK	473	0.00	1	826.4462	2		0		no
SKEEAEALYHSK	700	8.61	14	1390.6755	3		0		no
SKEEAEALYHSK	13299	---	---	1390.6695	2		0		no
SLVGLGGTK	280	8.65	16	830.4875	2		0		no
SLVGLGGTK	1919	8.65	2	830.4852	1		0		no
SSFSCLSR	2808	0.00	2	942.4219	1		0	[5] Carbamidomethyl C	no
STSSFSCLSR	276	8.68	15	1130.5099	2		0	[7] Carbamidomethyl C	no
STSSFSCLSR	8803	---	---	1130.5063	1		0	[7] Carbamidomethyl C	no
TAAENDFVTLK	330	8.98	16	1207.6109	2		0		no
TAAENDFVTLKK	186	9.04	16	1335.7115	3		0		yes
TAAENDFVTLKK	422	9.04	16	1335.7052	2		0		yes
TAAENDFVTLKK+	1453	0.00	2	1317.6882	3		0	[C-term] Dehydrated	no
TSQNSELNNMQDLVEDYKK	404	9.06	11	2255.0415	3		0		no
TSQNSELNNMQDLVEDYKK	842	7.60	3	2271.0297	3		0	[4] Hydroxyl DKNP	no
TSQNSELNNMQDLVEDYKK	4562	7.31	1	2271.0257	3		0	[8] Hydroxyl DKNP	no
VDLLNQEIEFLK	4609	8.89	2	1459.7946	3		0		yes
VDLLNQEIEFLK	111	8.89	16	1459.7962	2		0		yes
VDLLNQEIEFLKVLYDAEISQIHQSVTDTNVILSMDNSR	1043	---	---	4519.2616	4		0	[12] Hydroxyl DKNP|[12] Methyl K	yes
VDLLNQEIEFLKVLYDAEISQIHQSVTDTNVILSMDNSR	232	6.21	1	4519.2507	5		0	[12] Hydroxyl DKNP|[12] Methyl K	yes
VDPEIQNVK	187	8.80	16	1040.5524	2		0		no
VELQSKVDLLNQEIEFLK	3488	7.87	1	2144.1721	3		0		no

Sequence	Peptide Ion	Score	Hits	Mass	Charge	Tags	Conflicts	Modifications	In quantitati
VLYDAEISQIHQSVTDTNVILSMDNSR	2747	8.23	2	3047.4785	3		0		no
VLYDAEISQIHQSVTDTNVILSMDNSR	8444	8.23	1	3047.4724	4		0		no
VLYDAEISQIHQSVTDTNVILSMDNSR	2756	7.74	2	3063.4650	4		0	[24] Hydroxyl DKNP	no
VLYDAEISQIHQSVTDTNVILSMDNSR	1692	7.74	2	3063.4745	3		0	[24] Hydroxyl DKNP	no
YEELQVTVGR	730	9.11	5	1192.6003	2		0		no
YEELQVTVGR	923	4.84	1	1192.5881	2		0		no
YEELQVTVGR	1678	5.85	1	1192.5858	3		0		no
YLDGLTAER	516	8.59	9	1036.5214	2		0		no
YLDGLTAER	781	5.09	5	1036.5081	2		0		no

Tags	
	Anova p-value ≤ 0.05
	power>0.8
	q<0.05

P63261

Actin_ cytoplasmic 2 OS=Homo sapiens OX=9606 GN=ACTG1 PE=1 SV=1
46 peptides

Sequence	Peptide Ion	Score	Hits	Mass	Charge	Tags	Conflicts	Modifications	In quantitation	Drift time (ms)
AGFAGDDAPR	175	9.00	16	975.4459	2		0		yes	4.09
ALAPSTMK	4121	0.00	1	833.4305	1		0	[8] Hydroxyl DKNP	no	9.28
APEEHPVLLTEAPLNPK	7383	0.00	1	1853.9755	2		0		no	7.34
APPER	4412	---	---	568.2976	2		0		no	2.91
APPER	235	0.00	8	568.2984	1		0		no	6.37
APSTMK	2277	0.00	1	649.3116	1		0	[6] Hydroxyl DKNP	no	7.48
AVFPSIVGRPR	1742	8.35	2	1197.6975	2		0		no	5.19
AVFPSIVGRPR	1717	8.35	2	1197.6988	3		0		no	3.67
CDVDIR	2185	8.16	2	776.3481	2		0	[1] Carbamidomethyl C	no	3.67
DLTDYLMK	733	8.19	4	1013.4763	2		0	[8] Hydroxyl DKNP	no	4.16
DLTDYLMK	13980	---	---	1013.4846	1		0	[8] Hydroxyl DKNP	no	11.43
DLTDYLMK	5264	7.53	1	1029.4747	2		0	[7] Oxidation M | [8] Hydroxyl DKNP	no	4.22
DLYANTVLSGGTTMYPGIADR	1286	---	---	2230.0519	2		0	[20] Hydroxyl DKNP	no	8.24
DLYANTVLSGGTTMYPGIADR	796	6.12	1	2230.0562	3		0	[20] Hydroxyl DKNP	no	4.99
DSYVGDEAQSK	837	8.72	8	1197.5176	2		0		no	4.64
DSYVGDEAQSKR	945	9.05	6	1353.6158	2		0		no	5.61
DSYVGDEAQSKR	822	9.05	8	1353.6216	3		0		no	3.60

Sequence	Peptide Ion	Score	Hits	Mass	Charge	Tags	Conflicts	Modifications	In quantitation	Drift time (ms)
DSYVGDEAQSKR	3434	7.78	3	1367.6311	3		0	[11] Methyl K	no	3.60
DSYVGDEAQSKR	6535	7.62	1	1367.6302	2		0	[11] Methyl K	no	5.61
EITALAPSTMK	3219	7.69	1	1160.5989	2		0		no	4.71
EITALAPSTMK	5680	8.70	1	1176.6077	1		0	[11] Hydroxyl DKNP	no	13.30
EITALAPSTMK	3402	7.17	1	1190.6032	2		0	[7] Hydroxyl DKNP|[11] Methyl K	no	4.71
EITALAPSTMK	594	8.49	3	1192.5928	2		0	[10] Oxidation M|[11] Hydroxyl DKNP	no	4.85
EITALAPSTMKIK	5370	7.16	1	1417.7800	2		0	[11] Hydroxyl DKNP	no	6.09
EITALAPSTMKIK	2599	7.16	1	1417.7816	3		0	[11] Hydroxyl DKNP	no	4.09
EITALAPSTMKIK+	6390	0.00	1	1399.7566	3		0	[11] Hydroxyl DKNP|[C-term] Dehydrated	no	3.88
EIVRDIK	1848	7.59	3	871.5098	2		0		no	3.81
FTTTAER	4213	0.00	1	824.4010	1		0		no	9.42
GILTLK	3899	8.29	1	643.4275	2		0		no	3.12
GILTLK	429	8.46	5	643.4278	1		0		no	7.89
GYSFTTTAER	210	8.98	16	1131.5243	2		0		no	4.57
GYSFTTTAER	4140	8.98	2	1131.5171	1		0		no	12.47
GYSFTTTAEREIVR	1544	8.22	3	1628.8181	3		0		no	4.09
GYSFTTTAEREIVRDIK	2562	6.08	1	1985.0330	2		0		no	7.69
GYSFTTTAEREIVRDIK	2003	6.06	1	1985.0377	3		0		no	5.06
GYSFTTTAEREIVRDIK	2393	6.06	1	1985.0298	2		0		no	7.55
IAPPER	5139	---	---	681.3814	2		0		no	3.25
IAPPER	814	0.00	2	681.3805	1		0		no	7.69
IIAPPER	7598	---	---	794.4651	1		0		no	9.49
IIAPPER	348	8.85	11	794.4660	2		0		no	3.67
IIAPPERK	1702	8.20	3	922.5589	2		0		no	3.88
IKIIAPPER	2257	7.83	2	1035.6449	2		0		no	4.29
IKIIAPPER	11867	---	---	1035.6405	3		0		no	3.12
KDLYANTVLSGGTTMYPGIADR	1824	5.78	1	2358.1409	3		0	[17] Hydroxyl DKNP	no	4.71
KDLYANTVLSGGTTMYPGIADR	6379	5.78	1	2358.1378	2		0	[17] Hydroxyl DKNP	no	7.96
LAPSTMK	3542	0.00	1	762.3959	1		0	[7] Hydroxyl DKNP	no	8.59
LCYVALDFEQEMATAASSSSLEK	5041	7.29	1	2565.1563	3		0	[2] Carbamidomethyl C|[7] Hydroxyl DKNP	no	6.44
LDLAGR	2620	8.03	2	643.3660	2		0		no	3.19
LDLAGR	2533	8.03	2	643.3653	1		0		no	7.89
LDLAGRDLTDYLMK	1225	7.84	1	1638.8282	3		0	[14] Hydroxyl DKNP	no	3.95
LDLAGRDLTDYLMK	8915	---	---	1638.8189	2		0	[14] Hydroxyl DKNP	no	6.99
MQKEITALAPSTMK	10528	---	---	1579.7929	2		0	[3] Hydroxyl DKNP|[14] Hydroxyl DKNP	no	6.23
MQKEITALAPSTMK	1485	7.63	2	1579.7943	3		0		no	4.16

Sequence	Peptide Ion	Score	Hits	Mass	Charge	Tags	Conflicts	Modifications	In quantitation	Drift time (ms)
								[3] Hydroxyl DKNP|[14] Hydroxyl DKNP		
MQKEITALAPSTMK	3174	6.78	1	1593.7988	3		0	[3] Hydroxyl DKNP|[3] Methyl K|[10] Hydroxyl DKNP	no	4.16
PEEHPVLLTEAPLNPK	1146	0.00	4	1782.9476	2		0		no	6.93
PEEHPVLLTEAPLNPK	1035	0.00	3	1782.9506	3		0		no	4.57
QEYDESGPSIVHR	1411	7.84	4	1515.7152	3		0		no	4.02
SFTTTAER	2107	0.00	1	911.4350	1		0		no	10.25
SYELPDGQVITIGNER	81	8.82	15	1789.8861	2		0		yes	6.72
SYELPDGQVITIGNER	1803	8.64	1	1789.8841	3		0		no	4.57
SYELPDGQVITIGNER	631	8.82	11	1789.8846	3		0		yes	4.57
SYELPDGQVITIGNER	515	8.64	1	1789.8892	2		0		no	6.58
TALAPSTMK	4125	---	---	934.4742	2		0	[9] Hydroxyl DKNP	no	3.81
TALAPSTMK	2216	0.00	1	934.4798	1		0	[9] Hydroxyl DKNP	no	10.66
TTGIVMDSGDGVTHTVPIYEGYALPHAILR	2362	7.54	2	3198.5904	4		0	[7] Hydroxyl DKNP	no	6.44
TTTAER	2371	0.00	1	677.3341	1		0		no	7.69
VAPEEHPVLLTEAPLNPK	2129	8.81	4	1953.0499	2		0		yes	7.76
VAPEEHPVLLTEAPLNPK	58	8.81	16	1953.0603	3		0		yes	4.92
VAPEEHPVLLTEAPLNPK	1997	7.00	1	1985.0436	3		0	[3] Hydroxyl DKNP|[7] Hydroxyl DKNP	no	5.06
VAPEEHPVLLTEAPLNPK	1398	7.00	1	1985.0365	2		0	[3] Hydroxyl DKNP|[7] Hydroxyl DKNP	no	7.62
VAPEEHPVLLTEAPLNPKANR	3073	7.21	2	2294.2417	4		0		no	4.78

Tags	
	Anova p-value ≤ 0.05
	power>0.8
	q<0.05

P35579

Myosin-9 OS=Homo sapiens OX=9606 GN=MYH9 PE=1 SV=4
51 peptides

Sequence	Peptide Ion	Score	Hits	Mass	Charge	Tags	Conflicts	Modifications	In quantitation	Drift time (ms)	Average Normalised Abundance	
											CTR	M[illegible]
ADFCIIHYAGK	7906	5.19	1	1293.6385	2		0	[4] Carbamidomethyl C	no	5.06	167.14	
AGVLAHLEEERDLK	3032	7.08	2	1578.8263	3		0		yes	3.95	582.22	[illegible]
ALEEAMEQKAELER	1760	6.98	2	1661.7914	3		0	[6] Oxidation M	no	4.22	1367.73	[illegible]
ALEEK	5266	0.00	1	588.3137	1		0		no	6.93	130.37	
ALELDSNLYR	463	8.06	2	1192.6254	2		0		yes	4.92	2287.45	[illegible]

Sequence	Peptide Ion	Score	Hits	Mass	Charge	Tags	Conflicts	Modifications	In quantitation	Drift time (ms)	Average Normalised Abundance
											CTR
ANLQIDQINTDLNLER	6732	7.23	2	1868.9508	3		0		no	4.57	85.27
ANLQIDQINTDLNLER	4211	7.23	2	1868.9519	2		0		no	6.99	202.42
ASITALEAK	1302	7.62	2	902.4980	2		0		no	3.88	1022.82
ASREEILAQAK	4181	7.27	2	1214.6603	2		0		no	5.06	152.42
ASREEILAQAKENEK	8067	5.86	1	1714.8734	3		0		no	4.43	69.21
DELADEIANSSGK	3110	5.27	1	1347.6037	3		0		no	3.32	130.89
DLQGRDEQSEEK	5489	6.58	1	1432.6438	3		0		no	3.53	78.04
EEVGEEAIVELVENGK	11622	5.46	1	1742.8385	2		0		no	6.65	49.50
ELEDATETADAMNR	2452	7.32	2	1580.6611	2		0	[13] Hydroxyl DKNP	no	5.75	773.03
ELEDATETADAMNR	14300	5.85	1	1596.6622	2		0	[12] Oxidation M|[13] Hydroxyl DKNP	no	5.75	52.06
ELEDATETADAMNREVSSLK	6618	5.73	1	2224.0230	3		0	[4] Hydroxyl DKNP	no	5.19	175.07
EMEAELEDERK	5883	6.24	1	1393.6024	3		0	[2] Oxidation M	no	3.67	108.61
EQEVNILK	1165	7.48	2	971.5279	2		0		no	4.09	1171.97
EQEVNILKK	3127	7.66	2	1099.6218	2		0		no	4.64	325.81
EQEVNILKK	7345	7.33	1	1099.6211	3		0		no	3.05	26.63
FDQLLAEEK	1305	7.61	2	1091.5499	2		0		yes	4.57	1321.43
FVSELWK	5928	5.70	1	907.4750	2		0		no	3.95	104.30
GALALEEK	2953	7.23	2	829.4534	2		0		no	3.67	621.42
GALALEEKR	3769	6.43	1	985.5404	2		0		no	4.36	93.29
GDLPFVVPR	5231	6.82	1	998.5534	2		0		no	4.29	72.25
IAEFTTNLTEEEEK	4460	7.38	2	1652.7778	2		0		no	6.37	233.80
IAEFTTNLTEEEEKSK	1347	7.57	2	1867.9068	3		0		no	4.43	979.17
IAQLEEELEEEQGNTELINDR	14702	---	---	2471.1615	2		0		no	8.59	10.30
IAQLEEELEEEQGNTELINDR	6631	6.61	2	2471.1612	3		0		no	5.75	119.25
IAQLEEQLDNETK	4311	6.85	2	1529.7565	2		0		no	5.96	161.58
IAQLEEQLDNETKER	1407	7.50	2	1814.9000	3		0		no	4.57	1224.12
IIGLDQVAGMSETALPGAFK	4075	6.84	2	2033.0437	2		0	[10] Oxidation M	no	7.62	360.90
IIGLDQVAGMSETALPGAFK	5141	6.84	2	2033.0551	3		0	[10] Oxidation M	no	5.26	285.35
IMGIPEEEQMGLLR	3101	6.95	2	1646.8000	2		0	[2] Oxidation M| [5] Hydroxyl DKNP	no	6.23	732.79
KANLQIDQINTDLNLER	5212	6.38	2	1997.0548	3		0		no	4.57	171.09
KEEELQAALAR	6454	6.38	1	1256.6618	2		0		no	4.92	168.39
KFDQLLAEEK	2449	6.67	2	1219.6372	2		0		no	5.06	327.38
KVEAQLQELQVK	5652	6.10	2	1411.7987	3		0		no	3.74	96.80
LEVNLQAMK	1558	6.93	2	1060.5514	2		0	[9] Hydroxyl DKNP	no	4.36	1106.39
LQQELDDLLVDLDHQR	6016	6.42	2	1948.9830	3		0		no	4.92	124.22
LQVELDNVTGLLSQSDSK	10953	6.50	1	1944.9973	2		0		no	7.34	42.51
NTDQASMPDNTAAQK	4098	4.98	1	1604.6995	2		0	[15] Methyl K	no	5.82	467.45
QAQQERDELADEIANSSGK	3255	6.42	2	2087.9697	3		0		no	4.57	439.38
QEEEMMAKEEELVK	4992	5.78	1	1721.7840	2		0		no	6.23	240.41
QLEEAEEEAQR	3690	7.36	2	1330.5986	2		0		no	5.06	362.25
QLEEAEEEAQRANASR	5068	7.01	2	1829.8517	3		0		no	4.71	389.25
QLLQANPILEAFGNAK	2308	7.74	2	1725.9382	2		0		no	6.93	596.60

Sequence	Peptide Ion	Score	Hits	Mass	Charge	Tags	Conflicts	Modifications	In quantitation	Drift time (ms)	Average Normalised Abundance
											CTR
QTLENERGELANEVK	2207	6.55	1	1728.8470	3		0		no	4.43	775.16
QTLENERGELANEVK	6834	5.37	1	1742.8722	2		0	[15] Methyl K	no	6.58	256.91
RGDLPFVVPR	10055	---	---	1154.6544	3		0		no	3.81	18.69
RGDLPFVVPR	3218	6.18	2	1154.6517	2		0		no	4.78	333.49
VAAYDKLEK	7053	5.99	1	1035.5604	2		0		no	4.64	36.92
VEAQLQELQVK	3108	7.12	2	1283.7010	2		0		no	5.12	393.43
VNKDDIQK	1921	6.11	1	1006.4966	2		0	[2] Hydroxyl DKNP | [4] Hydroxyl DKNP | [8] Hydroxyl DKNP	no	4.09	1008.85
VVFQEFR	1322	7.48	2	923.4874	2		0		no	3.95	916.20
YEILTPNSIPK	2666	6.80	2	1273.6854	2		0		no	5.06	396.01

Tags	
	Anova p-value ≤ 0.05
	power>0.8
	q<0.05

O43707

Alpha-actinin-4 OS=Homo sapiens OX=9606 GN=ACTN4 PE=1 SV=2
36 peptides

Sequence	Peptide Ion	Score	Hits	Mass	Charge	Tags	Conflicts	Modifications	In quantitation	Drift time (ms)	Average Normalised Abundance
											CTR
AGTQIENIDEDFR	961	6.01	2	1506.6955	2		0		no	5.54	1411.67
AGTQIENIDEDFR	1085	6.71	3	1506.6959	2		0		no	5.47	1548.11
AGTQIENIDEDFRDGLK	1915	7.86	2	1919.9250	3		0		no	4.43	564.56
ALDFIASK	2470	7.98	2	863.4727	2		0		no	3.67	380.82
DAKGISQEQMQEFR	5313	6.19	2	1695.7861	3		0	[3] Methyl K | [10] Oxidation M	no	3.95	145.34
DDPVTNLNNAFEVAEK	7967	6.42	1	1774.8352	2		0		no	6.37	86.85
DFIASK	5454	0.00	1	679.3541	1		0		no	7.76	87.80
EAQRIAESNHIK	894	5.60	1	1410.7251	2		0	[12] Hydroxyl DKNP	no	5.33	978.57
EAQRIAESNHIK	1992	---	---	1410.7076	3		0	[12] Hydroxyl DKNP	no	4.02	570.76
EGLLLWCQR	5931	7.61	2	1173.5980	2		0	[7] Carbamidomethyl C	no	4.64	88.85
ELPPDQAEYCIAR	4953	6.36	1	1560.7239	2		0	[10] Carbamidomethyl C	no	5.89	933.55
ETTDTDTADQVIASFK	3539	8.29	2	1740.8138	3		0		no	4.29	388.82
ETTDTDTADQVIASFK	918	8.29	7	1740.8040	2		0		no	6.72	1927.38
FAIQDISVEETSAK	1417	8.02	6	1536.7673	2		0		no	5.89	788.66
HRPELIEYDKLR	5539	6.41	1	1567.8441	4		0		no	3.46	151.99

Sequence	Peptide Ion	Score	Hits	Mass	Charge	Tags	Conflicts	Modifications	In quantitation	Drift time (ms)	Average Normalise Abundanc
											CTR
IAESNHIK	2520	6.45	2	926.4780	2		0	[8] Hydroxyl DKNP	no	4.02	606.18
ICDQWDALGSLTHSR	8303	6.36	2	1757.8207	3		0	[2] Carbamidomethyl C	no	4.57	108.39
KDDPVTNLNNAFEVAEK	1132	8.03	5	1902.9310	3		0		no	4.64	1379.27
KDDPVTNLNNAFEVAEK	8668	8.03	2	1902.9245	2		0		no	6.99	83.20
KTFTAWCNSHLR	370	4.81	1	1519.7497	2		0	[7] Carbamidomethyl C	no	6.16	2876.63
LEDFRDYR	4357	7.09	2	1112.5211	3		0		no	3.25	97.94
LSNRPAFMPSEGK	1216	6.49	2	1448.7070	3		0	[9] Hydroxyl DKNP	no	4.02	2134.55
MAPYQGPDAVPGALDYK	1516	7.55	4	1807.8421	2		0	[3] Hydroxyl DKNP	no	6.72	1117.10
MAPYQGPDAVPGALDYK	5436	7.55	1	1807.8416	3		0	[3] Hydroxyl DKNP	no	4.71	149.54
MAPYQGPDAVPGALDYK	6756	6.60	1	1823.8399	2		0	[3] Hydroxyl DKNP|[7] Hydroxyl DKNP	no	6.72	133.83
MLDAEDIVNTARPDEK	7399	7.80	1	1831.8569	2		0	[1] Oxidation M	no	7.48	47.77
MLDAEDIVNTARPDEK	816	7.96	3	1831.8660	3		0	[1] Oxidation M	no	4.71	2315.85
NFITAEELR	6655	7.48	1	1091.5626	2		0		no	4.57	191.83
QFASQANVVGPWIQTK	3261	7.93	2	1772.9151	2		0		no	6.99	296.24
QGEAEFNR	4673	7.05	2	949.4351	2		0		no	3.95	161.78
RDHALLEEQSK	5466	6.66	2	1324.6714	3		0		no	3.74	192.31
RDHALLEEQSK	231	4.89	2	1340.6713	2		0	[2] Hydroxyl DKNP	yes	5.26	1.85e+004
RDHALLEEQSK	586	4.00	1	1340.6699	2		0	[2] Hydroxyl DKNP	yes	5.33	6262.96
RDHALLEEQSK	2312	4.96	1	1340.6671	2		0	[11] Hydroxyl DKNP	no	5.26	1191.77
RQFASQANVVGPWIQTK	1292	4.42	1	1945.0107	3		0	[17] Hydroxyl DKNP	no	5.47	710.77
RQFASQANVVGPWIQTK	1260	4.42	1	1945.0086	2		0	[17] Hydroxyl DKNP	no	7.48	611.89
RTIPWLEDR	2840	5.28	1	1184.6134	2		0		no	4.64	1990.97
STLPDADRER	2083	7.60	2	1158.5631	3		0		no	3.39	395.15
STLPDADRER	1827	7.60	2	1158.5629	2		0		no	4.71	562.34
STLPDADREREAILAIHKEAQR	1188	4.94	1	2518.3030	3		0		no	6.51	1214.71
TINEVENQILTR	4721	8.38	1	1428.7559	3		0		no	3.88	144.75
TINEVENQILTR	382	8.50	7	1428.7558	2		0		yes	5.47	5626.98
VGWEQLLTTIAR	2486	7.62	2	1385.7630	2		0		no	5.47	523.35
VLAGDKNFITAEELR	1987	7.33	4	1674.8875	3		0		no	4.29	948.60
VQQLVPK	1084	6.41	1	810.4931	2		0		no	3.60	589.05
YLDIPK	3752	6.73	1	747.4164	1		0		no	9.00	84.01
YLDIPK	886	6.73	2	747.4155	2		0		no	3.74	515.16

Tags	
	Anova p-value ≤ 0.05
	power>0.8
	q<0.05

P35527

Keratin_ type I cytoskeletal 9 OS=Homo sapiens OX=9606 GN=KRT9 PE=1 SV=3
35 peptides

Sequence	Peptide Ion	Score	Hits	Mass	Charge	Tags	Conflicts	Modifications	In quantit
DIDNTR	826	0.00	4	732.3409	1		0		no
DIENQYETQITQIEHEVSSSGQEVQSSAK	3538	7.20	1	3263.4997	4		0		no
DIENQYETQITQIEHEVSSSGQEVQSSAK	665	7.48	9	3263.5006	3		0		no
DQIVDLTVGNNK	502	6.25	2	1314.6675	2		0		no
EIETYHNLLEGGQEDFESSGAGK	247	8.75	16	2509.1238	3		0		no
FEMEQNLR	6330	6.49	1	1065.4998	2		0		no
FSSSSGYGGGSSR	393	8.68	16	1234.5231	2		0		no
GGSGGSHGGGSGFGGESGGSYGGGEEASGSGGGYGGGSGK	3776	6.57	1	3222.2670	3		0		no
GGSGGSYGGGGSGGGYGGGSGSR	2068	7.02	4	1790.7165	2		0		no
GGSGGSYGGGGSGGGYGGGSGSR	6570	---	---	1790.7195	3		0		no
GGSGGSYGGGGSGGGYGGGSGSR	9107	---	---	1790.7140	3		0		no
GGSGGSYGGGGSGGGYGGGSGSR	1550	8.49	7	1790.7159	2		0		no
HGVQELEIELQSQLSK	1536	8.05	11	1836.9565	3		0		no
IKFEMEQNLR	2613	7.41	1	1306.6601	2		0		no
IKFEMEQNLR	1124	7.41	6	1306.6703	3		0		no
IKFEMEQNLR	1021	7.78	11	1322.6637	2		0	[5] Oxidation M	no
IKFEMEQNLR	318	7.78	11	1322.6687	3		0	[5] Oxidation M	no
+IKFEMEQNLR	1594	0.00	1	1305.6424	3		0	[N-term] Ammonia-loss|[5] Oxidation M	no
+KGPAAIQK	2505	6.48	1	839.4845	2		0	[N-term] Formyl K N-TERM	no
LASYLDKVQALEEANNDLENK	5364	7.02	1	2376.1729	3		0		no
LASYLDKVQALEEANNDLENK	2097	7.02	5	2376.1743	3		0		no
LDDFR	1077	0.00	1	664.3184	1		0		no
LDIDNTR	428	0.00	12	845.4244	1		0		no
LDIDNTR	4986	---	---	845.4248	2		0		no
MTLDDFR	8128	---	---	896.4082	1		0		no
MTLDDFR	211	8.47	13	896.4091	2		0		no
MTLDDFR	229	7.82	12	912.4037	2		0	[1] Oxidation M	no
MTLDDFR	1469	6.73	3	912.4023	2		0	[1] Oxidation M	no
NDMRQEYEQLIAK	786	0.00	1	1619.7604	2		0		no
NYSPYYNTIDDLKDQIVDLTVGNNK	1269	7.48	7	2901.3942	3		0		no
QEYEQLIAK	455	8.19	10	1120.5719	2		0		no
QFSSSYLSR	1273	8.59	5	1073.5278	2		0		no
QGVDADINGLR	167	8.79	16	1156.5867	2		0		no
QVLDNLTMEK	1264	7.28	8	1189.6023	2		0		no
QVLDNLTMEK	557	7.79	4	1205.5979	2		0		no

Sequence	Peptide Ion	Score	Hits	Mass	Charge	Tags	Conflicts	Modifications	In quantit
								[8] Oxidation M	
SGGGGGGGLGSGGSIR	228	9.20	16	1231.5931	2		0		yes
SGGGGGGGLGSGGSIRSSYSR	8742	5.35	1	1811.8693	3		0		no
STMQELNSR	1565	6.88	4	1080.4885	2		0	[3] Oxidation M	no
TLDDFR	1316	0.00	1	765.3658	1		0		no
TLLDIDNTR	8825	---	---	1059.5552	1		0		yes
TLLDIDNTR	78	8.43	16	1059.5632	2		0		yes
TLLDIDNTRMTLDDFR	2256	6.37	2	1953.9454	3		0	[13] Hydroxyl DKNP	no
TLNDMRQEYEQLIAK	5703	7.81	1	1850.9137	2		0		no
TLNDMRQEYEQLIAK	1529	4.81	1	1850.9264	2		0		no
TLNDMRQEYEQLIAK	552	7.83	12	1850.9205	3		0		no
TLNDMRQEYEQLIAK	6898	6.84	1	1866.9021	2		0	[3] Hydroxyl DKNP	yes
TLNDMRQEYEQLIAK	351	7.06	6	1866.9174	3		0	[3] Hydroxyl DKNP	yes
TLNDMRQEYEQLIAK	3239	6.19	1	1866.9150	3		0	[4] Hydroxyl DKNP	no
VQALEEANNDLENK	2407	5.82	1	1585.7412	2		0		no

Tags	
	Anova p-value ≤ 0.05
	power>0.8
	q<0.05

P02545

Prelamin-A/C OS=Homo sapiens OX=9606 GN=LMNA PE=1 SV=1
29 peptides

Sequence	Peptide Ion	Score	Hits	Mass	Charge	Tags	Conflicts	Modifications	In quantitation	Drift time (ms)	Average Normalis Abundances	
											CTR	MCD
AAQAR	617	0.00	1	515.2850	1		0		yes	5.89	2193.57	549
AAYEAELGDARK	10353	7.22	1	1292.6311	2		0		no	5.33	50.22	
AAYEAELGDARK	3850	7.22	2	1292.6350	3		0		no	3.60	320.85	15
AQHEDQVEQYKK	6971	6.18	1	1501.7082	3		0		no	3.81	50.43	1
ATRSGAQASSTPLSPTR	2332	5.46	1	1718.8606	2		0	[12] Hydroxyl DKNP|[15] Hydroxyl DKNP	no	6.51	397.75	179
DLEDSLAR	5382	7.01	1	917.4473	2		0		no	3.81	193.47	11
EAALSTALSEKR	4930	7.62	1	1274.6761	2		0		no	5.40	265.48	7
EAALSTALSEKR	4314	7.62	2	1274.6835	3		0		no	3.74	205.41	27
EREMAEMR	1412	6.23	1	1050.4765	2		0		no	4.22	725.92	36
ITESEEVVSR	953	8.03	7	1147.5724	2		0		no	4.43	1870.80	150
+KEGDLIAAQAR	2634	4.62	1	1198.6314	3		0	[N-term] Formyl K N-TERM	no	3.67	676.53	15

Sequence	Peptide Ion	Score	Hits	Mass	Charge	Tags	Conflicts	Modifications	In quantitation	Drift time (ms)	Average Normalis Abundances	
											CTR	MCD
LADALQELR	539	8.07	7	1027.5681	2		0		no	4.29	2130.53	200
LAVYIDR	4793	4.25	1	848.4813	2		0		no	3.95	343.29	10
LAVYIDR	1354	7.95	4	848.4723	2		0		no	3.74	1083.52	76
LEAALGEAK	2430	7.41	2	900.4845	2		0		no	3.81	1101.91	38
LLEGEEER	1649	7.65	3	973.4717	2		0		no	4.22	1135.49	72
LLEGEEERLR	1689	7.01	2	1242.6578	3		0		no	3.53	462.88	32
LLEGEEERLR	4798	7.01	1	1242.6528	2		0		no	5.26	120.48	4
LQEKEDLQELNDR	2063	7.30	5	1628.8041	3		0		no	4.09	622.97	49
LQTMKEELDFQK	1694	6.71	1	1524.7611	3		0	[5] Hydroxyl DKNP	no	3.95	944.67	37
LQTMKEELDFQK	11985	---	---	1524.7252	2		0	[5] Hydroxyl DKNP	no	5.89	18.45	
LRDLEDSLAR	4652	7.76	1	1186.6295	3		0		no	3.53	94.34	2
LRDLEDSLAR	2019	7.76	3	1186.6226	2		0		no	4.99	596.79	37
LSPSPTSQR	1572	8.02	5	971.5031	2		0		no	3.95	693.96	31
MQQQLDEYQELLDIK	5506	7.63	2	1908.9074	2		0	[1] Oxidation M	no	7.20	201.88	3
MQQQLDEYQELLDIK	8595	7.63	2	1908.9198	3		0	[1] Oxidation M	no	4.57	81.54	
NSNLVGAAHEELQQSR	1846	7.40	4	1751.8547	3		0		no	4.29	691.64	58
RTLEGELHDLR	123	5.38	1	1337.7303	3		0		yes	4.09	1.68e+004	4.91e-
SGAQASSTPLSPTR	991	7.94	6	1358.6784	2		0		no	5.12	1455.42	138
SLETENAGLR	687	7.81	6	1088.5449	2		0		yes	4.22	3327.10	228
SNEDQSMGNWQIK	9562	5.95	1	1551.6635	2		0	[4] Hydroxyl DKNP	no	5.75	60.94	2
SVGGSGGGSFGDNLVTR	1966	7.72	6	1565.7414	2		0		no	5.89	428.94	60
SVGGSGGGSFGDNLVTR	2493	4.46	1	1565.7296	2		0		no	5.82	609.69	78
TALINSTGEEVAMR	2204	6.65	2	1506.7291	2		0	[5] Hydroxyl DKNP	no	5.75	644.96	35
VAVEEVDEEGK	1198	5.13	1	1202.5892	2		0		no	4.71	965.40	74
VAVEEVDEEGKFVR	6406	7.85	2	1604.7978	2		0		no	6.44	105.81	5
VYIDR	3961	0.00	1	664.3537	1		0		no	7.96	133.79	7

Tags	
	Anova p-value ≤ 0.05
	power>0.8
	q<0.05

P11047

Laminin subunit gamma-1 OS=Homo sapiens OX=9606 GN=LAMC1 PE=1 SV=3
29 peptides

Sequence	Peptide Ion	Score	Hits	Mass	Charge	Tags	Conflicts	Modifications	In quantitation	Drift time (ms)	Average Normalised Abundances	
											CTR	MCI
AFDITYVR	5477	6.63	1	983.5064	2		0		no	4.22	141.88	
CELCDDGYFGDPLGR	9870	6.63	1	1772.7182	2		0	[1] Carbamidomethyl	no	6.37	52.63	

Sequence	Peptide Ion	Score	Hits	Mass	Charge	Tags	Conflicts	Modifications	In quantitation	Drift time (ms)	Average Normalised Abundances	
											CTR	MC[illegible]
								C | [4] Carbamidomethyl C				
CIYNTAGFYCDR	9838	7.10	2	1538.6418	2		0	[1] Carbamidomethyl C | [10] Carbamidomethyl C	no	5.89	83.66	
CKDGFFGNPLAPNPADK	9085	5.94	1	1846.8686	3		0	[1] Carbamidomethyl C	no	4.43	37.99	
CVCKDNVEGFNCER	7247	5.99	1	1785.7484	3		0	[1] Carbamidomethyl C | [3] Carbamidomethyl C | [12] Carbamidomethyl C	no	4.36	104.38	
DIRNLEDIR	725	5.35	1	1142.5996	2		0		yes	4.64	4790.00	1
DIRNLEDIR	3293	5.35	1	1142.6045	3		0		yes	3.32	468.25	
EAQDVKDVDQNLMDR	6821	4.99	1	1774.8408	3		0		no	4.78	71.73	
EAQDVKDVDQNLMDR	2036	6.32	3	1790.8103	3		0	[14] Hydroxyl DKNP	no	4.16	741.16	
EAQQALGSAAADATEAK	1813	7.71	2	1630.7759	2		0		no	6.30	571.59	
EIASR	1130	0.00	1	574.3084	1		0		no	6.58	531.80	
EVVCTNCPTGTTGK	11017	6.00	1	1522.6750	2		0	[4] Carbamidomethyl C | [7] Carbamidomethyl C	no	5.61	37.80	
GKTEQQTADQLLAR	7553	7.31	1	1557.8071	2		0		no	6.16	64.62	
GKTEQQTADQLLAR	2030	7.31	2	1557.8098	3		0		no	3.88	379.76	
LCQCSDNIDPNAVGNCNR	13792	---	---	2105.8729	2		0	[2] Carbamidomethyl C | [4] Carbamidomethyl C | [16] Carbamidomethyl C	no	7.41	25.18	
LCQCSDNIDPNAVGNCNR	7453	6.44	2	2105.8684	3		0	[2] Carbamidomethyl C | [4] Carbamidomethyl C | [16] Carbamidomethyl C	no	4.64	103.22	
LIEIASR	947	7.28	2	800.4748	2		0		no	3.67	716.20	
LKDYEDLR	201	5.45	2	1050.5483	2		0		yes	4.29	4252.09	1.2[illegible]
LNEIEGTLNK	1223	6.08	2	1143.6145	2		0	[10] Methyl K	no	4.85	1392.01	
LNEIEGTLNKAK	3572	4.59	1	1344.7088	2		0	[9] Hydroxyl DKNP	no	5.33	398.00	
LNTFGDEVFNDPK	6007	5.99	1	1494.7005	2		0		no	6.02	88.80	
LSAEDLVLEGAGLR	2673	7.77	3	1441.7749	2		0		no	5.61	722.78	
NATSTKAEAER	1666	5.19	1	1176.5769	3		0		no	3.19	986.65	
NLEDIRK	1882	7.30	2	886.4840	2		0		no	3.67	745.73	
NTIEETGNLAEQAR	1116	8.06	4	1544.7407	2		0		no	5.82	1482.28	1
QLQEAEK	376	4.30	1	844.4371	1		0		yes	9.42	2994.36	5
QLQEAEK	374	4.30	1	844.4405	2		0		yes	3.74	3627.86	6
SQECYFDPELYR	6214	7.20	2	1605.6775	2		0		no	6.09	187.58	

Sequence	Peptide Ion	Score	Hits	Mass	Charge	Tags	Conflicts	Modifications	In quantitation	Drift time (ms)	Average Normalised Abundances	
											CTR	MC[illegible]
								[4] Carbamidomethyl C				
SWPGCQECPACYR	12856	5.61	1	1669.6463	2		0	[5] Carbamidomethyl C|[8] Carbamidomethyl C|[11] Carbamidomethyl C	no	6.02	51.26	
TEQQTADQLLAR	4148	7.14	2	1372.6785	2		0		no	5.40	414.56	
TGQCECQPGITGQHCER	9147	6.65	1	2016.8155	3		0	[4] Carbamidomethyl C|[6] Carbamidomethyl C|[15] Carbamidomethyl C	no	4.57	70.89	
TKEVVCTNCPTGTTGK	10010	---	---	1751.7929	2		0	[6] Carbamidomethyl C|[9] Carbamidomethyl C	no	6.23	225.11	
TKEVVCTNCPTGTTGK	3059	4.35	1	1751.8005	3		0	[6] Carbamidomethyl C|[9] Carbamidomethyl C	no	4.36	1072.38	
VSVPLIAQGNSYPSETTVK	6489	6.12	1	1989.0266	2		0		no	7.69	39.21	

Tags	
	Anova p-value ≤ 0.05
	power>0.8
	q<0.05

O00468

Agrin OS=Homo sapiens OX=9606 GN=AGRN PE=1 SV=6
23 peptides

Sequence	Peptide Ion	Score	Hits	Mass	Charge	Tags	Conflicts	Modifications	In quantitation	Drift time (ms)
ALGPAGCEADASAPATCAEMR	4590	6.23	2	2120.8866	3		0	[7] Carbamidomethyl C|[17] Carbamidomethyl C|[20] Oxidation M	no	5.61
CDRCEPGFWNFR	11127	5.72	1	1642.6744	3		0	[1] Carbamidomethyl C|[4] Carbamidomethyl C	no	4.16
CEPGFWNFR	6542	6.67	2	1211.5224	2		0	[1] Carbamidomethyl C	no	4.71
DLGPGK	22	6.28	1	601.3102	1		0	[1] Hydroxyl DKNP	yes	7.13
DLGPGK	164	6.28	1	601.3107	2		0		yes	2.91

Sequence	Peptide Ion	Score	Hits	Mass	Charge	Tags	Conflicts	Modifications	In quantitation	Drift time (ms)
								[1] Hydroxyl DKNP		
EAACLQQTQIEEAR	2022	7.51	2	1645.7699	2		0	[4] Carbamidomethyl C	no	6.30
FGALCEAETGR	1189	7.61	2	1209.5561	2		0	[5] Carbamidomethyl C	yes	4.64
FNAVCLSR	2818	6.59	1	965.4766	2		0	[5] Carbamidomethyl C	no	4.02
GAPEGTVCGSDGADYPGECQLLR	6144	5.99	1	2408.0365	3		0	[8] Carbamidomethyl C | [19] Carbamidomethyl C	no	5.06
GMLCGFGAVCEPNAEGPGR	9420	6.62	1	1993.8405	2		0	[2] Oxidation M | [4] Carbamidomethyl C | [10] Carbamidomethyl C	no	6.99
GMLCGFGAVCEPNAEGPGR	11844	6.62	1	1993.8435	3		0	[2] Oxidation M | [4] Carbamidomethyl C | [10] Carbamidomethyl C	no	4.29
KFDGPCDPCQGALPDPSR	4271	6.30	1	2015.8807	3		0	[6] Carbamidomethyl C | [9] Carbamidomethyl C	no	4.29
LLDVNNQR	1573	7.11	2	970.5139	2		0		yes	4.16
QENVFK	2053	6.12	1	763.3862	1		0		no	8.52
QENVFK	5852	6.12	1	763.3863	2		0		no	3.60
QVDVTSFAGHPCTRASGHPCLNGASCVPR	5323	5.37	1	3137.4264	3		0	[12] Carbamidomethyl C | [20] Carbamidomethyl C | [26] Carbamidomethyl C	no	6.02
SADGLTASCLCPATCR	4728	7.00	2	1738.7427	2		0	[9] Carbamidomethyl C | [11] Carbamidomethyl C | [15] Carbamidomethyl C	no	6.37
SAGDVDTLAFDGR	2090	7.80	2	1322.6156	2		0		no	5.06
SELFGETAR	2290	7.45	2	1008.4871	2		0		no	4.16
SIESTLDDLFR	5739	7.02	2	1294.6402	2		0		no	5.12
STVPVNTNR	4852	6.66	2	986.5121	2		0		no	3.95
TEATQGLVLWSGK	8250	6.04	1	1388.7145	2		0		no	5.47
TFVGAGLR	6310	6.73	1	819.4545	2		0		no	3.53
VLGAPVPAFEGR	1880	7.28	2	1211.6651	2		0		no	4.64
VLPEGGAQCECPLGR	4048	7.07	2	1641.7583	2		0	[9] Carbamidomethyl C | [11] Carbamidomethyl C	no	6.09
VTCDGAYRPVCAQDGR	4970	7.02	2	1823.8043	3		0	[3] Carbamidomethyl C | [11]	no	4.22

Sequence	Peptide Ion	Score	Hits	Mass	Charge	Tags	Conflicts	Modifications	In quantitation	Drift time (ms)
								Carbamidomethyl C		

Tags	
	Anova p-value ≤ 0.05
	power>0.8
	q<0.05

P68871

Hemoglobin subunit beta OS=Homo sapiens OX=9606 GN=HBB PE=1 SV=2
23 peptides

Sequence	Peptide Ion	Score	Hits	Mass	Charge	Tags	Conflicts	Modifications	In quantitation	Drift time (ms)	Average N Abundanc
											CTR
DEVGGEALGR	1459	0.00	3	1001.4772	1		0		no	10.60	488.93
EFTPPVQAAYQK	4263	---	---	1377.6980	3		0		no	3.88	218.40
EFTPPVQAAYQK	614	8.55	10	1377.6949	2		0		no	5.26	3573.67
FFESFGDLSTPDAVMGNPK	3876	7.47	2	2073.9328	2		0	[17] Hydroxyl DKNP	no	7.55	495.07
FFESFGDLSTPDAVMGNPK	5776	---	---	2073.9368	3		0	[17] Hydroxyl DKNP	no	5.12	219.17
FFESFGDLSTPDAVMGNPK	3545	---	---	2087.9455	3		0	[11] Hydroxyl DKNP|[19] Methyl K	no	5.19	439.39
FFESFGDLSTPDAVMGNPK	3393	7.62	3	2087.9426	2		0	[11] Hydroxyl DKNP|[19] Methyl K	no	7.48	457.88
FFESFGDLSTPDAVMGNPK	1365	8.39	3	2089.9277	2		0	[17] Hydroxyl DKNP|[18] Hydroxyl DKNP	no	7.48	2350.72
FFESFGDLSTPDAVMGNPK	3948	8.39	3	2089.9267	3		0	[17] Hydroxyl DKNP|[18] Hydroxyl DKNP	no	5.19	382.26
FFESFGDLSTPDAVMGNPKVK	1687	7.31	3	2301.0986	3		0	[11] Hydroxyl DKNP	no	4.99	707.49
FFESFGDLSTPDAVMGNPKVK	852	6.92	1	2315.1085	3		0	[17] Hydroxyl DKNP|[19] Methyl K	no	4.85	1411.56
GEALGR	565	0.00	1	601.3214	1		0		yes	6.65	6271.54
+KVLGAFSDGLAHLDNLK	784	6.25	1	1856.9748	4		0	[N-term] Formyl K N-TERM|[1] Hydroxyl DKNP|[8] Hydroxyl DKNP	yes	4.22	1626.07
+KVLGAFSDGLAHLDNLK	848	6.25	1	1856.9735	3		0	[N-term] Formyl K N-TERM|[1] Hydroxyl DKNP|[8] Hydroxyl DKNP	yes	5.19	1914.44
LHVDPENFR	2359	7.54	3	1125.5689	2		0		no	4.64	1004.25
LLVVYPWTQR	224	9.05	10	1273.7223	2		0		no	5.19	1.12e+004

Sequence	Peptide Ion	Score	Hits	Mass	Charge	Tags	Conflicts	Modifications	In quantitation	Drift time (ms)	Average N Abundanc
											CTR
LLVVYPWTQR	1727	8.24	4	1289.7100	2		0	[6] Hydroxyl DKNP	no	5.19	959.86
LLVVYPWTQR	8274	---	---	1289.7090	3		0	[6] Hydroxyl DKNP	no	3.88	64.03
SAVTALWGK	3979	8.85	1	931.5135	1		0		no	10.80	129.41
SAVTALWGK	494	9.11	7	931.5147	2		0		no	3.88	2951.28
SAVTALWGKVNVDEVGGEALGR	8268	7.55	1	2241.1747	3		0	[9] Methyl K	no	5.96	63.46
TALWGK	1499	0.00	4	674.3754	1		0		no	7.96	408.77
VDEVGGEALGR	3107	0.00	2	1100.5462	1		0		no	11.70	236.00
VGGEALGR	1568	0.00	1	757.4178	1		0		no	9.00	971.79
VHLTPEEK	1190	8.71	6	951.5019	2		0		no	4.22	1433.67
VLGAFSDGLAHLDNLK	2370	7.93	4	1668.8838	3		0		no	4.64	535.43
VNVDEVGGEALGR	4712	9.45	3	1313.6599	3		0		yes	3.67	238.05
VNVDEVGGEALGR	50	9.45	14	1313.6652	2		0		yes	5.19	3.61e+004
VNVDEVGGEALGR	6459	7.09	1	1329.6437	2		0	[2] Hydroxyl DKNP	no	5.12	135.10
VNVDEVGGEALGR	5164	7.17	1	1329.6472	2		0	[4] Hydroxyl DKNP	no	5.19	256.27
VTALWGK	2236	0.00	2	773.4440	1		0		no	8.93	263.20
VTALWGK	6161	---	---	773.4453	2		0		no	3.46	60.29

Tags	
	Anova p-value ≤ 0.05
	power>0.8
	q<0.05

P55268

Laminin subunit beta-2 OS=Homo sapiens OX=9606 GN=LAMB2 PE=1 SV=2
25 peptides

Sequence	Peptide Ion	Score	Hits	Mass	Charge	Tags	Conflicts	Modifications	In quantitation	Drift time (ms)	Average Normalise Abundanc
											CTR
AAQLDGLEAR	768	7.56	5	1042.5354	2		0		yes	4.43	2634.42
AGNSLAASTAEETAGSAQGR	4395	7.64	2	1847.8548	2		0		no	7.20	244.77
ALAEGGSILSR	662	7.99	5	1072.5874	2		0		yes	4.43	2406.17
AQEAEQLLR	850	7.66	5	1056.5540	2		0		no	4.36	1519.89
AQGIAQGAIR	998	7.22	5	983.5491	2		0		no	3.95	1709.16
ARSWAEDEK	701	4.46	1	1104.5198	2		0	[9] Methyl K	no	4.43	2222.64
ARSWAEDEK	3099	---	---	1104.5154	1		0	[9] Methyl K	no	12.26	178.77
EDFNSKHMANQR	10477	3.85	1	1491.6433	2		0	[10] Hydroxyl DKNP	no	5.40	132.33
GAVADTRDTEQTLYQVQER	2202	6.44	2	2179.0413	3		0		no	5.40	544.30
GLNCEQCQDFYR	7961	6.47	1	1588.6385	2		0	[4] Carbamidomethyl C|[7]	no	5.82	70.72

Sequence	Peptide Ion	Score	Hits	Mass	Charge	Tags	Conflicts	Modifications	In quantitation	Drift time (ms)	Average Normalised Abundances
											CTR
								Carbamidomethyl C			
GPLGDQYQTVK	1650	7.09	2	1204.6013	2		0		no	4.85	946.82
GSCYPATGDLLVGR	6979	6.16	2	1464.7035	2		0	[3] Carbamidomethyl C	no	5.61	133.37
HRTEALMDAQK	2827	5.59	2	1298.6543	3		0		no	3.60	527.60
HSNFLGAYDSIR	3474	4.07	1	1394.6562	2		0	[3] Hydroxyl DKNP	no	5.40	433.59
IQNVVTSFAPQRR	2606	5.00	1	1514.8425	2		0		no	6.23	206.21
LGIVQGIVGAR	1942	7.46	2	1081.6618	2		0		no	4.50	556.89
LQEGQTLEFLVASVPK	11186	---	---	1757.9607	3		0		no	4.71	28.34
LQEGQTLEFLVASVPK	8419	6.02	1	1757.9472	2		0		no	6.86	81.60
LQELEGTYEENER	3070	7.38	2	1608.7258	2		0		no	6.02	320.31
LQELEGTYEENERALESK	5983	6.51	1	2137.0197	3		0		no	4.92	149.21
LVTPGETPSWTGSGFVR	1893	4.24	1	1789.8968	2		0		yes	6.72	791.67
NTSAASTAQLVEATEELRR	11877	5.44	1	2046.0421	3		0		no	4.57	62.22
RAEQLLQDAR	2406	6.69	2	1198.6411	2		0		no	5.12	376.58
RAEQLLQDAR	2076	4.64	1	1198.6481	2		0		no	4.85	875.83
SLADVDAILAR	1111	7.60	4	1142.6293	2		0		no	4.71	946.00
VLDPAIPIPDPYSSR	7887	6.40	1	1638.8509	2		0		no	6.23	94.50
VVQDLAAR	3056	4.66	2	870.4904	2		0		no	3.95	347.01
VVQDLAAR	1450	7.18	2	870.4883	2		0		no	3.81	1147.27
YSEIEPSTEGEVIYR	3144	6.88	2	1770.8257	2		0		no	6.72	361.27

Tags	
	Anova p-value ≤ 0.05
	power>0.8
	q<0.05

P48681

Nestin OS=Homo sapiens OX=9606 GN=NES PE=1 SV=2
24 peptides

Sequence	Peptide Ion	Score	Hits	Mass	Charge	Tags	Conflicts	Modifications	In quantitation	Drift time (ms)	Average Normalised Abundances	
											CTR	MCD
ALEEQNELLSAELGGLR	13351	---	---	1840.9461	3		0		no	4.78	22.98	1.85
ALEEQNELLSAELGGLR	7274	6.59	2	1840.9430	2		0		no	6.93	101.65	22.51
AQDAPLSLLQTQGGR	6367	6.08	2	1553.7921	2		0		no	5.96	181.66	160.06
AQDAPLSLLQTQGGRK	4454	6.46	2	1681.9120	3		0		no	4.29	152.47	86.56
AQSADTSWR	3308	5.68	1	1020.4596	2		0		yes	4.09	1786.14	250.91
DNLAEELEGVAGR	4737	6.60	2	1371.6628	2		0		no	5.19	214.98	77.00
EGRLELQQLQAER	1756	6.98	2	1568.8278	3		0		no	3.88	529.57	350.94
EGRLELQQLQAER	13147	---	---	1568.8038	2		0		no	6.02	18.71	26.56

Sequence	Peptide Ion	Score	Hits	Mass	Charge	Tags	Conflicts	Modifications	In quantitation	Drift time (ms)	Average Normalised Abundances	
											CTR	MCD
EIQDSQVPLEKETLK	4473	5.69	1	1755.9056	3		0		no	4.22	114.76	104.65
GLVEGASVK	3668	6.25	2	858.4707	2		0		no	3.74	307.01	184.99
GPPAPAPEVEELAR	2665	6.81	2	1431.7266	2		0		no	5.61	403.76	111.41
LELQFPR	2133	7.01	2	901.5011	2		0		no	3.95	399.01	172.16
LELQQLQAER	3784	7.19	2	1226.6484	2		0		no	4.92	294.53	113.45
LLEEENQESLR	1787	7.08	2	1358.6651	2		0		no	5.19	720.03	755.22
RLEAYLAR	3569	5.75	1	990.5449	2		0		no	4.43	166.03	63.12
SLDQEIARPLENENQEFLK	8931	6.28	2	2272.1185	3		0		no	4.85	48.17	10.84
SLEDENKEAFR	5088	6.04	2	1336.6274	3		0		no	3.67	240.69	304.61
SLEEDLETLK	4200	6.24	2	1175.5899	2		0		no	4.78	275.47	152.09
SLEEQDQETLR	3045	6.73	2	1346.6294	2		0		no	5.12	374.81	207.84
SLETEILESLK	2762	6.58	2	1260.6829	2		0		no	5.40	212.30	120.53
SLKEESVEAVK	5683	5.18	1	1217.6445	2		0		no	4.99	88.12	21.28
SPEEVDKESQR	2034	5.21	1	1302.5957	2		0		yes	4.85	804.41	710.13
SPEEVDKESQR	4505	4.58	1	1318.5979	2		0	[6] Hydroxyl DKNP	no	5.12	360.02	91.06
TSLSFQDPK	1470	6.23	2	1021.5106	2		0		yes	4.22	1272.40	483.67
VAIPASVLPGPEEPGGQR	7035	6.31	1	1772.9160	2		0		no	6.51	64.72	40.17
VKALEEQNELLSAELGGLR	8374	5.61	2	2068.0932	3		0		no	4.71	74.10	15.38

Tags	
	Anova p-value ≤ 0.05
	power>0.8
	q<0.05

P13647

Keratin_ type II cytoskeletal 5 OS=Homo sapiens OX=9606 GN=KRT5 PE=1 SV=3
22 peptides

Sequence	Peptide Ion	Score	Hits	Mass	Charge	Tags	Conflicts	Modifications	In quantitation	Drift time (ms)	Average Normalise Abundanc
											CTR
AENEFVMLKK	4085	0.00	1	1223.6123	2		0	[9] Hydroxyl DKNP	no	4.85	680.64
AQYEEIANR	383	7.95	14	1092.5220	2		0		yes	4.36	1.09e+004
GRLDSELR	833	7.35	7	944.5026	2		0		no	4.16	4014.17
KDVDAAYMNK	2541	5.42	2	1153.5530	2		0		no	4.50	1050.29
LDSELRNMQDLVEDFK	6254	6.25	1	1996.9492	3		0	[2] Hydroxyl DKNP|[7] Hydroxyl DKNP|[16] Methyl K	no	4.64	577.75
LDSELRNMQDLVEDFK	1931	6.25	1	1996.9439	2		0	[2] Hydroxyl DKNP|[7] Hydroxyl DKNP|[16] Methyl K	no	7.20	2331.59
LLREYQELMNTK	6291	6.25	1	1536.7902	3		0		no	4.02	1870.51
QLDSIVGER	797	7.71	9	1015.5318	2		0		no	4.22	2204.01

Sequence	Peptide Ion	Score	Hits	Mass	Charge	Tags	Conflicts	Modifications	In quantitation	Drift time (ms)	Average Normalise Abundanc
											CTR
QLDSIVGERGR	859	5.81	1	1228.6527	2		0		no	5.06	4146.08
SFSTASAITPSVSR	1833	7.19	6	1409.7090	2		0		no	5.33	807.32
TEAESWYQTK	1734	7.87	4	1241.5758	2		0		no	4.85	2011.05
TEAESWYQTKYEELQQTAGR	5098	7.00	2	2417.1047	3		0		no	5.12	608.64
TSFTSVSR	2579	7.16	2	883.4408	2		0		no	3.74	791.71
TTAENEFVMLK	2247	5.77	2	1297.6048	2		0	[11] Hydroxyl DKNP	no	5.12	606.49
TTAENEFVMLKK	6585	---	---	1409.7155	2		0		no	5.75	285.84
TTAENEFVMLKK	1234	7.95	2	1409.7227	3		0		no	3.60	2960.57
TTAENEFVMLKK	1797	6.91	1	1425.7204	3		0	[9] Oxidation M	no	3.60	5240.18
TTAENEFVMLKK	3118	7.16	2	1425.7147	2		0	[11] Hydroxyl DKNP	yes	5.54	477.80
TTAENEFVMLKK	530	7.16	6	1425.7179	3		0	[11] Hydroxyl DKNP	yes	3.60	3217.70
VDALMDEINFMK	1962	3.73	1	1424.6886	2		0		no	5.54	467.64
VDALMDEINFMK	7319	6.38	1	1440.6623	2		0	[9] Hydroxyl DKNP	no	5.40	1347.38
VSLAGACGVGGYGSR	1176	7.84	6	1409.6733	2		0	[7] Carbamidomethyl C	no	5.40	2536.54
WTLLQEQGTK	987	5.47	1	1202.6297	2		0		no	4.78	1149.12
YEEIANR	4597	0.00	2	893.4215	1		0		no	9.90	432.58
YEELQQTAGR	812	7.90	14	1193.5690	2		0		yes	4.57	5725.32

Tags	
	Anova p-value ≤ 0.05
	power>0.8
	q<0.05

P07355

Annexin A2 OS=Homo sapiens OX=9606 GN=ANXA2 PE=1 SV=2
17 peptides

Sequence	Peptide Ion	Score	Hits	Mass	Charge	Tags	Conflicts	Modifications	In quantitation	Drift time (ms)	Average Normalised Abundances	
											CTR	MC
AEDGSVIDYELIDQDAR	8813	7.88	1	1907.8729	3		0		no	4.71	75.64	1
AEDGSVIDYELIDQDAR	6309	7.88	2	1907.8773	2		0		no	6.99	166.58	2
ASMKGLGTDEDSLIEIICSR	1196	5.39	1	2226.0422	3		0	[3] Oxidation M|[11] Hydroxyl DKNP|[18] Carbamidomethyl C	yes	5.33	514.68	420
ASMKGLGTDEDSLIEIICSR	1200	---	---	2226.0393	4		0	[3] Oxidation M|[11] Hydroxyl DKNP|[18] Carbamidomethyl C	yes	4.50	579.03	385
AYTNFDAER	3664	4.69	1	1085.4825	3		0		no	3.19	755.98	1
AYTNFDAER	2713	4.69	1	1085.4819	2		0		no	4.57	1307.65	10

Sequence	Peptide Ion	Score	Hits	Mass	Charge	Tags	Conflicts	Modifications	In quantitation	Drift time (ms)	Average Normalised Abundances	
											CTR	MC
AYTNFDAER	3379	7.49	2	1085.4906	2		0		no	4.43	815.43	37
AYTNFDAERDALNIETAIK	2066	7.10	2	2154.0530	3		0		no	4.92	511.81	11
DALNIETAIK	4057	7.57	2	1086.5748	2		0		no	4.36	395.41	13
DIISDTSGDFR	2811	6.18	2	1224.5671	2		0		no	4.71	687.45	45
DIISDTSGDFRK	1771	5.65	1	1352.6393	2		0		no	5.26	939.73	76
DLYDAGVK	12839	---	---	879.4344	1		0		no	9.63	12.29	
DLYDAGVK	2987	7.72	2	879.4348	2		0		no	3.81	360.97	15
GVDEVTIVNILTNR	1814	8.18	2	1541.8384	2		0		no	6.09	608.42	15
GVDEVTIVNILTNR	9293	8.18	1	1541.8492	3		0		no	4.09	54.46	
RAEDGSVIDYELIDQDAR	1779	8.55	4	2064.0096	3		0		no	5.12	919.04	171
SYSPYDMLESIR	5575	6.69	1	1475.6595	2		0	[6] Hydroxyl DKNP	no	5.75	124.81	
TDLEKDIISDTSGDFR	10530	8.33	2	1810.8450	2		0		no	6.72	32.10	
TDLEKDIISDTSGDFR	2758	8.33	2	1810.8588	3		0		no	4.22	429.62	7
TKGVDEVTIVNILTNR	6069	6.96	2	1770.9818	3		0		no	4.50	164.15	
TNQELQEINR	640	8.61	8	1243.6155	2		0		yes	4.71	3035.01	139
TNQELQEINRVYK	1494	4.18	1	1633.8314	3		0		yes	4.29	268.48	268
WISIMTER	1708	8.01	2	1050.5296	2		0	[5] Oxidation M	no	4.29	1474.51	83
YDAGVK	4129	0.00	1	651.3247	1		0		no	7.55	109.37	

Tags	
	Anova p-value ≤ 0.05
	power>0.8
	q<0.05

P11142

Heat shock cognate 71 kDa protein OS=Homo sapiens OX=9606 GN=HSPA8 PE=1 SV=1
20 peptides

Sequence	Peptide Ion	Score	Hits	Mass	Charge	Tags	Conflicts	Modifications	In quantitation	Drift time (ms)	Average Normalised Abundances	
											CTR	MCD
ARFEELNADLFR	9560	6.80	1	1479.7394	2		0		no	5.96	50.38	5.9
ARFEELNADLFR	4761	6.80	2	1479.7468	3		0		no	3.95	174.79	45.6
DAGTIAGLNVLR	1005	7.70	5	1198.6656	2		0		no	4.92	1268.30	656.4
EIAEAYLGK	2184	5.48	1	992.5158	2		0		no	4.09	767.00	757.3
ENKITITNDK	1665	4.66	1	1190.6188	2		0	[2] Hydroxyl DKNP	yes	4.85	1617.62	939.61
FEELNADLFR	6075	6.58	1	1252.6225	2		0		no	4.99	125.74	91.6
FELTGIPPAPR	2150	4.99	1	1196.6751	3		0		no	3.67	555.72	251.1
FELTGIPPAPR	4206	4.99	1	1196.6653	2		0		no	4.78	310.93	107.1
GTLDPVEK	3491	7.00	4	857.4404	2		0		no	3.67	487.45	450.2
HWPFMVVNDAGRPK	6925	5.39	1	1652.7915	2		0		no	6.23	98.48	18.8
IINEPTAAAIAYGLDK	8471	6.55	1	1658.8664	2		0		no	6.37	98.95	10.9

Sequence	Peptide Ion	Score	Hits	Mass	Charge	Tags	Conflicts	Modifications	In quantitation	Drift time (ms)	Average Normalised Abundances	
											CTR	MCD
IINEPTAAAIAYGLDKK	2741	7.41	4	1786.9813	3		0		no	4.92	683.19	197.26
IINEPTAAAIAYGLDKK	3807	6.33	3	1800.9844	3		0	[16] Methyl K	no	4.92	345.30	56.63
INDEDKQK	4545	6.47	1	1018.4845	2		0	[3] Hydroxyl DKNP|[6] Methyl K	no	4.02	418.44	119.05
NQVAMNPTNTVFDAK	2848	6.95	2	1664.7806	2		0	[6] Hydroxyl DKNP	no	6.37	377.50	147.67
NQVAMNPTNTVFDAKR	6555	5.22	1	1804.8862	3		0		no	4.64	194.27	35.20
NQVAMNPTNTVFDAKR	3936	6.13	1	1820.8825	3		0	[6] Hydroxyl DKNP	no	4.50	315.20	102.49
STAGDTHLGGEDFDNR	4868	6.10	1	1690.7237	3		0		no	3.95	229.80	112.91
TTPSYVAFTDTER	736	7.68	8	1486.6944	2		0		yes	5.75	2324.71	1855.08
TVTNAVVTVPAYFNDSQR	10534	7.07	1	1980.9817	2		0		no	7.41	89.27	0.00
VEIIANDQGNR	1620	7.72	5	1227.6196	2		0		yes	4.71	1194.60	905.12
VSSKNSLESYAFNMK	1590	5.40	1	1719.8145	3		0	[4] Hydroxyl DKNP	no	3.88	788.36	396.57

Tags	
	Anova p-value ≤ 0.05
	power>0.8
	q<0.05

P02533

Keratin_ type I cytoskeletal 14 OS=Homo sapiens OX=9606 GN=KRT14 PE=1 SV=4
16 peptides

Sequence	Peptide Ion	Score	Hits	Mass	Charge	Tags	Conflicts	Modifications	In quantitation	Drift time (ms)	Average Abunda
											CTR
ALEEANADLEVK	2852	6.77	3	1300.6466	2		0		no	5.40	1019.
APSTYGGGLSVSSSR	1279	8.43	8	1424.6868	2		0		no	5.40	1974.
ASLENSLEETKGR	853	7.59	6	1432.7156	3		0		no	3.81	2406.
DAEEWFFTK	1560	7.82	6	1171.5188	2		0		no	4.57	1754.
DAEEWFFTKTEELNR	3757	6.66	2	1913.8748	3		0		no	4.50	858.
EVATNSELVQSGK	741	8.33	12	1360.6841	2		0		yes	5.54	4068.
GSCGIGGGIGGGSSR	2851	7.75	3	1277.5905	2		0	[3] Carbamidomethyl C	no	4.92	853.
ILNEMRDQYEK	14252	---	---	1453.6883	2		0	[11] Hydroxyl DKNP	no	5.61	49.
ILNEMRDQYEK	4027	6.43	1	1453.6890	3		0	[11] Hydroxyl DKNP	no	3.67	3377.
ILNEMRDQYEK	1816	5.61	1	1453.6886	3		0	[11] Hydroxyl DKNP	no	3.67	1403.
ISSVLAGGSCR	1098	8.01	5	1105.5555	2		0	[10] Carbamidomethyl C	no	4.29	1433.
LLEGEDAHLSSSQFSSGSQSSR	1287	8.04	8	2308.0541	3		0		no	4.99	2377.
MSVEADINGLRR	2126	5.74	1	1375.6902	3		0	[1] Oxidation M	no	3.74	1593.
RLLEGEDAHLSSSQFSSGSQSSR	2975	4.89	1	2480.1258	4		0	[7] Hydroxyl DKNP	no	4.92	916.

Sequence	Peptide Ion	Score	Hits	Mass	Charge	Tags	Conflicts	Modifications	In quantitation	Drift time (ms)	Average Abunda
											CTR
RLLEGEDAHLSSSQFSSGSQSSR	5166	4.89	1	2480.1334	5		0	[7] Hydroxyl DKNP	no	4.09	473.
SEISELRR	4991	---	---	988.5306	3		0		yes	3.05	297.
SEISELRR	707	8.08	7	988.5284	2		0		yes	4.16	4405.
TEELNREVATNSELVQSGK	1503	7.58	3	2103.0405	3		0		no	5.26	1345.
TKYETELNLR	1162	7.39	6	1265.6601	2		0		no	5.19	2407.
TKYETELNLR	1397	7.39	5	1265.6622	3		0		no	3.67	1854.
VLDELTLAR	14914	---	---	1028.5847	1		0		yes	11.84	40.
VLDELTLAR	182	8.50	16	1028.5897	2		0		yes	4.29	1.03e+0

Tags	
	Anova p-value ≤ 0.05
	power>0.8
	q<0.05

P18206

Vinculin OS=Homo sapiens OX=9606 GN=VCL PE=1 SV=4
19 peptides

Sequence	Peptide Ion	Score	Hits	Mass	Charge	Tags	Conflicts	Modifications	In quantitation	Drift time (ms)	Average Normalis Abundan
											CTR
AGEVINQPMMMAAR	7471	6.43	2	1565.6946	2		0	[8] Hydroxyl DKNP|[9] Oxidation M|[10] Oxidation M	no	5.89	139.87
ALASIDSK	4768	6.55	1	803.4356	2		0		no	3.46	106.29
ALASQLQDSLK	2937	6.46	2	1172.6324	2		0		no	5.06	308.59
AQQVSQGLDVLTAK	2938	7.19	2	1456.7809	2		0		no	5.89	480.30
AVAGNISDPGLQK	2028	6.48	2	1268.6671	2		0		no	5.12	416.14
DPSASPGDAGEQAIR	11446	6.06	1	1469.6683	2		0		no	5.40	35.14
ELTPQVVSAAR	1366	6.92	2	1169.6358	2		0		yes	4.64	787.86
EVAKQCTDK	2917	5.43	1	1091.5322	2		0	[6] Carbamidomethyl C|[9] Methyl K	no	4.43	816.80
EVENSEDPKFR	8319	5.94	1	1348.6226	3		0		no	3.81	92.61
GWLRDPSASPGDAGEQAIR	4017	6.79	2	1981.9555	3		0		no	4.16	322.62
LLAVAATAPPDAPNREEVFDER	2275	6.81	2	2380.2032	3		0		no	5.12	754.78
MTGLVDEAIDTK	3898	6.89	2	1307.6299	2		0	[1] Oxidation M	no	5.06	433.03
QILDEAGK	4187	4.83	1	872.4493	2		0		no	3.81	351.58
SLGEISALTSK	284	5.65	1	1104.5943	2		0		yes	4.50	3460.52
SLLDASEEAIKK	3883	6.04	1	1302.7006	3		0		no	3.60	169.08
SLLDASEEAIKK	5416	6.04	1	1302.6881	2		0		no	5.47	154.49
STVEGIQASVK	3153	6.99	2	1117.5864	2		0		no	4.50	428.40
TDAGFTLR	3458	6.74	2	879.4477	2		0		no	3.74	235.53
TNLLQVCER	3522	5.80	1	1131.5590	2		0		no	4.57	292.19

Sequence	Peptide Ion	Score	Hits	Mass	Charge	Tags	Conflicts	Modifications	In quantitation	Drift time (ms)	Average Normalis Abundan
											CTR
								[7] Carbamidomethyl C			
VMLVNSMNTVK	246	5.22	1	1248.6599	2		0	[11] Methyl K	yes	5.12	3348.13

Tags
Anova p-value ≤ 0.05
power>0.8
q<0.05

P12883

Myosin-7 OS=Homo sapiens OX=9606 GN=MYH7 PE=1 SV=5
18 peptides

Sequence	Peptide Ion	Score	Hits	Mass	Charge	Tags	Conflicts	Modifications	In quantitation	Drift time (ms)	Average Abundan
											CTR
DTQIQLDDAVR	4668	6.64	1	1272.6294	2		0		no	5.26	104.5
IEDEQALGSQLQK	5833	6.33	2	1457.7377	2		0		no	5.61	60.9
IEDEQALGSQLQKK	6942	5.44	1	1585.8278	3		0		no	4.36	2.9
IEELEEELEAER	4616	7.04	2	1487.7001	2		0		no	5.75	52.9
ILNPAAIPEGQFIDSR	2181	7.47	2	1739.9151	2		0		no	6.79	0.3
ILNPAAIPEGQFIDSR	5788	7.47	1	1739.9155	3		0		no	4.22	1.1
ILYGDFR	3854	6.75	2	882.4614	2		0		no	4.09	17.6
INATLETK	4303	6.63	2	888.4858	2		0		no	3.81	68.5
IQAQSR	367	3.91	1	701.3718	1		0		no	7.89	1670.9
+KGSSFQTVSALHR	103	4.19	1	1444.7357	2		0	[N-term] Formyl K N-TERM	yes	5.68	3.04e+00
+KGSSFQTVSALHR	11272	---	---	1444.7285	3		0	[N-term] Formyl K N-TERM	yes	3.95	42.7
+KGSSFQTVSALHR	306	5.07	1	1444.7336	2		0	[N-term] Formyl K N-TERM	yes	5.68	1.28e+00
KMDADLSQLQTEVEEAVQECR	2880	---	---	2492.1271	5		0	[1] Methyl K|[20] Carbamidomethyl C	yes	4.22	493.8
KMDADLSQLQTEVEEAVQECR	1144	5.14	1	2492.1245	4		0	[1] Methyl K|[20] Carbamidomethyl C	yes	4.85	2676.5
LAEQELIETSER	3372	7.13	2	1416.7074	2		0		no	5.40	40.0
LQEAEEAVEAVNAK	6090	6.48	1	1499.7418	2		0		no	5.82	4.1
LQQFFNHHMFVLEQEEYKK	2636	4.87	1	2494.2579	3		0		no	6.16	121.6
NKDPLNETVVGLYQK	1743	4.10	1	1716.8979	2		0		no	6.37	515.7
QLDEKEALISQLTR	4423	6.11	2	1642.8784	3		0		no	4.29	14.6
SLSTELFK	3694	6.27	2	923.4967	2		0		no	4.02	11.8
SVNDLTSQR	3673	7.01	2	1018.5000	2		0		no	4.16	247.5
VVDSLQTSLDAETR	5227	7.33	2	1532.7660	2		0		no	5.96	22.8

Tags
Anova p-value ≤ 0.05

	power>0.8
	q<0.05

P14543

Nidogen-1 OS=Homo sapiens OX=9606 GN=NID1 PE=1 SV=3
21 peptides

Sequence	Peptide Ion	Score	Hits	Mass	Charge	Tags	Conflicts	Modifications	In quantitation	Drift time (ms)	Average Normalised Abundances	
											CTR	MCD
AECLNPSQPSR	5385	6.93	2	1257.5786	2		0	[3] Carbamidomethyl C	no	4.85	244.64	105
AECLNPSQPSRR	5172	5.83	1	1413.6853	3		0	[3] Carbamidomethyl C	no	3.74	166.74	7
ALEGLQYPFAVTSYGK	8722	6.14	2	1742.8744	2		0		no	6.44	55.44	54
ASLHGGEPTTIIR	3222	5.60	1	1350.7107	3		0		no	3.95	254.13	393
CVPGEVEK	4805	5.76	1	916.4395	2		0	[1] Carbamidomethyl C	no	4.16	240.26	51
EDLSPSITQR	3486	6.71	2	1144.5657	2		0		no	4.57	1009.04	412
EYTVTEPER	4573	4.97	1	1122.5300	2		0		no	4.43	218.89	46
EYTVTEPERDGASPSR	2413	6.92	2	1792.8211	3		0		no	4.36	728.25	888
GNLYWTDWNRDNPK	6000	5.53	1	1777.8143	3		0		no	4.16	139.90	26
GNLYWTDWNRDNPK	131	5.03	1	1793.8105	2		0	[9] Hydroxyl DKNP	yes	6.37	4196.91	5.44e+
GNLYWTDWNRDNPK	946	---	---	1793.8080	3		0	[9] Hydroxyl DKNP	yes	4.43	363.03	5315
LASSSR	5523	4.81	1	619.3191	1		0		no	7.06	68.65	9
NIFWTDSNLDRIEVAK	4095	5.90	1	1919.9692	3		0		no	4.78	241.01	68
QAEVTFVGHPGNLVIK	4498	5.47	2	1707.9206	3		0		no	4.57	182.13	91
QCVAEGSPQR	5430	6.38	1	1130.5153	2		0	[2] Carbamidomethyl C	no	4.36	62.32	31
QDLGSPEGIAVDHLGR	1998	6.51	2	1662.8289	3		0		no	4.57	450.03	485
RVLFETDLVNPR	6145	5.46	1	1457.7942	3		0		no	4.02	85.96	67
TDSVR	2824	0.00	1	576.2860	1		0		no	6.65	213.52	155
TQFTCECSIGFR	6647	6.56	2	1504.6528	2		0	[5] Carbamidomethyl C|[7] Carbamidomethyl C	no	5.61	171.37	69
VLFETDLVNPR	446	6.79	2	1301.6978	2		0		yes	5.40	2270.48	4472
VTDSVR	9501	---	---	675.3538	2		0		no	3.32	23.46	6
VTDSVR	2081	0.00	1	675.3544	1		0		no	7.89	280.04	235
YALSNSIGPVR	1475	7.22	2	1175.6257	2		0		yes	4.64	1050.40	869

Tags	
	Anova p-value ≤ 0.05
	power>0.8
	q<0.05

P08572

Collagen alpha-2(IV) chain OS=Homo sapiens OX=9606 GN=COL4A2 PE=1 SV=4
18 peptides

Sequence	Peptide Ion	Score	Hits	Mass	Charge	Tags	Conflicts	Modifications	In quantitation	Drift time (ms)	Aver Norr Abur
											CTR
AHNQDLGLAGSCLAR	2794	6.51	2	1581.7729	3		0	[12] Carbamidomethyl C	no	4.22	386
AHNQDLGLAGSCLAR	13789	6.51	1	1581.7617	2		0	[12] Carbamidomethyl C	no	6.16	16
ATPFIECNGGR	2971	6.67	2	1220.5668	2		0	[7] Carbamidomethyl C	no	4.71	433
EGFPGPPGFIGPR	1020	5.50	1	1358.6618	2		0	[4] Hydroxyl DKNP|[6] Hydroxyl DKNP	no	5.06	1667
EGFPGPPGFIGPR	2700	6.14	1	1374.6572	2		0	[4] Hydroxyl DKNP|[6] Hydroxyl DKNP|[7] Hydroxyl DKNP	no	5.12	464
FDVPCGGR	2209	5.76	1	906.4166	2		0	[5] Carbamidomethyl C	no	3.88	786
FSTMPFLYCNPGDVCYYASR	11499	---	---	2463.0511	2		0	[9] Carbamidomethyl C|[10] Hydroxyl DKNP|[15] Carbamidomethyl C	no	8.80	39
FSTMPFLYCNPGDVCYYASR	5419	5.56	1	2463.0334	3		0	[9] Carbamidomethyl C|[10] Hydroxyl DKNP|[15] Carbamidomethyl C	no	5.06	180
GDPGDTGAPGPVGMK	830	4.43	1	1370.5968	2		0	[5] Hydroxyl DKNP	yes	5.19	3134
GEQGFMGNTGPTGAVGDRGPK	4540	5.52	1	2047.9327	3		0	[8] Hydroxyl DKNP	no	4.43	251
GLDGYQGPDGPR	1174	6.84	2	1230.5682	2		0		yes	4.71	2767
GLPGEVLGAQPGPR	4485	5.62	2	1346.7095	2		0		no	5.12	209
GLPGEVLGAQPGPR	3879	6.16	2	1362.7194	2		0	[11] Hydroxyl DKNP	no	5.19	254
GLPGEVLGAQPGPR	767	6.50	2	1378.7189	2		0	[3] Hydroxyl DKNP|[11] Hydroxyl DKNP	no	5.19	1344
GPRGEQGFMGNTGPTGAVGDR	341	4.59	1	2059.9466	2		0		yes	7.41	3766
GVSGFPGADGIPGHPGQGGPR	12138	4.89	1	1931.9517	2		0	[9] Hydroxyl DKNP	no	7.48	52
GVSGFPGADGIPGHPGQGGPR	1379	6.25	1	1963.9110	3		0	[9] Hydroxyl DKNP|[12] Hydroxyl DKNP|[15] Hydroxyl DKNP	no	4.16	1203
IAVQPGTVGPQGR	566	7.15	2	1278.7038	2		0		no	4.92	1480
SVSIGYLLVK	3315	6.62	2	1077.6437	2		0		no	4.57	277
SYWLSTTAPLPMMPVAEDEIKPYISR	11163	5.26	1	3026.4439	3		0		no	6.09	58

Sequence	Peptide Ion	Score	Hits	Mass	Charge	Tags	Conflicts	Modifications	In quantitation	Drift time (ms)	Aver Norr Abur
											CTR
								[11] Hydroxyl DKNP|[14] Hydroxyl DKNP			

Tags	
	Anova p-value ≤ 0.05
	power>0.8
	q<0.05

P02538

Keratin_ type II cytoskeletal 6A OS=Homo sapiens OX=9606 GN=KRT6A PE=1 SV=3
12 peptides

Sequence	Peptide Ion	Score	Hits	Mass	Charge	Tags	Conflicts	Modifications	In quantitation	Drift time (ms)	Average Normalised Abundances	
											CTR	MCD
ADTLTDEINFLR	571	8.23	16	1406.7042	2		0		no	5.54	3915.82	37
AIGGGLSSVGGGSSTIK	1319	7.31	9	1446.7603	2		0		no	5.61	1012.24	14
GEMALKDAK	2409	6.30	1	993.4826	2		0	[7] Hydroxyl DKNP|[9] Hydroxyl DKNP	no	4.22	1747.15	6
ISIGGGSCAISGGYGSR	3837	6.89	2	1597.7466	2		0	[8] Carbamidomethyl C	no	5.96	448.33	5
ISIGGGSCAISGGYGSR	6135	5.94	1	1597.7682	3		0	[8] Carbamidomethyl C	no	4.02	122.38	
LEGLEDALQK	1321	5.91	1	1114.5882	2		0		no	4.50	2984.82	4
NTKQEIAEINR	1530	6.19	1	1330.6882	3		0	[1] Hydroxyl DKNP	no	3.74	580.86	5
RTAAENEFVTLK	377	6.04	1	1393.7116	3		0	[6] Hydroxyl DKNP	yes	3.81	6210.25	46
RTAAENEFVTLK	2142	6.04	1	1393.7051	2		0	[6] Hydroxyl DKNP	yes	5.54	957.68	4
SGFSSVSVSR	1416	7.44	5	1011.4975	2		0		no	4.09	1166.08	11
TAAENEFVTLK	2516	5.43	1	1221.6108	2		0		no	4.85	741.90	4
TAAENEFVTLKK	540	8.05	11	1349.7200	3		0		yes	3.46	2992.63	40
TAAENEFVTLKK	2995	8.05	3	1349.7163	2		0		yes	5.47	362.89	4
VELQAKADTLTDEINFLR	219	4.82	2	2075.0856	3		0		yes	5.68	6061.02	2.27
VELQAKADTLTDEINFLR	461	4.82	2	2075.0856	4		0		yes	4.78	2509.05	93
YEELQVTAGR	798	7.57	10	1164.5854	2		0		no	4.78	3599.80	26

Tags	
	Anova p-value ≤ 0.05
	power>0.8
	q<0.05

P00738

Haptoglobin OS=Homo sapiens OX=9606 GN=HP PE=1 SV=1
16 peptides

Sequence	Peptide Ion	Score	Hits	Mass	Charge	Tags	Conflicts	Modifications	In quantitation	Drift time (ms)	Average Normalised Abundance	
											CTR	[illegible]
AVGDKLPECEAVCGKPK	2978	6.86	2	1856.9182	4		0	[9] Carbamidomethyl C|[13] Carbamidomethyl C	no	3.88	322.61	[illegible]
DIAPTLTLYVGK	7169	6.28	2	1289.7133	2		0		no	5.19	179.33	
GSFPWQAK	6943	6.71	1	919.4584	2		0		no	3.74	32.91	
HYEGSTVPEK	5502	7.08	1	1145.5334	2		0		no	4.57	75.72	
+KQLVEIEK	2596	6.98	2	1013.5726	2		0	[N-term] Formyl K N-TERM	no	4.50	327.92	[illegible]
LNNEK	8622	---	---	616.3169	2		0		yes	3.12	30.60	
LNNEK	1079	0.00	1	616.3193	1		0		yes	7.13	459.15	[illegible]
LRTEGDGVYTLNNEK	1643	6.66	2	1707.8351	3		0		no	4.16	429.80	[illegible]
LRTEGDGVYTLNNEK	14550	---	---	1707.8278	2		0		no	6.44	15.91	
NPANPVQR	4925	6.44	1	894.4583	2		0		no	3.88	130.26	
QLVEIEK	2384	6.57	2	857.4852	2		0		no	4.09	194.60	[illegible]
SPVGVQPILNEHTFCAGMSK	4016	6.33	2	2187.0455	3		0	[7] Hydroxyl DKNP|[15] Carbamidomethyl C	no	5.12	193.35	[illegible]
TEGDGVYTLNDK	4934	7.05	1	1310.6034	2		0		no	5.12	193.59	[illegible]
TEGDGVYTLNDKK	5519	7.10	2	1438.6969	3		0		no	3.95	133.79	
TEGDGVYTLNNEK	1945	7.89	2	1438.6591	2		0		yes	5.54	546.59	[illegible]
VMPICLPSK	3551	6.70	1	1059.5433	2		0	[2] Oxidation M|[5] Carbamidomethyl C	no	4.50	351.66	[illegible]
VMPICLPSKDYAEVGR	864	7.54	2	1849.9072	3		0	[2] Oxidation M|[5] Carbamidomethyl C	yes	4.85	1555.00	[illegible]
YVMLPVADQDQCIR	4109	7.20	2	1722.8001	2		0	[3] Oxidation M|[12] Carbamidomethyl C	no	6.44	286.22	[illegible]

Tags	
	Anova p-value ≤ 0.05
	power>0.8
	q<0.05

Q96JY6

PDZ and LIM domain protein 2 OS=Homo sapiens OX=9606 GN=PDLIM2 PE=1 SV=1
13 peptides

Sequence	Peptide Ion	Score	Hits	Mass	Charge	Tags	Conflicts	Modifications	In quantitation	Drift time (ms)	Average Normalise Abundanc
											CTR
AGDSAVLVLPPSPGPR	2130	7.60	2	1531.8273	2		0		no	5.96	1122.72
AGSPFSPPPSSSSLTGEAAISR	6994	7.15	1	2102.0193	2		0		no	7.62	102.20
AGSPFSPPPSSSSLTGEAAISR	7132	7.15	1	2102.0150	3		0		no	5.12	104.57
CSTSIANQAVR	4550	7.30	2	1205.5784	2		0	[1] Carbamidomethyl C	no	4.71	147.41
DFHTPIMVTKVAER	7460	6.05	1	1642.8470	3		0		no	4.02	100.58
GGTPAFLPSSLSPQSSLPASR	4044	7.85	2	2056.0517	3		0		no	4.64	172.46
GGTPAFLPSSLSPQSSLPASR	4492	7.85	2	2056.0491	2		0		no	7.69	220.50
GHFWVGDELYCEK	6091	5.05	1	1654.7158	3		0	[7] Hydroxyl DKNP|[11] Carbamidomethyl C	no	3.95	327.35
LLQEALEAEER	715	8.71	7	1299.6651	2		0		yes	5.19	1930.85
LQLDR	4255	7.16	1	643.3649	2		0		no	3.19	118.48
LQLDR	6591	---	---	643.3575	1		0		no	7.76	37.15
LSYSGRPGSR	6319	---	---	1078.5519	1		0		no	11.91	42.63
SFQSLACSPGLPAADR	5085	7.19	2	1675.7953	2		0	[7] Carbamidomethyl C	no	6.37	81.09
SSYSSPTSLSPR	1371	8.00	6	1267.6132	2		0		no	4.99	985.96
TYTESQSSLR	1455	8.16	6	1170.5497	2		0		yes	4.64	1763.74
YSAPATLSSR	1526	7.92	5	1051.5287	2		0		yes	4.22	1008.94

Tags	
	Anova p-value ≤ 0.05
	power>0.8
	q<0.05

P33778

Histone H2B type 1-B OS=Homo sapiens OX=9606 GN=H2BC3 PE=1 SV=2
13 peptides

Sequence	Peptide Ion	Score	Hits	Mass	Charge	Tags	Conflicts	Modifications	In quantitation	Drift time (ms)	Average Normalised Abundances	
											CTR	MCD
AMGIMNSFVNDIFER	3284	8.00	1	1774.8015	2		0	[2] Oxidation M|[5] Oxidation M	no	6.72	279.35	18.4
AMGIMNSFVNDIFER	3122	6.26	1	1774.7991	2		0	[2] Oxidation M|[6] Hydroxyl DKNP	no	6.65	1122.48	615.2
EIQTAVR	432	8.42	12	815.4507	2		0		yes	3.53	5046.49	5616.3
EIQTAVR	1757	8.42	1	815.4506	1		0		yes	8.93	402.54	254.6
EIQTAVR+	1348	0.00	3	797.4385	2		0	[C-term] Dehydrated	no	3.46	1001.98	874.8
ESYSIYVYK	3767	6.78	2	1150.5530	2		0		no	4.78	348.35	203.7
IAGEASRLAHYNK	4	5.65	3	1428.7500	2		0		yes	5.61	1.06e+005	3.20e+00
IAGEASRLAHYNK	6049	5.52	1	1458.7519	2		0		no	5.54	295.42	37.0

Sequence	Peptide Ion	Score	Hits	Mass	Charge	Tags	Conflicts	Modifications	In quantitation	Drift time (ms)	Average Normalised Abundances	
											CTR	MCD
								[12] Hydroxyl DKNP|[13] Methyl K				
LLLPGELAK	3725	8.59	2	952.5944	1		0		no	11.70	130.90	42.0
LLLPGELAK	90	8.59	15	952.6019	2		0		yes	4.22	1.80e+004	2.09e+00
LLLPGELAK	4563	6.67	1	966.6097	2		0	[9] Methyl K	no	4.22	92.24	189.3
QTAVR	682	0.00	3	573.3244	1		0		no	6.58	1730.83	1566.9
QTAVR	4246	---	---	573.3238	2		0		no	3.12	117.90	42.1
QVHPDTGISSK	1574	8.00	4	1167.5880	3		0		no	3.60	448.00	245.9
QVHPDTGISSK	4126	8.00	1	1167.5883	2		0		no	4.71	166.97	85.0
QVHPDTGISSK	6443	4.74	1	1167.5851	3		0		no	3.60	25.51	332.7
QVHPDTGISSK	15224	5.73	1	1183.5843	2		0	[5] Hydroxyl DKNP	no	4.78	213.16	52.0
STITSR	971	7.48	1	663.3556	1		0		no	7.69	530.57	283.9
STITSR	8653	---	---	663.3559	2		0		no	3.12	22.79	0.9

Tags	
	Anova p-value ≤ 0.05
	power>0.8
	q<0.05

P68363

Tubulin alpha-1B chain OS=Homo sapiens OX=9606 GN=TUBA1B PE=1 SV=1
12 peptides

Sequence	Peptide Ion	Score	Hits	Mass	Charge	Tags	Conflicts	Modifications	In quantitation	Drift time (ms)	Average Normalised Abundanc
											CTR
AVFVDLEPTVIDEVR	495	8.69	9	1700.8969	2		0		yes	6.58	3158.47
AVFVDLEPTVIDEVR	2754	8.69	3	1700.8988	3		0		yes	4.50	415.18
DVNAAIATIK	661	8.64	4	1014.5670	2		0		yes	4.29	2176.40
IHFPLATYAPVISAEK	4830	7.07	2	1755.9542	3		0		no	4.78	437.22
LSVDYGK	3421	6.59	1	780.3994	2		0		no	3.53	196.23
LSVDYGKK	3520	7.51	2	908.4912	2		0		no	3.95	299.18
NLDIERPTYTNLNR	7702	6.07	1	1717.8667	3		0		no	4.22	73.84
QLFHPEQLITGK	7925	6.01	1	1409.7622	3		0		no	4.09	60.10
QLFHPEQLITGKEDAANNYAR	3860	6.79	2	2414.1878	4		0		no	4.85	382.31
SIQFVDWCPTGFK	10980	7.60	2	1583.7464	2		0	[8] Carbamidomethyl C	no	5.89	60.06
TGTYRQLFHPEQLITGK	602	5.04	2	1988.0685	3		0		yes	4.57	1381.79
TGTYRQLFHPEQLITGK	3104	5.04	2	1988.0634	3		0		no	5.47	95.93
TIGGGDDSFNTFFSETGAGK	4168	7.59	2	2006.8807	2		0		no	7.06	356.61
VGINYQPPTVVPGGDLAK	2144	7.76	5	1823.9720	2		0		no	6.79	848.75
VGINYQPPTVVPGGDLAK	3920	7.76	2	1823.9703	3		0		no	4.71	210.02

Tags	
	Anova p-value ≤ 0.05
	power>0.8
	q<0.05

P26038

Moesin OS=Homo sapiens OX=9606 GN=MSN PE=1 SV=3
16 peptides

Sequence	Peptide Ion	Score	Hits	Mass	Charge	Tags	Conflicts	Modifications	In quantitation	Drift time (ms)	Average Normalised Abundances	
											CTR	MCD
ALELEQER	750	6.70	2	986.5033	2		0		yes	4.22	4381.75	2595.6
ALTSELANARDESK	3650	5.87	1	1517.7682	3		0	[14] Methyl K	no	4.09	491.12	0.2
APDFVFYAPR	1948	7.32	3	1181.5876	2		0		no	4.85	636.30	255.7
AQMVQEDLEK	4547	6.04	2	1205.5612	2		0	[3] Oxidation M	no	5.06	334.24	49.3
AQMVQEDLEKTR	6842	5.82	1	1462.7080	3		0	[3] Oxidation M	no	3.88	95.86	15.1
AQQELEEQTR	9545	6.36	1	1230.5830	2		0		no	4.78	69.84	1.3
AQQELEEQTRR	2159	6.45	2	1386.6825	3		0		no	3.60	438.36	105.1
EDAVLEYLK	117	5.33	1	1078.5546	1		0		yes	11.98	3907.25	1.85e+00
ESEAVEWQQK	3972	6.05	2	1232.5744	2		0		no	4.71	442.51	226.6
IGFPWSEIR	3680	6.95	2	1103.5776	2		0		no	4.57	289.92	65.6
ISQLEMAR	1559	6.47	2	962.4801	2		0	[6] Oxidation M	yes	4.02	2682.65	683.7
KTQEQLALEMAELTAR	5129	5.78	1	1846.9354	3		0	[10] Oxidation M	no	4.43	148.45	21.6
QLFDQVVK	3002	5.67	2	975.5385	2		0		no	4.22	416.61	142.4
QRIDEFESM	5582	5.91	2	1153.5188	2		0		no	4.43	246.87	65.6
QRIDEFESM	2468	6.52	3	1169.5060	2		0	[9] Oxidation M	no	4.57	648.94	233.1
RKPDTIEVQQMK	4229	5.97	2	1487.7746	3		0	[12] Hydroxyl DKNP	no	3.81	324.39	14.1

Tags	
	Anova p-value ≤ 0.05
	power>0.8
	q<0.05

P01024

Complement C3 OS=Homo sapiens OX=9606 GN=C3 PE=1 SV=2
17 peptides

Sequence	Peptide Ion	Score	Hits	Mass	Charge	Tags	Conflicts	Modifications	In quantitation	Drift time (ms)	Averag Normal Abunda
											CTR
AAVYHHFISDGVR	4260	4.67	1	1470.7334	2		0		no	5.68	333.21

Sequence	Peptide Ion	Score	Hits	Mass	Charge	Tags	Conflicts	Modifications	In quantitation	Drift time (ms)	Averag Normal Abunda
											CTR
AGDFLEANYMNLQR	3925	6.02	1	1656.7567	2		0	[11] Hydroxyl DKNP	no	6.23	345.08
AGDFLEANYMNLQR	9587	---	---	1656.7577	3		0	[11] Hydroxyl DKNP	no	3.95	56.95
CAEENCFIQK	9258	5.69	1	1297.5510	2		0	[1] Carbamidomethyl C|[6] Carbamidomethyl C	no	4.99	96.04
DICEEQVNSLPGSITK	8576	5.99	2	1788.8475	2		0	[3] Carbamidomethyl C	no	6.65	131.35
DQLTCNKFDLK	10282	5.41	1	1380.6686	2		0	[5] Carbamidomethyl C	no	5.26	131.12
DSCVGSLVVK	4723	5.80	1	1062.5279	2		0	[3] Carbamidomethyl C	no	4.43	179.32
FYYIYNEK	1642	5.28	1	1138.5363	2		0		yes	4.71	1311.43
ILLQGTPVAQMTEDAVDAER	6337	5.76	2	2172.0636	2		0	[14] Hydroxyl DKNP	no	7.76	170.90
IPIEDGSGEVVLSR	2924	6.33	2	1469.7647	2		0		no	5.61	786.95
IPIEDGSGEVVLSRK	2328	5.64	1	1597.8573	3		0		no	4.02	580.70
ISLPESLK	3509	5.76	2	885.5156	2		0		no	3.88	202.25
NEQVEIR	2722	6.37	2	886.4547	2		0		no	3.95	713.22
NTMILEICTR	878	5.69	2	1265.6147	2		0	[1] Hydroxyl DKNP|[8] Carbamidomethyl C	yes	4.99	3678.50
QGALELIK	546	5.46	2	870.5176	2		0		yes	3.88	1683.30
SGSDEVQVGQQR	2873	7.08	2	1288.6011	2		0		no	4.85	406.06
TFISPIK	6108	6.05	1	804.4709	2		0		no	3.67	96.61
VPVAVQGEDTVQSLTQGDGVAK	8916	5.59	2	2197.1057	3		0		no	5.26	131.15

Tags	
	Anova p-value ≤ 0.05
	power>0.8
	q<0.05

P53420

Collagen alpha-4(IV) chain OS=Homo sapiens OX=9606 GN=COL4A4 PE=1 SV=3
18 peptides

Sequence	Peptide Ion	Score	Hits	Mass	Charge	Tags	Conflicts	Modifications	In quantitation	Drift time (ms)	Averag Norma Abunc
											CTR
AAPFLECQGR	525	7.01	2	1147.5475	2		0	[7] Carbamidomethyl C	yes	4.50	3698.1
ADLQFSSAPAPDTLK	4767	6.03	2	1559.7805	2		0		no	6.16	180.4
ADLQFSSAPAPDTLK	3973	5.32	1	1575.7699	2		0	[9] Hydroxyl DKNP	no	6.16	106.7
ADLQFSSAPAPDTLKESQAQR	10659	4.96	1	2275.0846	3		0		no	5.06	42.7

Sequence	Peptide Ion	Score	Hits	Mass	Charge	Tags	Conflicts	Modifications	In quantitation	Drift time (ms)	Averag Norma Abund
											CTR
								[9] Hydroxyl DKNP			
AGPVGPPGLGFPGPPGER	3511	4.78	1	1673.8326	3		0	[12] Hydroxyl DKNP	no	4.22	337.0
GDPGFPGGR	2380	6.10	1	890.3914	2		0	[3] Hydroxyl DKNP|[6] Hydroxyl DKNP	no	3.53	541.7
GDPGFPGGR	10698	---	---	890.3843	1		0	[3] Hydroxyl DKNP|[6] Hydroxyl DKNP	no	9.00	24.3
GDPGSPGISPPGPR	3452	5.43	2	1321.6230	2		0	[2] Hydroxyl DKNP|[3] Hydroxyl DKNP	no	4.85	355.3
GDPGSPGISPPGPR	1141	5.92	1	1337.6192	2		0	[3] Hydroxyl DKNP|[6] Hydroxyl DKNP|[10] Hydroxyl DKNP	no	4.99	1195.9
GEPGPPADVDDCPR	10330	3.83	1	1480.6151	3		0	[12] Carbamidomethyl C	no	3.95	27.4
GLPGFPGFPGER	1042	5.79	2	1277.6053	2		0	[3] Hydroxyl DKNP|[6] Hydroxyl DKNP|[9] Hydroxyl DKNP	yes	4.64	4090.0
GPEGAMGLPGMR	1629	6.31	2	1219.5329	2		0	[6] Oxidation M|[9] Hydroxyl DKNP|[11] Oxidation M	no	4.57	1427.5
GPTGLPGPR	5781	5.45	1	866.4505	2		0	[6] Hydroxyl DKNP	no	3.60	369.5
GPTGLPGPRGEPGPPADVDDCPR	366	4.33	1	2313.0780	4		0	[21] Carbamidomethyl C	yes	4.64	2726.2
GPTGLPGPRGEPGPPADVDDCPR	4879	---	---	2313.0680	3		0	[21] Carbamidomethyl C	yes	5.26	61.6
GPTGLPGPRGEPGPPADVDDCPR	1432	---	---	2313.0702	5		0	[21] Carbamidomethyl C	yes	3.95	592.7
GQKGDTISCNVTYPGR	10602	4.84	1	1767.8206	2		0	[9] Carbamidomethyl C|[14] Hydroxyl DKNP	no	6.51	71.8
GVDGVPGSPGPPGR	3194	5.72	1	1295.6079	2		0	[6] Hydroxyl DKNP|[9] Hydroxyl DKNP|[11] Hydroxyl DKNP	no	4.85	493.7
IPGLPGAPGMR	1270	5.75	2	1112.5707	2		0	[5] Hydroxyl DKNP|[8] Hydroxyl DKNP|[10] Oxidation M	no	4.29	2317.9
LWTGYSLLYLEGQEK	9963	5.34	1	1798.9081	2		0		no	6.93	56.2
PGFPGGR	9648	---	---	718.3395	1		0	[1] Hydroxyl DKNP|[4] Hydroxyl DKNP	no	7.89	33.5
PGFPGGR	2748	0.00	1	718.3398	2		0	[1] Hydroxyl DKNP|[4] Hydroxyl DKNP	no	3.25	318.1

Tags	
	Anova p-value ≤ 0.05
	power>0.8

	q<0.05

P69905

Hemoglobin subunit alpha OS=Homo sapiens OX=9606 GN=HBA1 PE=1 SV=2
17 peptides

Sequence	Peptide Ion	Score	Hits	Mass	Charge	Tags	Conflicts	Modifications	In quantitation	Drift time (ms)	Av Ab CT
DPVNFK	2635	---	---	718.3646	2		0		no	3.25	
DPVNFK	223	0.00	1	718.3662	1		0		no	8.10	
LRVDPVNFK	5596	---	---	1086.6152	3		0		no	3.32	
LRVDPVNFK	1155	7.48	5	1086.6118	2		0		no	4.78	
MFLSFPTTK	115	8.41	10	1086.5495	2		0	[1] Oxidation M	yes	4.43	2.
MFLSFPTTK	5036	8.41	1	1086.5412	1		0	[1] Oxidation M	no	12.12	
MFLSFPTTK	2336	7.12	4	1100.5582	2		0	[1] Oxidation M|[9] Methyl K	no	4.43	
PVNFK	149	0.00	1	603.3376	1		0		yes	6.65	2.
PVNFK	119	0.00	4	603.3396	1		0		no	7.13	
PVNFK	472	0.00	1	603.3400	2		0		no	2.98	
SFPTTK	2197	0.00	1	679.3553	1		0		no	7.62	
TNVKAAWGK	2681	6.25	3	973.5259	2		0		no	4.16	
TYFPHFDLSHGSAQVK	1844	6.02	3	1832.8869	4		0		no	4.02	
TYFPHFDLSHGSAQVK	3075	6.02	2	1832.8859	3		0		no	4.85	
TYFPHFDLSHGSAQVKGHGK	533	4.19	1	2212.0790	3		0		no	4.85	1.
VADALTNAVAHVDDMPNALSALSDLHAHK	6206	5.48	1	3011.4797	5		0	[14] Hydroxyl DKNP	no	4.64	
VDPVNFK	778	8.01	4	817.4335	1		0		no	9.35	
VDPVNFK	168	8.01	12	817.4383	2		0		no	3.46	
VGAHAGEYG	2392	0.00	2	841.3708	1		0		no	9.14	
VGAHAGEYG	5145	---	---	841.3721	2		0		no	3.46	
VGAHAGEYGA	3080	0.00	1	912.4068	1		0		no	9.83	
VGAHAGEYGA	2704	0.00	1	912.4150	2		0		no	3.74	
VGAHAGEYGAE	1719	0.00	1	1041.4567	2		0		no	4.02	
VGAHAGEYGAEA	2559	0.00	1	1112.5053	2		0		no	4.29	
VGAHAGEYGAEALER	775	8.18	6	1528.7255	2		0		yes	5.82	
VGAHAGEYGAEALER	127	8.18	12	1528.7329	3		0		yes	3.95	1.
VGAHAGEYGAEALER+	1956	0.00	1	1510.7147	3		0	[C-term] Dehydrated	no	4.16	

Tags	
	Anova p-value ≤ 0.05
	power>0.8
	q<0.05

P11021

Endoplasmic reticulum chaperone BiP OS=Homo sapiens OX=9606 GN=HSPA5 PE=1 SV=2
15 peptides

Sequence	Peptide Ion	Score	Hits	Mass	Charge	Tags	Conflicts	Modifications	In quantitation	Drift time (ms)	Average Normalised Abundances	
											CTR	MCD
DAGTIAGLNVMR	2343	6.38	2	1232.6153	2		0	[9] Hydroxyl DKNP	no	4.92	919.87	385.56
EFFNGKEPSR	2177	4.67	1	1209.5801	2		0		no	4.78	719.21	1411.35
ELEEIVQPIISK	6033	6.37	1	1396.7579	2		0		no	5.54	88.59	87.49
FEELNMDLFRSTMKPVQK	967	5.00	1	2212.0776	3		0		yes	4.78	6807.30	1722.06
IINEPTAAAIAYGLDKR	1739	7.07	3	1814.9894	3		0		no	4.99	630.42	316.18
ITITNDQNR	2325	6.52	2	1073.5386	2		0		no	4.36	1881.74	329.41
ITPSYVAFTPEGER	1037	6.47	5	1565.7896	2		0		no	5.96	1277.04	1224.00
LSSEDKETMEK	4236	4.05	1	1309.6181	2		0	[6] Methyl K	no	4.99	323.88	21.43
NQLTSNPENTVFDAK	5432	6.64	2	1676.7992	2		0		no	6.44	167.18	62.52
NQLTSNPENTVFDAKR	3154	6.56	2	1832.8971	3		0		no	4.78	377.28	254.19
SQIFSTASDNQPTVTIK	6568	6.55	2	1835.9072	2		0		no	6.93	161.23	65.47
STMKPVQKVLEDSDLK	1505	4.47	2	1830.9937	4		0	[16] Methyl K	yes	3.81	1083.44	363.25
STMKPVQKVLEDSDLK	1909	4.47	2	1830.9765	3		0	[16] Methyl K	yes	4.71	1437.23	650.53
TWNDPSVQQDIK	3873	4.05	1	1429.6584	2		0		no	5.47	497.64	205.09
VTAEDKGTGNK	386	5.84	1	1132.5708	2		0	[11] Methyl K	yes	4.43	3405.05	8894.29
VYEGERPLTK	5394	5.16	1	1190.6184	3		0		no	3.53	209.71	80.89

Tags	
	Anova p-value ≤ 0.05
	power>0.8
	q<0.05

P02787

Serotransferrin OS=Homo sapiens OX=9606 GN=TF PE=1 SV=3
12 peptides

Sequence	Peptide Ion	Score	Hits	Mass	Charge	Tags	Conflicts	Modifications	In quantitation	Drift time (ms)	Average Normalised Abundances	
											CTR	MCD
ASYLDCIR	1762	6.39	4	996.4769	2		0	[6] Carbamidomethyl C	no	4.22	1065.70	233.5
DGAGDVAFVK	6980	4.39	1	977.4800	2		0		no	4.02	114.58	55.4
DLLFKDSAHGFLK	8220	4.88	1	1489.7874	2		0		no	5.75	91.67	25.9
DLLFRDDTVCLAK	3543	4.90	1	1564.7886	3		0	[10] Carbamidomethyl C	no	4.09	349.94	23.6
DLLFRDDTVCLAK	8088	4.90	1	1564.7821	2		0	[10] Carbamidomethyl C	no	6.37	118.38	2.6
DSGFQMNQLR	1107	5.22	1	1194.5225	3		0		yes	3.39	538.13	1881.[illegible]
DSGFQMNQLR	1542	5.22	1	1194.5346	2		0		yes	4.92	945.00	1837.2
DSGFQMNQLR	2419	5.84	3	1210.5438	2		0	[6] Oxidation M	no	4.64	1010.90	195.1

Sequence	Peptide Ion	Score	Hits	Mass	Charge	Tags	Conflicts	Modifications	In quantitation	Drift time (ms)	Average Normalised Abundances	
											CTR	MCD
EGTCPEAPTDECKPVK	3980	5.79	4	1816.7948	3		0	[4] Carbamidomethyl C|[12] Carbamidomethyl C	no	4.22	303.44	3.5
IECVSAETTEDCIAK	5527	6.41	4	1724.7591	2		0	[3] Carbamidomethyl C|[12] Carbamidomethyl C	no	6.44	215.44	7.8
MYLGYEYVTAIR	2221	6.44	4	1493.7191	2		0	[1] Oxidation M	no	5.82	751.02	103.0
SASDLTWDNLK	3654	6.08	4	1248.6027	2		0		yes	4.92	561.44	644.1
SVIPSDGPSVACVK	1927	6.53	4	1414.7074	2		0	[12] Carbamidomethyl C	no	5.33	752.10	300.0
YLGEEYVK	1744	6.58	4	999.4936	2		0		no	4.36	886.72	158.5

Tags	
	Anova p-value ≤ 0.05
	power>0.8
	q<0.05

P02462

Collagen alpha-1(IV) chain OS=Homo sapiens OX=9606 GN=COL4A1 PE=1 SV=4
13 peptides

Sequence	Peptide Ion	Score	Hits	Mass	Charge	Tags	Conflicts	Modifications	In quantitation	Drift time (ms)	Average Normalis
											CTR
AHGQDLGTAGSCLRK	2556	4.29	1	1569.7866	3		0	[12] Carbamidomethyl C	no	4.09	468.90
GDHGFPGSSGPR	2997	4.01	1	1169.5138	2		0		no	4.43	370.53
GDPGTPGVPGK	5645	5.62	1	1012.4824	2		0	[2] Hydroxyl DKNP|[6] Hydroxyl DKNP	no	3.88	87.12
GEAGPPGPPGIVIGTGPLGEK	3598	4.71	1	1915.0031	3		0	[6] Hydroxyl DKNP	no	5.75	155.09
GEAGPPGPPGIVIGTGPLGEK	3409	4.71	1	1914.9944	2		0	[6] Hydroxyl DKNP	no	7.20	135.53
GEKGFPGFPGLDMPGPK	2561	4.98	1	1759.8520	2		0	[3] Methyl K|[9] Hydroxyl DKNP	no	6.72	327.92
GFPGTSLPGPSGR	3563	5.42	2	1244.6154	2		0	[3] Hydroxyl DKNP	no	4.78	466.73
GFPGTSLPGPSGR	636	6.33	2	1260.6110	2		0	[3] Hydroxyl DKNP|[8] Hydroxyl DKNP	yes	4.78	2933.53
GPPGGVGFPGSR	845	6.09	2	1115.5389	2		0	[2] Hydroxyl DKNP|[9] Hydroxyl DKNP	yes	4.16	1595.06
GQIGPIGEK	1097	4.20	1	897.5037	2		0		yes	3.88	2667.26
GVPGIPGPQGSPGLPGDK	7093	4.15	1	1628.8516	2		0		no	6.37	139.96
GYPGTPGPR	7921	---	---	932.4331	1		0		no	10.32	30.28

Sequence	Peptide Ion	Score	Hits	Mass	Charge	Tags	Conflicts	Modifications	In quantitation	Drift time (ms)	Average Normalis Abundan
											CTR
								[3] Hydroxyl DKNP | [6] Hydroxyl DKNP			
GYPGTPGPR	1466	5.72	2	932.4354	2		0	[3] Hydroxyl DKNP | [6] Hydroxyl DKNP	no	3.67	654.22
HSQTIDDPQCPSGTK	13760	---	---	1669.7431	2		0	[10] Carbamidomethyl C	no	6.23	10.68
HSQTIDDPQCPSGTK	1794	6.03	2	1669.7356	3		0	[10] Carbamidomethyl C	no	3.88	697.56
SAPFIECHGR	3609	6.21	1	1172.5432	2		0	[7] Carbamidomethyl C	no	4.57	335.85
SAPFIECHGR	1875	5.81	1	1172.5423	2		0	[7] Carbamidomethyl C	no	4.57	795.34
SAPFIECHGR	9300	6.21	1	1172.5363	3		0	[7] Carbamidomethyl C	no	3.25	36.59
SAPFIECHGR	5905	---	---	1172.5382	3		0	[7] Carbamidomethyl C	no	3.25	78.07

Tags	
	Anova p-value ≤ 0.05
	power>0.8
	q<0.05

P15311

Ezrin OS=Homo sapiens OX=9606 GN=EZR PE=1 SV=4
13 peptides

Sequence	Peptide Ion	Score	Hits	Mass	Charge	Tags	Conflicts	Modifications	In quantitation	Drift time (ms)	Average Normalised Abundances	
											CTR	MCD
AERELSEQIQR	256	5.15	1	1357.6971	2		0		yes	5.47	1.41e+004	6201.62
AERELSEQIQR	450	5.06	2	1357.6962	3		0		no	3.74	5991.53	2829.87
AERELSEQIQR+	184	0.00	1	1339.6877	3		0	[C-term] Dehydrated	yes	3.60	1.92e+004	1.12e+004
AKEELER	3460	5.20	1	889.4489	2		0	[2] Hydroxyl DKNP	no	3.74	270.57	279.51
ALQLEEER	1710	5.10	1	986.4980	2		0		no	4.09	1200.42	568.33
EAQDDLVK	4613	5.69	1	916.4473	2		0		no	3.95	414.59	187.76
ELSEQIQR	1832	6.94	4	1001.5136	2		0		no	4.09	810.34	406.96
ETVEREKEQMMR	3661	5.28	1	1564.7185	3		0		no	4.02	164.24	109.26
ETVEREKEQMMR	5950	5.28	1	1564.7219	4		0		no	3.60	55.51	8.85
IALLEEAR	2922	6.73	4	913.5207	2		0		no	4.09	416.67	211.81
LQDYEEK	4483	5.29	1	923.4228	2		0		no	3.95	271.24	85.09
QLEEER	5759	0.00	1	802.3823	1		0		no	8.66	85.69	10.50
QRIDEFEAL	1388	6.96	4	1119.5546	2		0		no	4.43	462.63	665.32
SGYLSSER	6458	6.44	1	897.4220	2		0		no	3.81	217.47	129.48

Sequence	Peptide Ion	Score	Hits	Mass	Charge	Tags	Conflicts	Modifications	In quantitation	Drift time (ms)	Average Normalised Abundances	
											CTR	MCD
VSAQEVRK	421	5.33	1	915.5145	2		0		yes	3.95	1.07e+004	3817.46
VSAQEVRK	14373	---	---	915.5168	3		0		yes	2.98	30.83	0.00

Tags	
	Anova p-value ≤ 0.05
	power>0.8
	q<0.05

P62805

Histone H4 OS=Homo sapiens OX=9606 GN=H4C1 PE=1 SV=2
9 peptides

Sequence	Peptide Ion	Score	Hits	Mass	Charge	Tags	Conflicts	Modifications	In quantitation	Drift time (ms)	Average Normalised Abundances	
											CTR	MCD
DAVTYTEHAK	1959	8.09	5	1133.5365	2		0		no	4.71	561.72	462.64
DAVTYTEHAK	9410	---	---	1133.5328	3		0		no	3.32	34.52	2.97
DNIQGITKPAIR	818	8.28	6	1324.7453	3		0		no	3.95	1530.88	1162.93
DNIQGITKPAIR	1949	8.28	4	1324.7449	2		0		no	5.47	550.44	310.00
ISGLIYEETR	541	8.87	12	1179.6119	2		0		yes	4.71	3617.04	3275.47
ISGLIYEETRGVLK	4667	7.62	2	1576.8809	3		0		no	4.09	226.77	164.45
QGITKPAIR	3436	0.00	1	982.5929	2		0		no	4.22	209.28	137.51
TLYGFGG	73	8.12	12	713.3402	1		0		yes	8.24	1.75e+004	1.64e+004
TVTAMDVVYALK	1726	6.98	2	1325.6890	2		0	[5] Oxidation M	no	5.61	606.56	234.94
TVTAMDVVYALK	2364	7.60	1	1325.6889	2		0	[5] Oxidation M	no	5.61	585.30	241.03
TVTAMDVVYALKR	1293	7.69	2	1481.7920	3		0	[5] Oxidation M	no	3.74	1200.82	544.17
TVTAMDVVYALKR	1325	6.96	4	1481.7931	3		0	[5] Oxidation M	no	3.74	1163.44	441.20
VFLENVIR	726	8.71	6	988.5717	2		0		yes	4.36	1179.46	1649.02

Tags	
	Anova p-value ≤ 0.05
	power>0.8
	q<0.05

P01860

Immunoglobulin heavy constant gamma 3 OS=Homo sapiens OX=9606 GN=IGHG3 PE=1 SV=2
11 peptides

Sequence	Peptide Ion	Score	Hits	Mass	Charge	Tags	Conflicts	Modifications	In quantitation	Drift time (ms)	Average Normalised Abundances	
											CTR	MCD

Sequence	Peptide Ion	Score	Hits	Mass	Charge	Tags	Conflicts	Modifications	In quantitation	Drift time (ms)	Average Normalised Abundances	
											CTR	MCD
ALPAPIEK	1677	7.30	4	837.4897	2		0		no	4.02	383.65	168.9
APIEK	4070	0.00	1	556.3195	1		0		no	6.58	104.61	60.2
DTLMISR	538	7.55	4	850.4242	2		0	[4] Oxidation M	yes	3.67	4950.25	2246.4
DTLMISR	4486	7.55	2	850.4229	1		0	[4] Oxidation M	no	9.42	162.44	1.3
EPQVYTLPPSREEMTK	7536	5.39	1	1903.9163	3		0		no	4.29	144.22	22.3
EPQVYTLPPSREEMTK	1749	6.71	2	1919.9293	3		0	[9] Hydroxyl DKNP	yes	4.64	3363.18	377.0
GPSVFPLAPCSR	790	7.75	4	1286.6443	2		0	[10] Carbamidomethyl C	no	4.92	1732.07	206.4
NQVSLTCLVK	1671	8.30	4	1160.6208	2		0	[7] Carbamidomethyl C	no	4.92	1224.40	527.3
PAPIEK	573	0.00	2	653.3754	1		0		no	7.48	1001.12	170.3
PAPIEK	959	0.00	2	653.3751	2		0		no	3.25	670.02	136.5
SCDTPPPCPR	3620	6.62	2	1185.4927	2		0	[2] Carbamidomethyl C | [8] Carbamidomethyl C	no	4.50	602.14	11.6
STSGGTAALGCLVK	2289	7.51	4	1320.6467	2		0	[11] Carbamidomethyl C	no	5.19	665.50	156.3
WYVDGVEVHNAK	2879	5.34	1	1415.6685	2		0		yes	5.40	324.31	1125.0

Tags	
	Anova p-value ≤ 0.05
	power>0.8
	q<0.05

P68431

Histone H3.1 OS=Homo sapiens OX=9606 GN=H3C1 PE=1 SV=2
6 peptides

Sequence	Peptide Ion	Score	Hits	Mass	Charge	Tags	Conflicts	Modifications	In quantitation	Drift time (ms)	Average Normalised Abundances	
											CTR	MCD
DIQLAR	1024	8.54	5	714.4024	1		0		no	8.38	1034.15	1004.35
DIQLAR	1392	8.54	3	714.4024	2		0		no	3.39	607.57	756.33
EIAQDFKTDLR	999	7.49	5	1334.6822	3		0		no	4.02	1204.71	1331.05
EIAQDFKTDLR	2657	7.49	2	1334.6807	2		0		no	5.47	201.14	160.73
EIAQDFKTDLR	727	7.22	1	1348.6991	3		0	[7] Methyl K	yes	3.88	1474.60	1584.27
EIAQDFKTDLR	1834	7.22	1	1348.6964	2		0	[7] Methyl K	yes	5.61	466.81	399.27
EIAQDFKTDLR+	1946	0.00	2	1316.6717	3		0	[C-term] Dehydrated	no	3.88	396.59	398.37
STELLIR	253	8.29	11	830.4883	2		0		yes	3.67	5092.18	5648.60
STELLIR	1569	8.29	2	830.4868	1		0		no	9.63	466.08	327.96
VTIMPKDIQLAR	2692	6.45	2	1399.7829	3		0	[4] Oxidation M	yes	4.09	592.99	2476.29

Tags	
	Anova p-value ≤ 0.05
	power>0.8
	q<0.05

Q15599

Na(+)/H(+) exchange regulatory cofactor NHE-RF2 OS=Homo sapiens OX=9606 GN=SLC9A3R2 PE=1 SV=2
9 peptides

Sequence	Peptide Ion	Score	Hits	Mass	Charge	Tags	Conflicts	Modifications	In quantitation	Drift time (ms)	Average Normalised Abundances	
											CTR	MCD
EIFSNF	3213	5.18	1	755.3490	1		0		yes	8.31	182.12	244.05
HAEVVASIK	3917	6.51	2	952.5325	2		0		no	4.57	221.71	47.85
LIEVNGQNVEGLR	6550	6.32	1	1439.7498	2		0		no	5.54	129.30	13.91
LLVVDPETDEHFK	6581	5.31	2	1540.7758	3		0		no	3.95	74.24	13.27
LLVVDPETDEHFKR	4021	6.38	2	1696.8770	3		0		no	4.22	156.84	92.00
LLVVDPETDEHFKR	4733	6.38	2	1696.8754	4		0		no	3.39	129.48	55.16
LLVVDQETDEELR	7257	6.43	2	1557.7726	2		0		no	6.16	115.16	7.29
LLVVDQETDEELRR	3614	6.11	2	1713.8872	3		0		no	4.36	272.56	108.24
RVEPGSPAEAAALR	2689	6.76	2	1422.7497	3		0		yes	4.09	348.68	177.25
RVEPGSPAEAAALR	5253	6.76	1	1422.7466	2		0		yes	5.61	78.54	12.70
SVDPGSPAAR	1320	6.71	2	955.4717	2		0		yes	3.67	568.80	385.89

Tags	
	Anova p-value ≤ 0.05
	power>0.8
	q<0.05

P0CG47

Polyubiquitin-B OS=Homo sapiens OX=9606 GN=UBB PE=1 SV=1
8 peptides

Sequence	Peptide Ion	Score	Hits	Mass	Charge	Tags	Conflicts	Modifications	In quantitation	Drift time (ms)	Average Normalised Abundances	
											CTR	MCD
EGIPPDQQR	6271	6.56	1	1038.5168	2		0		no	4.22	403.86	84.8[illegible]
IQDKEGIPPDQQR	2023	6.62	2	1522.7709	3		0		yes	3.81	5860.93	3091.1[illegible]
LIFAGK	2930	8.16	2	647.4003	1		0		no	7.89	289.59	120.7[illegible]
LIFAGK	4431	---	---	647.3997	2		0		no	3.19	158.02	42.2[illegible]
LIFAGKQLEDGR	155	5.74	1	1359.7523	2		0	[6] Methyl K	yes	5.47	4897.06	2.59e+00[illegible]
QLEDGRTLSDYNIQK	3047	6.25	2	1778.8765	3		0		no	4.22	564.59	238.4[illegible]
TITLEVEPSDTIENVK	310	9.05	15	1786.9173	2		0		yes	7.06	6360.76	4301.9[illegible]
TITLEVEPSDTIENVK	3313	9.05	4	1786.9248	3		0		yes	4.64	549.26	250.6[illegible]

Sequence	Peptide Ion	Score	Hits	Mass	Charge	Tags	Conflicts	Modifications	In quantitation	Drift time (ms)	Average Normalised Abundances	
											CTR	MCD
TITLEVEPSDTIENVKAK	3132	7.48	4	1986.0488	3		0		no	5.12	645.59	230.7[illegible]
TLSDYNIQK	605	8.52	11	1080.5455	2		0		no	4.36	3117.50	1984.0[illegible]

Tags	
	Anova p-value ≤ 0.05
	power>0.8
	q<0.05

P07911

Uromodulin OS=Homo sapiens OX=9606 GN=UMOD PE=1 SV=1
10 peptides

Sequence	Peptide Ion	Score	Hits	Mass	Charge	Tags	Conflicts	Modifications	In quantitation	Drift time (ms)	Average Normalised Abundances	
											CTR	MCD
DGPCGTVLTR	2061	6.70	3	1074.5317	2		0	[4] Carbamidomethyl C	no	4.29	1111.11	449.61
FSVQMFR	2084	6.03	2	929.4502	2		0	[5] Oxidation M	no	4.02	701.51	126.37
FSVQMFR	2361	6.49	2	929.4448	2		0	[5] Oxidation M	no	3.95	631.61	80.54
INFACSYPLDMK	6701	5.56	2	1473.6643	2		0	[5] Carbamidomethyl C|[8] Hydroxyl DKNP	no	5.68	171.04	0.38
MAETCVPVLR	2542	7.48	1	1174.5861	2		0	[5] Carbamidomethyl C	no	4.57	772.38	65.59
MAETCVPVLR	943	7.14	4	1190.5797	2		0	[1] Oxidation M|[5] Carbamidomethyl C	yes	4.50	1990.45	208.02
STEYGEGYACDTDLR	3919	6.99	4	1735.6975	2		0	[10] Carbamidomethyl C	no	6.51	518.80	26.21
TALQPMVSALNIR	1429	6.32	4	1428.7665	2		0	[5] Hydroxyl DKNP	yes	5.61	1769.71	448.23
TLDEYWR	1431	7.05	4	981.4572	2		0		no	4.09	1292.07	218.76
VGGTGMFTVR	1194	6.58	4	1039.5125	2		0	[6] Oxidation M	yes	4.16	2197.83	158.97
YFIIQDR	2231	6.88	4	953.4973	2		0		no	4.16	744.78	159.42

Tags	
	Anova p-value ≤ 0.05
	power>0.8
	q<0.05

P07437

Tubulin beta chain OS=Homo sapiens OX=9606 GN=TUBB PE=1 SV=2
10 peptides

Sequence	Peptide Ion	Score	Hits	Mass	Charge	Tags	Conflicts	Modifications	In quantitation	Drift time (ms)	Average Normalised Abundances	
											CTR	MCD
AILVDLEPGTMDSVR	508	7.04	1	1630.8221	2		0	[11] Oxidation M	yes	6.23	3431.46	2167.37
AILVDLEPGTMDSVR	5117	---	---	1630.8123	3		0	[11] Oxidation M	yes	4.36	91.06	30.72
AILVDLEPGTMDSVR	4838	6.47	2	1646.8140	2		0	[8] Hydroxyl DKNP|[12] Hydroxyl DKNP	no	6.16	163.89	100.49
ALTVPELTQQVFDAK	3043	5.91	2	1658.8829	2		0		no	6.51	544.81	223.95
ALTVPELTQQVFDAK	6716	5.91	2	1658.8869	3		0		no	4.36	160.33	42.05
FPGQLNADLR	2369	5.97	2	1129.5922	2		0		yes	4.57	1692.98	809.07
FPGQLNADLRK	5686	4.80	1	1257.6794	3		0		no	3.32	181.96	10.04
GHYTEGAELVDSVLDVVR	7293	5.17	1	1958.0014	2		0		no	7.34	206.51	30.85
IMNTFSVVPSPK	1933	6.23	2	1334.6860	2		0	[3] Hydroxyl DKNP	no	5.26	924.07	287.89
IREEYPDR	5420	7.16	2	1076.5238	2		0		no	4.36	163.39	5.24
ISEQFTAMFR	2229	5.25	1	1228.5948	2		0		no	4.78	975.98	371.73
ISVYYNEATGGK	1090	6.40	1	1300.6294	2		0		yes	5.06	1294.77	932.61

Tags	
	Anova p-value ≤ 0.05
	power>0.8
	q<0.05

Q02413

Desmoglein-1 OS=Homo sapiens OX=9606 GN=DSG1 PE=1 SV=2
10 peptides

Sequence	Peptide Ion	Score	Hits	Mass	Charge	Tags	Conflicts	Modifications	In quantitation	Drift time (ms)
ALNSMGQDLERPLELR	4797	5.35	2	1840.9389	3		0		no	4.71
IHSDCAANQQVTYR	3517	6.44	2	1661.7602	3		0	[5] Carbamidomethyl C	yes	4.16
IIRQEPSDSPMFIINR	4406	5.52	1	1930.9900	3		0	[15] Hydroxyl DKNP	yes	4.29
ISGVGIDQPPYGIFVINQK	3150	6.72	2	2044.0933	3		0		yes	5.19
ISGVGIDQPPYGIFVINQK	4279	6.72	2	2044.0934	2		0		yes	7.62
MDWSFFRVVAMLFIFLVVVEVNSEFR	4167	5.53	1	3195.6160	4		0	[2] Hydroxyl DKNP	no	5.75
SAAGFEPVPECSDGAIHSWAVEGPQPEPR	4419	5.10	2	3076.3781	3		0	[11] Carbamidomethyl C	no	5.96
TMNNFLDREQYGQYALAVR	5587	5.33	1	2304.0956	3		0		no	5.12

Sequence	Peptide Ion	Score	Hits	Mass	Charge	Tags	Conflicts	Modifications	In quantitation	Drift time (ms)
								[7] Hydroxyl DKNP		
VGDFVATDLDTGRPSTTVR	4676	5.16	1	2006.0043	3		0		no	4.64
YQGTILSIDDNLQR	4321	6.81	2	1634.8224	2		0		no	6.37
YVMGNNPADLLAVDSR	4158	5.57	1	1749.8351	2		0	[7] Hydroxyl DKNP	no	6.58

Tags	
	Anova p-value ≤ 0.05
	power>0.8
	q<0.05

P67936

Tropomyosin alpha-4 chain OS=Homo sapiens OX=9606 GN=TPM4 PE=1 SV=3
9 peptides

Sequence	Peptide Ion	Score	Hits	Mass	Charge	Tags	Conflicts	Modifications	In quantitation	Drift time (ms)	Average Normalised Abundances	
											CTR	MCD
AEERAEVSELK	6086	5.45	1	1259.6360	3		0		no	3.46	194.18	16
AEFAERTVAK	543	5.47	1	1120.5878	2		0		yes	4.57	6412.91	2262
AEGDVAALNR	3445	6.23	2	1014.5134	2		0		yes	4.16	629.24	323
IQALQQQADEAEDR	12608	6.71	1	1613.7511	2		0		no	6.02	34.36	5
IQALQQQADEAEDRAQGLQR	9387	5.63	1	2267.1228	3		0		no	5.26	98.75	39
IQLVEEELDR	2815	6.93	2	1242.6433	2		0		no	4.92	336.15	230
IQLVEEELDRAQER	464	6.85	2	1726.8872	3		0		yes	4.36	3625.18	4007
IQLVEEELDRAQER	5389	6.85	2	1726.8811	2		0		yes	7.06	141.27	79
LATALQK	3802	6.18	1	743.4521	2		0		no	3.25	169.57	78
LATALQKLEEAEK	2999	6.27	1	1442.7967	3		0		no	3.95	345.65	224
LATALQKLEEAEK	6878	---	---	1442.7816	2		0		no	6.16	51.85	6

Tags	
	Anova p-value ≤ 0.05
	power>0.8
	q<0.05

P06396

Gelsolin OS=Homo sapiens OX=9606 GN=GSN PE=1 SV=1
10 peptides

Sequence	Peptide Ion	Score	Hits	Mass	Charge	Tags	Conflicts	Modifications	In quantitation	Drift time (ms)	Average Normalised Abundance	
											CTR	M
AGALNSNDAFVLK	1380	7.14	2	1318.6852	2		0		yes	5.33	944.42	3
AMAELAA	4143	5.76	2	691.3205	1		0	[2] Oxidation M	no	7.55	201.99	
AQPVQVAEGSEPDGFWEALGGK	7448	4.97	1	2271.0675	3		0		no	5.54	92.97	
AQPVQVAEGSEPDGFWEALGGK	12383	---	---	2271.0697	2		0		no	8.10	12.48	
AVEVLPK	1940	5.70	1	754.4553	2		0		no	3.67	214.59	
AVEVLPK	4348	---	---	754.4593	1		0		no	9.00	49.85	
QTQVSVLPEGGETPLFK	4551	6.72	2	1828.9498	2		0		no	6.72	206.47	
TASDFITK	1022	6.50	2	881.4359	2		0		yes	3.74	3487.07	5
TGAQELLR	1396	7.01	2	886.4863	2		0		yes	3.81	949.99	4
TPSAAYLWVGTGASEAEK	12711	5.29	1	1836.8888	2		0		no	6.93	35.49	
YIETDPANR	2914	6.79	2	1077.5103	2		0		no	4.36	616.50	2
YIETDPANRDR	2920	5.93	2	1348.6367	3		0		no	3.74	587.24	1
YIETDPANRDR	14454	---	---	1348.6328	2		0		no	5.19	26.42	

Tags	
	Anova p-value ≤ 0.05
	power>0.8
	q<0.05

P08779

Keratin_ type I cytoskeletal 16 OS=Homo sapiens OX=9606 GN=KRT16 PE=1 SV=4
10 peptides

Sequence	Peptide Ion	Score	Hits	Mass	Charge	Tags	Conflicts	Modifications	In quantitation	Drift time (ms)	Average Normalised Abundances	
											CTR	MC
APSTYGGGLSVSSR	3182	6.61	4	1337.6540	2		0		no	5.19	826.35	58
DAETWFLSK	2046	7.01	4	1095.5241	2		0		no	4.43	779.87	86
DAETWFLSKTEELNK	3098	5.55	1	1841.8581	3		0	[1] Hydroxyl DKNP|[14] Hydroxyl DKNP	no	4.22	289.60	17
DQYEQMAEK	1341	5.94	1	1140.4837	2		0		yes	4.57	2085.03	89
EVFTSSSSSSR	5291	6.45	3	1259.5612	2		0		no	4.92	323.63	45
ILNEMRDQYEQMAEK	8245	5.81	1	1928.8546	3		0	[3] Hydroxyl DKNP|[15] Hydroxyl DKNP	no	4.57	48.68	30
KNHEEEMLALR	1865	4.53	1	1368.6923	2		0		yes	5.33	87.13	223
LLEGEDAHLSSQQASGQSYSSR	4777	5.73	1	2349.0721	3		0		no	5.06	257.15	51
TEELNKEVASNSELVQSSR	1902	6.86	4	2119.0337	3		0		yes	4.57	897.69	150
TKYEHELALR	2433	6.44	3	1258.6647	3		0		no	3.88	845.38	70

Tags	
	Anova p-value ≤ 0.05
	power>0.8

	q<0.05

Q01955

Collagen alpha-3(IV) chain OS=Homo sapiens OX=9606 GN=COL4A3 PE=1 SV=3
12 peptides

Sequence	Peptide Ion	Score	Hits	Mass	Charge	Tags	Conflicts	Modifications	In quantitation	Drift time (ms)	Average Normalisec Abundance	
											CTR	M
AHGQDLGTLGSCLQR	14129	5.94	1	1611.7759	2		0	[12] Carbamidomethyl C	no	6.16	19.79	
ALEPYISR	384	7.16	2	947.5056	2		0		no	4.02	3612.80	3
FTTMPFLFCNVNDVCNFASR	5045	5.52	1	2455.0675	3		0	[4] Oxidation M | [9] Carbamidomethyl C | [15] Carbamidomethyl C	no	5.33	191.37	
GAPGFPGLPGR	1435	6.15	2	1072.5350	2		0	[3] Hydroxyl DKNP | [6] Hydroxyl DKNP | [9] Hydroxyl DKNP	no	4.29	1927.41	1
GDLGSTGNPGEPGLR	424	6.95	2	1457.6751	2		0	[9] Hydroxyl DKNP | [12] Hydroxyl DKNP	yes	5.33	4711.84	3
GDSGSPATWTTR	7426	5.27	1	1250.5549	2		0	[6] Hydroxyl DKNP	no	4.78	159.80	
GEPGLK	198	5.35	1	613.3448	1		0	[6] Methyl K	yes	7.34	3733.37	3
GFAGNPGEK	4713	4.82	1	891.4114	2		0	[6] Hydroxyl DKNP	no	3.88	205.50	
GFPGR	3902	0.00	1	548.2718	1		0	[3] Hydroxyl DKNP	no	6.44	130.93	
GTLGFPGR	3258	5.47	1	819.4228	2		0	[6] Hydroxyl DKNP	no	3.60	301.75	
LGAPGTPGLPGPR	321	6.39	2	1236.6469	2		0	[4] Hydroxyl DKNP | [7] Hydroxyl DKNP | [10] Hydroxyl DKNP	yes	4.78	4248.88	3
PYISR	975	0.00	1	634.3447	1		0		no	7.76	608.93	

Tags	
	Anova p-value ≤ 0.05
	power>0.8
	q<0.05

P32119

Peroxiredoxin-2 OS=Homo sapiens OX=9606 GN=PRDX2 PE=1 SV=5
8 peptides

Sequence	Peptide Ion	Score	Hits	Mass	Charge	Tags	Conflicts	Modifications	In quantitation	Drift time (ms)	Average Normalised Abundances	
											CTR	MCD
GLFIIDGK	3026	7.23	1	861.4950	2		0		no	3.74	322.17	56.39
GLFIIDGK	10485	---	---	861.4941	1		0		no	9.97	22.82	0.00
KEGGLGPLNIPLLADVTR	6137	7.16	2	1862.0612	3		0		no	4.29	154.59	10.99
LSEDYGVLK	2174	6.81	4	1022.5232	2		0		yes	4.22	568.46	272.90
LSEDYGVLKTDEGIAYR	4444	6.76	4	1927.9502	3		0		no	4.50	479.04	111.79
QITVNDLPVGR	286	8.24	12	1210.6674	2		0		yes	4.78	6840.89	3658.28
RLSEDYGVLK	6288	6.00	1	1192.6375	2		0	[10] Methyl K	no	4.99	92.93	3.70
SVDEALR	1881	7.59	4	788.4035	2		0		yes	3.53	799.23	286.76
TDEGIAYR	3822	7.68	3	923.4328	2		0		no	3.81	543.48	80.04

Tags	
	Anova p-value ≤ 0.05
	power>0.8
	q<0.05

P0DMV8

Heat shock 70 kDa protein 1A OS=Homo sapiens OX=9606 GN=HSPA1A PE=1 SV=1
7 peptides

Sequence	Peptide Ion	Score	Hits	Mass	Charge	Tags	Conflicts	Modifications	In quantitation	Drift time (ms)	Average Normalised Abundances	
											CTR	MCD
DAGVIAGLNVLR	2200	7.60	2	1196.6865	2		0		no	4.99	344.97	101.34
FGDPVVQSDMK	1028	5.10	1	1221.5477	2		0		yes	4.57	1299.45	957.59
IINEPTAAAIAYGLDR	2011	8.02	5	1686.8923	2		0		yes	6.44	706.67	339.91
IINEPTAAAIAYGLDR	4315	8.02	2	1686.8923	3		0		yes	4.64	215.97	79.23
NQVALNPQNTVFDAK	3872	7.68	2	1657.8335	2		0		no	6.37	228.87	84.17
NQVALNPQNTVFDAKR	5541	6.76	2	1813.9372	3		0		no	4.50	148.09	52.97
STLEPVEK	1869	7.39	4	901.4840	2		0		yes	3.88	1654.06	774.65
YKAEDEVQR	6500	5.73	1	1150.5589	2		0	[2] Methyl K	no	4.71	123.83	26.26

Tags	
	Anova p-value ≤ 0.05
	power>0.8
	q<0.05

P01876

Immunoglobulin heavy constant alpha 1 OS=Homo sapiens OX=9606 GN=IGHA1 PE=1 SV=2
6 peptides

Sequence	Peptide Ion	Score	Hits	Mass	Charge	Tags	Conflicts	Modifications	In quantitation	Drift time (ms)	Average Normalised Abundances	
											CTR	MCD
QEPSQGTTTFAVTSILR	2788	7.96	4	1834.9403	2		0		yes	6.86	852.96	80.4[illegible]
QEPSQGTTTFAVTSILR	4854	7.96	3	1834.9423	3		0		yes	4.92	327.67	245.0[illegible]
SAVQGPPER	1390	8.15	4	939.4772	2		0		yes	3.81	1277.55	229.5[illegible]
TFTCTAAYPESK	1627	7.70	4	1374.6136	2		0	[4] Carbamidomethyl C	no	5.33	998.76	254.6[illegible]
TPLTATLSK	427	8.29	4	930.5397	2		0		yes	3.95	3184.12	941.8[illegible]
VAAEDWK	2360	7.59	3	817.3975	2		0		no	3.39	993.39	46.4[illegible]
WLQGSQELPR	1699	7.36	4	1212.6234	2		0		no	4.78	1145.51	249.3[illegible]

Tags	
	Anova p-value ≤ 0.05
	power>0.8
	q<0.05

P02679

Fibrinogen gamma chain OS=Homo sapiens OX=9606 GN=FGG PE=1 SV=3
8 peptides

Sequence	Peptide Ion	Score	Hits	Mass	Charge	Tags	Conflicts	Modifications	In quantitation	Drift time (ms)	Average Normalis[illegible] Abundan[illegible]
											CTR
AIQLTYNPDESSKPNMIDAATLK	4589	6.46	1	2535.2389	3		0	[14] Hydroxyl DKNP	no	5.54	207.46
DNCCILDER	1240	7.76	2	1193.4848	2		0	[3] Carbamidomethyl C|[4] Carbamidomethyl C	yes	4.57	872.62
EGFGHLSPTGTTEFWLGNEK	9565	5.57	1	2206.0265	3		0		no	4.71	53.88
LDGSVDFK	4177	6.43	1	879.4332	2		0		no	3.60	209.22
TSTADYAMFK	1674	7.05	2	1149.5068	2		0	[10] Hydroxyl DKNP	yes	4.57	1027.66
VELEDWNGR	2931	6.85	2	1116.5320	2		0		no	4.43	421.13
VGPEADKYR	4244	7.04	2	1033.5162	2		0		no	4.36	182.04
VGPEADKYR	6645	---	---	1033.5174	3		0		no	3.19	39.89
YLQEIYNSNNQK	2195	7.62	2	1512.7203	2		0		yes	5.75	712.56

Tags	
	Anova p-value ≤ 0.05
	power>0.8
	q<0.05

P04406

Glyceraldehyde-3-phosphate dehydrogenase OS=Homo sapiens OX=9606 GN=GAPDH PE=1 SV=3
7 peptides

Sequence	Peptide Ion	Score	Hits	Mass	Charge	Tags	Conflicts	Modifications	In quantitation	Drift time (ms)	Average Normalised Abundanc	
											CTR	[illegible]
GALQNIIPASTGAAK	977	8.06	6	1410.7712	2		0		yes	5.47	2796.54	2
IISNASCTTNCLAPLAK	5318	6.25	2	1832.9050	2		0	[7] Carbamidomethyl C|[11] Carbamidomethyl C	no	6.93	265.04	
LISWYDNEFGYSNR	3077	7.76	5	1762.7919	2		0		no	6.58	684.81	
LWRDGR	269	4.75	1	801.4228	1		0		yes	8.86	4802.35	3
VIISAPSADAPMFVMGVNHEK	3602	6.02	1	2244.0839	3		0	[11] Hydroxyl DKNP|[18] Hydroxyl DKNP	no	5.54	536.39	
VVDLMAHMASKE	1016	5.97	1	1361.6314	3		0	[5] Oxidation M| [8] Oxidation M	yes	3.81	2424.13	1
VVDLMAHMASKE	8095	5.97	1	1361.6279	2		0	[5] Oxidation M| [8] Oxidation M	yes	5.33	141.62	
VVKQASEGPLK	927	4.92	1	1170.6575	3		0	[3] Hydroxyl DKNP	no	3.67	1349.71	1
VVKQASEGPLK	2458	4.92	1	1170.6582	2		0	[3] Hydroxyl DKNP	no	4.71	446.16	

Tags	
	Anova p-value ≤ 0.05
	power>0.8
	q<0.05

P00761

Trypsin OS=Sus scrofa OX=9823 PE=1 SV=1
14 peptides

Sequence	Peptide Ion	Score	Hits	Mass	Charge	Tags	Conflicts	Modifications	In quantitation	Drift time (ms)	Average Norm Abundances	
											CTR	MC
ATLNSR	354	0.00	9	660.3556	1		0		no	7.55	2328.52	[illegible]
ATVSLPR	13	0.00	6	742.4350	1		0		no	8.93	2.32e+004	5.4
ATVSLPR	381	0.00	6	742.4289	2		0		no	3.39	2335.91	[illegible]
ATVSLPR	301	0.00	1	724.4237	1		0		no	8.52	1899.00	[illegible]
ATVSLPR	643	---	---	724.4224	2		0		no	3.88	757.06	
LGEHNIDVLEGNEQFINAAK	391	7.84	16	2210.0963	3		0		no	4.92	8450.27	[illegible]
LGEHNIDVLEGNEQFINAAK	720	7.09	16	2224.1107	3		0	[20] Methyl K	no	4.92	2912.68	[illegible]
LSSPATLNSR	21	8.35	15	1044.5696	2		0		yes	4.16	7.18e+004	1.4
LSSPATLNSR	1315	8.35	3	1044.5559	1		0		yes	11.70	643.97	
LSSPATLNSR+	491	0.00	3	1026.5453	2		0	[C-term] Dehydrated	no	4.16	2695.91	[illegible]
LSSPATLNSR	1770	5.87	1	1076.5419	2		0	[4] Hydroxyl DKNP|[8]	no	4.36	217.66	

Sequence	Peptide Ion	Score	Hits	Mass	Charge	Tags	Conflicts	Modifications	In quantitation	Drift time (ms)	Average Norm Abundances	
											CTR	MC[illegible]
								Hydroxyl DKNP				
PATLNSR	190	0.00	1	757.4086	1		0		no	8.66	5015.07	[illegible]
PATLNSR	599	0.00	1	757.4077	2		0		no	3.46	1677.33	[illegible]
SSPATLNSR	349	0.00	11	931.4708	1		0		no	10.11	3679.84	[illegible]
TLNSR	507	0.00	6	589.3191	1		0		no	6.99	1431.37	[illegible]
TVSLPR	5	0.00	8	671.3995	1		0		yes	8.10	4.27e+004	8.7[illegible]
TVSLPR	129	0.00	8	671.3980	2		0		yes	3.46	5074.01	1.2[illegible]
VATVSLPR	141	8.07	15	841.5021	1		0		yes	10.11	3842.21	[illegible]
VATVSLPR	14	8.07	12	841.5132	2		0		yes	3.67	6.78e+004	1.5[illegible]
VATVSLPR+	23	0.00	1	823.4945	2		0	[C-term] Dehydrated	no	3.74	2.35e+004	5.6[illegible]
VSLPR	30	0.00	11	570.3501	1		0		no	7.20	1.31e+004	3.0[illegible]
VSLPR	3424	---	---	570.3483	2		0		no	3.12	90.68	

Tags	
	Anova p-value ≤ 0.05
	power>0.8
	q<0.05

P07195

L-lactate dehydrogenase B chain OS=Homo sapiens OX=9606 GN=LDHB PE=1 SV=2
8 peptides

Sequence	Peptide Ion	Score	Hits	Mass	Charge	Tags	Conflicts	Modifications	In quantitation	Drift time (ms)	Average Normalised Abundances	
											CTR	MCD
FRYLMAEK	2004	4.76	1	1056.5485	2		0		yes	4.29	1746.24	1397.4[illegible]
GLTSVINQK	2240	6.89	2	958.5295	2		0		yes	4.09	1228.14	1798.5[illegible]
IVADKDYSVTANSK	2259	6.71	3	1509.7615	3		0		yes	4.02	1248.22	242.8[illegible]
IVVVTAGVR	2726	6.74	2	912.5686	2		0		no	3.88	521.07	96.6[illegible]
LIAPVAEEEATVPNNK	2002	7.69	3	1693.8862	2		0		no	6.51	903.67	214.5[illegible]
LNLVQR	2438	7.23	3	741.4489	2		0		no	3.32	322.81	80.3[illegible]
SADTLWDIQK	3330	5.86	2	1175.5965	2		0		no	4.57	598.02	214.1[illegible]
VIGSGCNLDSAR	2316	7.38	3	1247.5914	2		0	[6] Carbamidomethyl C	no	5.06	608.41	238.8[illegible]

Tags	
	Anova p-value ≤ 0.05
	power>0.8
	q<0.05

P08238

Heat shock protein HSP 90-beta OS=Homo sapiens OX=9606 GN=HSP90AB1 PE=1 SV=4
8 peptides

Sequence	Peptide Ion	Score	Hits	Mass	Charge	Tags	Conflicts	Modifications	In quantitation	Drift time (ms)	Average Normalised Abundances	
											CTR	MCD
ADLINNLGTIAK	3454	6.84	4	1241.6948	2		0		no	4.99	329.45	67.19
EQVANSAFVER	2307	6.97	4	1248.6080	2		0		no	4.92	669.55	339.69
GVVDSEDLPLNISR	1458	7.92	4	1512.7747	2		0		yes	5.75	1212.48	616.52
IDIIPNPQER	2454	6.68	4	1193.6437	2		0		yes	4.71	1305.10	909.82
NPDDITQEEYGEFYK	7047	6.37	2	1846.7906	2		0		no	6.86	117.77	38.03
TLTLVDTGIGMTK	5210	6.08	1	1364.7080	2		0	[11] Oxidation M	no	5.33	116.00	34.08
YHTSQSGDEMTSLSEYVSR	11979	5.36	1	2191.9532	3		0	[8] Hydroxyl DKNP	no	4.78	56.96	18.13
YIDQEELNK	2149	6.56	3	1150.5518	2		0		yes	4.50	700.27	446.39

Tags	
	Anova p-value ≤ 0.05
	power>0.8
	q<0.05

P00558

Phosphoglycerate kinase 1 OS=Homo sapiens OX=9606 GN=PGK1 PE=1 SV=3
10 peptides

Sequence	Peptide Ion	Score	Hits	Mass	Charge	Tags	Conflicts	Modifications	In quantitation	Drift time (ms)	Ave Abu
											CTR
AEPAKIEAFR	4756	5.63	1	1130.5874	2		0		yes	4.64	
ALESPERPFLAILLGGAK	4878	6.08	1	1767.9870	3		0		no	4.16	[illegible]
ALMDEVVK	3092	5.48	2	919.4691	2		0	[3] Oxidation M	yes	4.02	[illegible]
ITLPVDFVTADKFDENAK	4552	6.44	1	2022.0328	3		0		no	5.12	
ITLPVDFVTADKFDENAK	5769	5.94	1	2022.0285	3		0		no	5.12	[illegible]
LGDVYYVNDAFGTAHR	3976	6.13	2	1633.7859	3		0		no	4.16	[illegible]
LGDVYYVNDAFGTAHR	8400	5.15	1	1649.7733	2		0	[3] Hydroxyl DKNP	no	6.37	
LTLDKLDVK	8919	---	---	1043.6223	3		0		no	3.19	
TGQATVASGIPAGWMGLDCGPESSK	16	5.62	2	2476.1495	4		0	[19] Carbamidomethyl C	yes	4.92	6.1[illegible]
TGQATVASGIPAGWMGLDCGPESSK	194	---	---	2476.1356	3		0	[19] Carbamidomethyl C	yes	5.54	4[illegible]
VLNNMEIGTSLFDEEGAK	6411	5.69	1	1981.9188	2		0	[3] Hydroxyl DKNP	no	7.27	
YAEAVTR	2643	6.26	1	808.4048	2		0		no	3.53	[illegible]
YAEAVTR	11644	---	---	808.4039	1		0		no	9.49	

Tags	
	Anova p-value ≤ 0.05
	power>0.8
	q<0.05

P02675

Fibrinogen beta chain OS=Homo sapiens OX=9606 GN=FGB PE=1 SV=2
8 peptides

Sequence	Peptide Ion	Score	Hits	Mass	Charge	Tags	Conflicts	Modifications	In quantitation	Drift time (ms)	Average Normalised Abundances	
											CTR	M[illegible]
DNDGWLTSDPR	3633	5.93	1	1274.5767	2		0		yes	5.06	387.17	[illegible]
DNDGWLTSDPRK	4556	4.73	1	1402.6506	2		0		no	5.26	213.60	[illegible]
ECEEIIR	1673	7.44	2	947.4410	2		0	[2] Carbamidomethyl C	yes	4.22	438.58	[illegible]
EEAPSLRPAPPPISGGGYR	4317	6.16	1	1949.9906	3		0		no	4.43	141.51	[illegible]
QDGSVDFGR	1426	7.72	2	979.4382	2		0		yes	4.02	1735.88	2[illegible]
QGFGNVATNTDGK	4570	6.71	2	1307.6115	2		0		no	5.12	207.36	[illegible]
TPCTVSCNIPVVSGK	2956	7.23	2	1617.7835	2		0	[3] Carbamidomethyl C|[7] Carbamidomethyl C	no	5.96	342.39	[illegible]
TPCTVSCNIPVVSGKECEEIIR	3461	6.44	1	2547.2020	3		0	[3] Carbamidomethyl C|[7] Carbamidomethyl C|[17] Carbamidomethyl C	no	5.75	250.06	[illegible]

Tags	
	Anova p-value ≤ 0.05
	power>0.8
	q<0.05

P50895

Basal cell adhesion molecule OS=Homo sapiens OX=9606 GN=BCAM PE=1 SV=2
8 peptides

Sequence	Peptide Ion	Score	Hits	Mass	Charge	Tags	Conflicts	Modifications	In quantitation	Drift time (ms)	Average Normalised Abundances	
											CTR	MCD
AGAAGTAEATAR	3331	6.86	1	1045.5153	2		0		no	4.36	138.93	61.81
DGISCEASNPHGNKR	4156	5.32	1	1640.7353	3		0	[5] Carbamidomethyl C	yes	4.02	466.29	5.70
DGISCEASNPHGNKR	4367	5.32	1	1640.7366	4		0	[5] Carbamidomethyl C	yes	3.39	262.77	0.74

Sequence	Peptide Ion	Score	Hits	Mass	Charge	Tags	Conflicts	Modifications	In quantitation	Drift time (ms)	Average Normalised Abundances	
											CTR	MCD
GDGSPSPEYTLFR	4571	5.75	2	1424.6570	2		0		no	5.33	252.68	138.09
LSVPPLVEVMR	5236	5.21	1	1254.6915	2		0	[5] Hydroxyl DKNP	no	4.92	369.25	96.99
SPPYQLDSQGR	3836	6.61	2	1246.5915	2		0		yes	4.78	312.44	144.45
TAEIEPKADGSWR	5843	4.43	1	1458.6913	2		0		no	5.61	210.12	92.17
VAYLDPLELSEGK	2251	7.33	2	1432.7445	2		0		yes	5.96	460.51	264.60
VEDYDAADDVQLSK	9534	5.49	1	1566.7092	2		0		no	6.02	34.33	3.92

Tags	
	Anova p-value ≤ 0.05
	power>0.8
	q<0.05

P04083

Annexin A1 OS=Homo sapiens OX=9606 GN=ANXA1 PE=1 SV=2
7 peptides

Sequence	Peptide Ion	Score	Hits	Mass	Charge	Tags	Conflicts	Modifications	In quantitation	Drift time (ms)	Average Normalised Abundances	
											CTR	MCD
DITSDTSGDFR	3618	7.05	2	1212.5390	2		0		yes	4.57	329.56	173.6
DLAKDITSDTSGDFR	6509	6.77	2	1639.7692	3		0		no	4.09	121.77	22.6
DLAKDITSDTSGDFR	9449	6.77	2	1639.7741	2		0		no	6.23	60.22	2.0
GDRSEDFGVNEDLADSDAR	5804	6.46	2	2066.8806	3		0		no	4.43	165.18	102.2
GLGTDEDTLIEILASR	8289	6.60	1	1701.8706	2		0		no	6.44	65.88	11.7
GLGTDEDTLIEILASR	13691	---	---	1701.8712	3		0		no	4.29	15.29	0.0
GVDEATIIDILTKR	5511	5.83	1	1542.8627	3		0		no	4.16	80.85	3.4
QQIKAAYLQETGKPLDETLK	2622	5.68	2	2273.2231	3		0		yes	5.61	777.71	1144.3
TPAQFDADELR	2876	6.98	2	1261.5967	2		0		yes	4.99	669.12	385.8

Tags	
	Anova p-value ≤ 0.05
	power>0.8
	q<0.05

P12036

Neurofilament heavy polypeptide OS=Homo sapiens OX=9606 GN=NEFH PE=1 SV=4
10 peptides

Sequence	Peptide Ion	Score	Hits	Mass	Charge	Tags	Conflicts	Modifications	In quantitation	Drift time (ms)	Average Normalised Abundances	
											CTR	MCD

Sequence	Peptide Ion	Score	Hits	Mass	Charge	Tags	Conflicts	Modifications	In quantitation	Drift time (ms)	Average Normalised Abundances	
											CTR	MCD
AKSPTK	303	5.51	1	630.3709	1		0		no	7.48	6124.43	1990.56
AKSPTK	4283	---	---	630.3700	2		0		no	2.91	234.67	29.66
EEEKPQEVKVK	93	4.12	1	1357.7065	3		0	[9] Hydroxyl DKNP	yes	3.74	4.46e+004	3.13e+004
EPSKPK	749	4.15	1	684.3830	2		0		no	3.46	895.62	2253.06
KEPAVEKPK	262	4.77	2	1052.6233	2		0	[1] Methyl K | [7] Methyl K	yes	4.50	2698.16	8516.42
+KEPAVEKPKESK	298	5.58	1	1428.7394	3		0	[N-term] Formyl K N-TERM | [1] Hydroxyl DKNP | [9] Hydroxyl DKNP	yes	4.09	3319.06	1.08e+004
LGAAR	394	5.29	2	486.2920	1		0		no	6.02	2545.26	3295.39
SAQEEITEYR	7805	5.70	1	1224.5521	2		0		no	4.85	51.68	3.11
SPPAEEAASPEKEAK	10266	5.31	1	1553.7546	2		0	[12] Methyl K	no	6.02	43.34	1.84
SSSTDQKDSKPPEK	8707	5.16	1	1548.7170	2		0	[5] Hydroxyl DKNP	no	5.82	67.77	4.68
VEVKEDAKPK	928	4.55	1	1141.6194	2		0		no	4.71	3499.85	590.70
VEVKEDAKPK	5179	---	---	1141.6188	3		0		no	3.32	356.51	0.68

Tags	
	Anova p-value ≤ 0.05
	power>0.8
	q<0.05

P61981

14-3-3 protein gamma OS=Homo sapiens OX=9606 GN=YWHAG PE=1 SV=2
7 peptides

Sequence	Peptide Ion	Score	Hits	Mass	Charge	Tags	Conflicts	Modifications	In quantitation	Drift time (ms)	Average Normalised Abundances	
											CTR	MCD
LAEQAERYDDMAAAMK	3949	4.37	1	1825.8508	3		0	[16] Methyl K	no	4.43	417.79	89.15
NLLSVAYK	1212	7.36	4	906.5175	2		0		yes	3.88	926.48	472.71
NVTELNEPLSNEER	5797	7.49	4	1642.7735	2		0		no	6.09	233.97	53.49
RATVVESSEK	1695	4.32	1	1104.5655	2		0		yes	4.16	4006.31	1105.21
SSWRVISSIEQK	5693	5.48	1	1418.7572	3		0		no	4.02	177.79	38.92
VISSIEQK	997	7.36	4	902.5048	2		0		yes	3.81	1511.27	722.01
YLAEVATGEKR	6224	6.76	1	1235.6478	3		0		no	3.60	113.32	18.64
YLAEVATGEKR	7134	6.76	1	1235.6267	2		0		no	4.78	136.28	11.08

Tags	
	Anova p-value ≤ 0.05
	power>0.8
	q<0.05

Q5TZA2

Rootletin OS=Homo sapiens OX=9606 GN=CROCC PE=1 SV=1
10 peptides

Sequence	Peptide Ion	Score	Hits	Mass	Charge	Tags	Conflicts	Modifications	In quantitation	Drift time (ms)	Average Normalised Abundances	
											CTR	MCD
ALAQLEAEKR	4323	4.47	1	1127.6330	2		0		no	4.64	288.79	372[illegible]
DEGLLLAESEK	2910	4.57	1	1232.6166	2		0	[1] Hydroxyl DKNP|[11] Methyl K	no	4.92	807.06	388[illegible]
DELRTQTSALNR	9102	4.43	1	1418.7019	2		0	[11] Hydroxyl DKNP	no	5.40	44.18	[illegible]
DLAQQQMQSDLDK	255	5.18	1	1518.6976	2		0		yes	5.68	7456.84	3937[illegible]
DLAQQQMQSDLDK	1577	4.19	1	1534.6900	2		0	[1] Hydroxyl DKNP	yes	5.82	1827.52	615[illegible]
EELSEEIAALQQER	10416	4.18	1	1643.7720	2		0		no	6.23	99.51	[illegible]
QLEQLEGK	9480	4.60	1	943.4936	2		0		no	3.95	149.96	40[illegible]
QMKMLDSENTR	3544	4.54	1	1383.6081	2		0	[2] Oxidation M|[3] Hydroxyl DKNP	no	5.33	228.47	139[illegible]
REEESFNAYFSNEHSR	1929	4.55	1	2000.8674	3		0		yes	4.36	681.92	841[illegible]
REEESFNAYFSNEHSR	3720	4.55	1	2000.8717	2		0		yes	6.93	274.99	262[illegible]
SVDGRLSGVQAELALQEESVR	4150	4.53	1	2242.1252	3		0		no	5.06	331.66	166[illegible]

Tags	
	Anova p-value ≤ 0.05
	power>0.8
	q<0.05

Q06830

Peroxiredoxin-1 OS=Homo sapiens OX=9606 GN=PRDX1 PE=1 SV=1
6 peptides

Sequence	Peptide Ion	Score	Hits	Mass	Charge	Tags	Conflicts	Modifications	In quantitation	Drift time (ms)	Average Normalised Abundances	
											CTR	MCD
ATAVMPDGQFK	4705	6.55	3	1179.5581	2		0	[5] Oxidation M	no	4.64	346.98	35.76
GLFIIDDK	2644	7.30	4	919.5011	2		0		no	3.95	414.10	132.37
GLFIIDDKGILR	6194	6.79	3	1358.7879	3		0		no	4.02	330.76	20.22
GLFIIDDKGILR	8805	6.76	1	1358.7890	2		0		no	5.68	201.80	20.06
LVQAFQFTDK	2377	7.07	4	1195.6202	2		0		yes	4.85	668.76	343.30
SVDETLR	1545	7.65	2	818.4113	2		0		yes	3.67	1658.26	638.13
SVDETLR	13390	---	---	818.3997	1		0		yes	9.14	28.42	0.00
TIAQDYGVLK	1384	7.48	4	1106.5940	2		0		yes	4.57	1157.33	589.54

Tags	
	Anova p-value ≤ 0.05

	power>0.8
	q<0.05

Q05682

Caldesmon OS=Homo sapiens OX=9606 GN=CALD1 PE=1 SV=3
8 peptides

Sequence	Peptide Ion	Score	Hits	Mass	Charge	Tags	Conflicts	Modifications	In quantitation	Drift time (ms)	Average Normalised Abundances	
											CTR	MCD
ASVDTKEAEGAPQVEAGK	11103	5.45	1	1785.8726	3		0		no	4.02	42.74	10.77
EAEGAPQVEAGK	6858	6.04	2	1184.5655	2		0		no	4.71	96.23	8.36
LQEALER	1120	6.90	2	857.4604	2		0		yes	3.67	1145.68	950.53
NDDDEEEAARER	7694	5.30	1	1447.5803	3		0		no	3.88	57.01	39.96
QKEFDPTITDASLSLPSR	5915	6.20	2	2004.0081	3		0		no	4.50	125.87	27.73
QQEAALELEELKK	3589	6.32	2	1527.8031	3		0		yes	3.95	194.13	298.22
RLQEALER	8987	5.70	1	1013.5570	2		0		no	4.50	136.27	47.31
VLEEEEQR	3724	7.16	2	1030.4929	2		0		yes	4.22	241.37	73.46

Tags	
	Anova p-value ≤ 0.05
	power>0.8
	q<0.05

Q03252

Lamin-B2 OS=Homo sapiens OX=9606 GN=LMNB2 PE=1 SV=4
9 peptides

Sequence	Peptide Ion	Score	Hits	Mass	Charge	Tags	Conflicts	Modifications	In quantitation	Drift time (ms)	Average Normalised Abundances	
											CTR	MCD
AAATMATPLPGR	761	5.32	1	1155.6134	2		0		yes	4.64	1440.76	970.71
AGGPATPLSPTR	5125	5.79	2	1123.5881	2		0		no	4.36	226.16	85.63
ALELENDRLLLK	6192	4.96	1	1425.8178	2		0		no	5.89	81.88	3.40
ATSSSSGSLSATGR	7930	6.25	1	1267.5914	2		0		no	4.85	67.03	4.16
CQSLQEELDFR	2365	4.17	1	1423.6611	3		0	[1] Carbamidomethyl C	yes	4.29	454.40	250.97
EGELTVAQGR	5130	6.28	1	1058.5284	2		0		no	4.16	258.24	105.67
ELEEAMAGER	2775	4.05	1	1133.5239	2		0		yes	4.43	746.99	635.14
ELEEAMAGERDK	8910	4.47	1	1376.6386	2		0		no	5.33	97.29	13.92
GLESDVAELR	4114	6.33	2	1087.5599	2		0		no	4.43	449.65	165.66

Tags	
	Anova p-value ≤ 0.05

	power>0.8
	q<0.05

P02671

Fibrinogen alpha chain OS=Homo sapiens OX=9606 GN=FGA PE=1 SV=2
8 peptides

Sequence	Peptide Ion	Score	Hits	Mass	Charge	Tags	Conflicts	Modifications	In quantitation	Drift time (ms)	Average Normalised Abundances	
											CTR	MCD
ALTDMPQMR	3497	5.17	1	1093.4943	2		0	[5] Oxidation M|[6] Hydroxyl DKNP	yes	4.36	452.60	496.64
GDFSSANNRDNTYNR	9843	5.52	1	1729.7351	3		0		no	3.95	64.79	10.81
GLIDEVNQDFTNR	5971	6.05	2	1519.7253	2		0		no	5.89	129.92	24.36
GSESGIFTNTK	1331	7.00	2	1139.5449	2		0		yes	4.57	1005.31	1093.89
MELERPGGNEITR	1786	5.89	2	1516.7313	3		0	[1] Oxidation M	yes	4.16	1111.87	1061.12
QFTSSTSYNR	9653	5.30	1	1189.5366	2		0		no	4.71	102.75	20.17
QFTSSTSYNRGDSTFESK	4811	6.38	2	2040.9033	3		0		no	4.78	77.82	130.34
QLEQVIAK	2607	5.99	2	927.5359	2		0		no	4.16	191.80	83.23

Tags	
	Anova p-value ≤ 0.05
	power>0.8
	q<0.05

P04004

Vitronectin OS=Homo sapiens OX=9606 GN=VTN PE=1 SV=1
8 peptides

Sequence	Peptide Ion	Score	Hits	Mass	Charge	Tags	Conflicts	Modifications	In quantitation	Drift time (ms)	Average Normalised Abundances	
											CTR	MCD
DVWGIEGPIDAAFTR	5421	6.67	2	1645.8072	2		0		no	6.23	320.04	[illegible]
DWHGVPGQVDAAMAGR	3923	5.11	1	1681.7622	3		0	[13] Oxidation M	no	4.16	281.66	[illegible]
FEDGVLDPDYPR	2451	6.34	3	1421.6537	2		0		yes	5.47	1196.43	[illegible]
FEDGVLDPDYPR	5255	5.59	1	1421.6324	2		0		no	5.54	593.53	[illegible]
GQYCYELDEK	2044	6.04	4	1303.5381	2		0	[4] Carbamidomethyl C	no	5.12	826.77	[illegible]
QPQFISR	2137	5.58	4	874.4623	2		0		no	3.81	1040.51	[illegible]
RVDTVD	2819	0.00	3	685.3403	1		0		no	7.48	451.97	[illegible]
RVDTVD	10276	---	---	685.3405	2		0		no	3.05	43.12	[illegible]
RVDTVDPPYPR	2215	5.96	4	1313.6673	2		0		yes	5.19	1415.72	[illegible]

Sequence	Peptide Ion	Score	Hits	Mass	Charge	Tags	Conflicts	Modifications	In quantitation	Drift time (ms)	Average Normalised Abundances	
											CTR	MCD
RVDTVDPPYPR	3398	5.96	4	1313.6706	3		0		yes	4.02	445.77	
VDTVDPPYPR	139	5.35	3	1157.5706	2		0		yes	4.64	8445.72	2.21

Tags	
	Anova p-value ≤ 0.05
	power>0.8
	q<0.05

P08758

Annexin A5 OS=Homo sapiens OX=9606 GN=ANXA5 PE=1 SV=2
7 peptides

Sequence	Peptide Ion	Score	Hits	Mass	Charge	Tags	Conflicts	Modifications	In quantitation	Drift time (ms)	Average Normalised Abundances	
											CTR	MCD
GLGTDEESILTLLTSR	6384	6.94	1	1703.8923	2		0		no	6.58	69.13	12.
GLGTDEESILTLLTSR	11578	---	---	1703.8985	3		0		no	4.43	20.33	1.
GTVTDFPGFDER	2122	7.44	4	1339.6069	2		0		yes	5.06	633.20	377.
GTVTDFPGFDERADAETLR	3630	5.78	2	2095.9790	3		0		no	4.64	526.26	282.
QEISAAFK	327	5.38	2	892.4683	2		0		yes	4.09	2253.81	6711.
QEISAAFK	14554	---	---	892.4670	1		0		yes	10.32	8.68	57.
SEIDLFNIR	3557	7.13	2	1105.5797	2		0		no	4.71	297.68	58.
SEIDLFNIRK	6947	6.39	1	1233.6673	3		0		no	3.46	86.26	14.
VLTEIIASR	712	7.20	4	1000.5874	2		0		yes	4.22	1834.02	1202.

Tags	
	Anova p-value ≤ 0.05
	power>0.8
	q<0.05

P14618

Pyruvate kinase PKM OS=Homo sapiens OX=9606 GN=PKM PE=1 SV=4
8 peptides

Sequence	Peptide Ion	Score	Hits	Mass	Charge	Tags	Conflicts	Modifications	In quantitation	Drift time (ms)	Averag Norma Abund
											CTR
APIIAVTR	2865	6.52	2	839.5210	2		0		no	3.81	364.4
GDLGIEIPAEK	1421	7.13	4	1140.5988	2		0		yes	4.57	1654.1
ITLDNAYMEK	328	3.73	1	1196.5793	2		0		yes	4.57	3826.0
SVETLK	921	5.52	1	675.3786	1		0		no	7.89	798.7

Sequence	Peptide Ion	Score	Hits	Mass	Charge	Tags	Conflicts	Modifications	In quantitation	Drift time (ms)	Averag Norma Abund
											CTR
TATESFASDPILYRPVAVALDTK	8069	6.46	2	2464.2747	3		0		no	5.26	133.7
TATESFASDPILYRPVAVALDTKGPEIR	7034	5.40	1	3016.5976	4		0		no	5.89	169.3
VFLAQKMMIGR	1178	5.63	1	1308.6922	2		0	[7] Oxidation M	yes	5.26	1554.9
VNFAMNVGKAR	7660	5.72	1	1219.6514	2		0	[9] Methyl K	no	4.85	158.1

Tags	
	Anova p-value ≤ 0.05
	power>0.8
	q<0.05

P09467

Fructose-1_6-bisphosphatase 1 OS=Homo sapiens OX=9606 GN=FBP1 PE=1 SV=5
9 peptides

Sequence	Peptide Ion	Score	Hits	Mass	Charge	Tags	Conflicts	Modifications	In quantitation	Drift time (ms)	Average Norn Abundances	
											CTR	M
AGIAHLYGIAGSTNVTGDQVK	4981	4.78	2	2071.0676	3		0		no	4.50	311.56	
APVILGSPDDVLEFLK	6986	5.42	1	1711.9264	2		0		no	7.06	62.51	
DFDPAVTEYIQR	2685	6.30	2	1452.6877	2		0		no	5.61	407.48	
EAVLDVIPTDIHQR	4602	6.28	2	1604.8505	3		0		no	4.36	199.37	
FVMEEGR	110	6.12	1	866.3985	2		0		yes	3.74	1.10e+004	3.
FVMEEGR	3851	5.59	1	882.3919	2		0	[3] Oxidation M	no	3.67	413.54	
+KLDVLSNDLVMNMLK	1486	5.11	1	1759.9203	3		0	[N-term] Formyl K N-TERM	yes	5.06	709.67	
STDEPSEKDALQPGR	3857	6.20	2	1628.7619	3		0		no	4.16	550.58	
VMEEGR	96	0.00	1	719.3257	1		0		yes	7.76	6972.39	2.

Tags	
	Anova p-value ≤ 0.05
	power>0.8
	q<0.05

Q07157

Tight junction protein ZO-1 OS=Homo sapiens OX=9606 GN=TJP1 PE=1 SV=3
9 peptides

Sequence	Peptide Ion	Score	Hits	Mass	Charge	Tags	Conflicts	Modifications	In quantitation	Drift time (ms)	Average Normalised Abundances	
											CTR	MCD
AEQLASVQYTLPK	6552	4.83	1	1446.7580	2		0		no	5.89	83.43	4.28

Sequence	Peptide Ion	Score	Hits	Mass	Charge	Tags	Conflicts	Modifications	In quantitation	Drift time (ms)	Average Normalised Abundances	
											CTR	MCD
DGNIQEGDVVLK	3892	5.11	1	1285.6547	2		0		yes	5.06	978.21	330.74
EEPDIYQIAK	6211	4.87	1	1204.6002	2		0		no	4.71	290.66	79.14
EISQDSLAAR	3364	5.56	1	1088.5510	2		0		yes	4.36	881.07	209.76
FEEPAPLSYDSRPR	2973	5.60	1	1662.8026	3		0		yes	4.57	251.04	116.92
IPEPQKPQLKPPEDIVR	8508	4.62	1	1983.0986	4		0		no	4.29	74.83	5.82
LASHIFVK	4434	4.53	1	913.5211	1		0		no	9.97	62.49	1.04
QYFEQYSR	8186	5.16	1	1119.5099	2		0		no	4.57	128.15	77.13
YESSSYTDQFSR	8343	5.09	1	1468.6211	2		0		no	5.40	70.58	19.02

Tags	
	Anova p-value ≤ 0.05
	power>0.8
	q<0.05

O14745

Na(+)/H(+) exchange regulatory cofactor NHE-RF1 OS=Homo sapiens OX=9606 GN=SLC9A3R1 PE=1 SV=4
7 peptides

Sequence	Peptide Ion	Score	Hits	Mass	Charge	Tags	Conflicts	Modifications	In quantitation	Drift time (ms)	Average Normalised Abundances	
											CTR	MCD
EALAEAALESPRPALVR	2123	6.83	4	1791.9774	3		0		no	4.92	901.40	436.38
KGPSGYGFNLHSDK	2169	5.48	3	1505.7102	2		0		no	5.54	839.58	427.41
KNELFSNL	1977	5.71	1	963.5011	2		0		no	3.95	724.90	525.61
LLVVDPETDEQLQK	2500	7.41	4	1625.8491	2		0		no	6.23	717.56	368.67
LVEPGSPAEK	1304	7.31	4	1025.5387	2		0		yes	4.09	1097.73	393.56
QHGDVVSAIRAGGDETK	438	4.89	1	1754.8483	3		0	[14] Hydroxyl DKNP	yes	4.36	4205.19	3655.05
QHGDVVSAIRAGGDETK	5993	---	---	1754.8395	2		0	[14] Hydroxyl DKNP	yes	6.86	130.49	53.38
SVDPDSPAEASGLR	1230	7.56	6	1399.6568	2		0		yes	5.19	1175.92	655.72

Tags	
	Anova p-value ≤ 0.05
	power>0.8
	q<0.05

P25705

ATP synthase subunit alpha_ mitochondrial OS=Homo sapiens OX=9606 GN=ATP5F1A PE=1 SV=1
7 peptides

Sequence	Peptide Ion	Score	Hits	Mass	Charge	Tags	Conflicts	Modifications	In quantitation	Drift time (ms)	Average Normalised Abundances	
											CTR	MCD
AVDSLVPIGR	1413	7.09	4	1025.5879	2		0		yes	4.22	767.34	560.64
ILGADTSVDLEETGR	4819	6.64	4	1574.7707	2		0		no	5.89	371.32	244.97
QMSLLLR	3378	5.43	1	875.4873	2		0	[2] Oxidation M	no	4.16	249.24	107.16
TGAIVDVPVGEELLGR	1735	7.04	6	1623.8847	2		0		yes	6.30	1337.53	933.62
TGTAEMSSILEER	5361	5.52	1	1438.6599	2		0	[6] Oxidation M	no	5.54	210.65	113.63
VVDALGNAIDGK	2587	6.62	4	1170.6139	2		0		yes	4.78	559.12	337.82
VVDALGNAIDGKGPIGSK	3675	6.08	4	1709.9290	3		0		no	4.36	545.61	288.53

Tags	
	Anova p-value ≤ 0.05
	power>0.8
	q<0.05

P04792

Heat shock protein beta-1 OS=Homo sapiens OX=9606 GN=HSPB1 PE=1 SV=2
6 peptides

Sequence	Peptide Ion	Score	Hits	Mass	Charge	Tags	Conflicts	Modifications	In quantitation	Drift time (ms)	Average Normalised Abundances	
											CTR	MCD
AQLGGPEAAK	2734	6.25	2	940.4847	2		0		no	3.95	723.42	567.51
AQLGGPEAAKSDETAAK	2502	6.79	5	1642.8162	3		0		no	4.36	1244.79	564.61
LATQSNEITIPVTFESR	1800	7.78	6	1904.9792	2		0		yes	7.06	1087.12	694.65
LFDQAFGLPR	2746	7.20	4	1162.6075	2		0		no	4.64	406.82	253.04
QLSSGVSEIR	677	7.63	6	1074.5568	2		0		yes	4.43	2606.09	1483.54
VSLDVNHFAPDELTVK	1592	7.18	5	1782.9146	3		0		yes	4.22	1623.92	744.54

Tags	
	Anova p-value ≤ 0.05
	power>0.8
	q<0.05

Q5T749

Keratinocyte proline-rich protein OS=Homo sapiens OX=9606 GN=KPRP PE=1 SV=1
7 peptides

Sequence	Peptide Ion	Score	Hits	Mass	Charge	Tags	Conflicts	Modifications	In quantitation	Drift time (ms)	Av No Ab
											CT
CPVEIPPIR	2296	6.89	2	1079.5789	2		0		yes	4.64	36[illegible]

Sequence	Peptide Ion	Score	Hits	Mass	Charge	Tags	Conflicts	Modifications	In quantitation	Drift time (ms)	Av No Ab
											CT
								[1] Carbamidomethyl C			
FEPCSSSYLPLRPSEGFPNYCTPPRR	4307	4.89	1	3113.4357	4		0	[4] Carbamidomethyl C|[21] Carbamidomethyl C	no	5.75	8[illegible]
IEISSPCCPR	1050	7.02	2	1217.5588	2		0	[7] Carbamidomethyl C|[8] Carbamidomethyl C	yes	4.71	27[illegible]
RLDQCPESPLQR	1244	5.53	1	1497.7357	3		0	[5] Carbamidomethyl C	no	3.67	18[illegible]
RLDQCPESPLQR	6910	---	---	1497.7330	2		0	[5] Carbamidomethyl C	no	5.61	2[illegible]
SEPIYNSR	5551	6.45	2	964.4648	2		0		no	4.02	6[illegible]
SPSPCWGPNPVPYPGDLGCHESSPHR	655	5.16	1	2918.2421	4		0	[5] Carbamidomethyl C|[9] Hydroxyl DKNP|[19] Carbamidomethyl C|[24] Hydroxyl DKNP	yes	5.33	15[illegible]
SPSPCWGPNPVPYPGDLGCHESSPHR	3758	---	---	2918.2487	3		0	[5] Carbamidomethyl C|[9] Hydroxyl DKNP|[19] Carbamidomethyl C|[24] Hydroxyl DKNP	yes	5.96	1[illegible]
TSFSPCVPQCQTQGSYGSFTEQHR	4034	6.78	2	2788.1870	3		0	[6] Carbamidomethyl C|[10] Carbamidomethyl C	no	5.61	10[illegible]

Tags	
	Anova p-value ≤ 0.05
	power>0.8
	q<0.05

Q8N3V7

Synaptopodin OS=Homo sapiens OX=9606 GN=SYNPO PE=1 SV=2
8 peptides

Sequence	Peptide Ion	Score	Hits	Mass	Charge	Tags	Conflicts	Modifications	In quantitation	Drift time (ms)	Average Normalised Abundances	
											CTR	MC
AASPAKPSSLDLVPNLPK	3657	5.06	1	1803.9882	3		0		no	4.92	164.91	3[illegible]
CSTLLIDK	5631	3.48	1	948.4811	1		0	[1] Carbamidomethyl C	no	10.46	46.32	[illegible]
SPPSYSVLYPSSDPK	4093	5.77	2	1622.7767	2		0		yes	6.37	184.78	8[illegible]
TARPFGIQAPGGTSQMER	3710	5.04	2	1918.9311	3		0	[16] Oxidation M	no	4.16	266.12	3[illegible]

Sequence	Peptide Ion	Score	Hits	Mass	Charge	Tags	Conflicts	Modifications	In quantitation	Drift time (ms)	Average Normalised Abundances	
											CTR	MC
TGILEESMAR	1991	5.88	2	1121.5409	2		0	[8] Oxidation M	yes	4.43	1024.95	[illegible]
VASEEEEVPLVVYLK	7963	5.13	2	1702.8928	2		0		no	6.72	61.67	[illegible]
VTPNPDLLDLVQTADEK	5654	5.63	1	1866.9460	2		0		no	7.13	106.98	[illegible]
VTPNPDLLDLVQTADEK	10317	5.55	1	1866.9537	2		0		no	7.27	59.26	[illegible]
YVIESSSHTPELAR	2641	5.06	1	1587.8004	3		0		yes	4.02	299.81	[illegible]

Tags	
	Anova p-value ≤ 0.05
	power>0.8
	q<0.05

P19012

Keratin_ type I cytoskeletal 15 OS=Homo sapiens OX=9606 GN=KRT15 PE=1 SV=3
7 peptides

Sequence	Peptide Ion	Score	Hits	Mass	Charge	Tags	Conflicts	Modifications	In quantitation	Drif tim (ms
CEMEAQNQEYK	2206	---	---	1442.5842	3		0	[1] Carbamidomethyl C|[11] Methyl K	yes	3.7
CEMEAQNQEYK	5576	5.79	1	1442.5890	2		0	[1] Carbamidomethyl C|[11] Methyl K	yes	5.3
GGSLLAGGGGFGGGSLSGGGGSRSISASSAR	5390	5.23	1	2580.2329	3		0		no	5.1
LKYENELALR	9294	5.78	1	1247.6844	3		0		no	3.7
QGVEADINGLR	3677	5.33	1	1170.6026	2		0		yes	4.7
RDVEAWFFSK	4099	5.43	1	1299.6248	2		0	[10] Hydroxyl DKNP	no	5.6
TIEELRDK	5932	6.21	1	1002.5253	2		0		no	4.2
VILEIDNAR	3327	6.56	2	1041.5803	2		0		yes	4.5

Tags	
	Anova p-value ≤ 0.05
	power>0.8
	q<0.05

P35749

Myosin-11 OS=Homo sapiens OX=9606 GN=MYH11 PE=1 SV=3
8 peptides

Sequence	Peptide Ion	Score	Hits	Mass	Charge	Tags	Conflicts	Modifications	In quantitation	Drift time (ms)	Average Normalised Abundances	
											CTR	MC
ALEEALEAKEELER	13277	4.23	1	1628.8416	2		0		no	6.44	26.95	
DADFNGTKASE	4624	4.69	1	1153.4900	2		0		yes	4.57	1153.90	4
ENADLAGELR	5835	4.81	1	1086.5467	2		0		no	4.36	470.16	11
IAQLEEQVEQEAR	1180	5.53	1	1541.7683	3		0		yes	3.81	1384.14	20
LQAQMKDFQRELEDAR	5667	4.98	1	2006.9756	3		0	[6] Methyl K|[14] Hydroxyl DKNP	no	4.36	135.93	2
QQKAENELK	3929	5.31	1	1102.5534	2		0	[6] Hydroxyl DKNP	no	4.57	326.93	12
TEFSIIHYAGKVDYNASAWLTK	4494	4.98	1	2529.2386	3		0	[15] Hydroxyl DKNP	no	5.12	144.51	6
VIQYLAVVASSHK	831	5.45	1	1413.7897	3		0		yes	3.95	2067.36	151

Tags	
	Anova p-value ≤ 0.05
	power>0.8
	q<0.05

P02042

Hemoglobin subunit delta OS=Homo sapiens OX=9606 GN=HBD PE=1 SV=2
5 peptides

Sequence	Peptide Ion	Score	Hits	Mass	Charge	Tags	Conflicts	Modifications	In quantitation	Drift time (ms)	Average N Abundanc
											CTR
FFESFGDLSSPDAVMGNPK	3782	6.49	2	2059.9128	2		0	[11] Hydroxyl DKNP	no	7.62	338.8
FFESFGDLSSPDAVMGNPK	7910	---	---	2059.9136	3		0	[11] Hydroxyl DKNP	no	5.12	148.9
FFESFGDLSSPDAVMGNPK	183	6.13	1	2073.9363	2		0	[18] Hydroxyl DKNP|[19] Methyl K	yes	7.62	1.40e+00
FFESFGDLSSPDAVMGNPK	434	---	---	2073.9373	3		0	[18] Hydroxyl DKNP|[19] Methyl K	yes	5.19	5288.6
TAVNALWGK	3245	7.50	1	958.5111	2		0		no	3.88	365.2
VNVDAVGGEALGR	695	7.76	7	1255.6506	2		0		yes	4.92	2011.8
VNVDAVGGEALGRLLVVYPWTQR	277	5.58	1	2543.3871	5		0	[2] Hydroxyl DKNP|[19] Hydroxyl DKNP	yes	4.16	4515.2
VNVDAVGGEALGRLLVVYPWTQR	283	5.58	1	2543.3778	4		0	[2] Hydroxyl DKNP|[19] Hydroxyl DKNP	yes	4.57	3162.6
VNVDAVGGEALGRLLVVYPWTQR	2352	---	---	2543.3711	6		0	[2] Hydroxyl DKNP|[19] Hydroxyl DKNP	yes	3.74	131.8
VNVDAVGGEALGRLLVVYPWTQR	7124	---	---	2543.3685	3		0	[2] Hydroxyl DKNP|[19] Hydroxyl DKNP	yes	5.75	8.31

Tags

	Anova p-value ≤ 0.05
	power>0.8
	q<0.05

P08473

Neprilysin OS=Homo sapiens OX=9606 GN=MME PE=1 SV=2
7 peptides

Sequence	Peptide Ion	Score	Hits	Mass	Charge	Tags	Conflicts	Modifications	In quantitation	Drift time (ms)	Average Normalised Abundances	
											CTR	MCD
ALYGTTSETATWR	1004	3.77	1	1455.6834	3		0		yes	3.95	782.26	2734.
ALYGTTSETATWR	5997	6.59	2	1455.6972	2		0		no	5.61	267.06	46.
AYQNYIKK	1539	4.24	1	1042.5411	2		0	[8] Hydroxyl DKNP	yes	4.43	524.11	1262.
+KNGEEK	5062	---	---	731.3422	2		0	[N-term] Formyl K N-TERM	no	3.19	65.43	207.
LGLPSRDYYECTGIYK	7562	6.17	1	1933.9243	3		0	[11] Carbamidomethyl C	no	4.36	107.50	2.
LIQNMDATTEPCTDFFK	7461	5.60	1	2045.9112	2		0	[4] Hydroxyl DKNP|[12] Carbamidomethyl C	no	7.83	152.05	7.
LIQNMDATTEPCTDFFK	8355	---	---	2045.9243	3		0	[4] Hydroxyl DKNP|[12] Carbamidomethyl C	no	4.78	102.17	31.
LYVEAAFAGESK	6312	4.93	1	1283.6183	2		0		no	4.85	145.84	123.
TEDIVAVQK	1943	7.33	3	1001.5308	2		0		yes	4.09	1845.14	859.

Tags	
	Anova p-value ≤ 0.05
	power>0.8
	q<0.05

Q9NZN3

EH domain-containing protein 3 OS=Homo sapiens OX=9606 GN=EHD3 PE=1 SV=2
7 peptides

Sequence	Peptide Ion	Score	Hits	Mass	Charge	Tags	Conflicts	Modifications	In quantitation	Drift time (ms)	Average Normalised Abundances	
											CTR	MCD
ADQIETQQLMR	8046	5.25	1	1347.6418	2		0	[10] Oxidation M	no	5.33	109.39	4
ELVNNLAEIYGR	8934	5.16	1	1389.7155	2		0		no	5.61	43.88	0
IVNTPEVIR	9758	5.27	1	1039.5832	2		0		no	4.16	25.06	13
LEGHELPNELPAHLLPPSK	243	3.75	1	2090.0874	4		0		yes	4.71	4931.11	1.89e+(
LEGHELPNELPAHLLPPSK	297	3.75	1	2090.0832	3		0		yes	5.68	4095.68	1.60e+(
LFEAEEQDLFR	7618	5.38	1	1395.6688	2		0		no	5.61	96.85	29

Sequence	Peptide Ion	Score	Hits	Mass	Charge	Tags	Conflicts	Modifications	In quantitation	Drift time (ms)	Average Normalised Abundances	
											CTR	MCD
MQDQLQAQDFSK	5845	5.70	1	1453.6555	2		0	[1] Oxidation M	yes	5.54	213.07	35
MQDQLQAQDFSK	6688	4.33	1	1453.6385	2		0	[3] Hydroxyl DKNP	yes	5.40	150.38	29

Tags	
	Anova p-value ≤ 0.05
	power>0.8
	q<0.05

P60660

Myosin light polypeptide 6 OS=Homo sapiens OX=9606 GN=MYL6 PE=1 SV=2
6 peptides

Sequence	Peptide Ion	Score	Hits	Mass	Charge	Tags	Conflicts	Modifications	In quantitation	Drift time (ms)	Average Normalised Abundances	
											CTR	MCD
ALGQNPTNAEVLK	305	8.31	4	1353.7252	2		0		yes	5.47	2869.51	3518.18
EAFQLFDR	379	6.02	2	1024.5000	2		0		yes	4.29	1270.61	3806.29
EAFQLFDR	895	8.09	3	1024.4996	2		0		yes	4.22	1172.08	2643.46
EAFQLFDRTGDGK	4275	4.00	1	1496.7462	2		0	[13] Methyl K	no	5.89	376.05	25.27
HVLVTLGEK	1210	5.31	1	994.5798	2		0		no	4.36	717.67	1685.33
HVLVTLGEK	3932	6.67	1	994.5708	2		0		no	4.36	439.55	449.88
ILYSQCGDVMR	2990	5.75	1	1340.6000	2		0	[6] Carbamidomethyl C	no	5.33	431.03	161.94
VLGNPKSDEMNVK	3129	5.60	1	1443.7201	3		0	[13] Methyl K	no	3.88	977.86	169.21

Tags	
	Anova p-value ≤ 0.05
	power>0.8
	q<0.05

Q93088

Betaine--homocysteine S-methyltransferase 1 OS=Homo sapiens OX=9606 GN=BHMT PE=1 SV=2
6 peptides

Sequence	Peptide Ion	Score	Hits	Mass	Charge	Tags	Conflicts	Modifications	In quantitation	Drift time (ms)	Average Norm Abundances	
											CTR	M
AGPWTPEAAVEHPEAVR	3220	6.58	3	1815.8924	3		0		no	4.71	747.07	
AIAEELAPER	425	7.77	4	1097.5733	2		0		yes	4.43	3713.82	
EAYNLGVR	1219	6.92	2	920.4698	2		0		yes	3.88	1843.53	
GNYVLEK	4775	6.27	1	821.4275	2		0		no	3.60	228.55	

Sequence	Peptide Ion	Score	Hits	Mass	Charge	Tags	Conflicts	Modifications	In quantitation	Drift time (ms)	Average Norm Abundances	
											CTR	M
ISGQEVNEAACDIAR	6970	6.65	2	1631.7520	2		0	[11] Carbamidomethyl C	no	6.02	155.48	
WDIQKYAR	86	4.10	1	1078.5604	2		0		yes	4.43	1.05e+004	3.

Tags	
	Anova p-value ≤ 0.05
	power>0.8
	q<0.05

Q03154

Aminoacylase-1 OS=Homo sapiens OX=9606 GN=ACY1 PE=1 SV=1
7 peptides

Sequence	Peptide Ion	Score	Hits	Mass	Charge	Tags	Conflicts	Modifications	In quantitation	Drift time (ms)	Average Normalised Abundances	
											CTR	MCD
AVGVPALGFSPMNR	2025	4.96	1	1414.7428	2		0		no	5.61	1374.54	125.40
AVGVPALGFSPMNR	2072	6.39	2	1430.7313	2		0	[11] Hydroxyl DKNP	yes	5.47	1631.07	267.31
DIYTR	2623	0.00	1	666.3335	1		0		no	7.55	379.81	67.49
EGSVTSVNLTK	1318	7.07	2	1133.5791	2		0		yes	4.57	1269.32	301.63
GPEEEHPSVTLFR	6088	5.52	2	1496.7265	3		0		no	4.22	163.50	66.81
GVDIYTR	3180	6.97	2	822.4260	2		0		no	3.60	403.39	144.39
LLPALASVPALPSDS	1009	7.06	2	1449.8075	2		0		yes	5.75	1953.97	682.16

Tags	
	Anova p-value ≤ 0.05
	power>0.8
	q<0.05

P62937

Peptidyl-prolyl cis-trans isomerase A OS=Homo sapiens OX=9606 GN=PPIA PE=1 SV=2
5 peptides

Sequence	Peptide Ion	Score	Hits	Mass	Charge	Tags	Conflicts	Modifications	In quantitation	Drift time (ms)	Average Normalised Abundances	
											CTR	MCD
EGMNIVEAMER	4234	5.34	1	1309.5644	2		0	[3] Oxidation M| [9] Oxidation M	yes	5.06	865.75	76.36
KITIADCGQLE	2844	5.72	1	1246.6189	2		0	[7] Carbamidomethyl C	no	4.92	443.99	224.75
SIYGEKFEDENFILK	1613	4.79	1	1830.9024	3		0		yes	4.36	1007.92	414.46
SIYGEKFEDENFILK	10891	4.79	1	1830.8942	2		0		yes	7.20	35.11	0.00

Sequence	Peptide Ion	Score	Hits	Mass	Charge	Tags	Conflicts	Modifications	In quantitation	Drift time (ms)	Average Normalised Abundances	
											CTR	MCD
VSFELFADK	7459	5.33	1	1054.5366	2		0		no	4.29	75.88	62.44
VSFELFADKVPK	1222	5.44	1	1378.7493	3		0		yes	3.88	1109.56	276.95
VSFELFADKVPK	2932	5.44	1	1378.7479	2		0		yes	5.75	275.96	22.09

	Tags
	Anova p-value ≤ 0.05
	power>0.8
	q<0.05

P05556

Integrin beta-1 OS=Homo sapiens OX=9606 GN=ITGB1 PE=1 SV=2
6 peptides

Sequence	Peptide Ion	Score	Hits	Mass	Charge	Tags	Conflicts	Modifications	In quantitation	Drift time (ms)	Average Normalised Abundances	
											CTR	MCD
CDDLEALK	4432	5.35	1	962.4485	2		0	[1] Carbamidomethyl C	no	4.43	149.44	46.16
DNTNEIYSGK	7713	6.35	1	1139.5127	2		0		no	4.36	186.41	10.70
IGFGSFVEK	2420	6.92	2	982.5137	2		0		yes	4.02	436.90	217.90
LSEGVTISYKSYCK	908	5.18	1	1633.7814	2		0	[13] Carbamidomethyl C	yes	6.16	2735.71	2195.55
SGEPQTFTLK	1605	6.97	2	1106.5584	2		0		yes	4.29	1242.46	550.87
TVMPYISTTPAK	4740	6.03	1	1323.6643	2		0	[3] Oxidation M	no	5.19	412.11	166.24

	Tags
	Anova p-value ≤ 0.05
	power>0.8
	q<0.05

P30086

Phosphatidylethanolamine-binding protein 1 OS=Homo sapiens OX=9606 GN=PEBP1 PE=1 SV=3
6 peptides

Sequence	Peptide Ion	Score	Hits	Mass	Charge	Tags	Conflicts	Modifications	In quantitation	Drift time (ms)	Average Normalised Abundance[illegible]	
											CTR	M[illegible]
CDEPILSNR	417	5.56	1	1102.5174	2		0	[1] Carbamidomethyl C	yes	4.36	2611.69	67[illegible]
GNDISSGTVLSDYVGSGPPK	3730	7.31	3	1948.9310	2		0		no	7.20	226.64	[illegible]
LYEQLSGK	1108	7.92	4	936.4900	2		0		yes	3.95	1916.64	[illegible]

Sequence	Peptide Ion	Score	Hits	Mass	Charge	Tags	Conflicts	Modifications	In quantitation	Drift time (ms)	Average Normalised Abundance	
											CTR	M[illegible]
LYTLVLTDPDAPSR	3482	7.83	4	1559.8123	2		0		no	6.16	589.72	[illegible]
VLTPTQVK	990	7.79	4	884.5332	2		0		yes	3.81	1326.27	[illegible]
VLTPTQVK+	3117	0.00	2	866.5196	2		0	[C-term] Dehydrated	no	3.81	305.80	

Tags	
	Anova p-value ≤ 0.05
	power>0.8
	q<0.05

P10909

Clusterin OS=Homo sapiens OX=9606 GN=CLU PE=1 SV=1
7 peptides

Sequence	Peptide Ion	Score	Hits	Mass	Charge	Tags	Conflicts	Modifications	In quantitation	Drift time (ms)	Average Normalised Abundances	
											CTR	MCD
ASSIIDELFQDR	5861	5.92	2	1392.6887	2		0		no	5.26	168.57	30.14
EILSVDCSTNNPSQAK	4377	6.13	3	1761.8160	2		0	[7] Carbamidomethyl C	no	6.51	218.19	46.88
FMETVAEK	3578	5.65	3	969.4529	2		0	[2] Oxidation M	yes	4.22	679.66	94.00
FYARVCR	1862	5.48	1	970.4821	2		0	[6] Carbamidomethyl C	yes	4.09	701.62	267.17
LFDSDPITVTVPVEVSR	5034	3.97	1	1873.0029	2		0		no	7.27	115.87	24.02
NPKFMETVAEK	1783	3.93	1	1308.6284	2		0	[11] Hydroxyl DKNP	yes	4.92	789.64	1384.80
TLLSNLEEAKK	5014	5.26	1	1260.6752	2		0	[5] Hydroxyl DKNP	no	5.06	154.47	47.27

Tags	
	Anova p-value ≤ 0.05
	power>0.8
	q<0.05

P59665

Neutrophil defensin 1 OS=Homo sapiens OX=9606 GN=DEFA1 PE=1 SV=1
4 peptides

Sequence	Peptide Ion	Score	Hits	Mass	Charge	Tags	Conflicts	Modifications	In quantitation	Drift time (ms)	Average Normalised Abundances	
											CTR	MCD
IPACIAGER	459	7.86	6	985.5045	2		0	[4] Carbamidomethyl C	yes	4.09	3507.85	945.36

Sequence	Peptide Ion	Score	Hits	Mass	Charge	Tags	Conflicts	Modifications	In quantitation	Drift time (ms)	Average Normalised Abundances	
											CTR	MCD
IPACIAGERR	2817	6.45	2	1141.6037	3		0	[4] Carbamidomethyl C	yes	3.32	312.67	14.07
IPACIAGERR	6333	6.45	2	1141.5966	2		0	[4] Carbamidomethyl C	yes	4.78	104.17	0.75
LWAFCC	3259	6.62	2	855.3401	1		0	[5] Carbamidomethyl C|[6] Carbamidomethyl C	no	9.14	337.42	21.47
YGTCIYQGR	1045	8.54	5	1116.5072	2		0	[4] Carbamidomethyl C	yes	4.29	1729.78	462.13

Tags	
[red]	Anova p-value ≤ 0.05
[green]	power>0.8
[blue]	q<0.05

Q6S8J3

POTE ankyrin domain family member E OS=Homo sapiens OX=9606 GN=POTEE PE=2 SV=3
9 peptides

Sequence	Peptide Ion	Score	Hits	Mass	Charge	Tags	Conflicts	Modifications	In quantitation	Drift time (ms)	Average Norm Abundances	
											CTR	M
AGDDAPR	1046	0.00	2	700.3145	1		0		no	8.10	542.72	
ALLLYGADIESK	442	4.37	1	1291.6772	2		0		yes	5.12	8016.47	
EIAALAPSMMK	2646	5.85	1	1160.5957	2		0		no	4.71	592.35	
EKDVLHENSTLR	1925	6.15	1	1439.7235	2		0		no	5.47	1877.54	
GDDAPR	839	0.00	2	629.2773	1		0		no	7.34	602.37	
ISSENSNPEQELKLTSEEESQR	635	4.39	1	2533.1452	4		0		yes	4.85	2060.51	1.
ISSENSNPEQELKLTSEEESQR	847	4.44	1	2533.1635	4		0		no	4.85	1283.13	
LKGSENGQPEK	333	5.26	1	1201.5974	2		0	[9] Hydroxyl DKNP	yes	4.78	1.03e+004	
MVVEVDSMPAASSVK	2131	5.13	1	1548.7604	2		0		no	6.02	291.62	
TPENQQFPDNESEEYHR	2981	4.65	1	2134.8684	3		0	[2] Hydroxyl DKNP	no	4.57	765.00	
TPENQQFPDNESEEYHR	9422	---	---	2134.8678	4		0	[2] Hydroxyl DKNP	no	3.95	179.78	

Tags	
[red]	Anova p-value ≤ 0.05
[green]	power>0.8
[blue]	q<0.05

Q14847

LIM and SH3 domain protein 1 OS=Homo sapiens OX=9606 GN=LASP1 PE=1 SV=2
6 peptides

Sequence	Peptide Ion	Score	Hits	Mass	Charge	Tags	Conflicts	Modifications	In quantitation	Drift time (ms)	Average Normalised Abundances	
											CTR	MCD
GFSVVADTPELQR	3671	6.67	2	1417.7185	2		0		yes	5.54	269.33	133.05
QQSELQSQVR	7760	6.91	1	1201.5985	2		0		no	4.78	84.59	10.32
QSFTMVADTPENLR	8908	5.72	1	1623.7591	2		0	[5] Oxidation M	no	6.02	147.32	31.95
TGDTGMLPANYVEAI	5704	4.62	1	1566.7226	2		0	[8] Hydroxyl DKNP	no	5.89	220.67	126.06
TQDQISNIK	2298	5.81	1	1045.5394	2		0		yes	4.36	522.00	96.78
TQDQISNIKYHEEFEK	1142	5.81	1	2021.9629	3		0	[9] Methyl K	yes	4.36	1773.92	1604.56

Tags	
	Anova p-value ≤ 0.05
	power>0.8
	q<0.05

B9A064

Immunoglobulin lambda-like polypeptide 5 OS=Homo sapiens OX=9606 GN=IGLL5 PE=2 SV=2
5 peptides

Sequence	Peptide Ion	Score	Hits	Mass	Charge	Tags	Conflicts	Modifications	In quantitation	Drift time (ms)	Average Normal Abunda
											CTR
SSLRSLWGR	2031	4.44	1	1060.5743	2		0		yes	4.50	474.20
SYSCQVTHEGSTVEK	2331	7.59	3	1710.7552	3		0	[4] Carbamidomethyl C	yes	4.57	1124.51
SYSCQVTHEGSTVEKTVAPTECS	2475	4.63	1	2556.1036	4		0	[4] Carbamidomethyl C|[22] Carbamidomethyl C	yes	4.99	1822.50
SYSCQVTHEGSTVEKTVAPTECS	4096	4.63	1	2556.1206	5		0	[4] Carbamidomethyl C|[22] Carbamidomethyl C	yes	4.29	985.22
TVAPTECS	1409	7.30	3	863.3686	1		0	[7] Carbamidomethyl C	no	9.00	689.22
TVAPTECS	6912	---	---	863.3740	2		0	[7] Carbamidomethyl C	no	3.53	72.76
YAASSYLSLTPEQWK	6852	6.78	2	1742.8404	2		0		no	6.58	123.71

Tags	
	Anova p-value ≤ 0.05
	power>0.8

	q<0.05

P02649

Apolipoprotein E OS=Homo sapiens OX=9606 GN=APOE PE=1 SV=1
6 peptides

Sequence	Peptide Ion	Score	Hits	Mass	Charge	Tags	Conflicts	Modifications	In quantitation	Drift time (ms)	Average Normalised Abundances	
											CTR	MCD
AATVGSLAGQPLQER	3240	6.24	3	1496.7885	2		0		no	5.75	262.57	230
AYKSELEEQLTPVAEETR	9344	5.35	1	2092.0210	3		0		no	4.71	136.94	4
LAVYQAGAR	2959	6.62	2	947.5163	2		0		no	3.88	512.98	99
LGADMEDVCGR	3726	5.82	1	1237.5093	2		0	[4] Hydroxyl DKNP | [9] Carbamidomethyl C	yes	4.71	669.26	43
LGPLVEQGR	2162	6.55	3	967.5411	2		0		yes	3.88	428.45	273
SWFEPLVEDMQR	2833	4.77	1	1551.6899	2		0	[5] Hydroxyl DKNP	yes	5.75	1880.21	1143

Tags	
	Anova p-value ≤ 0.05
	power>0.8
	q<0.05

P12109

Collagen alpha-1(VI) chain OS=Homo sapiens OX=9606 GN=COL6A1 PE=1 SV=3
6 peptides

Sequence	Peptide Ion	Score	Hits	Mass	Charge	Tags	Conflicts	Modifications	In quantitation	Drift time (ms)	Average Normalis Abundar
											CTR
EPCGGLEDAVNEAK	11709	4.65	1	1487.6452	2		0	[3] Carbamidomethyl C	no	5.82	22.82
GIDGVDGVKGEMGYPGLPGCK	2764	5.32	1	2118.9977	2		0	[20] Carbamidomethyl C | [21] Methyl K	yes	7.55	399.31
IALVITDGR	1521	7.29	2	956.5561	2		0		yes	4.02	611.59
KGLEQLLVGGSHLK	6760	5.63	1	1491.8729	2		0	[14] Methyl K	no	5.75	121.44
KGLEQLLVGGSHLK	10264	---	---	1491.8802	3		0	[14] Methyl K	no	3.95	33.58
VAVVQYSGTGQQRPER	3608	6.81	2	1773.9082	3		0		yes	4.22	353.62
VFSVAITPDHLEPR	6092	5.45	1	1579.8341	3		0		no	4.29	55.48

Tags	
	Anova p-value ≤ 0.05
	power>0.8
	q<0.05

P60174

Triosephosphate isomerase OS=Homo sapiens OX=9606 GN=TPI1 PE=1 SV=4
5 peptides

Sequence	Peptide Ion	Score	Hits	Mass	Charge	Tags	Conflicts	Modifications	In quantitation	Drift time (ms)	Average Normalised Abundances	
											CTR	MCD
IIYGGSVTGATCK	4051	6.91	4	1325.6558	2		0	[12] Carbamidomethyl C	yes	4.99	302.63	150.56
SNVSDAVAQSTR	1819	7.70	4	1233.5955	2		0		yes	4.78	1471.60	509.00
TATPQQAQEVHEK	3792	6.43	2	1465.7125	3		0		no	3.81	209.08	14.12
VVFEQTK	2610	6.94	4	849.4578	2		0		yes	3.67	723.25	129.91
VVLAYEPVWAIGTGK	5362	6.55	4	1601.8741	2		0		no	6.30	221.88	19.86

Tags	
	Anova p-value ≤ 0.05
	power>0.8
	q<0.05

P0C0L4

Complement C4-A OS=Homo sapiens OX=9606 GN=C4A PE=1 SV=2
6 peptides

Sequence	Peptide Ion	Score	Hits	Mass	Charge	Tags	Conflicts	Modifications	In quantitation	Drift time (ms)	Average Normali Abunda
											CTR
EMSGSPASGIPVK	2425	5.04	1	1274.6270	2		0	[2] Oxidation M	yes	4.99	619.84
GSFEFPVGDAVSK	3529	6.04	2	1338.6449	2		0		yes	5.19	340.47
QGSFQGGFR	4339	6.32	2	982.4671	2		0		yes	3.81	229.41
VGDTLNLNLR	4352	6.05	2	1113.6112	2		0		no	4.57	226.21
VTASDPLDTLGSEGALSPGGVASLLR	5244	5.62	1	2482.2737	3		0		no	6.65	95.64
YVLPNFEVK	7571	5.36	1	1107.5912	2		0		no	4.50	99.62

Tags	
	Anova p-value ≤ 0.05
	power>0.8
	q<0.05

P37802

Transgelin-2 OS=Homo sapiens OX=9606 GN=TAGLN2 PE=1 SV=3
6 peptides

Sequence	Peptide Ion	Score	Hits	Mass	Charge	Tags	Conflicts	Modifications	In quantitation	Drift time (ms)	Average Normalised Abundances	
											CTR	MCD
GASQAGMTGYGMPR	6921	5.04	1	1414.5973	2		0	[12] Oxidation M|[13] Hydroxyl DKNP	no	5.26	110.71	22.48
GPAYGLSR	8589	6.16	1	819.4207	2		0		no	3.67	92.96	49.01
NFSDNQLQEGK	2306	6.77	2	1278.5836	2		0		yes	4.92	464.01	422.36
NVIGLQMGTNR	1531	6.61	2	1217.6120	2		0	[7] Oxidation M	yes	4.78	1598.93	925.83
QMEQISQFLQAAER	926	4.59	1	1693.8029	3		0	[2] Oxidation M	yes	4.22	1947.55	1739.16
QMEQISQFLQAAER	7112	---	---	1693.7958	2		0	[2] Oxidation M	yes	6.30	54.05	2.32
TLMNLGGLAVAR	3681	5.19	1	1230.6650	2		0	[4] Hydroxyl DKNP	no	4.99	480.99	207.69

Tags	
	Anova p-value ≤ 0.05
	power>0.8
	q<0.05

P19440

Glutathione hydrolase 1 proenzyme OS=Homo sapiens OX=9606 GN=GGT1 PE=1 SV=2
5 peptides

Sequence	Peptide Ion	Score	Hits	Mass	Charge	Tags	Conflicts	Modifications	In quantitation	Drift time (ms)	Average Normalised Abundance	
											CTR	[illegible]
FVDVTEVVR	3157	5.32	2	1062.5666	2		0		no	4.36	446.93	[illegible]
GGLSVAVPGEIR	5007	5.87	2	1153.6411	2		0		no	4.64	228.73	[illegible]
GLAAALENK	919	4.23	1	899.5066	1		0	[9] Methyl K	yes	9.83	1432.94	[illegible]
GLAAALENK	2507	4.23	1	899.5124	2		0	[9] Methyl K	yes	3.74	557.77	[illegible]
LFQPSIQLAR	6699	6.07	2	1171.6677	2		0		no	4.71	162.53	[illegible]
QPLSSMCPTIMVGQDGQVR	1170	4.12	1	2118.9981	3		0	[7] Carbamidomethyl C|[15] Hydroxyl DKNP	yes	5.40	2208.31	[illegible]
QPLSSMCPTIMVGQDGQVR	2082	4.12	1	2118.9999	4		0	[7] Carbamidomethyl C|[15] Hydroxyl DKNP	yes	4.43	1060.51	[illegible]

Tags	
	Anova p-value ≤ 0.05
	power>0.8
	q<0.05

P63104

14-3-3 protein zeta/delta OS=Homo sapiens OX=9606 GN=YWHAZ PE=1 SV=1
4 peptides

Sequence	Peptide Ion	Score	Hits	Mass	Charge	Tags	Conflicts	Modifications	In quantitation	Drift time (ms)	Average Normalised Abundances	
											CTR	MCD
FLIPNASQAESK	1994	6.50	3	1303.6716	2		0		yes	5.26	1013.52	263.86
FLIPNASQAESK	6228	6.03	1	1319.6685	2		0	[5] Hydroxyl DKNP	no	5.19	272.64	141.59
SVTEQGAELSNEER	3202	7.87	4	1547.7047	2		0		yes	5.61	685.26	180.58
YLAEVAAGDDKK	3966	6.54	2	1278.6397	3		0		yes	3.74	360.74	162.46
YLAEVAAGDDKK	7731	6.54	1	1278.6398	2		0		no	5.47	119.29	8.23

Tags	
	Anova p-value ≤ 0.05
	power>0.8
	q<0.05

Q16555

Dihydropyrimidinase-related protein 2 OS=Homo sapiens OX=9606 GN=DPYSL2 PE=1 SV=1
6 peptides

Sequence	Peptide Ion	Score	Hits	Mass	Charge	Tags	Conflicts	Modifications	In quantitation	Drift time (ms)	Average Normalised Abundances	
											CTR	MCD
GSPLVVISQGK	4833	5.84	2	1083.6172	2		0		yes	4.57	227.32	45.06
MDENQFVAVTSTNAAK	13614	5.40	1	1740.7974	2		0	[2] Hydroxyl DKNP	no	6.51	46.19	0.00
MVIPGGIDVHTR	7193	4.75	1	1309.6767	3		0	[4] Hydroxyl DKNP	no	3.81	63.91	19.13
QIGENLIVPGGVK	2850	5.92	2	1322.7447	2		0		yes	5.26	232.58	100.79
SSAEVIAQAR	3061	5.70	2	1030.5421	2		0		yes	4.09	548.70	195.81
VFNLYPR	5096	5.44	1	907.4902	2		0		no	3.88	101.11	9.46

Tags	
	Anova p-value ≤ 0.05
	power>0.8
	q<0.05

P06576

ATP synthase subunit beta_ mitochondrial OS=Homo sapiens OX=9606 GN=ATP5F1B PE=1 SV=3
6 peptides

Sequence	Peptide Ion	Score	Hits	Mass	Charge	Tags	Conflicts	Modifications	In quantitation	Drift time (ms)	Average Normalised Abundances	
											CTR	MCD
AHGGYSVFAGVGER	891	4.88	1	1405.6830	2		0		yes	6.23	724.89	2781.43
AIAELGIYPAVDPLDSTSR	2993	6.48	4	1987.0191	2		0		no	7.41	507.67	394.93
AIAELGIYPAVDPLDSTSR	4418	---	---	1987.0132	3		0		no	4.64	180.49	100.14
IPVGPETLGR	1464	4.33	1	1069.5632	2		0	[2] Hydroxyl DKNP|[5] Hydroxyl DKNP	yes	4.29	1222.29	748.12
TIAMDGTEGLVR	1317	5.63	1	1277.6279	2		0	[4] Oxidation M	yes	4.92	1375.63	700.68
VLDSGAPIKIPVGPETLGR	2677	6.14	4	1918.0761	3		0		no	4.50	1765.60	316.49
VVDLLAPYAK	2735	5.46	4	1087.6197	2		0		no	4.64	405.80	239.76

Tags	
	Anova p-value ≤ 0.05
	power>0.8
	q<0.05

P81605

Dermcidin OS=Homo sapiens OX=9606 GN=DCD PE=1 SV=2
3 peptides

Sequence	Peptide Ion	Score	Hits	Mass	Charge	Tags	Conflicts	Modifications	In quantitation	Drift time (ms)	Average Normalised Abundances	
											CTR	MCD
DAVEDLESVGK	2779	5.55	1	1160.5618	2		0		yes	4.64	417.36	780.72
GAVHDVK	213	6.14	1	754.3984	1		0	[7] Hydroxyl DKNP|[7] Methyl K	yes	8.38	1.08e+004	1970.90
GAVHDVK	718	6.14	1	754.3982	2		0	[7] Hydroxyl DKNP|[7] Methyl K	yes	3.39	3077.53	609.41
LGKDAVEDLESVGK	634	7.23	8	1458.7565	3		0		yes	3.74	2518.69	4282.77
LGKDAVEDLESVGK	2893	7.23	4	1458.7480	2		0		yes	5.61	451.91	703.32

Tags	
	Anova p-value ≤ 0.05
	power>0.8
	q<0.05

O14950

Myosin regulatory light chain 12B OS=Homo sapiens OX=9606 GN=MYL12B PE=1 SV=2
5 peptides

Sequence	Peptide Ion	Score	Hits	Mass	Charge	Tags	Conflicts	Modifications	In quantitation	Drift time (ms)	Average Normalised Abundances	
											CTR	MCD
ATSNVFAMFDQSQIQEFK	7922	5.48	1	2105.9728	3		0	[4] Hydroxyl DKNP	no	4.92	56.81	2.65
ATSNVFAMFDQSQIQEFK	14085	---	---	2105.9556	2		0	[4] Hydroxyl DKNP	no	7.62	23.33	1.05
ELLTTMGDR	4081	6.07	1	1050.5050	2		0	[8] Hydroxyl DKNP	yes	4.29	391.78	157.76
ELLTTMGDRFTDEEVDELYR	5987	6.13	1	2447.1226	3		0	[16] Hydroxyl DKNP	no	5.19	189.24	36.55
GNFNYIEFTR	2230	7.95	3	1259.5950	2		0		yes	4.99	728.51	480.03
LNGTDPEDVIR	4420	6.65	2	1227.6093	2		0		yes	4.78	575.35	237.60

Tags	
	Anova p-value ≤ 0.05
	power>0.8
	q<0.05

P14625

Endoplasmin OS=Homo sapiens OX=9606 GN=HSP90B1 PE=1 SV=1
5 peptides

Sequence	Peptide Ion	Score	Hits	Mass	Charge	Tags	Conflicts	Modifications	In quantitation	Drift time (ms)	Average Normalised Abundances	
											CTR	MCD
GLFDEYGSK	2614	4.29	1	1014.4835	2		0		yes	4.02	730.46	435.17
GLFDEYGSK	4306	4.29	1	1014.4720	3		0		no	3.05	94.33	16.00
GVVDSDDLPLNVSR	4529	6.47	2	1484.7415	2		0		no	5.61	257.20	121.30
SGTSEFLNK	4091	5.77	1	981.4777	2		0		no	3.95	286.09	133.17
SGYLLPDTKAYGDR	2387	4.36	1	1570.7592	2		0	[7] Hydroxyl DKNP	yes	5.89	564.79	895.92
SILFVPTSAPR	2640	6.49	2	1186.6655	2		0		yes	4.85	353.39	153.25

Tags	
	Anova p-value ≤ 0.05
	power>0.8
	q<0.05

O75083

WD repeat-containing protein 1 OS=Homo sapiens OX=9606 GN=WDR1 PE=1 SV=4
6 peptides

Sequence	Peptide Ion	Score	Hits	Mass	Charge	Tags	Conflicts	Modifications	In quantitation	Drift time (ms)	Average Normalis Abundan	
											CTR	[illegible]
AHDGGIYAISWSPDSTHLLSASGDK	12126	4.00	1	2584.1889	3		0		no	6.44	36.13	[illegible]

Sequence	Peptide Ion	Score	Hits	Mass	Charge	Tags	Conflicts	Modifications	In quantitation	Drift time (ms)	Average Normalis Abundan	
											CTR	M
DYSGQGVVK	6992	5.35	1	951.4703	2		0		yes	3.95	218.98	5
IAVVGEGR	5653	5.80	1	799.4520	2		0		no	3.53	151.62	2
LYSILGTTLKDEGK	4526	5.06	1	1536.8331	3		0		yes	4.22	290.49	6
VFASLPQVER	4726	5.60	1	1144.6241	2		0		yes	4.57	249.34	4
YAPSGFYIASGDVSGK	9580	5.57	1	1617.7648	2		0		no	6.02	92.60	1

Tags	
	Anova p-value ≤ 0.05
	power>0.8
	q<0.05

P68133

Actin_ alpha skeletal muscle OS=Homo sapiens OX=9606 GN=ACTA1 PE=1 SV=1
4 peptides

Sequence	Peptide Ion	Score	Hits	Mass	Charge	Tags	Conflicts	Modifications	In quantitation	Dr ti (m
GYSFVTTAER	1655	7.77	2	1129.5450	2		0		no	4.
GYSFVTTAER	6128	6.74	1	1129.5532	2		0		no	4.
TTGIVLDSGDGVTHNVPIYEGYALPHAIMR	562	5.42	2	3195.6273	5		0		yes	4.
TTGIVLDSGDGVTHNVPIYEGYALPHAIMR	698	5.42	2	3195.6238	4		0		yes	5.
VAPEEHPTLLTEAPLNPK	799	7.33	3	1955.0359	3		0		yes	4.
YSVWIGGSILASLSTFQQMWITKQEYDEAGPSIVHR	1697	5.19	1	4097.0650	5		0		yes	6.
YSVWIGGSILASLSTFQQMWITKQEYDEAGPSIVHR	5757	---	---	4097.0634	4		0		yes	7.

Tags	
	Anova p-value ≤ 0.05
	power>0.8
	q<0.05

P01871

Immunoglobulin heavy constant mu OS=Homo sapiens OX=9606 GN=IGHM PE=1 SV=4
5 peptides

Sequence	Peptide Ion	Score	Hits	Mass	Charge	Tags	Conflicts	Modifications	In quantitation	Drift time (ms)	Average Normalised Abundances	
											CTR	MCD
DGFFGNPR	6560	5.86	1	908.4306	2		0		no	3.81	102.03	2.28
LICQATGFSPR	5009	6.10	2	1248.6212	2		0	[3] Carbamidomethyl C	yes	4.99	309.43	91.46

Sequence	Peptide Ion	Score	Hits	Mass	Charge	Tags	Conflicts	Modifications	In quantitation	Drift time (ms)	Average Normalised Abundances	
											CTR	MCD
QIQVSWLR	5616	6.39	1	1028.5721	2		0		no	4.29	86.68	9.26
QVGSGVTTDQVQAEAK	3833	6.90	2	1616.7992	2		0		yes	6.09	282.17	27.95
YVTSAPMPEPQAPGR	4383	5.46	2	1615.7621	2		0	[8] Hydroxyl DKNP	yes	5.96	402.98	3.73

Tags	
	Anova p-value ≤ 0.05
	power>0.8
	q<0.05

P10412

Histone H1.4 OS=Homo sapiens OX=9606 GN=H1-4 PE=1 SV=2
4 peptides

Sequence	Peptide Ion	Score	Hits	Mass	Charge	Tags	Conflicts	Modifications	In quantitation	Drift time (ms)	Average Normalised Abundances	
											CTR	MCD
AKKPAAAAGAK	10	5.38	1	1012.6054	2		0	[2] Hydroxyl DKNP|[11] Methyl K	yes	4.29	1.00e+005	2.79e+005
ALAAAGYDVEK	4120	5.52	1	1106.5495	2		0		no	4.78	198.58	108.35
ASGPPVSELITK	844	6.79	3	1197.6582	2		0		yes	4.57	1657.39	1727.63
KASGPPVSELITK	1684	6.50	2	1325.7523	3		0		yes	3.46	433.23	300.24
KASGPPVSELITK	3201	6.50	2	1325.7471	2		0		yes	5.12	220.80	196.18

Tags	
	Anova p-value ≤ 0.05
	power>0.8
	q<0.05

P08729

Keratin_ type II cytoskeletal 7 OS=Homo sapiens OX=9606 GN=KRT7 PE=1 SV=5
6 peptides

Sequence	Peptide Ion	Score	Hits	Mass	Charge	Tags	Conflicts	Modifications	In quantitation	Drift time (ms)	Average Normalised Abundance[illegible]	
											CTR	M[illegible]
AEAEAWYQTK	4686	5.55	1	1211.5564	2		0	[10] Hydroxyl DKNP	no	4.92	143.95	
AKLEAAIAEAEER	1345	4.84	1	1399.7470	3		0		yes	4.02	852.69	21[illegible]
AKLEAAIAEAEER	2577	---	---	1399.7481	2		0		yes	5.96	349.93	11[illegible]
LSSARPGGLGSSSLYGLGASRPR	3179	4.95	1	2245.1806	3		0		no	5.06	651.53	10[illegible]
SLDLDGIIAEVK	3211	4.55	1	1285.7215	3		0	[12] Methyl K	no	3.53	355.03	
SLDLDGIIAEVK	813	5.65	1	1301.7080	2		0		yes	5.26	3412.10	24[illegible]

Sequence	Peptide Ion	Score	Hits	Mass	Charge	Tags	Conflicts	Modifications	In quantitation	Drift time (ms)	Average Normalised Abundances	
											CTR	MC
								[12] Hydroxyl DKNP|[12] Methyl K				
YEDEINHR	1763	4.81	1	1090.4827	2		0	[6] Hydroxyl DKNP	yes	4.22	2091.32	5

Tags	
	Anova p-value ≤ 0.05
	power>0.8
	q<0.05

Q6UXI9

Nephronectin OS=Homo sapiens OX=9606 GN=NPNT PE=2 SV=3
5 peptides

Sequence	Peptide Ion	Score	Hits	Mass	Charge	Tags	Conflicts	Modifications	In quantitation	Drift time (ms)	Average Normalised Abundances	
											CTR	MCD
CKHGECIGPNK	2761	4.39	1	1312.6082	2		0	[1] Carbamidomethyl C|[6] Carbamidomethyl C|[11] Methyl K	yes	5.06	724.88	525.97
CQCPSPGLQLAPDGR	8902	6.10	1	1654.7483	2		0	[1] Carbamidomethyl C|[3] Carbamidomethyl C	no	5.96	51.56	4.10
IDCCWGWAR	8587	6.14	1	1222.5138	2		0	[3] Carbamidomethyl C|[4] Carbamidomethyl C	no	4.71	98.02	13.98
QIVSSIGLCR	3177	6.65	2	1131.6022	2		0	[9] Carbamidomethyl C	yes	4.57	467.73	161.53
TCVDVDECATGR	4742	6.89	2	1381.5654	2		0	[2] Carbamidomethyl C|[8] Carbamidomethyl C	yes	5.26	273.36	25.80

Tags	
	Anova p-value ≤ 0.05
	power>0.8
	q<0.05

P09210

Glutathione S-transferase A2 OS=Homo sapiens OX=9606 GN=GSTA2 PE=1 SV=4
5 peptides

Sequence	Peptide Ion	Score	Hits	Mass	Charge	Tags	Conflicts	Modifications	In quantitation	Drift time (ms)	Average Normalised Abundances	
											CTR	MCD
FLQPGSPR	2245	6.98	2	900.4762	2		0		yes	3.88	828.01	125.40
ISNLPTVK	3803	6.30	1	870.5169	2		0		no	3.74	218.17	26.33
LALIQEK	2151	7.19	2	813.4925	2		0		yes	3.67	546.61	88.33
SHGQDYLVGNK	735	4.74	1	1230.6015	2		0	[11] Methyl K	yes	4.57	3932.67	2668.95
YFPAFEK	4949	4.95	1	900.4556	2		0		no	3.95	358.26	95.80

Tags	
	Anova p-value ≤ 0.05
	power>0.8
	q<0.05

P39060

Collagen alpha-1(XVIII) chain OS=Homo sapiens OX=9606 GN=COL18A1 PE=1 SV=5
5 peptides

Sequence	Peptide Ion	Score	Hits	Mass	Charge	Tags	Conflicts	Modifications	In quantitation	Drift time (ms)	Average Normalised Abundances	
											CTR	MCD
ELLREETGAALKPR	3165	5.85	2	1581.8602	3		0		no	4.36	210.12	149.8[illegible]
GADFQCFQQAR	2191	7.30	2	1326.5835	2		0	[6] Carbamidomethyl C	yes	4.99	804.45	299.5[illegible]
GPPGPQGPPGPPGPSFR	10071	4.08	1	1597.8218	2		0		no	6.44	40.99	9.0[illegible]
LTESYCETWR	3581	6.17	2	1343.6068	2		0	[6] Carbamidomethyl C	yes	5.12	644.66	221.1[illegible]
TEAPSATGQASSLLGGR	1944	6.37	2	1601.7952	2		0		yes	6.09	524.67	516.2[illegible]

Tags	
	Anova p-value ≤ 0.05
	power>0.8
	q<0.05

P06733

Alpha-enolase OS=Homo sapiens OX=9606 GN=ENO1 PE=1 SV=2
5 peptides

Sequence	Peptide Ion	Score	Hits	Mass	Charge	Tags	Conflicts	Modifications	In quantitation	Drift time (ms)	Average Normalised Abundances	
											CTR	MCD
GNPTVEVDLFTSK	2417	6.66	4	1405.7075	2		0		no	5.33	773.68	361.55
IDKLMIEMDGTENK	4527	4.83	1	1651.7826	3		0	[2] Hydroxyl DKNP	no	4.16	146.01	36.69
IEEELGSK	1110	6.80	4	903.4557	2		0		yes	3.81	1796.66	767.57

Sequence	Peptide Ion	Score	Hits	Mass	Charge	Tags	Conflicts	Modifications	In quantitation	Drift time (ms)	Average Normalised Abundances	
											CTR	MCD
SGKYDLDFK	2608	4.84	1	1087.5239	2		0	[7] Hydroxyl DKNP	yes	4.29	1294.23	410.62
YISPDQLADLYK	1291	6.42	4	1424.7169	2		0		yes	5.68	1267.36	634.72

Tags	
	Anova p-value ≤ 0.05
	power>0.8
	q<0.05

Q86Y46

Keratin_ type II cytoskeletal 73 OS=Homo sapiens OX=9606 GN=KRT73 PE=1 SV=1
8 peptides

Sequence	Peptide Ion	Score	Hits	Mass	Charge	Tags	Conflicts	Modifications	In quantitation	Drift time (ms)	Average Norm[illegible] Abundances	
											CTR	MC[illegible]
AQYEEIARK	1203	4.65	1	1106.5669	2		0		yes	4.64	1062.52	[illegible]
EGEECR	1585	0.00	2	778.2922	1		0	[5] Carbamidomethyl C	no	8.10	1999.06	[illegible]
GLSGGFSSR	4069	4.59	1	866.4131	1		0		no	9.63	175.62	[illegible]
HGDDLKHTK	132	5.89	1	1049.5333	2		0		yes	4.22	1.22e+004	3.4[illegible]
LDELEGALQQAKEELAR	5882	4.65	1	1911.9870	2		0		no	7.41	72.04	[illegible]
LDELEGALQQAKEELAR	10819	---	---	1911.9609	3		0		no	4.85	75.88	[illegible]
LEGEECR	4055	0.00	2	891.3742	1		0	[6] Carbamidomethyl C	no	9.42	516.40	[illegible]
LGSASEFR	316	4.67	2	865.4183	1		0		yes	9.70	1.02e+004	[illegible]
VLNNKFASFIDK	2421	4.88	1	1410.7400	2		0	[3] Hydroxyl DKNP	no	5.61	406.52	[illegible]

Tags	
	Anova p-value ≤ 0.05
	power>0.8
	q<0.05

P01009

Alpha-1-antitrypsin OS=Homo sapiens OX=9606 GN=SERPINA1 PE=1 SV=3
5 peptides

Sequence	Peptide Ion	Score	Hits	Mass	Charge	Tags	Conflicts	Modifications	In quantitation	Drift time (ms)	Average Normalised Abundances	
											CTR	MCD
AVLTIDEK	3082	5.67	2	887.4941	2		0		no	3.81	257.46	100.36
FLENEDRR	3599	4.71	2	1077.5215	3		0		no	3.12	241.23	57.90

Sequence	Peptide Ion	Score	Hits	Mass	Charge	Tags	Conflicts	Modifications	In quantitation	Drift time (ms)	Average Normalised Abundances	
											CTR	MCD
FLENEDRR	4226	4.71	2	1077.5212	2		0		no	4.50	251.27	79.91
QINDYVEK	4047	4.53	1	1007.5030	2		0		yes	4.36	432.10	153.83
SPLFMGK	3228	3.97	1	794.4006	2		0	[7] Hydroxyl DKNP	yes	3.53	384.73	96.73
SVLGQLGITK	4772	5.59	1	1014.5979	2		0		yes	4.22	258.33	138.15

Tags	
	Anova p-value ≤ 0.05
	power>0.8
	q<0.05

P00915

Carbonic anhydrase 1 OS=Homo sapiens OX=9606 GN=CA1 PE=1 SV=2
5 peptides

Sequence	Peptide Ion	Score	Hits	Mass	Charge	Tags	Conflicts	Modifications	In quantitation	Drift time (ms)	Average Normalised Abundances	
											CTR	MCD
GGPFSDSYR	2069	6.30	2	984.4351	2		0		yes	4.02	589.03	195.07
SVLKGGPFSDSYR	188	4.98	1	1411.7127	2		0		yes	5.89	5300.44	1.82e+004
VGEANPKLQK	5957	5.07	1	1098.6030	2		0	[10] Hydroxyl DKNP	no	4.64	106.13	17.49
YSAELHVAHWNSAK	2740	5.61	1	1611.7895	3		0		yes	4.29	641.59	217.70
YSSLAEAASK	3760	7.11	2	1025.5049	2		0		no	4.16	358.52	74.76

Tags	
	Anova p-value ≤ 0.05
	power>0.8
	q<0.05

P22626

Heterogeneous nuclear ribonucleoproteins A2/B1 OS=Homo sapiens OX=9606 GN=HNRNPA2B1 PE=1 SV=2
4 peptides

Sequence	Peptide Ion	Score	Hits	Mass	Charge	Tags	Conflicts	Modifications	In quantitation	Drift time (ms)	Average Normalised Abundances	
											CTR	MCD
GGGGNFGPGPGSNFR	1853	7.24	3	1376.6266	2		0		yes	5.12	607.02	499.81
GGNFGFGDSR	1306	7.23	3	1012.4422	2		0		yes	4.09	749.41	788.10
IDTIEIITDR	5465	7.00	1	1187.6356	2		0		no	4.78	149.54	80.76
TLETVPLER	2534	7.14	2	1056.5712	2		0		yes	4.29	770.84	470.33

Tags	
	Anova p-value ≤ 0.05
	power>0.8
	q<0.05

P01857

Immunoglobulin heavy constant gamma 1 OS=Homo sapiens OX=9606 GN=IGHG1 PE=1 SV=1
4 peptides

Sequence	Peptide Ion	Score	Hits	Mass	Charge	Tags	Conflicts	Modifications	In quantitation	Drift time (ms)	Average Normalised Abundances	
											CTR	[illegible]
FNWYVDGVEVHNAK	5154	6.55	4	1676.7968	3		0		no	4.29	240.23	[illegible]
GPSVFPLAPSSK	868	8.20	4	1185.6380	2		0		yes	4.57	1371.46	[illegible]
TPEVTCVVVDVSHEDPEVK	3814	7.39	3	2138.0169	3		0	[6] Carbamidomethyl C	yes	5.40	443.21	[illegible]
TTPPVLDSDGSFFLYSK	3515	6.46	1	1872.9172	2		0		yes	6.93	363.95	[illegible]
TTPPVLDSDGSFFLYSK	4658	6.35	1	1872.8979	2		0		no	6.93	108.83	[illegible]

Tags	
	Anova p-value ≤ 0.05
	power>0.8
	q<0.05

P01834

Immunoglobulin kappa constant OS=Homo sapiens OX=9606 GN=IGKC PE=1 SV=2
3 peptides

Sequence	Peptide Ion	Score	Hits	Mass	Charge	Tags	Conflicts	Modifications	In quantitation	Drift time (ms)	Average Normalised Abundances	
											CTR	[illegible]
TVAAPSVFIFPPSDEQLK	2694	7.57	4	1945.0106	2		0		yes	7.41	1624.32	
TVAAPSVFIFPPSDEQLK	3121	7.57	2	1945.0114	3		0		yes	5.12	951.74	
VDNALQSGNSQESVTEQDSK	6830	6.12	1	2134.9529	3		0		yes	4.71	182.89	
VYACEVTHQGLSSPVTK	1766	7.16	4	1874.9195	3		0	[4] Carbamidomethyl C	yes	4.36	958.04	[illegible]

Tags	
	Anova p-value ≤ 0.05
	power>0.8
	q<0.05

P30101

Protein disulfide-isomerase A3 OS=Homo sapiens OX=9606 GN=PDIA3 PE=1 SV=4
5 peptides

Sequence	Peptide Ion	Score	Hits	Mass	Charge	Tags	Conflicts	Modifications	In quantitation	Drift time (ms)	Average Normalised Abundances	
											CTR	MCD
ELSDFISYLQR	8609	6.20	1	1369.6867	2		0		no	5.47	86.26	22.47
FVMQEEFSR	3571	5.11	2	1187.5425	2		0	[3] Oxidation M	yes	4.85	305.26	124.35
IFRDGEEAGAYDGPR	4910	5.76	2	1651.7570	3		0		yes	4.29	242.51	132.23
LAPEYEAAATR	2382	6.21	2	1190.5889	2		0		yes	4.64	1102.92	637.23
MDATANDVPSPYEVR	10592	5.06	1	1679.7525	2		0	[2] Hydroxyl DKNP	no	6.09	72.79	19.33

Tags	
	Anova p-value ≤ 0.05
	power>0.8
	q<0.05

P59998

Actin-related protein 2/3 complex subunit 4 OS=Homo sapiens OX=9606 GN=ARPC4 PE=1 SV=3
4 peptides

Sequence	Peptide Ion	Score	Hits	Mass	Charge	Tags	Conflicts	Modifications	In quantitation	Drift time (ms)	Average Normalised Abundances	
											CTR	MCD
AENFFILR	3628	7.73	2	1008.5386	2		0		yes	4.29	206.28	50.39
ELLLQPVTISR	4400	6.92	2	1267.7432	2		0		yes	5.06	185.09	46.80
IVAEEFLK	1337	7.41	3	947.5299	2		0		yes	4.16	559.70	361.01
VLIEGSINSVR	5709	6.25	2	1185.6652	2		0		no	5.06	98.84	40.80

Tags	
	Anova p-value ≤ 0.05
	power>0.8
	q<0.05

P06702

Protein S100-A9 OS=Homo sapiens OX=9606 GN=S100A9 PE=1 SV=1
3 peptides

Sequence	Peptide Ion	Score	Hits	Mass	Charge	Tags	Conflicts	Modifications	In quantitation	Drift time (ms)	Average Normalised Abundances	
											CTR	MCD
LGHPDTLNQGEFK	2225	6.60	2	1454.7117	3		0		yes	3.67	984.30	250.02

Sequence	Peptide Ion	Score	Hits	Mass	Charge	Tags	Conflicts	Modifications	In quantitation	Drift time (ms)	Average Normalised Abundances	
											CTR	MCD
LGHPDTLNQGEFK	10480	---	---	1454.7043	2		0		yes	5.61	84.03	10.22
LTWASHEK	2346	6.92	2	970.4885	2		0		yes	4.22	1822.03	378.16
NIETIINTFHQYSVK	2526	7.27	2	1805.9307	3		0		yes	4.71	1950.39	20.93
NIETIINTFHQYSVK	7685	7.27	1	1805.9291	2		0		yes	7.41	324.38	0.02

Tags	
	Anova p-value ≤ 0.05
	power>0.8
	q<0.05

Q99798

Aconitate hydratase_ mitochondrial OS=Homo sapiens OX=9606 GN=ACO2 PE=1 SV=2
6 peptides

Sequence	Peptide Ion	Score	Hits	Mass	Charge	Tags	Conflicts	Modifications	In quantitation	Drift time (ms)	Average Normalised Abundances	
											CTR	MCD
GEFDPGQDTYQHPPK	5052	4.16	1	1714.7595	2		0		yes	6.37	203.03	114.51
NAVTQEFGPVPDTAR	6095	5.54	1	1600.7775	2		0		yes	5.96	224.51	176.08
NTIVTSYNRNFTGR	5911	4.12	1	1641.8130	2		0		no	6.30	95.76	8.92
SQFTITPGSEQIR	5650	4.64	1	1462.7401	2		0		no	5.68	239.38	38.57
TGREDIANLADEFK	2201	4.77	1	1577.7636	2		0		yes	5.96	356.46	185.00
WVVIGDENYGEGSSR	4225	4.54	1	1666.7627	2		0		no	6.16	330.29	1.50

Tags	
	Anova p-value ≤ 0.05
	power>0.8
	q<0.05

P35580

Myosin-10 OS=Homo sapiens OX=9606 GN=MYH10 PE=1 SV=3
6 peptides

Sequence	Peptide Ion	Score	Hits	Mass	Charge	Tags	Conflicts	Modifications	In quantitation	Drift time (ms)	Average Normalised Abundances	
											CTR	MCD
AKLQELEGAVK	2474	3.85	1	1184.6746	3		0		yes	3.67	710.57	439.04
ALEQQVEEMR	5891	5.07	1	1231.5965	2		0		no	5.06	235.38	25.48
ELDDATEANEGLSR	4192	5.10	1	1518.6571	2		0		no	6.02	132.12	52.93
ELDDATEANEGLSR	8269	5.35	1	1518.6766	2		0		no	5.61	80.08	28.21

Sequence	Peptide Ion	Score	Hits	Mass	Charge	Tags	Conflicts	Modifications	In quantitation	Drift time (ms)	Average Normalised Abundances	
											CTR	MCD
LQNELDNVSTLLEEAEK	2584	4.53	1	1943.9505	3		0		yes	4.85	1045.58	405.73
QEVMISDLEER	2845	4.52	1	1347.6142	2		0		no	5.06	223.90	19.45
QLRADMEDLMSSK	3312	4.39	1	1536.7275	3		0	[13] Methyl K	yes	3.88	787.50	148.68

Tags	
	Anova p-value ≤ 0.05
	power>0.8
	q<0.05

P05062

Fructose-bisphosphate aldolase B OS=Homo sapiens OX=9606 GN=ALDOB PE=1 SV=2
4 peptides

Sequence	Peptide Ion	Score	Hits	Mass	Charge	Tags	Conflicts	Modifications	In quantitation	Drift time (ms)	Averag Norma Abund
											CTR
ETTIQGLDGLSER	1175	7.84	2	1417.7042	2		0		yes	5.47	1839.3
GILAADESVGTMGNR	1581	7.37	1	1505.7126	2		0	[14] Hydroxyl DKNP	yes	5.61	1243.7
LDQGGAPLAGTNK	2742	6.80	2	1240.6384	2		0		yes	4.92	657.0
LDQGGAPLAGTNKETTIQGLDGLSER	6217	5.62	2	2640.3316	3		0		no	5.68	248.6

Tags	
	Anova p-value ≤ 0.05
	power>0.8
	q<0.05

O00592

Podocalyxin OS=Homo sapiens OX=9606 GN=PODXL PE=1 SV=2
4 peptides

Sequence	Peptide Ion	Score	Hits	Mass	Charge	Tags	Conflicts	Modifications	In quantitation	Drift time (ms)	Average Normalised Abundances	
											CTR	MCD
ATFNPAQDK	5175	6.50	1	990.4818	2		0		yes	4.09	236.29	9.01
CEDLETQTQSEK	3338	7.36	2	1466.6119	2		0	[1] Carbamidomethyl C	yes	5.54	496.58	18.32
LASVPGSQTVVVK	468	7.01	2	1283.7473	2		0		yes	5.12	2284.88	1053.11
LISLICR	4282	6.69	1	873.5079	2		0	[6] Carbamidomethyl C	no	3.81	111.59	8.87

Tags	

	Anova p-value ≤ 0.05
	power>0.8
	q<0.05

P01833

Polymeric immunoglobulin receptor OS=Homo sapiens OX=9606 GN=PIGR PE=1 SV=4
4 peptides

Sequence	Peptide Ion	Score	Hits	Mass	Charge	Tags	Conflicts	Modifications	In quantitation	Drift time (ms)	Average Normalised Abundances	
											CTR	MCD
CGLGINSR	4857	6.31	1	875.4297	2		0	[1] Carbamidomethyl C	yes	3.81	294.40	1.[illegible]
EEFVATTESTTETKEPK	804	4.12	1	1939.9237	2		0	[14] Methyl K	yes	7.13	5146.50	2128.[illegible]
EEFVATTESTTETKEPK	2560	4.12	1	1939.9250	3		0	[14] Methyl K	yes	4.36	1185.64	286.[illegible]
GGCITLISSEGYVSSK	7667	5.40	1	1656.8154	2		0	[3] Carbamidomethyl C	no	6.44	208.96	5.[illegible]
QSSGENCDVVVNTLGK	4496	5.98	1	1705.7925	2		0	[7] Carbamidomethyl C	yes	6.37	230.77	1.[illegible]

Tags	
	Anova p-value ≤ 0.05
	power>0.8
	q<0.05

P68104

Elongation factor 1-alpha 1 OS=Homo sapiens OX=9606 GN=EEF1A1 PE=1 SV=1
5 peptides

Sequence	Peptide Ion	Score	Hits	Mass	Charge	Tags	Conflicts	Modifications	In quantitation	Drift time (ms)	Average Normalised Abundances	
											CTR	MCD
IGGIGTVPVGR	857	7.58	4	1024.6027	2		0		yes	4.16	2142.11	937.03
QLIVGVNK	100	4.93	1	869.5337	2		0		yes	4.29	6511.42	1.59e+004
QTVAVGVIK	1032	6.93	4	913.5541	2		0		yes	4.16	838.83	645.85
QTVAVGVIK+	4690	0.00	1	895.5402	2		0	[C-term] Dehydrated	no	3.88	145.42	102.39
TIEKFEK	4794	6.44	1	893.4831	2		0		no	3.95	291.07	69.96

Tags	
	Anova p-value ≤ 0.05
	power>0.8
	q<0.05

Q04695

Keratin_ type I cytoskeletal 17 OS=Homo sapiens OX=9606 GN=KRT17 PE=1 SV=2
10 peptides

Sequence	Peptide Ion	Score	Hits	Mass	Charge	Tags	Conflicts	Modifications	In quantitation	Drift time (ms)	Average Normalised Abundances	
											CTR	MCD
AADDFR	65	0.00	12	693.3101	1		0		yes	7.76	3.20e+004	1.43e+0
AADDFR	1810	0.00	1	693.3086	2		0		yes	3.25	743.62	165.
ADDFR	61	0.00	12	622.2733	1		0		yes	7.13	3.13e+004	1.55e+0
ADDFR	4635	---	---	622.2714	2		0		yes	3.12	268.31	45.
ASLEGNLAETENR	8450	4.97	1	1418.6629	2		0	[12] Hydroxyl DKNP	no	5.47	257.51	12.
ASYLDK	2075	0.00	1	695.3494	1		0		no	8.03	554.32	430.
ASYLDKVR	1879	0.00	1	950.5044	2		0		no	4.16	1329.56	280.
ATMQNLNDRLASYLDK	3347	5.08	1	1851.9062	3		0		no	4.29	219.85	686.
ATMQNLNDRLASYLDK	1261	6.03	1	1867.9018	3		0	[5] Hydroxyl DKNP	yes	4.36	1476.48	4482.
DELTLAR	1427	0.00	1	816.4406	1		0		no	9.14	862.65	565.
LSVEADINGLR	1280	4.63	2	1185.6487	2		0		no	4.92	748.55	2186.
TIEELQNK	6142	5.07	1	989.4985	2		0	[8] Hydroxyl DKNP	no	4.16	196.50	41.

Tags	
	Anova p-value ≤ 0.05
	power>0.8
	q<0.05

P00918

Carbonic anhydrase 2 OS=Homo sapiens OX=9606 GN=CA2 PE=1 SV=2
5 peptides

Sequence	Peptide Ion	Score	Hits	Mass	Charge	Tags	Conflicts	Modifications	In quantitation	Drift time (ms)	Average Normalised Abundances	
											CTR	MCD
AVLKGGPLDGTYR	1061	4.46	1	1361.7283	2		0	[4] Hydroxyl DKNP	yes	5.54	637.95	1857.8
DFPIAK	352	4.53	1	689.3736	1		0		yes	7.41	1.89e+004	5.88e+00
GGPLDGTYR	3224	6.34	4	934.4531	2		0		no	3.88	610.14	203.3
KYAAELHLVHWNTK	4159	3.97	1	1722.9163	3		0	[14] Methyl K	no	4.71	384.55	61.7
SADFTNFDPR	2054	6.29	4	1168.5254	2		0		yes	4.71	1012.98	289.0

Tags	
	Anova p-value ≤ 0.05
	power>0.8
	q<0.05

P05787

Keratin_ type II cytoskeletal 8 OS=Homo sapiens OX=9606 GN=KRT8 PE=1 SV=7
5 peptides

Sequence	Peptide Ion	Score	Hits	Mass	Charge	Tags	Conflicts	Modifications	In quantitation	Drift time (ms)	Average Normalised Abundances	
											CTR	MCD
AFSSR	176	4.96	1	566.2831	1		0		yes	6.58	1.29e+004	2977.25
AFSSR	2153	---	---	566.2821	2		0		yes	2.91	711.38	89.66
ASLEAAIADAEQR	1575	4.73	1	1343.6695	2		0		no	5.12	789.19	961.03
HGDDLRR	931	4.90	1	867.4337	2		0		yes	3.95	2610.13	6745.19
IETRDGK	462	4.68	1	817.4333	1		0		yes	8.66	1283.47	4582.52
IETRDGK	2823	---	---	817.4313	2		0		yes	3.53	95.04	331.41
LQAEIEGLKGQR	2763	5.67	1	1340.7364	3		0		no	3.88	215.84	574.22

Tags	
	Anova p-value ≤ 0.05
	power>0.8
	q<0.05

P55290

Cadherin-13 OS=Homo sapiens OX=9606 GN=CDH13 PE=1 SV=1
5 peptides

Sequence	Peptide Ion	Score	Hits	Mass	Charge	Tags	Conflicts	Modifications	In quantitation	Drift time (ms)	Average Normalised Abundances	
											CTR	MCD
DLHPNTDPFK	6350	4.84	1	1182.5638	2		0		yes	4.57	376.40	160.51
INENTGSVSVTR	5874	5.56	1	1275.6370	2		0		no	4.92	110.64	27.49
SIVVSPILIPENQR	13274	5.18	1	1563.9011	2		0		no	6.02	9.33	1.44
VNSDGGLVALRNITAVGK	4471	4.45	1	1782.9669	3		0		yes	4.57	269.06	48.93
VVDSDRPER	4685	4.66	1	1071.5277	2		0		yes	4.36	283.27	14.43

Tags	
	Anova p-value ≤ 0.05
	power>0.8
	q<0.05

P0DP25

Calmodulin-3 OS=Homo sapiens OX=9606 GN=CALM3 PE=1 SV=1
5 peptides

Sequence	Peptide Ion	Score	Hits	Mass	Charge	Tags	Conflicts	Modifications	In quantitation	Drift time (ms)	Average Normalised Abundances	
											CTR	MCD
EAFSLFDKDGDGTITTK	2518	6.77	3	1843.8836	3		0		yes	4.36	867.70	431.59
ELGTVMR	1495	6.18	3	820.4100	2		0	[6] Oxidation M	yes	3.53	1058.76	439.72
GTVMR	3484	0.00	1	578.2855	1		0	[4] Oxidation M	no	6.37	255.64	60.06
MKDTDSEEEIREAFR	5116	5.45	2	1870.8332	3		0	[2] Hydroxyl DKNP	yes	4.64	326.33	180.67
MKDTDSEEEIREAFR	7220	---	---	1870.8471	4		0	[2] Hydroxyl DKNP	yes	3.81	174.53	52.42
VFDKDGNGYISAAELR	4438	5.72	3	1753.8610	3		0		no	4.36	387.53	159.49

Tags	
	Anova p-value ≤ 0.05
	power>0.8
	q<0.05

Q7Z406

Myosin-14 OS=Homo sapiens OX=9606 GN=MYH14 PE=1 SV=2
4 peptides

Sequence	Peptide Ion	Score	Hits	Mass	Charge	Tags	Conflicts	Modifications	In quantitation	Drift time (ms)	Average Normalised Abundances	
											CTR	MCD
ALSLTR	1935	4.88	1	659.3948	1		0		yes	7.69	288.56	26.19
HEVPPHVYAVTEGAYR	1256	4.52	1	1823.8884	4		0		yes	3.53	709.61	379.51
HEVPPHVYAVTEGAYR	1733	4.52	1	1823.8852	3		0		yes	4.16	648.22	298.75
LKGLEAEVLR	5919	4.81	1	1126.6577	2		0		no	4.99	72.35	8.86
LKGLEAEVLR	13440	---	---	1126.6666	3		0		no	3.53	11.38	0.00
LLGLGVTDFSR	3829	5.35	1	1176.6455	2		0		yes	4.85	249.51	90.92

Tags	
	Anova p-value ≤ 0.05
	power>0.8
	q<0.05

P09382

Galectin-1 OS=Homo sapiens OX=9606 GN=LGALS1 PE=1 SV=2
4 peptides

Sequence	Peptide Ion	Score	Hits	Mass	Charge	Tags	Conflicts	Modifications	In quantitation	Drift time (ms)	Average Normalised Abundances	
											CTR	MCD
LPDGYEFK	2554	5.94	2	967.4668	2		0		yes	3.95	574.63	295.97
LPDGYEFKFPNR	3350	5.92	2	1481.7441	3		0		yes	3.95	343.82	128.80

Sequence	Peptide Ion	Score	Hits	Mass	Charge	Tags	Conflicts	Modifications	In quantitation	Drift time (ms)	Average Normalised Abundances	
											CTR	MCD
LPDGYEFKFPNR	4293	5.45	1	1527.7463	3		0	[8] Methyl K| [10] Hydroxyl DKNP|[11] Hydroxyl DKNP	yes	3.95	274.03	123.21
SFVLNLGK	3884	6.36	1	876.5051	2		0		no	3.81	109.63	8.41

Tags	
	Anova p-value ≤ 0.05
	power>0.8
	q<0.05

P00338

L-lactate dehydrogenase A chain OS=Homo sapiens OX=9606 GN=LDHA PE=1 SV=2
4 peptides

Sequence	Peptide Ion	Score	Hits	Mass	Charge	Tags	Conflicts	Modifications	In quantitation	Drift time (ms)	Average Normalised Abundances	
											CTR	MCD
DKEQWKEVHK	294	5.48	1	1339.6869	2		0	[2] Methyl K	yes	5.47	1.60e+004	4331.9[illegible]
LVIITAGAR	4368	6.78	1	912.5665	2		0		no	3.88	266.62	124.2[illegible]
VIGSGCNLDSARFR	4124	4.39	1	1550.7575	2		0	[6] Carbamidomethyl C	yes	5.96	531.95	61.5[illegible]
VTLTSEEEAR	1714	7.01	4	1133.5576	2		0		yes	4.85	835.18	395.4[illegible]

Tags	
	Anova p-value ≤ 0.05
	power>0.8
	q<0.05

P01591

Immunoglobulin J chain OS=Homo sapiens OX=9606 GN=JCHAIN PE=1 SV=4
3 peptides

Sequence	Peptide Ion	Score	Hits	Mass	Charge	Tags	Conflicts	Modifications	In quantitation	Drift time (ms)	Average Normalised Abundances	
											CTR	MCD
CYTAVVPLVYGGETK	5595	7.96	2	1655.8209	2		0	[1] Carbamidomethyl C	yes	6.30	550.53	254.87
MVETALTPDACYPD	3762	6.87	2	1597.6587	2		0	[1] Oxidation M| [11] Carbamidomethyl C	yes	5.89	522.49	1.09

Sequence	Peptide Ion	Score	Hits	Mass	Charge	Tags	Conflicts	Modifications	In quantitation	Drift time (ms)	Average Normalised Abundances	
											CTR	MCD
SSEDPNEDIVER	1073	8.48	3	1388.6079	2		0		yes	5.19	2279.57	96.41

Tags	
	Anova p-value ≤ 0.05
	power>0.8
	q<0.05

P04075

Fructose-bisphosphate aldolase A OS=Homo sapiens OX=9606 GN=ALDOA PE=1 SV=2
4 peptides

Sequence	Peptide Ion	Score	Hits	Mass	Charge	Tags	Conflicts	Modifications	In quantitation	Drift time (ms)	Average Normalised Abundances	
											CTR	MCD
ADDGRPFPQVIK	2012	4.78	1	1341.7018	3		0		yes	3.81	549.62	331.98
DGADFAKWR	2222	4.21	1	1064.5208	2		0		yes	4.22	743.88	593.86
GILAADESTGSIAK	2121	7.19	2	1331.6848	2		0		no	5.26	531.80	293.89
LQSIGTENTEENRR	2647	6.46	2	1645.8031	3		0		yes	4.09	553.69	382.77

Tags	
	Anova p-value ≤ 0.05
	power>0.8
	q<0.05

P05783

Keratin_ type I cytoskeletal 18 OS=Homo sapiens OX=9606 GN=KRT18 PE=1 SV=2
4 peptides

Sequence	Peptide Ion	Score	Hits	Mass	Charge	Tags	Conflicts	Modifications	In quantitation	Drift time (ms)
AQYDELARK	1480	5.44	1	1092.5514	2		0		no	4.64
GPQVRDWSHYFK	960	5.59	1	1518.7658	2		0		yes	6.58
IVLQIDNAR	1247	6.67	4	1040.5937	2		0		yes	4.50
STSFRGGMGSGGLATGIAGGLAGMGGIQNEK	1423	4.70	1	2868.3949	3		0	[8] Oxidation M|[31] Methyl K	yes	5.75

Tags	
	Anova p-value ≤ 0.05
	power>0.8
	q<0.05

Q01995

Transgelin OS=Homo sapiens OX=9606 GN=TAGLN PE=1 SV=4
4 peptides

Sequence	Peptide Ion	Score	Hits	Mass	Charge	Tags	Conflicts	Modifications	In quantitation	Drift time (ms)	Average Normalised Abundances	
											CTR	MCD
EFTESQLQEGK	5165	6.48	2	1294.6048	2		0		yes	5.06	177.83	105.92
QMEQVAQFLK	4921	5.30	1	1236.6187	2		0	[2] Oxidation M	no	5.47	175.53	47.10
VPENPPSMVFK	2676	5.28	1	1291.6348	2		0	[4] Hydroxyl DKNP|[6] Hydroxyl DKNP|[11] Hydroxyl DKNP	yes	4.92	538.09	166.46
VPENPPSMVFK	4266	5.27	1	1289.6316	2		0	[6] Hydroxyl DKNP|[11] Hydroxyl DKNP|[11] Methyl K	yes	4.99	321.25	125.51

Tags	
	Anova p-value ≤ 0.05
	power>0.8
	q<0.05

P06703

Protein S100-A6 OS=Homo sapiens OX=9606 GN=S100A6 PE=1 SV=1
3 peptides

Sequence	Peptide Ion	Score	Hits	Mass	Charge	Tags	Conflicts	Modifications	In quantitation	Drift time (ms)	Average Normalised Abundances	
											CTR	MCD
ELKELIQK	5796	6.45	2	999.5833	2		0		yes	4.16	96.73	13.84
ELTIGSK	3023	7.08	1	746.4146	2		0		yes	3.25	195.30	28.57
ELTIGSK	9692	---	---	746.4086	1		0		yes	8.31	41.22	0.00
LQDAEIAR	336	8.49	8	914.4840	2		0		yes	3.95	6323.38	3029.62
LQDAEIAR	8199	---	---	914.4807	1		0		yes	10.53	30.30	0.00

Tags	
	Anova p-value ≤ 0.05
	power>0.8
	q<0.05

P29400

Collagen alpha-5(IV) chain OS=Homo sapiens OX=9606 GN=COL4A5 PE=1 SV=2
5 peptides

Sequence	Peptide Ion	Score	Hits	Mass	Charge	Tags	Conflicts	Modifications	In quantitation	Drift time (ms)	Average Normalised Abundances	
											CTR	[illegible]
GDAGPPGIPGQPGLK	172	4.35	1	1375.7039	3		0	[6] Hydroxyl DKNP	yes	3.39	1.30e+004	7[illegible]
GDPGLPGQPGTR	4917	3.87	1	1150.5545	2		0		yes	4.57	142.58	
GDPGLPGVPGFPGMK	3719	4.32	1	1440.7145	3		0	[9] Hydroxyl DKNP	no	3.95	278.44	
GFPGPPGPPGRTGLDGLPGPK	4180	4.44	1	1984.0302	3		0	[21] Methyl K	yes	4.92	506.85	
GLPGIPGAPGAPGFPGSK	4446	4.76	1	1591.8310	2		0	[3] Hydroxyl DKNP	no	6.86	315.86	

Tags	
	Anova p-value ≤ 0.05
	power>0.8
	q<0.05

Q15365

Poly(rC)-binding protein 1 OS=Homo sapiens OX=9606 GN=PCBP1 PE=1 SV=2
4 peptides

Sequence	Peptide Ion	Score	Hits	Mass	Charge	Tags	Conflicts	Modifications	In quantitation	Drift time (ms)	Average Normalised Abundances	
											CTR	MCD
IANPVEGSSGR	3616	6.10	3	1085.5452	2		0		yes	4.22	496.02	210.[illegible]
INISEGNCPER	6967	5.17	1	1287.5917	2		0	[8] Carbamidomethyl C	yes	4.85	121.86	20.[illegible]
LVVPATQCGSLIGKGGCK	9224	4.31	1	1843.9936	3		0	[8] Carbamidomethyl C|[17] Carbamidomethyl C	no	4.57	31.76	0.[illegible]
QGANINEIR	3472	6.05	2	1013.5243	2		0		yes	4.16	747.67	417.[illegible]

Tags	
	Anova p-value ≤ 0.05
	power>0.8
	q<0.05

O00159

Unconventional myosin-Ic OS=Homo sapiens OX=9606 GN=MYO1C PE=1 SV=4
4 peptides

Sequence	Peptide Ion	Score	Hits	Mass	Charge	Tags	Conflicts	Modifications	In quantitation	Drift time (ms)	Average Normalised Abundances	
											CTR	MCD
DGTIDFTPGSELLITK	10401	5.17	2	1705.8783	2		0		no	6.65	69.59	6.80
LLQSNPVLEAFGNAK	6754	5.74	2	1599.8469	2		0		yes	6.09	73.19	5.15
SLASKDVESPSWR	8241	4.80	1	1476.7117	2		0	[5] Hydroxyl DKNP	yes	5.75	133.31	68.51
TLFATEDALEVR	1639	5.85	1	1363.7129	2		0		yes	5.40	337.18	1423.75

Tags	
	Anova p-value ≤ 0.05
	power>0.8
	q<0.05

Q12931

Heat shock protein 75 kDa_ mitochondrial OS=Homo sapiens OX=9606 GN=TRAP1 PE=1 SV=3
3 peptides

Sequence	Peptide Ion	Score	Hits	Mass	Charge	Tags	Conflicts	Modifications	In quantitation	Drift time (ms)	Average Normalised Abundances	
											CTR	MCD
AFLDALQNQAEASSK	5868	5.43	2	1591.7849	3		0		yes	4.02	93.79	41.39
LNELLVK	52	6.11	2	827.5127	1		0		yes	9.83	9900.80	3.08e+004
LNELLVK	1039	5.41	1	827.5106	2		0		yes	3.74	818.22	2224.70
NPAWSLQAGR	2050	4.58	1	1098.5628	2		0		yes	4.43	433.28	331.66

Tags	
	Anova p-value ≤ 0.05
	power>0.8
	q<0.05

P23526

Adenosylhomocysteinase OS=Homo sapiens OX=9606 GN=AHCY PE=1 SV=4
4 peptides

Sequence	Peptide Ion	Score	Hits	Mass	Charge	Tags	Conflicts	Modifications	In quantitation	Drift time (ms)	Average Normalised Abundances	
											CTR	MCD
ALDIAENEMPGLMR	7208	4.71	1	1590.7273	2		0	[7] Hydroxyl DKNP|[13] Oxidation M	no	5.96	148.22	19.00
IILLAEGR	3090	5.33	1	883.5478	2		0		yes	4.02	330.10	54.49
VAVVAGYGDVGK	1988	5.32	1	1133.6061	2		0		yes	4.57	331.65	836.67
VPAINVNDSVTK	1912	5.79	2	1255.6732	2		0		yes	4.99	598.22	1081.27

Tags

Tags	
	Anova p-value ≤ 0.05
	power>0.8
	q<0.05

O76013

Keratin_ type I cuticular Ha6 OS=Homo sapiens OX=9606 GN=KRT36 PE=1 SV=1
4 peptides

Sequence	Peptide Ion	Score	Hits	Mass	Charge	Tags	Conflicts	Modifications	In quantitation	Drift time (ms)	Average Normalised Abundances	
											CTR	MCD
CQYEALVENNR	8029	5.26	1	1394.6258	2		0	[1] Carbamidomethyl C	no	5.47	60.28	8.[illegible]
LEGEIATYRHLLEGEDCK	2013	4.93	1	2132.0206	3		0	[17] Carbamidomethyl C	yes	4.50	1024.35	620.[illegible]
SENARLVLQIDNAK	3600	5.27	1	1569.8438	3		0		yes	4.16	165.47	25.[illegible]
TIEDFQQK	1451	4.99	1	1021.5067	2		0	[8] Methyl K	yes	4.29	1432.55	910.[illegible]

Tags	
	Anova p-value ≤ 0.05
	power>0.8
	q<0.05

Q3LXA3

Triokinase/FMN cyclase OS=Homo sapiens OX=9606 GN=TKFC PE=1 SV=2
5 peptides

Sequence	Peptide Ion	Score	Hits	Mass	Charge	Tags	Conflicts	Modifications	In quantitation	Drift time (ms)	Average Normalise[illegible] Abundanc[illegible]	
											CTR	[illegible]
AILEVLQS	3739	5.05	2	871.5012	1		0		no	10.11	200.32	
LEQPDPGAVAAAAILR	4372	5.90	2	1590.8719	2		0		yes	6.23	295.34	
SRVAPAEPQEAPDSTAAGGSASK	8520	3.86	1	2215.0440	3		0	[5] Hydroxyl DKNP|[23] Hydroxyl DKNP	no	4.50	243.28	
VAPAEPQEAPDSTAAGGSASKR	5122	5.54	2	2096.0098	3		0		yes	5.54	289.69	
YGKAAPGDR+	347	0.00	1	931.4569	2		0	[6] Hydroxyl DKNP| [C-term] Dehydrated	yes	3.88	2274.27	7[illegible]

Tags	
	Anova p-value ≤ 0.05
	power>0.8
	q<0.05

P26006

Integrin alpha-3 OS=Homo sapiens OX=9606 GN=ITGA3 PE=1 SV=5
3 peptides

Sequence	Peptide Ion	Score	Hits	Mass	Charge	Tags	Conflicts	Modifications	In quantitation	Drift time (ms)	Average Normalised Abundances	
											CTR	MCD
AAFVSEQQQK	2494	6.38	2	1134.5629	2		0		yes	4.64	660.33	189.80
SETVLTCATGR	4349	6.74	2	1193.5684	2		0	[7] Carbamidomethyl C	yes	4.64	282.45	86.38
YLLLAGAPR	2669	7.00	2	972.5725	2		0		yes	4.29	339.22	122.47

Tags	
	Anova p-value ≤ 0.05
	power>0.8
	q<0.05

P02743

Serum amyloid P-component OS=Homo sapiens OX=9606 GN=APCS PE=1 SV=2
3 peptides

Sequence	Peptide Ion	Score	Hits	Mass	Charge	Tags	Conflicts	Modifications	In quantitation	Drift time (ms)	Average Normalised Abundances	
											CTR	MCD
IVLGQEQDSYGGK	5109	6.29	1	1392.6869	2		0		yes	5.33	220.40	84.64
IVLGQEQDSYGGKFDR	2042	6.52	3	1810.8844	3		0		yes	4.57	847.95	451.67
VGEYSLYIGR	2603	7.06	3	1155.5960	2		0		yes	4.78	650.78	230.83

Tags	
	Anova p-value ≤ 0.05
	power>0.8
	q<0.05

P23396

40S ribosomal protein S3 OS=Homo sapiens OX=9606 GN=RPS3 PE=1 SV=2
3 peptides

Sequence	Peptide Ion	Score	Hits	Mass	Charge	Tags	Conflicts	Modifications	In quantitation	Drift time (ms)	Average Normalised Abundances	
											CTR	MCD
AELNEFLTR	5615	7.01	2	1091.5636	2		0		yes	4.43	276.37	90.29
DEILPTTPISEQK	8457	5.90	1	1469.7512	2		0		yes	5.61	106.37	15.07
ELAEDGYSGVEVR	4640	6.80	3	1422.6606	2		0		yes	5.40	301.77	148.40

Tags	
	Anova p-value ≤ 0.05
	power>0.8
	q<0.05

Q9NP85

Podocin OS=Homo sapiens OX=9606 GN=NPHS2 PE=1 SV=1
3 peptides

Sequence	Peptide Ion	Score	Hits	Mass	Charge	Tags	Conflicts	Modifications	In quantitation	Drift time (ms)	Average Normalised Abundances	
											CTR	MCD
MAAEILSGTPAAVQLR	5877	6.42	2	1642.8583	2		0	[1] Oxidation M	yes	6.16	158.50	31.30
MAAEILSGTPAAVQLR	13194	---	---	1642.8750	3		0	[1] Oxidation M	yes	4.78	10.63	0.00
SLTEILLER	5314	6.96	1	1072.6093	2		0		yes	4.50	103.83	7.24
TQGSLPFPSPSKPVEPLNPK	4908	6.10	1	2119.0987	3		0		yes	5.26	141.38	5.92

Tags	
	Anova p-value ≤ 0.05
	power>0.8
	q<0.05

P68371

Tubulin beta-4B chain OS=Homo sapiens OX=9606 GN=TUBB4B PE=1 SV=1
3 peptides

Sequence	Peptide Ion	Score	Hits	Mass	Charge	Tags	Conflicts	Modifications	In quantitation	Drift time (ms)	Average Normalised Abundances	
											CTR	MCD
AVLVDLEPGTMDSVR	623	7.11	2	1616.8060	2		0	[12] Hydroxyl DKNP	yes	6.16	2750.84	958.24
AVLVDLEPGTMDSVR	4344	6.11	2	1632.7918	2		0	[8] Hydroxyl DKNP|[12] Hydroxyl DKNP	yes	6.16	263.04	97.04
INVYYNEATGGK	1368	6.10	2	1327.6417	2		0		yes	5.19	1082.81	610.42

Tags	
	Anova p-value ≤ 0.05
	power>0.8
	q<0.05

Q96KP4

Cytosolic non-specific dipeptidase OS=Homo sapiens OX=9606 GN=CNDP2 PE=1 SV=2
3 peptides

Sequence	Peptide Ion	Score	Hits	Mass	Charge	Tags	Conflicts	Modifications	In quantitation	Drift time (ms)	Average Normalised Abundances	
											CTR	MCD
EGGSIPVTLTFQEATGK	5566	6.36	3	1733.8843	2		0		yes	6.44	231.08	85.18
QLGGSVELVDIGK	4025	6.03	3	1313.7082	2		0		yes	5.19	431.30	113.78
TVFGVEPDLTR	1601	6.87	3	1232.6305	2		0		yes	4.78	1128.10	784.38

Tags	
	Anova p-value ≤ 0.05
	power>0.8
	q<0.05

Q9HC38

Glyoxalase domain-containing protein 4 OS=Homo sapiens OX=9606 GN=GLOD4 PE=1 SV=1
4 peptides

Sequence	Peptide Ion	Score	Hits	Mass	Charge	Tags	Conflicts	Modifications	In quantitation	Drift time (ms)	Average Normalised Abundances	
											CTR	MCD
ELPDLEDLMKR	177	4.31	1	1357.7014	3		0		yes	3.74	2.58e+004	1.21e+004
ELPDLEDLMKR	734	---	---	1357.6946	2		0		yes	5.54	5284.67	1451.03
FYRDVLGMK	2723	4.94	1	1127.5672	2		0		yes	4.57	1003.69	724.79
ILTPLVSLDTPGK	6948	5.37	1	1352.7814	2		0		yes	5.40	172.86	5.51
ITEDSFSKPYDGK	7521	4.44	1	1485.6920	2		0		no	5.61	76.22	20.50

Tags	
	Anova p-value ≤ 0.05
	power>0.8
	q<0.05

P17661

Desmin OS=Homo sapiens OX=9606 GN=DES PE=1 SV=3
4 peptides

Sequence	Peptide Ion	Score	Hits	Mass	Charge	Tags	Conflicts	Modifications	In quantitation	Drift time (ms)	Average Norm Abundances	
											CTR	M
IESLNEEIAFLK	9288	5.58	1	1404.7471	2		0		no	5.61	39.71	
LQEEIQLK	2437	4.94	1	999.5759	2		0		yes	4.36	283.31	
TIETRDGEVVSEATQQQHEVL	2190	3.72	1	2368.1537	3		0		yes	6.30	108.57	
VSDLTQAANK	84	4.50	1	1045.5510	2		0		yes	4.22	2.01e+004	4.

Sequence	Peptide Ion	Score	Hits	Mass	Charge	Tags	Conflicts	Modifications	In quantitation	Drift time (ms)	Average Norm[illegible] Abundances	
											CTR	M[illegible]

Tags	
	Anova p-value ≤ 0.05
	power>0.8
	q<0.05

Q08554

Desmocollin-1 OS=Homo sapiens OX=9606 GN=DSC1 PE=1 SV=2
3 peptides

Sequence	Peptide Ion	Score	Hits	Mass	Charge	Tags	Conflicts	Modifications	In quantitation	Drift time (ms)	Average Normalised Abundances	
											CTR	MCD
VQDQDLPNTPHSK	3716	5.44	2	1477.7159	3		0		yes	3.88	832.50	105.09
VTATDLDEPDTLHTR	1015	6.73	2	1682.8118	3		0		yes	4.29	2616.36	800.19
VTIFTVPENCR	2007	6.53	2	1334.6651	2		0	[10] Carbamidomethyl C	yes	5.12	1504.41	352.47

Tags	
	Anova p-value ≤ 0.05
	power>0.8
	q<0.05

P12110

Collagen alpha-2(VI) chain OS=Homo sapiens OX=9606 GN=COL6A2 PE=1 SV=4
4 peptides

Sequence	Peptide Ion	Score	Hits	Mass	Charge	Tags	Conflicts	Modifications	In quantitation	Drift time (ms)	Average Normalised Abundances	
											CTR	MCD
EEGIRLFAVAPNQNLK	6202	3.92	1	1797.9524	2		0		no	6.65	59.16	59.55
GDPGRPGFSYPGPR	1964	4.25	1	1458.6828	2		0		yes	5.26	1200.20	418.34
GPQGALGEPGK	1438	5.95	1	1025.5133	2		0	[9] Hydroxyl DKNP	yes	4.09	719.73	243.45
IDSLSSFKEAVK	4232	4.37	1	1322.7040	3		0		yes	3.67	234.37	127.73

Tags	
	Anova p-value ≤ 0.05
	power>0.8
	q<0.05

P00966

Argininosuccinate synthase OS=Homo sapiens OX=9606 GN=ASS1 PE=1 SV=2
3 peptides

Sequence	Peptide Ion	Score	Hits	Mass	Charge	Tags	Conflicts	Modifications	In quantitation	Drift time (ms)	Average Normalised Abundances	
											CTR	MCD
IDIVENR	1071	6.99	3	857.4590	2		0		yes	3.67	1502.42	485.07
TQDPAKAPNTPDILEIEFK	9177	4.45	1	2126.0816	3		0		no	4.71	138.47	23.95
VFIEDVSR	3496	4.71	1	963.4985	2		0		yes	4.02	413.22	160.77
VFIEDVSR	3953	6.80	2	963.5014	2		0		yes	4.22	370.45	84.86

Tags	
	Anova p-value ≤ 0.05
	power>0.8
	q<0.05

P30041

Peroxiredoxin-6 OS=Homo sapiens OX=9606 GN=PRDX6 PE=1 SV=3
3 peptides

Sequence	Peptide Ion	Score	Hits	Mass	Charge	Tags	Conflicts	Modifications	In quantitation	Drift time (ms)	Average Normalised Abundances	
											CTR	MCD
FHDFLGDSWGILFSHPR	8211	5.22	1	2061.9675	3		0	[7] Hydroxyl DKNP | [16] Hydroxyl DKNP	yes	4.36	55.66	21.27
LPFPIIDDR	4135	6.67	2	1084.5949	2		0		yes	4.50	172.88	15.95
NFDEILR	3143	6.34	2	905.4645	2		0		yes	3.95	219.57	186.17

Tags	
	Anova p-value ≤ 0.05
	power>0.8
	q<0.05

P09493

Tropomyosin alpha-1 chain OS=Homo sapiens OX=9606 GN=TPM1 PE=1 SV=2
3 peptides

Sequence	Peptide Ion	Score	Hits	Mass	Charge	Tags	Conflicts	Modifications	In quantitation	Drift time (ms)	Average Normalised Abundances	
											CTR	MCD
AEQAEADKK	1818	5.05	1	988.4921	2		0		yes	4.16	563.56	278.16
ATDAEADVASLNR	4587	7.24	2	1331.6287	2		0		yes	5.06	324.23	147.82

Sequence	Peptide Ion	Score	Hits	Mass	Charge	Tags	Conflicts	Modifications	In quantitation	Drift time (ms)	Average Normalised Abundances	
											CTR	MCD
SKQLEDELVSLQK	5414	5.91	1	1515.8218	3		0		yes	4.02	89.41	3.29

Tags	
	Anova p-value ≤ 0.05
	power>0.8
	q<0.05

P07900

Heat shock protein HSP 90-alpha OS=Homo sapiens OX=9606 GN=HSP90AA1 PE=1 SV=5
3 peptides

Sequence	Peptide Ion	Score	Hits	Mass	Charge	Tags	Conflicts	Modifications	In quantitation	Drift time (ms)	Average Normalised Abundances	
											CTR	MCD
DQVANSAFVER	3433	7.18	4	1234.6004	2		0		yes	4.78	530.47	356.2[illegible]
HIYYITGETKDQVANSAFVER	6097	4.46	1	2454.2126	3		0	[10] Methyl K	yes	5.19	225.40	38.7[illegible]
NPDDITNEEYGEFYK	6684	6.31	4	1832.7722	2		0		yes	6.65	231.80	40.3[illegible]

Tags	
	Anova p-value ≤ 0.05
	power>0.8
	q<0.05

Q13885

Tubulin beta-2A chain OS=Homo sapiens OX=9606 GN=TUBB2A PE=1 SV=1
2 peptides

Sequence	Peptide Ion	Score	Hits	Mass	Charge	Tags	Conflicts	Modifications	In quantitation	Drift time (ms)	Average Normalised Abundances	
											CTR	MCD
ALTVPELTQQMFDSK	3821	6.12	2	1706.8498	2		0		yes	6.65	502.18	129.57
ALTVPELTQQMFDSK	7229	6.12	2	1706.8506	3		0		yes	4.29	151.07	23.34
ALTVPELTQQMFDSK	9402	5.15	1	1722.8400	2		0	[5] Hydroxyl DKNP	yes	6.72	84.05	3.80

Tags	
	Anova p-value ≤ 0.05
	power>0.8
	q<0.05

P05023

Sodium/potassium-transporting ATPase subunit alpha-1 OS=Homo sapiens OX=9606 GN=ATP1A1 PE=1 SV=1
3 peptides

Sequence	Peptide Ion	Score	Hits	Mass	Charge	Tags	Conflicts	Modifications	In quantitation	Drift time (ms)	Average Normalised Abundances	
											CTR	MCD
AAVPDAVGKCR	6789	5.54	1	1142.5859	2		0	[10] Carbamidomethyl C	yes	4.71	180.53	29.13
LNIPVSQVNPR	3588	5.54	1	1235.6774	2		0		yes	5.06	278.72	71.85
SPDFTNENPLETR	4595	6.28	1	1518.6973	2		0		yes	5.82	292.23	100.66

Tags	
	Anova p-value ≤ 0.05
	power>0.8
	q<0.05

P01040

Cystatin-A OS=Homo sapiens OX=9606 GN=CSTA PE=1 SV=1
3 peptides

Sequence	Peptide Ion	Score	Hits	Mass	Charge	Tags	Conflicts	Modifications	In quantitation	Drift time (ms)	Average Normalised Abundances	
											CTR	MCD
IPGGLSEAKPATPEIQEIVDK	2908	5.35	2	2191.1632	3		0		yes	4.85	1282.56	391.0
TNETYGKLEAVQYK	3274	6.75	1	1642.8218	3		0		yes	4.22	716.63	350.2
TQVVAGTNYYIK	2948	5.07	1	1355.6986	2		0		yes	5.40	534.01	304.8

Tags	
	Anova p-value ≤ 0.05
	power>0.8
	q<0.05

P14550

Aldo-keto reductase family 1 member A1 OS=Homo sapiens OX=9606 GN=AKR1A1 PE=1 SV=3
3 peptides

Sequence	Peptide Ion	Score	Hits	Mass	Charge	Tags	Conflicts	Modifications	In quantitation	Drift time (ms)	Average Normalised Abundances	
											CTR	MCD
GLEVTAYSPLGSSDR	3546	6.33	3	1550.7608	2		0		yes	5.82	568.25	592.39
SPAQILLR	2557	6.14	3	896.5433	2		0		yes	3.88	494.91	130.83
VFDFTFSPEEMK	2276	4.53	1	1491.6441	2		0	[3] Hydroxyl DKNP	yes	5.61	444.89	1572.15

Tags	
	Anova p-value ≤ 0.05
	power>0.8
	q<0.05

P07737

Profilin-1 OS=Homo sapiens OX=9606 GN=PFN1 PE=1 SV=2
3 peptides

Sequence	Peptide Ion	Score	Hits	Mass	Charge	Tags	Conflicts	Modifications	In quantitation	Drift time (ms)	Average Normalised Abundances	
											CTR	MCD
DSLLQDGEFSMDLR	6993	5.75	2	1640.7381	2		0	[6] Hydroxyl DKNP	yes	6.09	177.96	37.91
SSFYVNGLTLGGQK	5079	5.23	1	1485.7373	2		0	[6] Hydroxyl DKNP	yes	5.96	164.72	72.74
TFVNITPAEVGVLVGK	9104	5.76	1	1642.9265	2		0		yes	6.44	51.31	1.06

Tags	
	Anova p-value ≤ 0.05
	power>0.8
	q<0.05

P17066

Heat shock 70 kDa protein 6 OS=Homo sapiens OX=9606 GN=HSPA6 PE=1 SV=2
3 peptides

Sequence	Peptide Ion	Score	Hits	Mass	Charge	Tags	Conflicts	Modifications	In quantitation	Drift time (ms)	Average Normalised Abundances	
											CTR	MCD
DLSGNK	1811	5.11	1	632.3127	1		0		yes	7.27	637.33	206.93
LSKEEVER	6301	6.40	1	988.5107	2		0		yes	4.16	150.77	17.25
LVNHFMEEFR	2182	5.03	1	1336.6249	2		0	[3] Hydroxyl DKNP	yes	5.40	583.38	463.32

Tags	
	Anova p-value ≤ 0.05
	power>0.8
	q<0.05

Q9BR76

Coronin-1B OS=Homo sapiens OX=9606 GN=CORO1B PE=1 SV=1
3 peptides

Sequence	Peptide Ion	Score	Hits	Mass	Charge	Tags	Conflicts	Modifications	In quantitation	Drift time (ms)	Average Normalised Abundances	
											CTR	MCD
AIFLADGK	3635	5.32	1	833.4620	2		0		yes	3.67	210.40	251.43
DADPILISLR	6666	6.04	1	1111.6212	2		0		yes	4.50	93.62	82.33
LEEVMQELR	2986	4.66	1	1145.5957	2		0		yes	4.50	530.72	296.45

Tags	
	Anova p-value ≤ 0.05
	power>0.8
	q<0.05

P01011

Alpha-1-antichymotrypsin OS=Homo sapiens OX=9606 GN=SERPINA3 PE=1 SV=2
3 peptides

Sequence	Peptide Ion	Score	Hits	Mass	Charge	Tags	Conflicts	Modifications	In quantitation	Drift time (ms)	Average Normalised Abundances	
											CTR	MCD
ADLSGITGAR	1096	6.65	2	959.5031	2		0		yes	4.02	905.82	1180.57
LINDYVKNGTR	325	4.51	1	1307.6836	2		0	[7] Hydroxyl DKNP	yes	5.19	1866.20	6590.89
LINDYVKNGTR	1873	4.75	2	1305.7038	2		0	[7] Methyl K	yes	5.26	769.86	378.08

Tags	
	Anova p-value ≤ 0.05
	power>0.8
	q<0.05

Q08380

Galectin-3-binding protein OS=Homo sapiens OX=9606 GN=LGALS3BP PE=1 SV=1
3 peptides

Sequence	Peptide Ion	Score	Hits	Mass	Charge	Tags	Conflicts	Modifications	In quantitation	Drift time (ms)	Average Normalised Abundances	
											CTR	MCD
ELSEALGQIFDSQR	7808	5.32	1	1591.7841	2		0		yes	6.16	202.50	29.24
SDLAVPSELALLK	6001	6.13	1	1354.7671	2		0		yes	5.54	276.96	52.41
SQLVYQSR	1937	4.28	1	979.4954	2		0		yes	4.29	783.40	375.70

Tags	
	Anova p-value ≤ 0.05
	power>0.8
	q<0.05

Q5D862

Filaggrin-2 OS=Homo sapiens OX=9606 GN=FLG2 PE=1 SV=1
3 peptides

Sequence	Peptide Ion	Score	Hits	Mass	Charge	Tags	Conflicts	Modifications	In quantitation	Drift time (ms)	Average Normalised Abundances	
											CTR	MCD
ENGQPQNCGGQWR	2783	4.30	1	1545.6449	3		0	[2] Hydroxyl DKNP|[8] Carbamidomethyl C	yes	3.74	1862.27	418.32
FSNSSSSNEFSK	2378	6.32	1	1319.5655	2		0		yes	5.33	1522.81	469.41
VLSKEYCK	1290	4.78	1	1025.5252	2		0	[7] Carbamidomethyl C	yes	4.16	1843.34	571.09

Tags	
	Anova p-value ≤ 0.05
	power>0.8
	q<0.05

Q9NSB2

Keratin_ type II cuticular Hb4 OS=Homo sapiens OX=9606 GN=KRT84 PE=2 SV=2
3 peptides

Sequence	Peptide Ion	Score	Hits	Mass	Charge	Tags	Conflicts	Modifications	In quantitation	Drift time (ms)	Average Normali[illegible] Abundances	
											CTR	MCD
DLNLDGIIAEVK	85	4.95	1	1328.7222	2		0	[12] Hydroxyl DKNP|[12] Methyl K	yes	5.54	3.84e+004	1.82e[illegible]
LCEGVGPVNISVSSSR	3649	5.24	1	1659.8197	3		0	[2] Carbamidomethyl C	yes	3.95	186.91	[illegible]
SRADAEAWYQTK	8140	5.10	1	1440.6435	2		0	[12] Hydroxyl DKNP	yes	5.89	108.55	[illegible]

Tags	
	Anova p-value ≤ 0.05
	power>0.8
	q<0.05

Q9BSE5

Agmatinase_ mitochondrial OS=Homo sapiens OX=9606 GN=AGMAT PE=1 SV=2
3 peptides

Sequence	Peptide Ion	Score	Hits	Mass	Charge	Tags	Conflicts	Modifications	In quantitation	Drift time (ms)	Average Normalised Abundances	
											CTR	MCD

Sequence	Peptide Ion	Score	Hits	Mass	Charge	Tags	Conflicts	Modifications	In quantitation	Drift time (ms)	Average Normalised Abundances	
											CTR	MCD
GSSTTLDPYR	2934	5.57	1	1095.5174	2		0		yes	4.36	368.09	270.20
GSSTTLDPYRYNR	676	4.96	1	1560.7167	2		0	[7] Hydroxyl DKNP|[8] Hydroxyl DKNP	yes	5.75	4029.97	2118.25
GSSTTLDPYRYNR	1963	4.63	1	1560.7125	2		0	[7] Hydroxyl DKNP|[12] Hydroxyl DKNP	yes	5.68	860.75	350.16

Tags	
	Anova p-value ≤ 0.05
	power>0.8
	q<0.05

P52566

Rho GDP-dissociation inhibitor 2 OS=Homo sapiens OX=9606 GN=ARHGDIB PE=1 SV=3
3 peptides

Sequence	Peptide Ion	Score	Hits	Mass	Charge	Tags	Conflicts	Modifications	In quantitation	Drift time (ms)	Average Normalised Abundances	
											CTR	MCD
APNVVVTR	4220	5.26	1	854.4903	2		0		yes	3.67	162.83	40.05
GTYHNKSFFTDDDK	2912	4.12	1	1673.7171	2		0		yes	6.02	1075.58	551.66
TLLGDGPVVTDPK	3916	5.74	1	1310.6940	2		0		yes	5.19	328.64	128.71

Tags	
	Anova p-value ≤ 0.05
	power>0.8
	q<0.05

O43809

Cleavage and polyadenylation specificity factor subunit 5 OS=Homo sapiens OX=9606 GN=NUDT21 PE=1 SV=1
2 peptides

Sequence	Peptide Ion	Score	Hits	Mass	Charge	Tags	Conflicts	Modifications	In quantitation	Drift time (ms)	Average Normalised Abundances	
											CTR	MCD
DSSVAARFQR	24	3.88	2	1135.5788	2		0		yes	4.64	5.89e+004	1.73e+005
DSSVAARFQR	289	3.88	1	1135.5728	3		0		yes	3.19	4837.33	1.51e+004
EPLYEK	180	5.75	3	777.3917	1		0		yes	8.73	2590.87	1.21e+004
EPLYEK	395	7.28	2	777.3914	1		0		yes	8.66	1126.58	4035.32
EPLYEK	3606	---	---	777.3898	2		0		yes	3.60	78.72	407.74
EPLYEK	4848	---	---	777.3912	2		0		yes	3.60	48.37	206.79

Tags	
	Anova p-value ≤ 0.05
	power>0.8
	q<0.05

P04040

Catalase OS=Homo sapiens OX=9606 GN=CAT PE=1 SV=3
3 peptides

Sequence	Peptide Ion	Score	Hits	Mass	Charge	Tags	Conflicts	Modifications	In quantitation	Drift time (ms)	Average Normalised Abundances	
											CTR	MCD
FNTANDDNVTQVR	2472	5.99	1	1492.6853	2		0		yes	5.61	775.58	251.35
FSTVAGESGSADTVR	3119	4.43	1	1482.6745	2		0		yes	5.54	404.78	130.59
FSTVAGESGSADTVRDPR	1706	4.54	1	1850.8733	3		0		yes	4.71	1055.02	646.27

Tags	
	Anova p-value ≤ 0.05
	power>0.8
	q<0.05

P01859

Immunoglobulin heavy constant gamma 2 OS=Homo sapiens OX=9606 GN=IGHG2 PE=1 SV=2
3 peptides

Sequence	Peptide Ion	Score	Hits	Mass	Charge	Tags	Conflicts	Modifications	In quantitation	Drift time (ms)	Average Normalised Abundances	
											CTR	MCD
GLPAPIEK	2637	6.75	2	823.4789	2		0		yes	3.88	220.81	23.31
LMISR	2292	0.00	1	634.3476	1		0	[2] Oxidation M	yes	7.55	461.50	15.17
LMISR	6438	---	---	634.3469	2		0	[2] Oxidation M	yes	3.32	87.57	1.05
STSESTAALGCLVK	1095	8.19	4	1422.7012	2		0	[11] Carbamidomethyl C	yes	5.47	1759.58	404.42

Tags	
	Anova p-value ≤ 0.05
	power>0.8
	q<0.05

Q13642

Four and a half LIM domains protein 1 OS=Homo sapiens OX=9606 GN=FHL1 PE=1 SV=4
3 peptides

Sequence	Peptide Ion	Score	Hits	Mass	Charge	Tags	Conflicts	Modifications	In quantitation	Drift time (ms)	Average Normalised Abundances	
											CTR	MCD
AIVAGDQNVEYK	5287	5.06	1	1305.6480	2		0		yes	5.40	108.32	38.24
DNPGTTTASTAKNAP	7621	4.68	1	1458.6944	2		0	[12] Methyl K	yes	5.61	112.24	44.24
QVIGTGSFFPK	11316	5.02	1	1179.6180	2		0		yes	4.85	46.36	13.12

Tags	
	Anova p-value ≤ 0.05
	power>0.8
	q<0.05

P50395

Rab GDP dissociation inhibitor beta OS=Homo sapiens OX=9606 GN=GDI2 PE=1 SV=2
2 peptides

Sequence	Peptide Ion	Score	Hits	Mass	Charge	Tags	Conflicts	Modifications	In quantitation	Drift time (ms)	Average Normalised Abundances	
											CTR	MCD
DLGTESQIFISR	6065	5.99	1	1364.6854	2		0		yes	5.33	78.21	119.70
VTEGSFVYK	5190	4.33	1	1028.5176	3		0		yes	3.05	79.55	1.49
VTEGSFVYK	2410	4.33	1	1028.5183	2		0		yes	4.43	1133.69	161.82

Tags	
	Anova p-value ≤ 0.05
	power>0.8
	q<0.05

P06727

Apolipoprotein A-IV OS=Homo sapiens OX=9606 GN=APOA4 PE=1 SV=4
3 peptides

Sequence	Peptide Ion	Score	Hits	Mass	Charge	Tags	Conflicts	Modifications	In quantitation	Drift time (ms)	Average Norma Abundances	
											CTR	MC
IDQTVEELR	1493	3.91	1	1117.5575	2		0	[2] Hydroxyl DKNP	yes	4.43	505.68	1
LAPLAEDVR	4944	5.89	1	982.5400	2		0		yes	4.22	243.20	
QKLGPHAGDVEGHLSFLEK	134	4.76	1	2077.0632	3		0	[2] Hydroxyl DKNP	yes	4.64	2.36e+004	1.1

Tags	
	Anova p-value ≤ 0.05
	power>0.8
	q<0.05

Q14240

Eukaryotic initiation factor 4A-II OS=Homo sapiens OX=9606 GN=EIF4A2 PE=1 SV=2
3 peptides

Sequence	Peptide Ion	Score	Hits	Mass	Charge	Tags	Conflicts	Modifications	In quantitation	Drift time (ms)	Average Normalised Abundances	
											CTR	MCD
DVIMREFR	2563	4.12	1	1064.5364	2		0		yes	4.36	809.94	549.96
ERDVIMR	4922	4.63	1	917.4584	2		0		yes	3.88	274.46	75.84
VLITTDLLAR	3816	5.66	2	1113.6753	2		0		yes	4.64	238.32	89.62

Tags	
	Anova p-value ≤ 0.05
	power>0.8
	q<0.05

P55060

Exportin-2 OS=Homo sapiens OX=9606 GN=CSE1L PE=1 SV=3
2 peptides

Sequence	Peptide Ion	Score	Hits	Mass	Charge	Tags	Conflicts	Modifications	In quantitation	Drift time (ms)	Average Normalised Abundances	
											CTR	MCD
NLFEDQNTLTSICEK	3592	4.57	1	1810.8489	3		0	[13] Carbamidomethyl C	yes	4.50	548.38	348.45
QLSDAISIIGREDFPQK	2301	4.84	1	1930.0391	3		0	[17] Methyl K	yes	5.06	612.30	358.57
QLSDAISIIGREDFPQK	1903	4.84	1	1930.0322	2		0	[17] Methyl K	yes	7.41	660.02	400.75

Tags	
	Anova p-value ≤ 0.05
	power>0.8
	q<0.05

P22061

Protein-L-isoaspartate(D-aspartate) O-methyltransferase OS=Homo sapiens OX=9606 GN=PCMT1 PE=1 SV=4
2 peptides

Sequence	Peptide Ion	Score	Hits	Mass	Charge	Tags	Conflicts	Modifica
MGYAEEAPYDAIHVGAAAPVVPQALIDQLKPGGRLILPVGPAGGNQMLEQYDK	275	3.98	1	5573.8191	6		0	[45] Hyd DKNP Hyd DKNP Met
MGYAEEAPYDAIHVGAAAPVVPQALIDQLKPGGRLILPVGPAGGNQMLEQYDK	579	3.98	1	5573.8354	5		0	[45] Hyd DKNP Hyd DKNP Met
VQLVVGDGR	3190	6.16	2	941.5315	2		0	

Tags	
	Anova p-value ≤ 0.05
	power>0.8
	q<0.05

P20700

Lamin-B1 OS=Homo sapiens OX=9606 GN=LMNB1 PE=1 SV=2
3 peptides

Sequence	Peptide Ion	Score	Hits	Mass	Charge	Tags	Conflicts	Modifications	In quantitation	Drift time (ms)	Average Normalised Abundances	
											CTR	MCD
CQSLTEDLEFR	6354	4.45	1	1396.6498	2		0	[1] Carbamidomethyl C	yes	5.33	125.76	74.19
IESLSSQLSNLQK	1149	4.78	1	1445.7712	2		0		yes	5.82	1027.53	841.72
LALDMEISAYRK	5265	4.52	1	1408.7280	2		0		yes	5.82	615.24	1870.10

Tags	
	Anova p-value ≤ 0.05
	power>0.8
	q<0.05

P04899

Guanine nucleotide-binding protein G(i) subunit alpha-2 OS=Homo sapiens OX=9606 GN=GNAI2 PE=1 SV=3
2 peptides

Sequence	Peptide Ion	Score	Hits	Mass	Charge	Tags	Conflicts	Modifications	In quantitation	Drift time (ms)	Average Normalised Abundances	
											CTR	MCD
IAQSDYIPTQQDVLR	5384	6.55	2	1745.8886	2		0		yes	6.58	114.25	20.38
LLLLGAGESGK	2194	6.90	2	1056.6167	2		0		yes	4.50	479.30	244.17

Tags

	Anova p-value ≤ 0.05
	power>0.8
	q<0.05

P52943

Cysteine-rich protein 2 OS=Homo sapiens OX=9606 GN=CRIP2 PE=1 SV=1
2 peptides

Sequence	Peptide Ion	Score	Hits	Mass	Charge	Tags	Conflicts	Modifications	In quantitation	Drift time (ms)	Average Normalised Abundances	
											CTR	MCD
ASSVTTFTGEPNTCPR	6880	6.75	2	1723.7824	2		0	[14] Carbamidomethyl C	yes	6.30	103.42	47
GVNTGAVGSYIYDRDPEGK	4088	6.48	2	1996.9485	3		0		yes	4.29	231.50	143

Tags	
	Anova p-value ≤ 0.05
	power>0.8
	q<0.05

P04439

HLA class I histocompatibility antigen_ A alpha chain OS=Homo sapiens OX=9606 GN=HLA-A PE=1 SV=2
2 peptides

Sequence	Peptide Ion	Score	Hits	Mass	Charge	Tags	Conflicts	Modifications	In quantitation	Drift time (ms)	Average Normalised Abundances	
											CTR	MCD
FDSDAASQR	3737	6.98	2	995.4315	2		0		yes	4.02	171.04	36.60
FIAVGYVDDTQFVR	3738	6.23	1	1628.8087	2		0		yes	6.23	428.44	190.26

Tags	
	Anova p-value ≤ 0.05
	power>0.8
	q<0.05

Q7Z794

Keratin_ type II cytoskeletal 1b OS=Homo sapiens OX=9606 GN=KRT77 PE=1 SV=3
3 peptides

Sequence	Peptide Ion	Score	Hits	Mass	Charge	Tags	Conflicts	Modifications	In quantitation	Drift time (ms)	Ave Abu
											CT
GEQALQDAWQK	7026	5.01	1	1272.6103	2		0		yes	5.12	

Sequence	Peptide Ion	Score	Hits	Mass	Charge	Tags	Conflicts	Modifications	In quantitation	Drift time (ms)	Ave Abu
											CT
QIEQMQSLISDAEERGEQALQDAWQK	225	4.04	1	3030.4615	4		0		yes	5.89	2
QIEQMQSLISDAEERGEQALQDAWQK	2612	4.04	1	3030.4595	3		0		yes	8.03	
VYSTSSSAGSGGGSPAVGSVCYARGR	59	---	---	2476.1534	5		0	[21] Carbamidomethyl C	yes	4.22	2.9

Tags	
	Anova p-value ≤ 0.05
	power>0.8
	q<0.05

Q9BW30

Tubulin polymerization-promoting protein family member 3 OS=Homo sapiens OX=9606 GN=TPPP3 PE=1 SV=1
2 peptides

Sequence	Peptide Ion	Score	Hits	Mass	Charge	Tags	Conflicts	Modifications	In quantitation	Drift time (ms)	Average Normalised Abundances	
											CTR	MCD
QDILDDSGYVSAYK	7113	6.29	2	1572.7262	2		0		yes	6.16	122.87	21.39
SVTGTDVDIVFSK	4074	6.62	2	1366.6924	2		0		yes	5.26	269.89	138.52

Tags	
	Anova p-value ≤ 0.05
	power>0.8
	q<0.05

P61626

Lysozyme C OS=Homo sapiens OX=9606 GN=LYZ PE=1 SV=1
2 peptides

Sequence	Peptide Ion	Score	Hits	Mass	Charge	Tags	Conflicts	Modifications	In quantitation	Drift time (ms)	A N A
											C
STDYGIFQINSR	1346	7.47	6	1399.6669	2		0		yes	5.40	1
TPGAVNACHLSCSALLQDNIADAVACAK	3481	5.08	1	2958.3312	3		0	[6] Hydroxyl DKNP | [8] Carbamidomethyl C | [12] Carbamidomethyl C | [18] Hydroxyl DKNP | [26] Carbamidomethyl C	yes	5.75	

Tags	
	Anova p-value ≤ 0.05

	power>0.8
	q<0.05

P21291

Cysteine and glycine-rich protein 1 OS=Homo sapiens OX=9606 GN=CSRP1 PE=1 SV=3
2 peptides

Sequence	Peptide Ion	Score	Hits	Mass	Charge	Tags	Conflicts	Modifications	In quantitation	Drift time (ms)	Average Normalised Abundances	
											CTR	MCD
GYGYGQGAGTLSTDK	9577	5.92	1	1473.6607	2		0		yes	5.54	73.64	4.79
GYGYGQGAGTLSTDKGESLGIK	8130	6.49	1	2158.0458	3		0		yes	4.99	74.57	2.97

Tags	
	Anova p-value ≤ 0.05
	power>0.8
	q<0.05

P61224

Ras-related protein Rap-1b OS=Homo sapiens OX=9606 GN=RAP1B PE=1 SV=1
2 peptides

Sequence	Peptide Ion	Score	Hits	Mass	Charge	Tags	Conflicts	Modifications	In quantitation	Drift time (ms)	Average Normalised Abundances	
											CTR	MCD
LVVLGSGGVGK	2509	6.72	2	984.5900	2		0		yes	4.16	4108.20	1.36e+004
YDPTIEDSYRK	7751	5.67	1	1385.6487	3		0		yes	3.74	85.39	18.68

Tags	
	Anova p-value ≤ 0.05
	power>0.8
	q<0.05

P05109

Protein S100-A8 OS=Homo sapiens OX=9606 GN=S100A8 PE=1 SV=1
2 peptides

Sequence	Peptide Ion	Score	Hits	Mass	Charge	Tags	Conflicts	Modifications	In quantitation	Drift time (ms)	Average Normalised Abundances	
											CTR	MCD
ALNSIIDVYHK	5671	4.96	1	1271.6987	2		0		yes	5.26	131.49	12.54
LLETECPQYIR	1660	7.11	4	1420.6991	2		0		yes	5.54	1767.71	538.53

Sequence	Peptide Ion	Score	Hits	Mass	Charge	Tags	Conflicts	Modifications	In quantitation	Drift time (ms)	Average Normalised Abundances	
											CTR	MCD
								[6] Carbamidomethyl C				

Tags	
	Anova p-value ≤ 0.05
	power>0.8
	q<0.05

P31946

14-3-3 protein beta/alpha OS=Homo sapiens OX=9606 GN=YWHAB PE=1 SV=3
2 peptides

Sequence	Peptide Ion	Score	Hits	Mass	Charge	Tags	Conflicts	Modifications	In quantitation	Drift time (ms)	Average Normalised Abundances	
											CTR	MCD
AVTEQGHELSNEER	7851	5.01	1	1597.7340	3		0		yes	3.88	94.88	10.66
YLSEVASGDNK	4154	6.90	2	1181.5560	2		0		yes	4.64	254.37	47.82

Tags	
	Anova p-value ≤ 0.05
	power>0.8
	q<0.05

P02760

Protein AMBP OS=Homo sapiens OX=9606 GN=AMBP PE=1 SV=1
2 peptides

Sequence	Peptide Ion	Score	Hits	Mass	Charge	Tags	Conflicts	Modifications	In quantitation	Drift time (ms)	Average Normalised Abundances	
											CTR	MCD
ETLLQDFR	1310	7.17	4	1020.5250	2		0		yes	4.29	1244.50	475.14
TVAACNLPIVR	2297	4.51	1	1212.6636	2		0	[5] Carbamidomethyl C	yes	4.92	469.89	302.91

Tags	
	Anova p-value ≤ 0.05
	power>0.8
	q<0.05

P13646

Keratin_ type I cytoskeletal 13 OS=Homo sapiens OX=9606 GN=KRT13 PE=1 SV=4
2 peptides

Sequence	Peptide Ion	Score	Hits	Mass	Charge	Tags	Conflicts	Modifications	In quantitation	Drift time (ms)	Average Normalised Abundances	
											CTR	MCD
ILTATIENNR	4379	6.37	2	1143.6118	2		0		yes	4.71	311.35	178.21
VLDELTLSK	55	5.31	1	1030.5984	2		0	[9] Methyl K	yes	4.29	6.34e+004	3.94e+004

Tags	
	Anova p-value ≤ 0.05
	power>0.8
	q<0.05

Q9UN36

Protein NDRG2 OS=Homo sapiens OX=9606 GN=NDRG2 PE=1 SV=2
2 peptides

Sequence	Peptide Ion	Score	Hits	Mass	Charge	Tags	Conflicts	Modifications	In quantitation	Drift time (ms)	Average Normalised Abundances	
											CTR	MCD
RPAILTYHDVGLNYK	9257	4.83	1	1758.9356	2		0		yes	6.99	50.81	0.23
RPAILTYHDVGLNYK	14383	---	---	1758.9577	3		0		yes	4.57	11.24	0.00
TASLTSAASVDGNR	4479	6.82	2	1348.6538	2		0		yes	5.19	143.83	3.35

Tags	
	Anova p-value ≤ 0.05
	power>0.8
	q<0.05

P05089

Arginase-1 OS=Homo sapiens OX=9606 GN=ARG1 PE=1 SV=2
2 peptides

Sequence	Peptide Ion	Score	Hits	Mass	Charge	Tags	Conflicts	Modifications	In quantitation	Drift time (ms)	Average Normalised Abundances	
											CTR	MCD
TIGIIGAPFSK	3232	6.42	2	1102.6365	2		0		yes	4.64	629.59	80.74
YFSMTEVDR	4043	5.21	1	1146.5096	2		0		yes	4.50	993.66	79.89

Tags	
	Anova p-value ≤ 0.05
	power>0.8

	q<0.05

P00403

Cytochrome c oxidase subunit 2 OS=Homo sapiens OX=9606 GN=MT-CO2 PE=1 SV=1
2 peptides

Sequence	Peptide Ion	Score	Hits	Mass	Charge	Tags	Conflicts	Modifications	In quantitation	Drift time (ms)	Average Normalised Abundances	
											CTR	MCD
LLDVDNR	1311	5.22	1	843.4538	2		0		yes	3.60	1659.71	1214.59
LLDVDNR	13258	---	---	843.4557	1		0		yes	9.21	43.56	0.00
VVLPIEAPIR	3793	6.22	1	1105.6858	2		0		yes	4.50	187.20	93.23

Tags	
	Anova p-value ≤ 0.05
	power>0.8
	q<0.05

Q562R1

Beta-actin-like protein 2 OS=Homo sapiens OX=9606 GN=ACTBL2 PE=1 SV=2
2 peptides

Sequence	Peptide Ion	Score	Hits	Mass	Charge	Tags	Conflicts	Modifications	In quantitation	Drift time (ms)	Average Normalised Abundances	
											CTR	MC[illegible]
EIITLAPSTMK	1161	5.57	1	1202.6468	2		0		yes	4.78	2943.34	16[illegible]
KDLYANTVLSGGSTMYPGIADR	984	5.86	1	2358.1543	3		0	[1] Methyl K | [15] Oxidation M	yes	5.19	1601.46	6[illegible]

Tags	
	Anova p-value ≤ 0.05
	power>0.8
	q<0.05

P06753

Tropomyosin alpha-3 chain OS=Homo sapiens OX=9606 GN=TPM3 PE=1 SV=2
2 peptides

Sequence	Peptide Ion	Score	Hits	Mass	Charge	Tags	Conflicts	Modifications	In quantitation	Drift time (ms)	Average Normalised Abundances	
											CTR	MCD
AADAEAEVASLNRR	2339	6.17	2	1471.7379	3		0		yes	4.16	460.30	276.43
KAADAEAEVASLNR	5366	5.11	1	1457.7353	2		0	[1] Methyl K	yes	5.75	246.97	100.85

Sequence	Peptide Ion	Score	Hits	Mass	Charge	Tags	Conflicts	Modifications	In quantitation	Drift time (ms)	Average Normalised Abundances	
											CTR	MCD

Tags	
	Anova p-value ≤ 0.05
	power>0.8
	q<0.05

P42765

3-ketoacyl-CoA thiolase_ mitochondrial OS=Homo sapiens OX=9606 GN=ACAA2 PE=1 SV=2
2 peptides

Sequence	Peptide Ion	Score	Hits	Mass	Charge	Tags	Conflicts	Modifications	In quantitation	Drift time (ms)	Average Normalised Abundances	
											CTR	MCD
ETPALTINR	2929	6.18	1	1013.5474	2		0		yes	4.22	337.17	358.53
SLDLDISK	2854	5.01	1	889.4814	2		0		yes	3.95	384.47	544.46

Tags	
	Anova p-value ≤ 0.05
	power>0.8
	q<0.05

Q9NZT1

Calmodulin-like protein 5 OS=Homo sapiens OX=9606 GN=CALML5 PE=1 SV=2
2 peptides

Sequence	Peptide Ion	Score	Hits	Mass	Charge	Tags	Conflicts	Modifications	In quantitation	Drift time (ms)	Average Normalised Abundances	
											CTR	MCD
ARAGLEDLQVAFR	3319	4.73	1	1460.7717	2		0	[7] Hydroxyl DKNP	yes	5.89	768.30	250.05
NLSEAQLR	903	6.39	5	929.4886	2		0		yes	3.95	3736.67	2656.23

Tags	
	Anova p-value ≤ 0.05
	power>0.8
	q<0.05

P52758

2-iminobutanoate/2-iminopropanoate deaminase OS=Homo sapiens OX=9606 GN=RIDA PE=1 SV=1
2 peptides

Sequence	Peptide Ion	Score	Hits	Mass	Charge	Tags	Conflicts	Modifications	In quantitation	Drift time (ms)	Average Normalised Abundances	
											CTR	MCD
AAGCDFTNVVK	114	4.94	1	1194.5751	2		0	[4] Carbamidomethyl C|[11] Methyl K	yes	4.85	9478.10	2.82e+004
AAYQVAALPK	1439	6.14	1	1030.5686	2		0		yes	4.43	1463.77	638.61

Tags	
	Anova p-value ≤ 0.05
	power>0.8
	q<0.05

Q13228

Methanethiol oxidase OS=Homo sapiens OX=9606 GN=SELENBP1 PE=1 SV=2
2 peptides

Sequence	Peptide Ion	Score	Hits	Mass	Charge	Tags	Conflicts	Modifications	In quantitation	Drift time (ms)	Average Normalised Abundances	
											CTR	MCD
IYVVDVGSEPR	2422	5.97	1	1232.6344	2		0		yes	5.06	947.88	277.49
NTGTEAPDYLATVDVDPK	6117	5.11	1	1904.9026	2		0		yes	7.06	184.46	20.55

Tags	
	Anova p-value ≤ 0.05
	power>0.8
	q<0.05

P61978

Heterogeneous nuclear ribonucleoprotein K OS=Homo sapiens OX=9606 GN=HNRNPK PE=1 SV=1
2 peptides

Sequence	Peptide Ion	Score	Hits	Mass	Charge	Tags	Conflicts	Modifications	In quantitation	Drift time (ms)	Average Normalised Abundances	
											CTR	MCD
DYDDMSPR	5544	5.32	1	1013.3758	2		0	[4] Hydroxyl DKNP	yes	4.22	109.34	13.02
TDYNASVSVPDSSGPER	6124	5.68	1	1779.7916	2		0		yes	6.44	142.57	115.40

Tags	
	Anova p-value ≤ 0.05

	power>0.8
	q<0.05

P13797

Plastin-3 OS=Homo sapiens OX=9606 GN=PLS3 PE=1 SV=4
3 peptides

Sequence	Peptide Ion	Score	Hits	Mass	Charge	Tags	Conflicts	Modifications	In quantitation	Drift time (ms)	Average Normalised Abundances	
											CTR	MCD
FSADIK	3188	0.00	1	679.3546	1		0		yes	7.62	170.61	47.78
INNFSADIK	606	5.03	1	1020.5291	2		0		yes	4.09	1455.32	737.94
VYALPEDLVEVKPK	2514	5.83	1	1598.8912	3		0		yes	4.22	496.38	199.42

Tags	
	Anova p-value ≤ 0.05
	power>0.8
	q<0.05

P54652

Heat shock-related 70 kDa protein 2 OS=Homo sapiens OX=9606 GN=HSPA2 PE=1 SV=1
2 peptides

Sequence	Peptide Ion	Score	Hits	Mass	Charge	Tags	Conflicts	Modifications	In quantitation	Drift time (ms)	Average Normalised Abundances	
											CTR	MCD
DAGTITGLNVLR	5630	6.37	1	1228.6698	2		0		yes	4.99	139.68	46.80
VAAKNALESYTYNIK	6499	4.44	1	1683.8898	2		0		yes	6.58	203.49	96.69

Tags	
	Anova p-value ≤ 0.05
	power>0.8
	q<0.05

P37837

Transaldolase OS=Homo sapiens OX=9606 GN=TALDO1 PE=1 SV=2
2 peptides

Sequence	Peptide Ion	Score	Hits	Mass	Charge	Tags	Conflicts	Modifications	In quantitation	Drift time (ms)	Average Normalised Abundances	
											CTR	MCD
ILDWHVANTDKK	2267	5.18	1	1438.7524	2		0		yes	5.75	310.18	189.12
LLGELLQDNAK	3659	5.58	1	1212.6612	2		0		yes	5.06	312.42	271.20

Sequence	Peptide Ion	Score	Hits	Mass	Charge	Tags	Conflicts	Modifications	In quantitation	Drift time (ms)	Average Normalised Abundances	
											CTR	MCD

Tags	
	Anova p-value ≤ 0.05
	power>0.8
	q<0.05

P09496

Clathrin light chain A OS=Homo sapiens OX=9606 GN=CLTA PE=1 SV=1
2 peptides

Sequence	Peptide Ion	Score	Hits	Mass	Charge	Tags	Conflicts	Modifications	In quantitation	Drift time (ms)	Average Normalised Abundances	
											CTR	MCD
EEQMERLEALDANSR	1232	4.85	1	1789.8372	2		0		yes	6.44	3531.74	578.75
LEALDANSR	5091	5.89	1	987.4985	2		0		yes	4.16	523.80	150.31

Tags	
	Anova p-value ≤ 0.05
	power>0.8
	q<0.05

Q7Z4W1

L-xylulose reductase OS=Homo sapiens OX=9606 GN=DCXR PE=1 SV=2
2 peptides

Sequence	Peptide Ion	Score	Hits	Mass	Charge	Tags	Conflicts	Modifications	In quantitation	Drift time (ms)	Average Normalised Abundances	
											CTR	MCD
MELFLAGR	1443	4.14	1	935.5085	2		0		yes	3.88	917.39	457.10
TQADLDSLVR	3036	6.58	1	1116.5744	2		0		yes	4.43	655.72	316.92

Tags	
	Anova p-value ≤ 0.05
	power>0.8
	q<0.05

P30038

Delta-1-pyrroline-5-carboxylate dehydrogenase_ mitochondrial OS=Homo sapiens OX=9606 GN=ALDH4A1 PE=1 SV=3
2 peptides

Sequence	Peptide Ion	Score	Hits	Mass	Charge	Tags	Conflicts	Modifications	In quantitation	Drift time (ms)	Average Normalised Abundances	
											CTR	MCD
SADVESVVSGTLR	4790	5.33	1	1318.6648	2		0		yes	5.12	240.94	111.42
STGSIVGQQPFGGAR	5228	5.37	1	1460.7298	2		0		yes	5.40	145.85	87.48

Tags	
	Anova p-value ≤ 0.05
	power>0.8
	q<0.05

Q9UBI6

Guanine nucleotide-binding protein G(I)/G(S)/G(O) subunit gamma-12 OS=Homo sapiens OX=9606 GN=GNG12 PE=1 SV=3
2 peptides

Sequence	Peptide Ion	Score	Hits	Mass	Charge	Tags	Conflicts	Modifications	In quantitation	Drift time (ms)	Average Normalised Abundances	
											CTR	MCD
SDPLLIGIPTSENPFKDK	1805	5.15	1	1984.0447	3		0	[16] Methyl K	yes	4.99	760.27	220.09
TASTNNIAQAR	7756	5.54	1	1145.5731	2		0		yes	4.50	93.77	4.60

Tags	
	Anova p-value ≤ 0.05
	power>0.8
	q<0.05

P10809

60 kDa heat shock protein_ mitochondrial OS=Homo sapiens OX=9606 GN=HSPD1 PE=1 SV=2
2 peptides

Sequence	Peptide Ion	Score	Hits	Mass	Charge	Tags	Conflicts	Modifications	In quantitation	Drift time (ms)	Average Normalised Abundances	
											CTR	MCD
DGKTLNDELEIIEGMK	1540	4.28	1	1819.8911	2		0	[1] Hydroxyl DKNP	yes	6.86	690.39	922.04
VTDALNATR	1789	6.41	1	959.4989	2		0		yes	4.02	861.22	921.08

Tags	
	Anova p-value ≤ 0.05

	power>0.8
	q<0.05

P14923

Junction plakoglobin OS=Homo sapiens OX=9606 GN=JUP PE=1 SV=3
2 peptides

Sequence	Peptide Ion	Score	Hits	Mass	Charge	Tags	Conflicts	Modifications	In quantitation	Drift time (ms)	Average Normalised Abundances	
											CTR	MCD
AGDKDDITEPAVCALR	3437	5.78	1	1729.8285	3		0	[13] Carbamidomethyl C	yes	4.22	815.69	80.32
LLNDEDPVVVTK	2183	4.90	1	1340.6938	2		0		yes	5.26	1412.34	600.28

Tags	
	Anova p-value ≤ 0.05
	power>0.8
	q<0.05

P38646

Stress-70 protein_ mitochondrial OS=Homo sapiens OX=9606 GN=HSPA9 PE=1 SV=2
2 peptides

Sequence	Peptide Ion	Score	Hits	Mass	Charge	Tags	Conflicts	Modifications	In quantitation	Drift time (ms)	Average Normalised Abundances	
											CTR	MCD
DQLPADECNKLK	777	4.17	1	1443.7042	3		0	[8] Carbamidomethyl C|[10] Methyl K	yes	4.16	1495.86	6075.22
TTPSVVAFTADGER	4089	6.33	1	1449.7052	2		0		yes	5.47	474.94	391.64

Tags	
	Anova p-value ≤ 0.05
	power>0.8
	q<0.05

P12277

Creatine kinase B-type OS=Homo sapiens OX=9606 GN=CKB PE=1 SV=1
2 peptides

Sequence	Peptide Ion	Score	Hits	Mass	Charge	Tags	Conflicts	Modifications	In quantitation	Drift time (ms)	Average Normalised Abundances	
											CTR	MCD

Sequence	Peptide Ion	Score	Hits	Mass	Charge	Tags	Conflicts	Modifications	In quantitation	Drift time (ms)	Average Normalised Abundances	
											CTR	MCD
VISMQKGGNMK	4384	3.87	1	1207.5947	2		0	[11] Hydroxyl DKNP	yes	4.85	206.49	83.83
VLTPELYAELR	3106	6.52	2	1302.7140	2		0		yes	5.26	242.83	74.95

Tags	
	Anova p-value ≤ 0.05
	power>0.8
	q<0.05

P23246

Splicing factor_ proline- and glutamine-rich OS=Homo sapiens OX=9606 GN=SFPQ PE=1 SV=2
2 peptides

Sequence	Peptide Ion	Score	Hits	Mass	Charge	Tags	Conflicts	Modifications	In quantitation	Drift time (ms)	Average Normalised Abundances	
											CTR	MCD
FGQGGAGPVGGQGPR	4577	5.64	1	1340.6527	2		0		yes	4.92	124.61	64.30
SEEKISDSEGFK	7051	4.64	1	1354.6269	3		0		yes	3.88	69.04	10.14

Tags	
	Anova p-value ≤ 0.05
	power>0.8
	q<0.05

P61313

60S ribosomal protein L15 OS=Homo sapiens OX=9606 GN=RPL15 PE=1 SV=2
2 peptides

Sequence	Peptide Ion	Score	Hits	Mass	Charge	Tags	Conflicts	Modifications	In quantitation	Drift time (ms)	Average Normalised Abundances	
											CTR	MCD
QSDVMRFLLR	2105	4.39	1	1263.6673	2		0		yes	5.40	342.71	278.98
SLQSVAEER	3416	5.83	2	1017.5075	2		0		yes	4.22	587.98	227.09

Tags	
	Anova p-value ≤ 0.05
	power>0.8
	q<0.05

P62424

60S ribosomal protein L7a OS=Homo sapiens OX=9606 GN=RPL7A PE=1 SV=2
2 peptides

Sequence	Peptide Ion	Score	Hits	Mass	Charge	Tags	Conflicts	Modifications	In quantitation	Drift time (ms)	Average Normalised Abundances	
											CTR	MCD
NFGIGQDIQPK	3498	5.75	1	1215.6265	2		0		yes	4.92	485.66	287.14
VVNPLFEK	2244	4.36	1	944.5420	2		0		yes	4.02	366.88	200.45

Tags	
	Anova p-value ≤ 0.05
	power>0.8
	q<0.05

Q15366

Poly(rC)-binding protein 2 OS=Homo sapiens OX=9606 GN=PCBP2 PE=1 SV=1
2 peptides

Sequence	Peptide Ion	Score	Hits	Mass	Charge	Tags	Conflicts	Modifications	In quantitation	Drift time (ms)	Average Normalised Abundances	
											CTR	MCD
IANPVEGSTDR	6147	5.45	2	1157.5656	2		0		yes	4.50	243.23	74.64
LVVPASQCGSLIGK	951	4.53	1	1427.7788	2		0	[8] Carbamidomethyl C	yes	5.68	2524.25	1198.98

Tags	
	Anova p-value ≤ 0.05
	power>0.8
	q<0.05

P50995

Annexin A11 OS=Homo sapiens OX=9606 GN=ANXA11 PE=1 SV=1
2 peptides

Sequence	Peptide Ion	Score	Hits	Mass	Charge	Tags	Conflicts	Modifications	In quantitation	Drift time (ms)	Average Normalised Abundances	
											CTR	MCD
DAQELYAAGENR	5846	4.43	1	1335.6001	2		0		yes	5.12	130.37	26.21
SETDLLDIR	4808	5.41	1	1060.5362	2		0		yes	4.29	202.26	148.52

Tags	
	Anova p-value ≤ 0.05
	power>0.8

	q<0.05

Q14195

Dihydropyrimidinase-related protein 3 OS=Homo sapiens OX=9606 GN=DPYSL3 PE=1 SV=1
2 peptides

Sequence	Peptide Ion	Score	Hits	Mass	Charge	Tags	Conflicts	Modifications	In quantitation	Drift time (ms)	Average Normalised Abundances	
											CTR	MCD
DKGVNSFMVYMAYK	2119	4.81	1	1683.7632	3		0	[1] Hydroxyl DKNP|[5] Hydroxyl DKNP	yes	4.22	335.25	87.37
QIGDNLIVPGGVK	4110	4.79	1	1308.7241	2		0		yes	5.47	139.33	153.42

Tags	
	Anova p-value ≤ 0.05
	power>0.8
	q<0.05

P07196

Neurofilament light polypeptide OS=Homo sapiens OX=9606 GN=NEFL PE=1 SV=3
2 peptides

Sequence	Peptide Ion	Score	Hits	Mass	Charge	Tags	Conflicts	Modifications	In quantitation	Drift time (ms)	Average Normalised Abundances	
											CTR	MCD
LAAKNMQNAEEWFK	5968	4.49	1	1692.8318	2		0	[14] Methyl K	yes	6.23	246.22	27.37
VHELEQQNK	4820	4.98	1	1153.5695	2		0	[8] Hydroxyl DKNP|[9] Methyl K	yes	4.71	273.86	240.18

Tags	
	Anova p-value ≤ 0.05
	power>0.8
	q<0.05

O95571

Persulfide dioxygenase ETHE1_ mitochondrial OS=Homo sapiens OX=9606 GN=ETHE1 PE=1 SV=2
2 peptides

Sequence	Peptide Ion	Score	Hits	Mass	Charge	Tags	Conflicts	Modifications	In quantitation	Drift time (ms)	Average Normalised Abundances	
											CTR	MCD

Sequence	Peptide Ion	Score	Hits	Mass	Charge	Tags	Conflicts	Modifications	In quantitation	Drift time (ms)	Average Normalised Abundances	
											CTR	MCD
EAVLIDPVLETAPR	10312	5.32	1	1521.8327	2		0		yes	6.09	38.67	41.07
FALETR	5770	4.12	1	735.3933	1		0		yes	8.31	70.12	14.18

Tags	
	Anova p-value ≤ 0.05
	power>0.8
	q<0.05

P09211

Glutathione S-transferase P OS=Homo sapiens OX=9606 GN=GSTP1 PE=1 SV=2
2 peptides

Sequence	Peptide Ion	Score	Hits	Mass	Charge	Tags	Conflicts	Modifications	In quantitation	Drift time (ms)	Average Normalised Abundances	
											CTR	MCD
CKYISLIYTNYEAGK	3781	4.26	1	1835.9170	3		0	[1] Carbamidomethyl C|[15] Methyl K	yes	4.29	259.60	72.82
FQDGDLTLYQSNTILR	10567	5.07	1	1882.9321	2		0		yes	6.99	82.81	1.15

Tags	
	Anova p-value ≤ 0.05
	power>0.8
	q<0.05

Q96AC1

Fermitin family homolog 2 OS=Homo sapiens OX=9606 GN=FERMT2 PE=1 SV=1
2 peptides

Sequence	Peptide Ion	Score	Hits	Mass	Charge	Tags	Conflicts	Modifications	In quantitation	Drift time (ms)	Average Normalised Abundances	
											CTR	MCD
GCEVTPDVNISGQK	9155	4.89	1	1502.6968	2		0	[2] Carbamidomethyl C	yes	5.54	46.81	3.81
SKEESSGTPAHQMNLR	8695	3.94	1	1786.8179	3		0	[14] Hydroxyl DKNP	yes	4.22	63.98	90.50

Tags	
	Anova p-value ≤ 0.05
	power>0.8
	q<0.05

P16104

Histone H2AX OS=Homo sapiens OX=9606 GN=H2AX PE=1 SV=2
3 peptides

Sequence	Peptide Ion	Score	Hits	Mass	Charge	Tags	Conflicts	Modifications	In quantitation	Drift time (ms)	Average Normalised Abundances	
											CTR	MCD
AGLQFPVGR	212	8.52	15	943.5278	2		0		yes	3.88	7249.13	8346.32
AGLQFPVGR	14032	---	---	943.5205	1		0		yes	10.53	11.80	0.00
FPVGR	1444	0.00	2	574.3234	1		0		yes	6.79	361.48	226.30
QFPVGR	2317	0.00	2	702.3814	1		0		yes	7.83	237.92	128.69

Tags	
	Anova p-value ≤ 0.05
	power>0.8
	q<0.05

Q99456

Keratin_ type I cytoskeletal 12 OS=Homo sapiens OX=9606 GN=KRT12 PE=1 SV=1
2 peptides

Sequence	Peptide Ion	Score	Hits	Mass	Charge	Tags	Conflicts	Modifications	In quantitation	Drift time (ms)	Average Normalised Abundances	
											CTR	MCD
GTGTADASQSDYSK	313	4.02	1	1386.5922	2		0		yes	5.06	3919.36	1.32e+004
LELEIETYRR	346	4.31	1	1320.7095	3		0		yes	4.02	3626.79	9824.41

Tags	
	Anova p-value ≤ 0.05
	power>0.8
	q<0.05

Q2TBA0

Kelch-like protein 40 OS=Homo sapiens OX=9606 GN=KLHL40 PE=1 SV=2
1 peptide

Sequence	Peptide Ion	Score	Hits	Mass	Charge	Tags	Conflicts	Modifications	In quantitation	Drift time (ms)	Average Normalise Abundanc
											CTR
EEAVFEAVMRWAGSGDAEAQAER	197	3.88	1	2508.1245	4		0		yes	4.85	3209.97
EEAVFEAVMRWAGSGDAEAQAER	441	3.88	1	2508.1272	5		0		yes	4.16	1478.06
EEAVFEAVMRWAGSGDAEAQAER	3084	---	---	2508.1280	3		0		yes	5.40	65.31

Tags

	Anova p-value ≤ 0.05
	power>0.8
	q<0.05

P08590

Myosin light chain 3 OS=Homo sapiens OX=9606 GN=MYL3 PE=1 SV=3
1 peptide

Sequence	Peptide Ion	Score	Hits	Mass	Charge	Tags	Conflicts	Modifications	In quantitation	Drift time (ms)	Average Normalised Abundances	
											CTR	MCD
ALGQNPTQAEVLR	1571	7.69	2	1395.7445	2		0		yes	5.47	57.29	793.71

Tags	
	Anova p-value ≤ 0.05
	power>0.8
	q<0.05

P16520

Guanine nucleotide-binding protein G(I)/G(S)/G(T) subunit beta-3 OS=Homo sapiens OX=9606 GN=GNB3 PE=1 SV=1
1 peptide

Sequence	Peptide Ion	Score	Hits	Mass	Charge	Tags	Conflicts	Modifications	In quantitation	Drift time (ms)	Average Normalised Abundances	
											CTR	MCD
LLVSASQDGK	2294	7.30	3	1016.5453	2		0		yes	4.22	539.22	202.21

Tags	
	Anova p-value ≤ 0.05
	power>0.8
	q<0.05

A0A0A0MRZ8

Immunoglobulin kappa variable 3D-11 OS=Homo sapiens OX=9606 GN=IGKV3D-11 PE=3 SV=6
1 peptide

Sequence	Peptide Ion	Score	Hits	Mass	Charge	Tags	Conflicts	Modifications	In quantitation	Drift time (ms)	Average Normalised Abundances	
											CTR	MCD
LLIYDASNR	2476	7.25	4	1063.5660	2		0		yes	4.57	732.02	148.69

Tags

	Anova p-value ≤ 0.05
	power>0.8
	q<0.05

A0A0B4J1V1

Immunoglobulin heavy variable 3-21 OS=Homo sapiens OX=9606 GN=IGHV3-21 PE=1 SV=1
1 peptide

Sequence	Peptide Ion	Score	Hits	Mass	Charge	Tags	Conflicts	Modifications	In quantitation	Drift time (ms)	Average Normalised Abundances	
											CTR	MCD
AEDTAVYYCAR	4850	7.23	4	1317.5728	2		0	[9] Carbamidomethyl C	yes	5.12	440.61	101.13

Tags	
	Anova p-value ≤ 0.05
	power>0.8
	q<0.05

P48741

Putative heat shock 70 kDa protein 7 OS=Homo sapiens OX=9606 GN=HSPA7 PE=5 SV=2
1 peptide

Sequence	Peptide Ion	Score	Hits	Mass	Charge	Tags	Conflicts	Modifications	In quantitation	Drift time (ms)	Average Normalis Abundances	
											CTR	MCD
VLPIINEATAAAIAYGLDR	70	3.60	1	1970.0675	4		0		yes	4.50	2.54e+004	8.96e-
VLPIINEATAAAIAYGLDR	332	3.60	1	1970.0594	3		0		yes	5.47	4993.30	1.90e-
VLPIINEATAAAIAYGLDR	13293	3.60	1	1970.0561	4		0		yes	2.15	0.00	

Tags	
	Anova p-value ≤ 0.05
	power>0.8
	q<0.05

P52565

Rho GDP-dissociation inhibitor 1 OS=Homo sapiens OX=9606 GN=ARHGDIA PE=1 SV=3
1 peptide

Sequence	Peptide Ion	Score	Hits	Mass	Charge	Tags	Conflicts	Modifications	In quantitation	Drift time (ms)	Average Normalised Abundances	
											CTR	MCD
AEEYEFLTPVEEAPK	3250	7.17	2	1750.8237	2		0		yes	6.65	419.52	100.95

Tags	
	Anova p-value ≤ 0.05
	power>0.8
	q<0.05

Q14103

Heterogeneous nuclear ribonucleoprotein D0 OS=Homo sapiens OX=9606 GN=HNRNPD PE=1 SV=1
1 peptide

Sequence	Peptide Ion	Score	Hits	Mass	Charge	Tags	Conflicts	Modifications	In quantitation	Drift time (ms)	Average Normalised Abundances	
											CTR	MCD
IFVGGLSPDTPEEK	1936	7.15	2	1487.7468	2		0		yes	5.89	548.38	403.01

Tags	
	Anova p-value ≤ 0.05
	power>0.8
	q<0.05

P16444

Dipeptidase 1 OS=Homo sapiens OX=9606 GN=DPEP1 PE=1 SV=3
1 peptide

Sequence	Peptide Ion	Score	Hits	Mass	Charge	Tags	Conflicts	Modifications	In quantitation	Drift time (ms)	Average Normalised Abundances	
											CTR	MCD
AVGFGGDFDGVPR	3289	6.75	2	1292.6153	2		0		yes	5.12	580.79	107.70

Tags	
	Anova p-value ≤ 0.05
	power>0.8
	q<0.05

P51153

Ras-related protein Rab-13 OS=Homo sapiens OX=9606 GN=RAB13 PE=1 SV=1
1 peptide

Sequence	Peptide Ion	Score	Hits	Mass	Charge	Tags	Conflicts	Modifications	In quantitation	Drift time (ms)	Average Normalised Abundances	
											CTR	MCD
LLLIGDSGVGK	3540	6.75	2	1070.6301	2		0		yes	4.50	240.51	211.04

Tags	
	Anova p-value ≤ 0.05
	power>0.8
	q<0.05

P27348

14-3-3 protein theta OS=Homo sapiens OX=9606 GN=YWHAQ PE=1 SV=1
1 peptide

Sequence	Peptide Ion	Score	Hits	Mass	Charge	Tags	Conflicts	Modifications	In quantitation	Drift time (ms)	Average Normalised Abundances	
											CTR	MCD
AVTEQGAELSNEER	9689	6.61	1	1531.7039	2		0		yes	5.54	74.47	2.54

Tags	
	Anova p-value ≤ 0.05
	power>0.8
	q<0.05

Q9BVC6

Transmembrane protein 109 OS=Homo sapiens OX=9606 GN=TMEM109 PE=1 SV=1
1 peptide

Sequence	Peptide Ion	Score	Hits	Mass	Charge	Tags	Conflicts	Modifications	In quantitation	Drift time (ms)	Average Normalised Abundances	
											CTR	MCD
EAPVDVLTQIGR	2753	6.58	2	1296.6985	2		0		yes	5.06	373.49	277.22

Tags	
	Anova p-value ≤ 0.05
	power>0.8
	q<0.05

P01619

Immunoglobulin kappa variable 3-20 OS=Homo sapiens OX=9606 GN=IGKV3-20 PE=1 SV=2
1 peptide

Sequence	Peptide Ion	Score	Hits	Mass	Charge	Tags	Conflicts	Modifications	In quantitation	Drift time (ms)	Average Normalised Abundances	
											CTR	MCD
LLIYGASSR	3130	6.58	1	978.5398	2		0		yes	4.22	339.97	104.31

Tags

(red)	Anova p-value ≤ 0.05
(green)	power>0.8
(blue)	q<0.05

Q9BVQ7

Spermatogenesis-associated protein 5-like protein 1 OS=Homo sapiens OX=9606 GN=SPATA5L1 PE=1 SV=2
1 peptide

Sequence	Peptide Ion	Score	Hits	Mass	Charge	Tags	Conflicts	Modifications	In quantitation	Drift time (ms)	Average Normalised Abundances	
											CTR	MCD
GVLLAGPPGVGK	2198	6.56	2	1063.6356	2		0		yes	4.36	390.97	406.69

Tags	
(red)	Anova p-value ≤ 0.05
(green)	power>0.8
(blue)	q<0.05

Q9NVA2

Septin-11 OS=Homo sapiens OX=9606 GN=SEPTIN11 PE=1 SV=3
1 peptide

Sequence	Peptide Ion	Score	Hits	Mass	Charge	Tags	Conflicts	Modifications	In quantitation	Drift time (ms)	Average Normalised Abundances	
											CTR	MCD
SYELQESNVR	3426	6.54	1	1223.5785	2		0		yes	4.78	527.35	162.71

Tags	
(red)	Anova p-value ≤ 0.05
(green)	power>0.8
(blue)	q<0.05

Q15517

Corneodesmosin OS=Homo sapiens OX=9606 GN=CDSN PE=1 SV=3
1 peptide

Sequence	Peptide Ion	Score	Hits	Mass	Charge	Tags	Conflicts	Modifications	In quantitation	Drift time (ms)	Average Normalised Abundances	
											CTR	MCD
IILQPCGSK	1721	6.54	1	1014.5344	2		0	[6] Carbamidomethyl C	yes	4.16	1686.79	852.69

Tags	
	Anova p-value ≤ 0.05
	power>0.8
	q<0.05

P01889

HLA class I histocompatibility antigen_ B alpha chain OS=Homo sapiens OX=9606 GN=HLA-B PE=1 SV=3
1 peptide

Sequence	Peptide Ion	Score	Hits	Mass	Charge	Tags	Conflicts	Modifications	In quantitation	Drift time (ms)	Average Normalised Abundances	
											CTR	MCD
FDSDAASPR	4077	6.51	2	964.4290	2		0		yes	3.95	340.29	54.62

Tags	
	Anova p-value ≤ 0.05
	power>0.8
	q<0.05

P31944

Caspase-14 OS=Homo sapiens OX=9606 GN=CASP14 PE=1 SV=2
1 peptide

Sequence	Peptide Ion	Score	Hits	Mass	Charge	Tags	Conflicts	Modifications	In quantitation	Drift time (ms)	Average Normalised Abundances	
											CTR	MCD
TNPEIQSTLR	1715	6.45	1	1157.5940	2		0		yes	4.64	2145.05	947.15

Tags	
	Anova p-value ≤ 0.05
	power>0.8
	q<0.05

Q86UL8

Membrane-associated guanylate kinase_ WW and PDZ domain-containing protein 2 OS=Homo sapiens OX=9606 GN=MAGI2 PE=1 SV=3
1 peptide

Sequence	Peptide Ion	Score	Hits	Mass	Charge	Tags	Conflicts	Modifications	In quantitation	Drift time (ms)	Average Normalised Abundances	
											CTR	MCD
APGLAAADAADAAR	3912	6.37	1	1239.6156	2		0		yes	4.92	239.55	51.53

Tags	
	Anova p-value ≤ 0.05
	power>0.8
	q<0.05

Q02252

Methylmalonate-semialdehyde dehydrogenase [acylating]_ mitochondrial OS=Homo sapiens OX=9606 GN=ALDH6A1 PE=1 SV=2
1 peptide

Sequence	Peptide Ion	Score	Hits	Mass	Charge	Tags	Conflicts	Modifications	In quantitation	Drift time (ms)	Average Normalised Abundances	
											CTR	MCD
AGEYIFER	3215	6.32	1	983.4738	2		0		yes	4.09	376.98	326.25

Tags	
	Anova p-value ≤ 0.05
	power>0.8
	q<0.05

P52209

6-phosphogluconate dehydrogenase_ decarboxylating OS=Homo sapiens OX=9606 GN=PGD PE=1 SV=3
1 peptide

Sequence	Peptide Ion	Score	Hits	Mass	Charge	Tags	Conflicts	Modifications	In quantitation	Drift time (ms)	Average Normalised Abundances	
											CTR	MCD
GILFVGSGVSGGEEGAR	6249	6.22	2	1590.7911	2		0		yes	5.96	148.66	32.80

Tags	
	Anova p-value ≤ 0.05
	power>0.8
	q<0.05

P47755

F-actin-capping protein subunit alpha-2 OS=Homo sapiens OX=9606 GN=CAPZA2 PE=1 SV=3
1 peptide

Sequence	Peptide Ion	Score	Hits	Mass	Charge	Tags	Conflicts	Modifications	In quantitation	Drift time (ms)	Average Normalised Abundances	
											CTR	MCD
LLLNNDNLLR	1930	6.18	3	1196.6812	2		0		yes	4.85	481.62	470.55

Tags	
	Anova p-value ≤ 0.05
	power>0.8
	q<0.05

P01877

Immunoglobulin heavy constant alpha 2 OS=Homo sapiens OX=9606 GN=IGHA2 PE=1 SV=4
1 peptide

Sequence	Peptide Ion	Score	Hits	Mass	Charge	Tags	Conflicts	Modifications	In quantitation	Drift time (ms)	Average Normalised Abundances	
											CTR	MCD
DASGATFTWTPSSGK	8051	6.13	1	1511.6833	2		0		yes	5.54	168.91	47.53

Tags	
	Anova p-value ≤ 0.05
	power>0.8
	q<0.05

P02748

Complement component C9 OS=Homo sapiens OX=9606 GN=C9 PE=1 SV=2
1 peptide

Sequence	Peptide Ion	Score	Hits	Mass	Charge	Tags	Conflicts	Modifications	In quantitation	Drift time (ms)	Average Normalised Abundances	
											CTR	MCD
AIEDYINEFSVR	4374	6.11	2	1454.7031	2		0		yes	5.61	264.15	97.46

Tags	
	Anova p-value ≤ 0.05
	power>0.8
	q<0.05

P18124

60S ribosomal protein L7 OS=Homo sapiens OX=9606 GN=RPL7 PE=1 SV=1
1 peptide

Sequence	Peptide Ion	Score	Hits	Mass	Charge	Tags	Conflicts	Modifications	In quantitation	Drift time (ms)	Average Normalised Abundances	
											CTR	MCD
IALTDNALIAR	2857	6.10	1	1169.6724	2		0		yes	4.78	275.13	133.66

Tags

	Anova p-value ≤ 0.05
	power>0.8
	q<0.05

Q969L2

Protein MAL2 OS=Homo sapiens OX=9606 GN=MAL2 PE=1 SV=1
1 peptide

Sequence	Peptide Ion	Score	Hits	Mass	Charge	Tags	Conflicts	Modifications	In quantitation	Drift time (ms)	Average Normalised Abundances	
											CTR	MCD
VTLPAGPDILR	2264	6.07	2	1150.6699	2		0		yes	4.64	336.00	120.76

Tags	
	Anova p-value ≤ 0.05
	power>0.8
	q<0.05

Q9BVM4

Gamma-glutamylaminecyclotransferase OS=Homo sapiens OX=9606 GN=GGACT PE=1 SV=2
1 peptide

Sequence	Peptide Ion	Score	Hits	Mass	Charge	Tags	Conflicts	Modifications	In quantitation	Drift time (ms)	Average Normalised Abundances	
											CTR	MCD
LVEGEVYAVDER	4644	6.06	2	1377.6713	2		0		yes	5.33	212.03	51.87

Tags	
	Anova p-value ≤ 0.05
	power>0.8
	q<0.05

Q00796

Sorbitol dehydrogenase OS=Homo sapiens OX=9606 GN=SORD PE=1 SV=4
1 peptide

Sequence	Peptide Ion	Score	Hits	Mass	Charge	Tags	Conflicts	Modifications	In quantitation	Drift time (ms)	Average Normalised Abundances	
											CTR	MCD
VAIEPGAPR	3937	6.05	1	908.4999	2		0		yes	3.81	295.24	118.10

Tags	
	Anova p-value ≤ 0.05

	power>0.8
	q<0.05

P14649

Myosin light chain 6B OS=Homo sapiens OX=9606 GN=MYL6B PE=1 SV=1
1 peptide

Sequence	Peptide Ion	Score	Hits	Mass	Charge	Tags	Conflicts	Modifications	In quantitation	Drift time (ms)	Average Normalised Abundances	
											CTR	MCD
+KDVPVK	295	6.03	1	726.4265	1		0	[N-term] Formyl K N-TERM|[6] Methyl K	yes	8.38	3277.60	3337.28

Tags	
	Anova p-value ≤ 0.05
	power>0.8
	q<0.05

P13489

Ribonuclease inhibitor OS=Homo sapiens OX=9606 GN=RNH1 PE=1 SV=2
1 peptide

Sequence	Peptide Ion	Score	Hits	Mass	Charge	Tags	Conflicts	Modifications	In quantitation	Drift time (ms)	Average Normalised Abundances	
											CTR	MCD
VNPALAELNLR	3439	6.00	1	1208.6807	2		0		yes	4.92	207.60	57.58

Tags	
	Anova p-value ≤ 0.05
	power>0.8
	q<0.05

Q9H299

SH3 domain-binding glutamic acid-rich-like protein 3 OS=Homo sapiens OX=9606 GN=SH3BGRL3 PE=1 SV=1
1 peptide

Sequence	Peptide Ion	Score	Hits	Mass	Charge	Tags	Conflicts	Modifications	In quantitation	Drift time (ms)	Average Normalised Abundances	
											CTR	MCD
VYSTSVTGSR	5300	5.98	1	1055.5172	2		0		yes	4.29	306.53	82.07

Tags	
	Anova p-value ≤ 0.05
	power>0.8
	q<0.05

P62942

Peptidyl-prolyl cis-trans isomerase FKBP1A OS=Homo sapiens OX=9606 GN=FKBP1A PE=1 SV=2
1 peptide

Sequence	Peptide Ion	Score	Hits	Mass	Charge	Tags	Conflicts	Modifications	In quantitation	Drift time (ms)	Average Normalised Abundances	
											CTR	MCD
GVQVETISPGDGR	7021	5.94	1	1313.6502	2		0		yes	5.06	246.87	141.58

Tags	
	Anova p-value ≤ 0.05
	power>0.8
	q<0.05

P35237

Serpin B6 OS=Homo sapiens OX=9606 GN=SERPINB6 PE=1 SV=3
1 peptide

Sequence	Peptide Ion	Score	Hits	Mass	Charge	Tags	Conflicts	Modifications	In quantitation	Drift time (ms)	Average Normalised Abundances	
											CTR	MCD
IAELLSPGSVDPLTR	6119	5.94	1	1566.8531	2		0		yes	6.16	136.06	6.18

Tags	
	Anova p-value ≤ 0.05
	power>0.8
	q<0.05

P61158

Actin-related protein 3 OS=Homo sapiens OX=9606 GN=ACTR3 PE=1 SV=3
1 peptide

Sequence	Peptide Ion	Score	Hits	Mass	Charge	Tags	Conflicts	Modifications	In quantitation	Drift time (ms)	Average Normalised Abundances	
											CTR	MCD
LSEELSGGR	4853	5.92	1	946.4716	2		0		yes	3.88	399.99	57.49

Tags

	Anova p-value ≤ 0.05
	power>0.8
	q<0.05

P50453

Serpin B9 OS=Homo sapiens OX=9606 GN=SERPINB9 PE=1 SV=1
1 peptide

Sequence	Peptide Ion	Score	Hits	Mass	Charge	Tags	Conflicts	Modifications	In quantitation	Drift time (ms)	Average Normalised Abundances	
											CTR	MCD
AQLLELPYAR	7585	5.83	1	1172.6406	2		0		yes	4.78	105.38	7.25

Tags	
	Anova p-value ≤ 0.05
	power>0.8
	q<0.05

Q92597

Protein NDRG1 OS=Homo sapiens OX=9606 GN=NDRG1 PE=1 SV=1
1 peptide

Sequence	Peptide Ion	Score	Hits	Mass	Charge	Tags	Conflicts	Modifications	In quantitation	Drift time (ms)	Average Normalised Abundances	
											CTR	MCD
TASGSSVTSLDGTR	5173	5.82	2	1337.6359	2		0		yes	5.12	329.30	434.82

Tags	
	Anova p-value ≤ 0.05
	power>0.8
	q<0.05

Q96A08

Histone H2B type 1-A OS=Homo sapiens OX=9606 GN=H2BC1 PE=1 SV=3
1 peptide

Sequence	Peptide Ion	Score	Hits	Mass	Charge	Tags	Conflicts	Modifications	In quantitation	Drift time (ms)	Average Normalised Abundances	
											CTR	MCD
KESYSIYIYK	2705	5.82	1	1292.6500	2		0		yes	5.06	878.89	436.93

Tags	
	Anova p-value ≤ 0.05

■	power>0.8
■	q<0.05

P45880

Voltage-dependent anion-selective channel protein 2 OS=Homo sapiens OX=9606 GN=VDAC2 PE=1 SV=2
1 peptide

Sequence	Peptide Ion	Score	Hits	Mass	Charge	Tags	Conflicts	Modifications	In quantitation	Drift time (ms)	Average Normalised Abundances	
											CTR	MCD
YQLDPTASISAK	4144	5.79	1	1292.6531	2	■	0		yes	5.12	373.54	124.84

Tags	
■	Anova p-value ≤ 0.05
■	power>0.8
■	q<0.05

P07858

Cathepsin B OS=Homo sapiens OX=9606 GN=CTSB PE=1 SV=3
1 peptide

Sequence	Peptide Ion	Score	Hits	Mass	Charge	Tags	Conflicts	Modifications	In quantitation	Drift time (ms)	Average Normalised Abundances	
											CTR	MCD
LPASFDAR	747	5.72	1	875.4459	2	■	0		yes	3.81	2962.50	1506.91

Tags	
■	Anova p-value ≤ 0.05
■	power>0.8
■	q<0.05

P02766

Transthyretin OS=Homo sapiens OX=9606 GN=TTR PE=1 SV=1
1 peptide

Sequence	Peptide Ion	Score	Hits	Mass	Charge	Tags	Conflicts	Modifications	In quantitation	Drift time (ms)	Average Normalised Abundances	
											CTR	MCD
AADDTWEPFASGK	7483	5.72	1	1393.6127	2	■	0		yes	5.19	169.47	65.60

Tags	
■	Anova p-value ≤ 0.05

	power>0.8
	q<0.05

P59910

DnaJ homolog subfamily B member 13 OS=Homo sapiens OX=9606 GN=DNAJB13 PE=1 SV=1
1 peptide

Sequence	Peptide Ion	Score	Hits	Mass	Charge	Tags	Conflicts	Modifications	In quantitation	Drift time (ms)	Average Normalised Abundances	
											CTR	MCD
SNEPSSAEIFR	226	5.66	1	1235.5805	2		0		yes	5.33	4241.96	1.25e+004

Tags	
	Anova p-value ≤ 0.05
	power>0.8
	q<0.05

P61604

10 kDa heat shock protein_ mitochondrial OS=Homo sapiens OX=9606 GN=HSPE1 PE=1 SV=2
1 peptide

Sequence	Peptide Ion	Score	Hits	Mass	Charge	Tags	Conflicts	Modifications	In quantitation	Drift time (ms)	Average Normalised Abundances	
											CTR	MCD
VLLPEYGGTK	2478	5.62	1	1075.5829	2		0		yes	4.43	464.51	504.67

Tags	
	Anova p-value ≤ 0.05
	power>0.8
	q<0.05

P12035

Keratin_ type II cytoskeletal 3 OS=Homo sapiens OX=9606 GN=KRT3 PE=1 SV=3
1 peptide

Sequence	Peptide Ion	Score	Hits	Mass	Charge	Tags	Conflicts	Modifications	In quantitation	Drift time (ms)	Average Normalised Abundances	
											CTR	MCD
KDVDSAYMNK	3977	5.62	1	1169.5292	2		0		yes	4.50	598.93	114.72

Tags	
	Anova p-value ≤ 0.05
	power>0.8

	q<0.05

P61106

Ras-related protein Rab-14 OS=Homo sapiens OX=9606 GN=RAB14 PE=1 SV=4
1 peptide

Sequence	Peptide Ion	Score	Hits	Mass	Charge	Tags	Conflicts	Modifications	In quantitation	Drift time (ms)	Average Normalised Abundances	
											CTR	MCD
LTSEPQPQR	7687	5.57	1	1054.5357	2		0		yes	4.09	44.13	1.11

Tags	
	Anova p-value ≤ 0.05
	power>0.8
	q<0.05

P51148

Ras-related protein Rab-5C OS=Homo sapiens OX=9606 GN=RAB5C PE=1 SV=2
1 peptide

Sequence	Peptide Ion	Score	Hits	Mass	Charge	Tags	Conflicts	Modifications	In quantitation	Drift time (ms)	Average Normalised Abundances	
											CTR	MCD
GVDLQENNPASR	6999	5.55	1	1298.6181	2		0		yes	5.12	128.50	46.32

Tags	
	Anova p-value ≤ 0.05
	power>0.8
	q<0.05

A0A075B6P5

Immunoglobulin kappa variable 2-28 OS=Homo sapiens OX=9606 GN=IGKV2-28 PE=3 SV=1
1 peptide

Sequence	Peptide Ion	Score	Hits	Mass	Charge	Tags	Conflicts	Modifications	In quantitation	Drift time (ms)	Average Normalised Abundances	
											CTR	MCD
FSGSGSGTDFTLK	3825	5.55	2	1302.6142	2		0		yes	5.06	554.05	140.14

Tags	
	Anova p-value ≤ 0.05
	power>0.8
	q<0.05

P22528

Cornifin-B OS=Homo sapiens OX=9606 GN=SPRR1B PE=1 SV=2
1 peptide

Sequence	Peptide Ion	Score	Hits	Mass	Charge	Tags	Conflicts	Modifications	In quantitation	Drift time (ms)	Average Normalised Abundances	
											CTR	MCD
VPEPC̄QPK	3407	5.53	1	953.4619	2		0	[5] Carbamidomethyl C	yes	4.09	759.85	129.43

Tags	
	Anova p-value ≤ 0.05
	power>0.8
	q<0.05

Q9BQI9

Nuclear receptor-interacting protein 2 OS=Homo sapiens OX=9606 GN=NRIP2 PE=1 SV=3
1 peptide

Sequence	Peptide Ion	Score	Hits	Mass	Charge	Tags	Conflicts	Modifications	In quantitation	Drift time (ms)	Average Normalised Abundances	
											CTR	MCD
VAVDTGTQYNR	7376	5.49	1	1222.5866	2		0		yes	4.71	71.53	21.17

Tags	
	Anova p-value ≤ 0.05
	power>0.8
	q<0.05

Q14894

Ketimine reductase mu-crystallin OS=Homo sapiens OX=9606 GN=CRYM PE=1 SV=1
1 peptide

Sequence	Peptide Ion	Score	Hits	Mass	Charge	Tags	Conflicts	Modifications	In quantitation	Drift time (ms)	Average Normalised Abundances	
											CTR	MCD
LVTFYEDR	5970	5.47	1	1041.5209	2		0		yes	4.36	338.30	96.42

Tags	
	Anova p-value ≤ 0.05
	power>0.8

	q<0.05

P07339

Cathepsin D OS=Homo sapiens OX=9606 GN=CTSD PE=1 SV=1
1 peptide

Sequence	Peptide Ion	Score	Hits	Mass	Charge	Tags	Conflicts	Modifications	In quantitation	Drift time (ms)	Average Normalised Abundances	
											CTR	MCD
VSTLPAITLK	4113	5.47	1	1041.6416	2		0		yes	4.36	400.26	268.46

Tags	
	Anova p-value ≤ 0.05
	power>0.8
	q<0.05

P28072

Proteasome subunit beta type-6 OS=Homo sapiens OX=9606 GN=PSMB6 PE=1 SV=4
1 peptide

Sequence	Peptide Ion	Score	Hits	Mass	Charge	Tags	Conflicts	Modifications	In quantitation	Drift time (ms)	Average Normalised Abundances	
											CTR	MCD
QVLLGDQIPK	686	5.44	2	1109.6436	2		0		yes	4.64	1207.01	3410.32

Tags	
	Anova p-value ≤ 0.05
	power>0.8
	q<0.05

P62750

60S ribosomal protein L23a OS=Homo sapiens OX=9606 GN=RPL23A PE=1 SV=1
1 peptide

Sequence	Peptide Ion	Score	Hits	Mass	Charge	Tags	Conflicts	Modifications	In quantitation	Drift time (ms)	Average Normalised Abundances	
											CTR	MCD
LAPDYDALDVANK	3265	5.40	1	1403.6949	2		0		yes	5.47	306.58	266.00

Tags	
	Anova p-value ≤ 0.05
	power>0.8
	q<0.05

P62258

14-3-3 protein epsilon OS=Homo sapiens OX=9606 GN=YWHAE PE=1 SV=1
1 peptide

Sequence	Peptide Ion	Score	Hits	Mass	Charge	Tags	Conflicts	Modifications	In quantitation	Drift time (ms)	Average Normalised Abundances	
											CTR	MCD
VAGMDVELTVEER	4963	5.39	2	1462.6977	2		0	[5] Hydroxyl DKNP	yes	5.75	821.16	174.45

Tags	
	Anova p-value ≤ 0.05
	power>0.8
	q<0.05

Q2M2I5

Keratin_ type I cytoskeletal 24 OS=Homo sapiens OX=9606 GN=KRT24 PE=1 SV=1
1 peptide

Sequence	Peptide Ion	Score	Hits	Mass	Charge	Tags	Conflicts	Modifications	In quantitation	Drift time (ms)	Average Normalised Abundances	
											CTR	MCD
VVSSQVSSISEVK	11	5.38	1	1347.7387	2		0		yes	5.33	4.94e+004	1.62e+005

Tags	
	Anova p-value ≤ 0.05
	power>0.8
	q<0.05

P14136

Glial fibrillary acidic protein OS=Homo sapiens OX=9606 GN=GFAP PE=1 SV=1
1 peptide

Sequence	Peptide Ion	Score	Hits	Mass	Charge	Tags	Conflicts	Modifications	In quantitation	Drift time (ms)	Average Normalised Abundances	
											CTR	MCD
HLQEYQDLLNVK	6477	5.37	1	1498.7703	2		0		yes	6.09	94.19	3.52
HLQEYQDLLNVK	13651	---	---	1498.7810	3		0		yes	3.95	19.09	0.08

Tags	
	Anova p-value ≤ 0.05
	power>0.8

	q<0.05

O75971

snRNA-activating protein complex subunit 5 OS=Homo sapiens OX=9606 GN=SNAPC5 PE=1 SV=1
1 peptide

Sequence	Peptide Ion	Score	Hits	Mass	Charge	Tags	Conflicts	Modifications	In quantitation	Drift time (ms)	Average Normalised Abundances	
											CTR	MCD
VEELALQSMISSRR	12	5.36	2	1617.8578	3		0		yes	4.29	1.17e+005	3.82e+005

Tags	
	Anova p-value ≤ 0.05
	power>0.8
	q<0.05

P19013

Keratin_ type II cytoskeletal 4 OS=Homo sapiens OX=9606 GN=KRT4 PE=1 SV=5
1 peptide

Sequence	Peptide Ion	Score	Hits	Mass	Charge	Tags	Conflicts	Modifications	In quantitation	Drift time (ms)	Average Normalised Abundances	
											CTR	MCD
VELEAALQQAK	457	5.34	1	1198.6560	2		0		yes	4.85	3045.39	1730.95

Tags	
	Anova p-value ≤ 0.05
	power>0.8
	q<0.05

P47914

60S ribosomal protein L29 OS=Homo sapiens OX=9606 GN=RPL29 PE=1 SV=2
1 peptide

Sequence	Peptide Ion	Score	Hits	Mass	Charge	Tags	Conflicts	Modifications	In quantitation	Drift time (ms)	Average Normalised Abundances	
											CTR	MCD
AQAAAPASVPAQAPK	4474	5.28	1	1376.7358	2		0		yes	5.61	128.70	91.38

Tags	
	Anova p-value ≤ 0.05
	power>0.8
	q<0.05

P04259

Keratin_ type II cytoskeletal 6B OS=Homo sapiens OX=9606 GN=KRT6B PE=1 SV=5
1 peptide

Sequence	Peptide Ion	Score	Hits	Mass	Charge	Tags	Conflicts	Modifications	In quantitation	Drift time (ms)	Average Normalised Abundances	
											CTR	MCD
ATGGGLSSVGGGSSTIK	2491	5.28	1	1434.7526	3		0		yes	3.88	1282.66	184.63
ATGGGLSSVGGGSSTIK	7506	---	---	1434.7365	2		0		yes	5.82	309.48	10.29

Tags	
	Anova p-value ≤ 0.05
	power>0.8
	q<0.05

P07305

Histone H1.0 OS=Homo sapiens OX=9606 GN=H1-0 PE=1 SV=3
1 peptide

Sequence	Peptide Ion	Score	Hits	Mass	Charge	Tags	Conflicts	Modifications	In quantitation	Drift time (ms)	Average Normalised Abundances	
											CTR	MCD
GVGASGSFR	2828	5.27	1	836.4027	2		0		yes	3.53	1008.62	431.01

Tags	
	Anova p-value ≤ 0.05
	power>0.8
	q<0.05

P29401

Transketolase OS=Homo sapiens OX=9606 GN=TKT PE=1 SV=3
1 peptide

Sequence	Peptide Ion	Score	Hits	Mass	Charge	Tags	Conflicts	Modifications	In quantitation	Drift time (ms)	Average Normalised Abundances	
											CTR	MCD
SVPTSTVFYPSDGVATEK	6156	5.24	1	1883.9069	2		0		yes	7.06	153.05	24.82

Tags	
	Anova p-value ≤ 0.05
	power>0.8
	q<0.05

P02792

Ferritin light chain OS=Homo sapiens OX=9606 GN=FTL PE=1 SV=2
1 peptide

Sequence	Peptide Ion	Score	Hits	Mass	Charge	Tags	Conflicts	Modifications	In quantitation	Drift time (ms)	Average Normalised Abundances	
											CTR	MCD
LGGPEAGLGEYLFER	4295	5.17	1	1606.7946	2		0		yes	6.30	483.73	331.07

Tags	
	Anova p-value ≤ 0.05
	power>0.8
	q<0.05

Q9H0W9

Ester hydrolase C11orf54 OS=Homo sapiens OX=9606 GN=C11orf54 PE=1 SV=1
1 peptide

Sequence	Peptide Ion	Score	Hits	Mass	Charge	Tags	Conflicts	Modifications	In quantitation	Drift time (ms)	Average Normalised Abundances	
											CTR	MCD
DPGFDLR	2295	5.04	1	818.3932	2		0		yes	3.74	650.47	105.86

Tags	
	Anova p-value ≤ 0.05
	power>0.8
	q<0.05

P69892

Hemoglobin subunit gamma-2 OS=Homo sapiens OX=9606 GN=HBG2 PE=1 SV=2
3 peptides

Sequence	Peptide Ion	Score	Hits	Mass	Charge	Tags	Conflicts	Modifications	In quantitation	Drift time (ms)	Average Normalised Abundances	
											CTR	MCD
EFTPEVQASWQK	2037	5.04	1	1464.6841	2		0	[4] Hydroxyl DKNP	yes	5.19	1541.83	396.62
VVYPWTQR	7115	0.00	1	1047.5449	1		0		yes	11.50	38.92	0.55
VYPWTQR	6371	0.00	1	948.4794	1		0		yes	10.46	76.00	13.52

Tags	
	Anova p-value ≤ 0.05

	power>0.8
	q<0.05

P02008

Hemoglobin subunit zeta OS=Homo sapiens OX=9606 GN=HBZ PE=1 SV=2
1 peptide

Sequence	Peptide Ion	Score	Hits	Mass	Charge	Tags	Conflicts	Modifications	In quantitation	Drift time (ms)	Average Normalised Abundances	
											CTR	MCD
LSELHAYILRVDPVNFK	17	5.00	1	2061.0841	3		0	[12] Hydroxyl DKNP|[13] Hydroxyl DKNP|[15] Hydroxyl DKNP	yes	4.64	8.60e+004	3.48e+0

Tags	
	Anova p-value ≤ 0.05
	power>0.8
	q<0.05

Q9H082

Ras-related protein Rab-33B OS=Homo sapiens OX=9606 GN=RAB33B PE=1 SV=1
1 peptide

Sequence	Peptide Ion	Score	Hits	Mass	Charge	Tags	Conflicts	Modifications	In quantitation	Drift time (ms)	Average Normalised Abundances	
											CTR	MCD
AVEIDGERIK	4804	4.91	1	1144.5988	2		0	[10] Hydroxyl DKNP	yes	4.64	237.13	84.30

Tags	
	Anova p-value ≤ 0.05
	power>0.8
	q<0.05

P08311

Cathepsin G OS=Homo sapiens OX=9606 GN=CTSG PE=1 SV=2
1 peptide

Sequence	Peptide Ion	Score	Hits	Mass	Charge	Tags	Conflicts	Modifications	In quantitation	Drift time (ms)	Average Normalised Abundances	
											CTR	MCD
GTDTLREVQLR	5048	---	---	1286.7108	3		0		yes	3.60	90.31	42.47
GTDTLREVQLR	5123	4.90	1	1286.6747	2		0		yes	5.26	209.48	89.95

Tags	
	Anova p-value ≤ 0.05
	power>0.8
	q<0.05

P04350

Tubulin beta-4A chain OS=Homo sapiens OX=9606 GN=TUBB4A PE=1 SV=2
1 peptide

Sequence	Peptide Ion	Score	Hits	Mass	Charge	Tags	Conflicts	Modifications	In quantitation	Drift time (ms)	Average Normalised Abundances	
											CTR	MCD
GHYTEGAELVDAVLDVVRK	9153	4.83	1	2086.0631	3		0	[11] Hydroxyl DKNP	yes	5.47	55.02	2.76
GHYTEGAELVDAVLDVVRK	12139	---	---	2086.0705	4		0	[11] Hydroxyl DKNP	yes	4.22	26.16	1.91

Tags	
	Anova p-value ≤ 0.05
	power>0.8
	q<0.05

Q58FF8

Putative heat shock protein HSP 90-beta 2 OS=Homo sapiens OX=9606 GN=HSP90AB2P PE=1 SV=2
1 peptide

Sequence	Peptide Ion	Score	Hits	Mass	Charge	Tags	Conflicts	Modifications	In quantitation	Drift time (ms)	Average Normalised Abundances	
											CTR	MCD
PEEVHLGEK	2000	4.77	1	1036.5299	2		0		yes	4.02	777.19	287.58

Tags	
	Anova p-value ≤ 0.05
	power>0.8
	q<0.05

A0A075B6H7

Probable non-functional immunoglobulin kappa variable 3-7 OS=Homo sapiens OX=9606 GN=IGKV3-7 PE=1 SV=1
1 peptide

Sequence	Peptide Ion	Score	Hits	Mass	Charge	Tags	Conflicts	Modifications	In quantitation	Drift time (ms)	Average Normalised Abundances	
											CTR	MCD
EIVMTQSPPTLSLSPGER	7507	4.63	1	1956.9737	3		0	[4] Oxidation M	yes	4.92	83.75	19.28

Tags	
	Anova p-value ≤ 0.05
	power>0.8
	q<0.05

P21695

Glycerol-3-phosphate dehydrogenase [NAD(+)]_ cytoplasmic OS=Homo sapiens OX=9606 GN=GPD1 PE=1 SV=4
1 peptide

Sequence	Peptide Ion	Score	Hits	Mass	Charge	Tags	Conflicts	Modifications	In quantitation	Drift time (ms)	Average Normalised Abundances	
											CTR	MCD
VTMWVFEEDIGGKK	3394	4.62	1	1653.7961	2		0	[3] Oxidation M	yes	6.30	607.86	223.35

Tags	
	Anova p-value ≤ 0.05
	power>0.8
	q<0.05

Q9NZ72

Stathmin-3 OS=Homo sapiens OX=9606 GN=STMN3 PE=1 SV=3
1 peptide

Sequence	Peptide Ion	Score	Hits	Mass	Charge	Tags	Conflicts	Modifications	In quantitation	Drift time (ms)	Average Normalised Abundances	
											CTR	MCD
MASTISAYKEK	2776	4.38	1	1257.6236	2		0	[9] Hydroxyl DKNP|[9] Methyl K	yes	4.99	902.44	353.58

Tags	
	Anova p-value ≤ 0.05
	power>0.8
	q<0.05

P78417

Glutathione S-transferase omega-1 OS=Homo sapiens OX=9606 GN=GSTO1 PE=1 SV=2
1 peptide

Sequence	Peptide Ion	Score	Hits	Mass	Charge	Tags	Conflicts	Modifications	In quantitation	Drift time (ms)	Average Normalised Abundances	
											CTR	MCD
LLPDDPYEK	3764	4.29	1	1088.5500	2		0		yes	4.29	308.37	93.24

Tags	
	Anova p-value ≤ 0.05
	power>0.8
	q<0.05

Q3MJ62

Zinc finger and SCAN domain-containing protein 23 OS=Homo sapiens OX=9606 GN=ZSCAN23 PE=1 SV=1
1 peptide

Sequence	Peptide Ion	Score	Hits	Mass	Charge	Tags	Conflicts	Modifications	In quantitation	Drift time (ms)	Average Normalised Abundances	
											CTR	MCD
AFSQNAGLFHHLR	702	4.17	1	1512.7590	2		0	[5] Hydroxyl DKNP	yes	6.09	1091.53	7469.26
AFSQNAGLFHHLR	1297	---	---	1512.7592	4		0	[5] Hydroxyl DKNP	yes	3.39	371.41	2899.54

Tags	
	Anova p-value ≤ 0.05
	power>0.8
	q<0.05

Q9BUT1

3-hydroxybutyrate dehydrogenase type 2 OS=Homo sapiens OX=9606 GN=BDH2 PE=1 SV=2
1 peptide

Sequence	Peptide Ion	Score	Hits	Mass	Charge	Tags	Conflicts	Modifications	In quantitation	Drift time (ms)	Average Normalised Abundances	
											CTR	MCD
SGNIINMSSVASSVKGVVNR	554	4.13	1	2034.0569	3		0	[6] Hydroxyl DKNP	yes	5.82	1883.68	1.38e+

Tags	
	Anova p-value ≤ 0.05
	power>0.8
	q<0.05

P12882

Myosin-1 OS=Homo sapiens OX=9606 GN=MYH1 PE=1 SV=3
1 peptide

Sequence	Peptide Ion	Score	Hits	Mass	Charge	Tags	Conflicts	Modifications	In quantitation	Drift time (ms)	Average Normalised Abundances	
											CTR	MCD
LINDLTAQRAR	1461	3.84	1	1285.7169	2		0	[3] Hydroxyl DKNP	yes	5.19	821.52	1029.17

Tags	
	Anova p-value ≤ 0.05
	power>0.8
	q<0.05

Q9H2M3

S-methylmethionine--homocysteine S-methyltransferase BHMT2 OS=Homo sapiens OX=9606 GN=BHMT2 PE=1 SV=1
1 peptide

Sequence	Peptide Ion	Score	Hits	Mass	Charge	Tags	Conflicts	Modifications	In quantitation	Drift time (ms)	Average Normalised Abundances	
											CTR	MCD
LVKAGASIVGVNCR	217	3.53	1	1442.7874	2		0	[13] Carbamidomethyl C	yes	5.61	3090.94	1.48e+00[illegible]

Tags	
	Anova p-value ≤ 0.05
	power>0.8
	q<0.05

Q8N257

Histone H2B type 3-B OS=Homo sapiens OX=9606 GN=H2BU1 PE=1 SV=3
2 peptides

Sequence	Peptide Ion	Score	Hits	Mass	Charge	Tags	Conflicts	Modifications	In quantitation	Drift time (ms)	Average Normalised Abundances	
											CTR	MCD
LLPGELAK	1334	0.00	3	839.5112	1		0		yes	10.18	508.42	343.18
LLPGELAK	9603	---	---	839.5090	2		0		yes	3.74	45.61	24.47
LPGELAK	1597	---	---	726.4259	2		0		yes	3.46	378.97	287.67

Tags	
	Anova p-value ≤ 0.05
	power>0.8
	q<0.05

Q5TEC6

Histone HIST2H3PS2 OS=Homo sapiens OX=9606 GN=H3-2 PE=1 SV=1
2 peptides

Sequence	Peptide Ion	Score	Hits	Mass	Charge	Tags	Conflicts	Modifications	In quantitation	Drift time (ms)	Average Normalised Abundances	
											CTR	MCD
ELLIR	443	0.00	2	642.4071	1		0		yes	7.76	1591.29	1470.87
ELLIR	8771	---	---	642.4043	2		0		yes	3.39	41.24	24.23
STELLIR+	1101	0.00	2	812.4725	2		0	[C-term] Dehydrated	yes	3.88	762.87	857.01

Tags	
	Anova p-value ≤ 0.05
	power>0.8
	q<0.05

P48668

Keratin_ type II cytoskeletal 6C OS=Homo sapiens OX=9606 GN=KRT6C PE=1 SV=3
2 peptides

Sequence	Peptide Ion	Score	Hits	Mass	Charge	Tags	Conflicts	Modifications	In quantitation	Drift time (ms)	Average Normalised Abundances	
											CTR	MCD
EEIAQR	3226	0.00	1	744.3763	1		0		yes	7.96	611.95	113.06
YEEIAQR	10175	---	---	907.4382	2		0		yes	3.81	187.58	24.90
YEEIAQR	1034	0.00	2	907.4389	1		0		yes	10.11	2229.37	482.45

Tags	
	Anova p-value ≤ 0.05
	power>0.8
	q<0.05
